# Supplementary material for: Efficacy and safety of the early implementation of a multimodal rehabilitation program in mechanically ventilated patients: A randomized clinical trial protocol
Source: PLoS One. 2025 May 19;20(5):e0324335. doi: 10.1371/journal.pone.0324335 (PMC12088510; doi:10.1371/journal.pone.0324335)
Supplement: S1 Checklist — (PDF) [file pone.0324335.s001.pdf]

Asociación entre terapia multimodal temprana y días de ventilación mecánica en la unidad de cuidado intensivos de la Fundación Santa Fe de Bogotá: un ensayo clínico controlado aleatorizado

Investigadores principales:

- **Jorge Iván Alvarado Sánchez**
  Médico Anestesiólogo
  Departamento Medicina Crítica y Cuidado Intensivo
  Fundación Santa Fe de Bogotá
  Correo: Jorge.alvarado@fsfb.org.co

- **Laura María Castillo Morales**
  Departamento Medicina Crítica y Cuidado Intensivo
  Fundación Santa Fe de Bogotá
  Correo: laura.castillo@fsfb.org.co

Coinvestigadores:

- **Catherine Lissell Arévalo Guerrero**
  Instituto de Medicina del Ejercicio y rehabilitación
  Fundación Santa Fe de Bogotá
  Correo: Catherine.arevalo@fsfb.org.co

- **Miguel Leonardo Pulido Bobadilla**
  Instituto de Medicina del Ejercicio y rehabilitación
  Fundación Santa Fe de Bogotá
  Correo: miguel.pulido@fsfb.org.co

- **Diana Marcela Melo Rojas**
  Instituto de Medicina del Ejercicio y rehabilitación
  Fundación Santa Fe de Bogotá
  Correo: dianammelo1992@gmail.com

Asociación entre terapia multimodal temprana y días de ventilación mecánica en la unidad de cuidado intensivos de la Fundación Santa Fe de Bogotá: un ensayo clínico controlado aleatorizado.

Versión 6.0
Fundación Santa Fe de Bogotá
8 de abril de 2024

- **Ana Gabriela López Rubio**
  Neumología
  Fundación Santa Fe de Bogotá
  correo: aglopez904@gmail.com

- **Diana Carolina Ortiz Moreno**
  Neumología
  Fundación Santa Fe de Bogotá
  Correo: carolinao.21@hotmail.com

- **Paula Andrea Barreto Garzón**
  Instituto de Medicina del Ejercicio y rehabilitación
  Fundación Santa Fe de Bogotá
  Correo: paula.barreto@fsfb.org.co

- **Marisol Murillo**
  Instituto de Medicina física y rehabilitación
  Fundación Santa Fe de Bogotá
  Correo: marisol.murillo@fsfb.org.co

- **Sara Stefania Martínez**
  Instituto de Medicina física y rehabilitación
  Fundación Santa Fe de Bogotá
  Correo: sahara.martinez@fsfb.org.co

Asistentes de investigación:

- **María Valentina Stozitzky Ríos**
  Magister en epidemiología
  Departamento Medicina Crítica y Cuidado Intensivo
  Fundación Santa Fe de Bogotá
  Correo: valentinastoziztky@gmail.com

❖ **Andrea Valentina Montañez Nariño**
Médico general
Departamento de Medicina Crítica y Cuidado intensivo
Fundación Santa Fe de Bogotá
Correo: Montanez.andreav@gmail.com

❖ **Andrés Felipe Mora Salamanca**
Magister en Epidemiología
Departamento Medicina Crítica y Cuidado Intensivo
Fundación Santa Fe de Bogotá
Correo: af.mora1963@uniandes.edu.co

Contenido

1. Resumen del proyecto 5
2. Planteamiento del problema y justificación 10
3. Pregunta de investigación 11
4. Marco teórico y estado del arte 15
5. Objetivos 25
6. Metodología 26
6.1. Diseño del estudio 26
6.2. Población 26
6.3. Tamaño de la muestra 28
6.4. Muestreo y aleatorización 30
6.5. Intervención 30
6.6. Desenlaces 34
6.7. Mediciones 36
6.8 Pérdidas y Manejo de Datos Perdidos: 40
6.9. Criterios de adherencia al protocolo: 41
6.10. Procedimientos de seguimiento y seguridad: 41
6.11. Recolección de la información 43
6.12. Calidad de datos: error y sesgos 62
6.13. Plan de análisis estadístico 63
7. Consideraciones éticas 66
8. Alcances 68
9. Trayectoria de los investigadores 69
10. Cronograma de actividades 70
11. Presupuesto y financiación 71
12. Referencias 71

Asociación entre terapia multimodal temprana y días de ventilación mecánica en la unidad de cuidado intensivos de la Fundación Santa Fe de Bogotá: un ensayo clínico controlado aleatorizado.

Versión 6.0
Fundación Santa Fe de Bogotá
8 de abril de 2024

1. Resumen del proyecto

**TÍTULO**	Asociación entre terapia multimodal temprana y días de ventilación mecánica en la unidad de cuidado intensivos de la Fundación Santa Fe de Bogotá: un ensayo clínico controlado aleatorizado.
**PATROCINADOR**	No
**ORGANIZACIÓN DE FINANCIACIÓN**	No. El estudio no cuenta con financiación externa ni interna.
**PREGUNTA DE INVESTIGACIÓN**	¿El uso de terapia multimodal temprana disminuye los días de ventilación mecánica invasiva en pacientes adultos comparado con la terapia multimodal tardía?
**OBJETIVO GENERAL**	Evaluar la diferencia en días de ventilación mecánica invasiva entre la terapia multimodal temprana y la terapia multimodal tardía en la unidad de cuidado intensivo adulto de la Fundación Santa Fe de Bogotá
**OBJETIVOS ESPECÍFICOS**	Describir las características sociodemográficas y clínicas de los pacientes con ventilación mecánica invasiva en la Unidad de Cuidado Intensivo Adultos de la Fundación Santa Fe de Bogotá.<br>Evaluar la eficacia de la terapia multimodal temprana frente a la terapia multimodal tardía en términos de días libres de ventilación mecánica y otros desenlaces secundarios.<br>Establecer los factores sociodemográficos y clínicos que estén asociados con el tipo de terapia (temprana o tardía) y con el desenlace primario (días libres de ventilación mecánica) y los desenlaces secundarios.

	Evaluar la seguridad de la terapia multimodal temprana frente a la terapia multimodal tardía en términos de eventos adversos, eventos adversos serios, episodios de broncoaspiración y neumonía asociada al cuidado de la salud.<br><br>Evaluar el impacto de los diferentes subgrupos previamente definidos, como el tipo de falla respiratoria (hipoxémica o hipercápnica) y el tipo de pacientes (quirúrgicos, respiratorios, quemados, cardiovasculares), en los resultados del desenlace principal.
**NÚMERO DE SUJETOS**	37 por cada grupo. 74 en total.
**CRITERIO DE SELECCIÓN DE SUJETOS**	Todos los pacientes adultos ingresados a la unidad de cuidado intensivo después de la aprobación por el comité de ética que ingresen o requieran ventilación mecánica invasiva.
**DURACIÓN DE LA PARTICIPACIÓN DEL SUJETO Y DURACIÓN DEL ESTUDIO**	Un año.
**ESTADÍSTICA, PLAN PRIMARIO DE ANÁLISIS**	Se realizará una prueba de Shapiro-Wilk para verificar la distribución de las variables cuantitativas, teniendo en cuenta el número de pacientes incluidos. Los datos se presentarán como medias o medianas, acompañadas de su distribución estándar o rango intercuartílico, según corresponda. Las variables categóricas se describirán como frecuencias absolutas y relativas.<br><br>Para comparar las variables continuas entre los dos grupos (terapia temprana vs. tardía), se empleará una prueba T o la prueba U de Mann-Whitney, dependiendo de la distribución de los datos. Para las variables categóricas, se utilizará una prueba de χ2 o la prueba exacta de Fisher, según corresponda.

	El objetivo principal del estudio es evaluar la asociación entre el uso de terapia multimodal temprana y los días de ventilación mecánica invasiva. Para responder a esta pregunta, realizaremos un análisis de supervivencia y una regresión Cox. Se creará una variable de riesgo combinando el tiempo de ventilación mecánica invasiva y la extubación (si/no). Luego, se realizará un análisis de regresión Cox univariable con la variable de riesgo como la variable dependiente y el grupo de intervención (temprana vs tardía) como la variable independiente. Asimismo, se generará una curva de supervivencia y se analizarán las diferencias entre los dos grupos mediante una prueba de log-rank.<br><br>Para evaluar los factores relacionados con el tiempo de ventilación mecánica, se realizarán modelos de regresión Cox univariable para todas las variables incluidas en el estudio. Para determinar si las variables continuas se pueden evaluar de manera continua o como variables categóricas, se verificará el supuesto de linealidad a través del gráfico de residuos de Martingala. En caso de no cumplirse la linealidad, se estratificará la variable y se definirán puntos de corte según la apariencia del gráfico. Posteriormente, se incluirán aquellas variables con un valor p de significancia estadística (menor a 0.25) y aquellas variables clínicamente importantes (edad, sexo, delirium, etc.) en un modelo de regresión Cox multivariable. Se seleccionará el mejor modelo mediante un proceso de Backward Stepwise basado en los criterios de información de Akaike (AIC) y bayesiano (BIC).<br><br>Se evaluarán las variables que podrían actuar como factores de confusión mediante un segundo modelo de regresión

	múltiple. En este modelo, se retirará la variable potencialmente confusora y se calculará la pendiente o estimador resultante (estimación ajustada). Si existe un cambio mayor al 15%-20%, la variable confusora se incluirá en el modelo final. En caso contrario, se optará por el modelo más parsimonioso.<br><br>Para evaluar la interacción entre dos variables, se realizará otro modelo de regresión que incluirá una nueva variable que resulta de multiplicar las dos variables que potencialmente interactúan. Si el coeficiente de regresión de esta nueva variable tiene un nivel de significancia menor a 0.05, se concluye que existe interacción y dicha variable se incluirá en el modelo. Las variables con potencial de interacción se identificarán mediante la evaluación de los factores de confusión. Los investigadores también evaluarán las variables clínicas que puedan ser consideradas como variables de interacción o de confusión a través de la estrategia arriba descrita.<br><br>Se evaluará el cumplimiento del supuesto de proporcionalidad de riesgo mediante residuos de Schoenfeld y se analizarán los valores influyentes mediante gráficos de residuos versus observaciones. La multicolinealidad se evaluará a través de las curvas de matriz.<br><br>Usaremos modelos de regresión logística para valorar la relación entre nuestras variables dicotómicas, como extubación fallida, VMNI pos extubación, ventilación mecánica prolongada, traqueotomía y disfonía, y las otras variables estudiadas. Las

	asunciones de un modelo de regresión logística, como la linealidad en el registro de las probabilidades, independencia de errores, homogeneidad de varianza, ausencia de multicolinealidad, ausencia de valores atípicos y proporcionalidad de los efectos, serán evaluadas a través de análisis de residuos y diagnósticos gráficos, como el análisis de residuos de Pearson o el análisis de residuos de deviance.<br><br>Se empleará una estrategia de ajuste por multiplicidad en las pruebas estadísticas, como el método de Bonferroni, Holm-Bonferroni u otros métodos que controlen la tasa de falso descubrimiento (FDR), con el fin de mantener la tasa global de error tipo 1 en un nivel aceptable.<br><br>Realizaremos una exploración detallada a través de un análisis de sensibilidad de posibles interacciones entre los subgrupos identificados, según escala RASS, según la adherencia al protocolo y otras variables relevantes, con el objetivo de profundizar en la comprensión de cualquier influencia conjunta que pueda afectar los resultados del estudio. En caso de no identificar diferencias estadísticamente significativas mediante los análisis de regresión, se llevará a cabo un análisis pos-hoc para evaluar si el tamaño de la muestra reclutada fue adecuado para detectar posibles diferencias entre los subgrupos establecidos. Este análisis adicional permitirá ajustar la interpretación de los resultados y proporcionará información valiosa sobre la validez de las conclusiones obtenidas.<br><br>Por último, se establecerá un comité independiente de monitoreo de datos para llevar a cabo revisiones intermedias del tamaño de

	muestra, permitiendo ajustes en función de las variaciones en las tasas de eventos observadas durante el estudio, garantizando así la potencia estadística adecuada a lo largo de su ejecución.

2. Planteamiento del problema y justificación

Los pacientes bajo ventilación mecánica (VM) en la unidad de cuidados intensivos (UCI) presentan una elevada mortalidad hospitalaria, oscilando entre el 23% y el 51%. Esta mortalidad está vinculada al éxito en el destete y la incidencia de infecciones nosocomiales. La prolongación de la VM puede aumentar las complicaciones en pacientes críticamente enfermos, prolongando la inmovilidad y aumentando el riesgo de neumonía asociada a la ventilación, restringiendo aún más a los pacientes. Se sostiene que la movilidad temprana mejora los resultados relacionados con la VM, como la duración del ventilador y los días sin necesidad de ventilación, rompiendo el círculo vicioso de la VM prolongada y la inmovilización. Además, la rehabilitación temprana se relaciona con una disminución de la morbilidad y mortalidad, así como con complicaciones de la enfermedad, la duración de la estancia en la UCI, la estancia hospitalaria y la tasa de rehospitalización. Por tanto, el destete de la VM mediante rehabilitación temprana resulta crucial para mejorar el pronóstico de pacientes en la UCI.

A pesar de la creciente evidencia sobre la utilidad de la rehabilitación temprana para pacientes críticamente enfermos, los programas y el interés en los resultados varían entre estudios. Ante esta diversidad, los profesionales de la salud deben evaluar si distintos programas tienen un efecto terapéutico similar y garantizar que la rehabilitación prescrita brinde beneficios consistentes con la literatura previa. Además de la heterogeneidad entre los programas, diferentes países e instituciones enfrentan barreras para la implementación, como la escasez de personal capacitado en rehabilitación y una brecha cultural en la UCI. En casos de escasez de personal y en entornos con éticas conservadoras en la UCI, la rehabilitación temprana se adapta a un nivel sostenible, incluyendo programas modificados o visitas menos frecuentes.

En cuanto al síndrome postcuidados intensivos (PICS), se estableció como un síndrome que abarca limitaciones nuevas o empeoradas en el estado físico, cognitivo o de salud mental que persisten más allá de la hospitalización aguda. El objetivo es iniciar mejoras para los sobrevivientes de la UCI y sus familias a lo largo del continuo de atención. Desde la creación del PICS en 2010, estudios observacionales han evaluado factores asociados con este síndrome; sin embargo, pocos estudios de intervención han abordado su prevención. La fisioterapia con rehabilitación temprana se considera integral en el manejo multidisciplinario de pacientes en las UCIs. Aunque se ha demostrado que el ejercicio mejora la fuerza y la función en otras poblaciones, reduciendo la

inflamación y afectando al estrés oxidativo, aún no se ha realizado una revisión sistemática sobre la efectividad de la rehabilitación temprana para prevenir el PICS en pacientes de la UCI. El objetivo de la presente revisión sistemática es evaluar la efectividad de intervenciones rehabilitativas tempranas en la prevención del PICS en pacientes de la UCI.

En nuestro conocimiento actual, no existen estudios que hayan evaluado el impacto y la seguridad de la terapia física, terapia respiratoria, fonoaudiología y terapia ocupacional (terapia multimodal) de forma temprana (24 horas). Por lo tanto, nuestro objetivo es evaluar si la terapia multimodal puede reducir los días de ventilación mecánica invasiva en pacientes críticamente enfermos. Esta pregunta es relevante dado el creciente interés en la rehabilitación y mejora de resultados en estos pacientes. Al abordar esta pregunta, se espera contribuir al conocimiento sobre las mejores prácticas para la atención de pacientes críticamente enfermos y mejorar sus resultados a largo plazo.

3. Pregunta de investigación

¿El uso de terapia multimodal temprana disminuye los días de ventilación mecánica invasiva en pacientes adultos comparado con la terapia multimodal tardía?

Estrategia PICOT

Población	Adultos (≥ 18 años) hospitalizados en la unidad de cuidado intensivo adulto de la Fundación Santa Fe de Bogotá que requieran ventilación mecánica invasiva a través de un tubo endotraqueal durante un periodo mayor de 24 horas
Intervención	Terapia multimodal temprana (<24 horas desde la intubación) definida como el conjunto de maniobras terapéuticas que

	realiza el grupo de fisioterapia, fonoaudiología, terapia respiratoria y terapia ocupacional en el momento que se realiza la intubación orotraqueal. La intervención se realizará exclusivamente durante la estancia en UCI.
Comparador	Terapia multimodal tardía definida como la instauración de la terapia multimodal a los 3 días o más del inicio de la ventilación mecánica. El comparador difiere de la intervención en estudio únicamente en el momento (tiempo desde la intubación orotraqueal hasta el inicio de la terapia multimodal) de la instauración de la terapia.
Desenlaces	Eficacia<br>Desenlace primario: Número de días con ventilación mecánica invasiva en la UCI<br>Desenlaces secundarios:<br>• Días libres de ventilación mecánica hasta el día 28<br>• Días de delirium hasta el día 28<br>• Tiempo en UCI con delirium hasta el día 28<br>• Días de hospitalización con delirium hasta el día 28<br>• Días de sedación hasta el día 28<br>• Días libres de sedación hasta el día 28<br>• Indice de Barthel al egreso<br>• Días de estancia hospitalaria<br>• Tiempo de estancia en UCI

	• Mortalidad por cualquier causa hasta los 90 días<br>• Tiempo desde la intubación hasta el inicio de la terapia<br>• Fuerza muscular medido con la escala Medical research council (MRC)<br>• Disfagia a las 72 horas<br>• Tiempo hasta el inicio de vía oral<br>• Desarrollo de neumonía asociada al cuidado de la salud,<br>• Movilidad máxima medida por JH-HLM<br>• Fuerza prensil<br>• Falla de extubación<br>• Necesidad de ventilación mecánica no invasiva<br>• Función cognitiva medida mediante el Montreal Cognitive Assessment (MOCA)<br>• Test rápido de sensibilidad y destreza<br>Seguridad<br>Desenlaces<br>• Numero de eventos adversos<br>o Presión arterial alterada<br>o Arritmia cardiaca<br>o Desaturación de oxigeno<br>o Dolor o agitación<br>o Remoción de línea invasiva<br>o Gastrointestinal (nausea, vomito o diarrea)<br>o Taquipnea

	○ Estado neurológico alterado<br>• Número de eventos adversos serios<br>• Extubación no programada<br>• Neumonía asociada al cuidado de la salud<br>• Broncoaspiración
Tiempo	Tamización: La verificación de los criterios de inclusión y exclusión de los pacientes tomará máximo 24 horas posteriores a la intubación endotraqueal<br>Intervención: Para los dos grupos de estudio la terapia multimodal se aplicará exclusivamente mientras el paciente se encuentre en la UCI<br>Seguimiento:<br>En el inicio del estudio, se medirán las variables descritas en el punto 6.6.<br>Al momento del destete de soporte ventilatorio se medirán las siguientes variables: delta POCC, presión muscular, P0.1, cantidad de secreciones por tubo orotraqueal, índice de asincronías, NIF, % fuga, pico flujo de tos, medición de excursión diafragmática, medición de porcentaje de grosos diafragmático, y secreciones traqueobronquiales.<br><br>El día del alta hospitalaria se medirá las variables consignadas en el punto 6.6.<br>A los 90 días posteriores a la intubación del paciente se realizará la última medición de variables de acuerdo con el punto 6.6.

4. Marco teórico y estado del arte

En las últimas décadas, se ha visto un aumento en la demanda de unidades de cuidado intensivo (UCI) a nivel mundial; acompañado del ingreso a las mismas de pacientes más longevos, con patologías más complejas, previamente consideradas intratables. Los avances médicos, sociales y tecnológicos han permitido un aumento en la supervivencia de los pacientes que ingresan a UCI, con la posibilidad de ofrecer y brindar un soporte a diferentes órganos vitales. (1–3) En Reino Unido, la cantidad de pacientes ingresados a UCI casi se duplicó en el periodo comprendido entre 2009 y 2018. (3) El aumento en la estancia y el reingreso en las unidades de cuidados intensivos requiere el uso persistente de medidas e intervenciones invasivas en estos pacientes. Se estima que el incremento de la población añosa continúe y con él, el requerimiento de camas y cuidado en unidades de cuidado intensivo. (1–3)

Si bien se ha logrado extender el criterio de ingreso a las unidades, impartir cuidado a un mayor número de pacientes, y disminuir la mortalidad de estos, el advenimiento de nuevas tecnologías y el uso de medidas invasivas, entre las que se destaca la ventilación mecánica invasiva, acarrea a su vez riesgos inherentes a su uso. Como consecuencia, los pacientes en unidades de cuidado intensivo, incluso tras recuperarse de la patología aguda, tienen mayor morbimortalidad y presentan deterioro en múltiples aspectos de su vida, los cuales pueden persistir hasta años después de su estancia hospitalaria. (3) Esto ha desencadenado una preocupación creciente por rehabilitar adecuadamente estos pacientes y velar por su recuperación global, comprendiendo aspectos sociales, físicos, psicológicos y en su calidad de vida. (2)

Los términos **debilidad asociada al cuidado intensivo** o ICU-AW por sus siglas en inglés y **síndrome post cuidado intensivo** o PICS, han surgido para agrupar el conjunto de alteraciones físicas, mentales y neurocognitivas que se han descrito ampliamente en pacientes internados en unidades de cuidado intensivo. Dentro de éstas, se resalta la pérdida y función de masa muscular secundario a miopatía y neuropatía por alteraciones en canales iónicos involucrados en la conducción nerviosa, así como degeneración axonal distal primaria de neuronas tanto motoras como sensitivas con consecuente atrofia por denervación. Hay también un componente muscular en relación con el desacople entre la excitación y la contracción y la capacidad mitocondrial funcional, que última instancia

conlleva a la pérdida de la capacidad de regeneración muscular (2,4) La debilidad muscular de estos pacientes afecta la capacidad funcional y conlleva, a una recuperación demorada, impide el retiro de la ventilación mecánica, aumenta los costos, disminuye la calidad de vida de los sobrevivientes (5)

Son múltiples los factores que pueden contribuir al desarrollo de PICS incluyendo procesos metabólicos y neuroendocrinos directamente relacionados con la condición crítica de estos pacientes. Sin embargo, se han documentado otros factores que pueden ser al menos parcialmente, inducidos por intervenciones médicas, procedimientos y medicamentos instaurados en la UCI, por lo que son considerados como potencialmente prevenibles o modificables. (2,6,7)

Los trastornos deglutorios son comunes en pacientes con PICS, su prevalencia es variable en diferentes estudios realizados dependiendo de la población elegida, el diseño del estudio y los criterios diagnósticos utilizados, reportándose en un rango amplio de 3-70%. (1,8–10) Se estima que más de uno por cada 6 pacientes admitidos en condición de emergencia y al menos 1 de cada 10 pacientes médico-quirúrgicos es afectado por disfagia postextubación. (11)

En algunos casos, la disfagia puede resolver o mejorar significativamente en cuestión de días, mientras que hasta en otros casos puede perpetuarse por largos periodos de tiempo. (12) Puntualmente en contexto postoperatorio, la incidencia de disfagia se ha reportado alrededor del 18.3%, de estos, hasta en el 80% persiste al egreso de la UCI y en más del 60% puede llegar a persistir hasta el egreso hospitalario. (10) Adicionalmente, la disfagia postoperatoria se ha establecido como predictor independiente de mortalidad a 28 y 90 días. (12) Por lo tanto, el uso temprano de estrategias de reconocimiento de la disfagia post extubación y la reevaluación periódica y regular en la UCI debe ser un estándar de cuidado. (13)

Los trastornos de la deglución están estrechamente asociados con eventos aspirativos post extubación, lo cual se ha relacionado persistentemente con secuelas y desenlaces deletéreos para los pacientes, incluyendo aumento en la morbimortalidad, mayor número de días de estancia hospitalaria y en la unidad de cuidado intensivo, requerimiento de re-intubación, neumonía, entre otros. (1)

La deglución segura y eficiente es un proceso complejo que requiere la coordinación de diferentes estructuras; la ejecución de nervios tanto craneales como espinales para convertir impulsos respiratorios en digestivos y viceversa. También involucra estructuras

del tracto aero digestivo superior, en el que cobra importancia el cierre del vestíbulo laríngeo, el cual debe ser realizado en un corto periodo de tiempo, dejando muy poco margen para errores. (1)

Los mecanismos de disfagia postoperatoria pueden ser multifactoriales, involucran la disminución de la capacidad respiratoria por disminución de la reserva respiratoria y bulbar, por desacondicionamiento y atrofia por desuso; La presencia de trastornos cognitivos asociados con el uso de sedación y de medicamentos con acción en sistema nervioso central, bien sea por compromiso directo del estado de consciencia o por intervención en sistemas de retroalimentación de la conducción nerviosa, y que en última instancia afectan la capacidad de ejecutar y coordinar movimientos precisos para alcanzar un proceso de deglución seguro y eficiente; El trauma mecánico que producen algunas medidas invasivas como el tubo orotraqueal o las sondas de alimentación, bien sea al momento de la instauración o del retiro, y que se incrementa con el tiempo prolongado de uso, la elección inadecuada del tamaño (sobredimensión), las maniobras traumáticas frecuentes en contextos agudos. (1,9) La sobre distensión del neumotaponador del tubo endotraqueal puede comprimir los cartílagos aritenoides o el nervio laríngeo recurrente y dislocar articulación cricotiroidea y/o cricoaritenoidea, afectando el cierre del vestíbulo laríngeo. (1,14) Otra causa de trauma mecánico que cobra particular importancia en los pacientes de cirugía cardiovascular es el uso frecuente del ecocardiograma transesofágico. (1)

La duración de la intubación influye en el periodo de desuso de la musculatura bulbar tanto para el habla como para la deglución, produciendo en algunos casos, atrofia difusa, debilidad, y alteración en la capacidad de producir la presión lingual y faríngea necesaria para la propulsión y el aclaramiento del bolo alimenticio, poniendo a los pacientes en riesgo de aspiración (1,8,10–12). Adicionalmente, los tubos endotraqueales impiden la función deglutoria normal y la elevación laríngea activa, reduciendo la apertura pasiva del esfínter esofágico superior, lo que impide el paso esofágico rápido, contribuyendo a la generación y perpetuación de disfagia. (10)

Adicional a las estructuras faríngeas, las funciones tanto somatosensoriales como motoras de la lengua, que resultan fundamentales para el gusto, la masticación, la deglución y el habla, se pueden ver comprometidas durante la estancia en la unidad de cuidado intensivo. (15) Esto puede contribuir a disartria, disfonía, disfagia, alteraciones en la masticación y traer consecuencias como aspiración, neumonía, deshidratación,

desnutrición, retracción social y disminución en la calidad de vida de estos pacientes. (10,12,15,16)

Son varios los momentos en que la función de la lengua se puede ver afectada con la ventilación mecánica. Desde el momento de inserción del tubo endotraqueal con un laringoscopio que comprime y desvía la lengua durante el procedimiento, se pueden producir lesiones del nervio lingual, con una consecuente pérdida de la sensación. Adicionalmente la inmovilización o la reducción de la frecuencia y del rango de movimiento de la lengua durante la intubación, puede disminuir su fuerza contráctil. (10,15,16) Se ha demostrado que el compromiso somatosensorial y motor de la lengua ocurre sin importar la edad, el sexo, el antecedente de tabaquismo, y las comorbilidades de los pacientes. Al comparar con un grupo de control, la disminución en la fuerza fue más persistente que la alteración sensorial, la cual ha demostrado mejoría al menos parcial en los 14 días post extubación. (15)

La disfagia post extubación se asocia con el aplazamiento del reinicio de la vía oral, potencialmente aumentado riesgos de deshidratación y desnutrición en pacientes que se encuentran frecuentemente en proceso hipercatabólicos, lo que cobra mayor importancia en la población añosa, frecuentemente frágil y con una reserva funcional limitada. (1,9) Otros factores asociados a los trastornos de deglución en pacientes en UCI son el reflujo gastroesofágico, la disincronía entre los procesos de respiración y la deglución. (10)

La falla en la extubación, definida usualmente como el requerimiento de re-intubación en las 24-72 h siguientes a una extubación programada, conlleva a un peor pronóstico, aumento de mortalidad y de estancia hospitalaria y puede ocurrir en 2-25% de pacientes. Dentro de las causas se encuentran la disfunción de la deglución, la tos inefectiva, la obstrucción de la vía aérea superior y el exceso de secreciones en la misma. (17) En un estudio relacionado en pacientes de una UCI médico quirúrgica, intubados más de 6 días, se encontró que el reflejo nauseoso es determinante para la falla en la extubación relacionada con aspiración o con secreciones excesivas en la vía aérea. (17) Tanto la fragilidad como la debilidad generalizada pueden se han visto asociadas con el compromiso de la capacidad para generar tos efectiva para expulsar material traqueal. (1)

La preocupación creciente por minimizar el impacto de las intervenciones médicas en el proceso de recuperación global de los pacientes ha llevado a un cambio cultural en las unidades de cuidado intensivo, donde previamente era más frecuente encontrar la mayoría de los pacientes profundamente sedados y con ventilación mecánica invasiva, a veces por

periodos prolongados de tiempo, y, hoy en día, fomentando un ambiente cada vez más propicio para la rehabilitación. (18)

Terapia física y movilización temprana.

Mientras que, en décadas previas, menos del 50% de pacientes con lesión pulmonar aguda que requerían intubación orotraqueal recibían una valoración de la deglución durante su hospitalización, (19) en la actualidad se ha incorporado cada vez más los grupos de rehabilitación multidisciplinaria. (7,20–22) En un estudio realizado en el transcurso de 5 años en Estados Unidos, que incluyo 264.137 pacientes con necesidad de ventilación mecánica, se encontró que los pacientes reciben terapia física, ocupacional y del lenguaje de manera variable. A pesar de la concientización sobre la movilización de estos pacientes, la rehabilitación no empieza tan temprano cómo es posible. Sólo en el 24% de los pacientes se instauró la terapia física el mismo día de la ventilación mecánica. (23) Hay menos información disponible en la literatura, pero en este estudio, sólo el 12.2% de pacientes bajo ventilación mecánica recibió terapia ocupacional mientras que el 33% recibió orden de terapia de lenguaje. (23)

La terapia de rehabilitación varía significativamente entre regiones geográficas, pero en general, puede involucrar además del equipo médico, profesionales de terapia física, terapia respiratoria, terapia del lenguaje o fonoaudiología, terapia ocupacional y enfermería. En países sin disponibilidad directa de terapias especializadas bien sea por cuestiones de formación o por disponibilidad de recursos, el grupo de enfermería o de terapia física asume el rol de terapia ocupacional, respiratoria y del lenguaje (20–24)

La rehabilitación física es crucial en pacientes que ingresan a la unidad de cuidado intensivo ya que 20-50% de pacientes críticamente enfermos experimentan algún grado de debilidad. En la actualidad, la movilización realizada en las unidades de cuidado intensivo es aceptada como una herramienta terapéutica con el potencial de prevenir o atenuar deterioro funcional en estos pacientes. Sin embargo, el momento ideal para el inicio de esta estrategia ha sido ampliamente debatido. (3,5)

La movilización temprana se ha propuesto como una política atractiva en este grupo de pacientes por parte de algunos autores. Pues en algunos estudios ha demostrado

adecuada tolerancia con menos incidencia de delirium, más días libres de ventilación mecánica y mejores desenlaces funcionales al alta hospitalaria. (2,25–27)

Sin embargo, su beneficio ha sido ampliamente debatido, debido a que algunos estudios no han demostrado mejoría de desenlaces, algunos incluso han reportado aumento en eventos adversos. Esto se ha atribuido a múltiples irregularidades al momento de comparar los estudios: principalmente, hay una falta de estandarización del concepto de *temprano*, que en muchos casos ni siquiera es definido. Tampoco hay consenso en la terapia establecida, el comparador o la “terapia usual”, que varía ampliamente dependiendo de las políticas institucionales, los criterios de inclusión y exclusión, ni la frecuencia de las intervenciones y los desenlaces. (3) Otro factor que puede contribuir a la falta de mejoría en los desenlaces es la heterogeneidad de los pacientes, tanto en su línea de base, como en su patología y en la respuesta a las intervenciones propuestas en la unidad. (3)

En un estudio reciente publicado en el New England Journal of Medicine (6) en el que se recogieron 750 pacientes en 49 hospitales de 6 países, y que fueron aleatorizados para movilización temprana o cuidado usual, no hubo diferencia en mortalidad a 180 días ni en estancia hospitalaria ni en desenlaces secundarios como días de ventilación mecánica y días fuera de la UCI. Hubo en cambio un aumento en efectos adversos y efectos adversos serios en el grupo de movilización temprana. Los autores reportan como limitaciones, un mayor nivel de movilización en el grupo de control respecto a estudios previos, barreras para la movilización que pudieron haber limitado el poder estadístico para detectar diferencias entre grupos, y sesgo de vigilancia en el grupo de movilización temprana. (6)

En un metaanálisis y revisión sistemática de la literatura publicada en 2019 que contó con 23 ensayos clínicos controlados aleatorizados, se concluyó que independientemente de las diferentes técnicas y periodos de movilización utilizados, la movilización temprana de los pacientes críticamente enfermos aumento el número de personas capaces de ponerse de pie, y el número de días libres de ventilación mecánica durante la hospitalización, una menor incidencia de debilidad asociada al cuidado intensivo, aumento en la distancia caminada al egreso hospitalario . No hubo en este estudio diferencias significativas en cuanto a mortalidad a 28 días o eventos adversos. (5)

Dentro de los tipos de intervención a considerar incluyen la movilización activa funcional, ergometría cíclica en cama, estimulación muscular eléctrica (con o sin ejercicios activos o pasivos), tablas de inclinación, entre otros. (4) Si bien los ejercicios pasivos no se encuentran formalmente dentro del proceso de rehabilitación, pues no se ha demostrado que aumenten la fuerza o la resistencia muscular. (3) Frecuentemente preceden el inicio de

las maniobras activas y son los más utilizados en pacientes bajo efectos de sedación profunda cuya condición clínica lo permite. (7,24,26,27)

La terapia debe ser en la medida de lo posible individualizada para la condición del paciente. Sin embargo, Hickman et al encontraron sólo 5 contraindicaciones para establecer la movilización temprana, que en su estudio fue definido como iniciada en las primeras 24 horas de estancia en UCI: Infarto agudo de miocardio, sangrado activo, aumento de la presión intracraneana y fractura de pelvis inestable. (25) En este grupo de pacientes, los parámetros hemodinámicos no fueron casi afectados por la terapia, causando su descontinuación en sólo 0.8% de las actividades, principalmente por hipotensión o arritmias. (25)

Una forma para determinar el riesgo/beneficio de aplicar la movilización temprana a pacientes en la unidad de cuidado intensivo fue desarrollado por un grupo multidisciplinario y es ampliamente utilizado en la actualidad. Consiste en la semaforización según el riesgo, de manera que se le otorga categoría verde a pacientes que presentan un bajo riesgo de eventos adversos, es decir, el beneficio sobrepasa las consecuencias potenciales de seguridad. Amarillo representa un riesgo de evento adverso, y en este grupo de pacientes debe haber una discusión de las precauciones a tener en cuenta y las contraindicaciones para la movilización temprana. La categoría roja implica un mayor riesgo de evento adverso, y en estos pacientes no se realiza movilización temprana a menos que sea autorizado por el equipo médico responsable. Si bien esta categoría no constituye una contraindicación, es una advertencia de que, en este caso, los riesgos podrían superar los beneficios.(20)

Diferentes guías clínicas apoyan el uso de un *bundle* para reducir o acortar el tiempo de rehabilitación y la incidencia de *delirium*. El ABCDEF incluye la evaluación, prevención y manejo de dolor; ensayos de despertar y de retiro de la ventilación mecánica invasiva, la evaluación, prevención y manejo del *delirium*, la movilización temprana y el ejercicio, y el involucrar y favorecer el ambiente familiar de los pacientes). (21)

Aún en equipos donde se promueve la rehabilitación temprana, los estudios han encontrado múltiples barreras tanto para movilizar a los pacientes en ventilación mecánica, como para mantener la dosis o el tiempo propuesto de intervención. (28)

En la práctica, ofrecer movilización temprana puede ser difícil, ya que requiere tiempo adicional, profesionales y materiales especializados, y un abordaje en equipo coordinado. (26)

Dentro de las barreras para la movilización temprana se han encontrado: Las relacionadas con el paciente, que pueden ser signos y síntomas o condiciones como inestabilidad hemodinámica o respiratoria; las barreras estructurales, como recurso humano y de equipo o técnicos; las barreras relacionadas con la cultura de la UCI, incluyendo hábitos y actitudes arraigadas en cada institución y limitaciones relacionadas con el proceso, falta de coordinación, ausencia de roles y reglas que determinen y distribuyan las tareas y responsabilidades de manera adecuada. (27)

Una de las barreras o de las razones más frecuentes para evitar que se cumplan los protocolos de movilización son los altos niveles de sedación. Los niveles adecuados de reducción y adecuación de la sedo analgesia para permitir la participación del paciente en ejercicios es importante para los desenlaces favorables. (27) Otros factores importantes son el adecuado manejo del dolor y el reconocimiento y manejo temprano del delirium. (27)

El objetivo de recuperación debe establecerse tan temprano como sea posible para Cada paciente. Y con él, se deben implementar estrategias para mejorar la implementación de la rehabilitación integral, dentro de las que es importante destacar la identificación de barreras y factores facilitadores; la creación de equipos multidisciplinarios con líderes que permita mejorar la comunicación, la educación y entrenamiento. (3)

El criterio de la seguridad del paciente es una barrera frecuentemente reportada. Con la movilización hay un aumento en el riesgo del retiro de medidas invasivas como sondas, tubos, catéteres y equipos y es una creencia común entre médicos y enfermeras que limita el rol de la fisioterapia y compromete la implementación de protocolos de movilización temprana. Este riego sin embargo se ha demostrado despreciable cuando el programa se realiza por personal capacitado y calificado para ello. (27)

Para mejorar la preocupación cultural en la UCI, un estudia aleatorizado multicéntrico internacional, implementó el establecimiento de los objetivos de movilización durante la revista médica diaria, con ello lograron alcanzarlos en 89% de los días en el grupo de intervención. En este estudio, se logró estancia en uci más corta y hospitalaria, mejoría en la movilidad funcional al alta hospitalaria. (7)

Terapia de lenguaje.

El rol de la terapia del lenguaje o fonoaudiología cobra cada vez mayor importancia en el cuidado intensivo, pues Similar a la movilización temprana, la terapia de lenguaje debería involucrarse en la valoración temprana de pacientes en UCI, pues el reconocimiento de la

disfunción laríngea y los trastornos deglutorios pueden minimizar el riesgo de disfagia orofaríngea y aspiración. (18)

Los terapeutas del lenguaje pueden proveer apoyo para los pacientes críticamente enfermos con condiciones agudas ya sean neurológicas o de otra etiología médica: trauma, hemorragias, tumores, lesiones de columna, condiciones respiratorias, así como pacientes en postoperatorios complejos de intervenciones neurológicas, cardiaca y de cirugía general. Estas pueden afectar habilidades del lenguaje motor, la deglución, la tos y la voz. (18)

El apoyo temprano de la terapia del lenguaje ayuda a facilitar la comunicación exitosa de los pacientes en cuidado intensivo con el equipo médico y con sus familiares, desarrollando programas de intervención especializadas para cada condición y trabajando con los pacientes desde el punto de vista funcional y de su patología. Esto a su vez mejor su bienestar psicosocial, su compromiso con el cuidado del día a día y la toma de decisiones consentidas. Además, las alteraciones en la comunicación se han asociado a delirium, un problema emergente en unidades de cuidado intensivo. (18,23)

El estudio videoflurosocópico de la deglución (VFS) y la evaluación endoscópica de la deglución (FEES) son necesarios para el diagnóstico certero de un trastorno deglutorio, pues la aspiración es una complicación devastadora que puede ocurrir de manera silente hasta en un 30-44% de pacientes y no puede ser diagnosticada con evaluaciones a la cabecera del paciente. Dentro de los factores asociados a procesos aspirativos se encuentra la neumonía, sondas de alimentación y traqueostomía, estancias en UCI prolongadas y aumento en la mortalidad hospitalaria. (29)

La FEES a diferencia de VFS es una herramienta portátil, con el beneficio adicional de que provee una vista directa de la dinámica faríngea y laríngea, así como de la anatomía y el manejo de secreciones por parte del paciente, por lo que puede apoyar decisiones clínicas y frecuentemente destaca dificultades que pueden después ser resueltas. (18) El uso de FEES permite hacer recomendaciones farmacológicas para el manejo de secreciones excesivas, así como intervenciones terapéuticas para fortalecer la base de la lengua, la constricción faríngea y el rango de movimiento laríngeo por lo que en el momento y en conjunto con VFS son considerados el *Gold-Standard* para la detección de trastornos deglutorios. (18,29) (18)

Sin embargo, el costo, en ocasiones la negativa de los pacientes, las dificultades para el traslado, y el requerimiento de personal y equipos especializados limitan la aplicación de estas herramientas en todos los pacientes posterior a la extubación. (18,19)

La habilidad de diagnosticar aspiración de manera fácil y acertada podría minimizar las complicaciones de la aspiración, limitando el retardo innecesario del inicio de la vía oral en pacientes de UCI. Por ello se han diseñado múltiples protocolos especializados y herramientas para identificar pacientes con trastorno deglutorio establecido o riesgo de desarrollarlo, con el objetivo de adoptar un abordaje preventivo que permita reducir complicaciones y desenlaces deletéreos. (18,19)

Dentro de estas, una combinación entre la prueba de deglución de agua y una evaluación de la deglución a la cabecera del paciente es la única estrategia que ha sido validada para la identificación de disfagia post extubación en pacientes sobrevivientes de falla respiratoria aguda. (13,29)El test de deglución de Gugging es una herramienta fácil de aplicar por diferentes terapeutas o enfermeras, y que permite una evaluación graduada de la capacidad de deglución del paciente, permitiendo recomendaciones nutricionales. (13)

En un estudio reciente el GUSS-ICU se comparó con FEES, demostrando 89-92% de sensibilidad y 67-89% especificidad para detectar pacientes con disfagia, comparable a los resultados obtenidos en pacientes con accidente cerebrovascular. La variabilidad confiabilidad inter observador fue buena. Esta herramienta tiene como ventaja su simplicidad y el uso de varias consistencias de alimentos, lo que le permite proveer recomendaciones dietarías para los pacientes en contexto post extubación.

Además de las maniobras de fortalecimiento de la musculatura orofaríngea con ejercicios, recientemente se ha propuesto la estimulación eléctrica a este nivel. En algunos estudios ha demostrado mejorar la reorganización de la corteza motora relacionada con la deglución, facilitar la activación de vías cortico bulbares y aumentar los niveles salivares de neurotransmisores asociados a la deglución, como la sustancia P. (8) En el estudio PHAST-TRAC se valoró la estimulación eléctrica faríngea en pacientes con accidente cerebrovascular traqueostomizados con disfagia neurogénica, encontrando que el uso de esta estrategia conllevó a un mayor número de pacientes listos para decanulación, así como menor prevalencia de neumonía, sin aumento en las complicaciones. (30)

En la última década se ha destacado el valor y la experticia de los terapeutas que apoyan el cuidado de pacientes en la unidad de cuidado intensivo. (22)Cada profesión relacionada con la terapia ofrece una amplia variedad de experiencia, conocimiento habilidades y experticia que contribuyen de diferentes formas a la rehabilitación temprana, óptima e integral de los pacientes en cuidado intensivo. Los objetivos de rehabilitación deben estar individualizados para cada paciente y la terapia debe formularse según dichos objetivos. Debe hacerse

énfasis en un trabajo interdisciplinario colaborativo, entendiendo el rol y responsabilidades de cada profesional. (10,12,18,22,23,31)

5. Objetivos

Objetivo general:

- Evaluar la diferencia en días de ventilación mecánica invasiva entre la terapia multimodal temprana y la terapia multimodal tardía en la unidad de cuidado intensivo adulto de la Fundación Santa Fe de Bogotá

Objetivos específicos:

- Describir las características sociodemográficas y clínicas de los pacientes con ventilación mecánica invasiva en la Unidad de Cuidado intensivo neurológico de la Fundación Santa Fe de Bogotá.
- Evaluar la eficacia de la terapia multimodal temprana frente a la terapia multimodal tardía en términos de días libres de ventilación mecánica y otros desenlaces secundarios.
- Establecer los factores sociodemográficos y clínicos que estén asociados con el tipo de terapia (temprana o tardía) y con el desenlace primario (días libres de ventilación mecánica) y los desenlaces secundarios.
- Evaluar la seguridad de la terapia multimodal temprana frente a la terapia multimodal tardía en términos de eventos adversos, eventos adversos serios, episodios de broncoaspiración y neumonía asociada al cuidado de la salud.
- Evaluar el impacto de los diferentes subgrupos previamente definidos, como el tipo de falla respiratoria (hipoxémica o hipercápnica) y el tipo de paciente (quirúrgicos, respiratorios, quemados, cardiovasculares), en los resultados del desenlace principal.

Hipótesis:

- Hipótesis alternativa principal: La terapia multimodal temprana reduce de forma estadísticamente significativa el número de días de ventilación mecánica invasiva comparada con la terapia multimodal tardía en pacientes intubados en la unidad de cuidado intensivo

- Hipótesis nula: No hay diferencias estadísticamente significativas entre el número de días de ventilación mecánica invasiva al comparar la terapia multimodal temprana frente a la terapia multimodal tardía en pacientes intubados en la unidad de cuidado intensivo

6. Metodología

6.1. Diseño del estudio

Se llevará a cabo un ensayo clínico controlado fase III, aleatorizado, en un único centro, con dos grupos paralelos de pacientes. Los pacientes que cumplan con los criterios de inclusión serán asignados, mediante aleatorización estratificada por sexo y grupo de edad, a uno de los dos grupos de estudio a una razón de 1:1. El ocultamiento de la asignación se realizará mediante sobres opacos sellados y secuencialmente numerados. En el primer grupo, llamado "brazo temprano" (EA), se incluirán pacientes que hayan sido intubados en la unidad de cuidados intensivos o en urgencias y/o salas de cirugía, y recibirán la terapia multimodal de manera temprana (<24 horas). En el segundo grupo, denominado "brazo tardío", también se aplicará la terapia multimodal, pero esta se iniciará a las 72 horas de la ventilación mecánica. Dado las características metodológicas del estudio, no será posible cegar a los participantes ni a los investigadores respecto al grupo al que pertenecen. Sin embargo, los profesionales de la salud que realizarán la última evaluación al día 90 de seguimiento no serán informados sobre el grupo de intervención en el que se encontraba el participante. Así mismo, los estadísticos y epidemiólogos que analizarán los datos estarán cegados sobre la intervención que se realizó en cada grupo. Por último, el estudio se analizará por intención de tratar.

6.2. Población

Criterios de inclusión: debe cumplir con todos los siguientes criterios:

- Pacientes adultos (mayores de 18 años)
- Pacientes hospitalizados en la unidad de cuidado intensivo adulto de la Fundación Santa Fe de Bogotá que requieran ventilación mecánica invasiva a través de un tubo endotraqueal durante un periodo mayor de 24 horas
- Índice de Barthel mayor o igual a 70.

Criterios de exclusión:

- Pacientes que requieran ventilación mecánica invasiva a través de una cánula de traqueostomía o tubo nasotraqueal.
- Pacientes a los que se realizó algún tipo de cirugía de cabeza y cuello.
- Paro cardiaco.
- Quemadura de la vía aérea.
- Quemaduras con una extensión mayor al 50% del área corporal.
- Paciente con enfermedad pulmonar obstructiva crónica.
- Pacientes remitidos de otra institución.
- Enfermedades desmielinizantes o de la placa neuromuscular.
- Pacientes requieran relajación neuromuscular.
- Pacientes con expectativa de vida menor a 180 días
- Pacientes que por criterio médico no se beneficiarían de tratamiento multimodal
- Pacientes que no ingresan a UCI por primera vez
- Pacientes que participan en otros ensayos clínicos de rehabilitación
- Paciente con trasplante hepático o renal.
- Paciente que se le practico cirugía de citoreducción (Sugar-Baker).

Criterio de evaluación y retiro del paciente:

1. **Cambios en el Estado de Salud del Participante:** Si un participante desarrolla condiciones de salud que podrían afectar la validez de los resultados, como infartos agudos de miocardio, tromboembolismo pulmonar, hemorragia intracerebrales, disfunción cardiaca (necesidad soporte inotrópico) y múltiples lavados quirúrgicos.

2. **Falta de cumplimiento:** Si un participante no cumple con los requisitos del estudio o no sigue las pautas del protocolo, podría ser retirado para mantener la integridad de los datos.
3. **Decisiones del participante:** Un participante tiene el derecho de retirarse del estudio en cualquier momento por cualquier razón, sin penalización.
4. **Resultados intermedios:** Si se descubren resultados significativos durante el estudio que hacen que sea éticamente necesario detener el estudio para proteger a los participantes.
5. **Criterios de finalización del estudio:** Una vez que se han recopilado suficientes datos para responder a las preguntas de investigación, los participantes restantes pueden ser retirados.

6.3. Tamaño de la muestra

Tomando en cuenta el estudio publicado por el estudio del Schweickert y colaboradores donde encontraron que la media de días de ventilación mecánica en paciente con terapia física usual fue de 6.1 $\pm$ 1.4 días y en terapia tempana fue de 3.4 $\pm$ 1.25 días (32) , para un error alfa de 0.05 y un poder estadístico de 0.8 se considera la siguiente formula:

A continuación, calcularemos el tamaño del efecto (ES, Effect Size) utilizando la diferencia de medias entre los grupos y las desviaciones estándar:

$$ES = \frac{|\ \mu 1 - \mu 2\ |}{\sqrt{DE1^2 - DE2^2/_2}}$$

$$ES = \frac{|\ 6.1 - 3.4\ |}{\sqrt{1.40^2 - 1.25^2/_2}}$$

$$ES = \frac{2.7}{\sqrt{3.52/_2}}$$

$$ES = 2.03$$

Con el tamaño del efecto calculado (ES ≈ 0.4463), podemos utilizar la fórmula para el tamaño de muestra que mencioné anteriormente:

$$n = \frac{2\,(\frac{\mathrm{Z\alpha}}{2} + Z\beta)^2\,.\,\,(\mathrm{\sigma 1}^2 + \,\mathrm{\sigma 2}^2)}{\mathrm{ES}^2}$$

Donde $\frac{Z\alpha}{2}$ es el valor crítico correspondiente al nivel de significancia alfa/2. Para α = 0.025, $\frac{Z\alpha}{2} \approx$ 2.81

Y $Z\beta$ es el valor crítico correspondiente al poder estadístico beta. Para β = 0.90, $Z\beta \approx 1.28$

Los otros valores que tenemos son los mismos:

- Media del grupo 1 (μ1) = 6.1 días
- Desviación estándar del grupo 1 (σ1) = 1.4 días
- Media del grupo 2 (μ2) = 3.4 días
- Desviación estándar del grupo 2 (σ2) = 1.25 días
- Tamaño del efecto (ES) ≈ 20.03(calculado previamente)

Sustituyendo estos valores en la fórmula:

$$n = \frac{2\,(2.81 + 1.28)^2\,.\,\,((1.40)^2 + \,(1.25)^2)}{(2.03)^2}$$

$$n = \frac{2\,(4.09)^2\,.\,\,(1.96 + 1.56)}{4.13}$$

$$n = \frac{2\,(16.72)\,.\,\,(3.52)}{4.13}$$

$$n = \frac{(35.35).\,(3.52)}{4.14}$$

$$n = 30.29$$

Redondeando:

$$n = 31 \text{ por cada grupo}$$

A los 31 pacientes por brazo estimados, se añade un 20% extra (6 pacientes por brazo) con el fin de minimizar la pérdida de poder estadístico en caso de que los pacientes se retiren del estudio o haya datos faltantes.

El tamaño de muestra también se ha determinado en función de la magnitud del efecto esperado de la movilización dentro de las 48-72 horas de la ventilación mecánica, como se sugiere en el estudio de referencia (33), lo cual justifica nuestra elección de evaluar la movilización temprana a las 24 horas versus a las 72 horas después de la ventilación mecánica en nuestro estudio.

6.4. Muestreo y aleatorización

Los pacientes que cumplan con los criterios de inclusión serán asignados, mediante aleatorización estratificada por sexo y grupo de edad (menores de 65 años y mayores de 65 años), a uno de los dos grupos de estudio a una razón de 1:1 (muestreo estratificado aleatorizado). El número de estratos resultantes corresponde a 4. La aleatorización se aplicará a cada estrato. Este tipo de muestreo y aleatorización reduce posibles desbalances y aumenta el poder estadístico, teniendo en cuenta que el número de participantes por brazo es de 37. El ocultamiento de la asignación se realizará mediante sobres opacos sellados y secuencialmente numerados, los cuales se localizarán en la oficina de la Unidad de Cuidado Intensivo en el cuarto piso del edificio de la expansión. Se llevará un registro de la persona que abrió el sobre de acuerdo con las guías de Buenas Prácticas Clínicas.

6.5. Intervención

La terapia multimodal temprana se define como el conjunto de maniobras terapéuticas que realiza el grupo de fisioterapia, fonoaudiología, terapia respiratoria y terapia ocupacional hasta las 24 horas de la intubación orotraqueal. Mientras la terapia multimodal tardía se caracteriza por el mismo tipo maniobras realizadas por el mismo grupo disciplinario a las 72 horas de la intubación orotraqueal.

La terapia multimodal se llevará a cabo según los resultados de la escala de sedación o conciencia RASS. Todo el personal involucrado en nuestro estudio recibirá capacitación específica en la evaluación precisa de esta escala, con el objetivo de reducir la variabilidad intra e interevaluador en la aplicación de la intervención.

Se aclara que el conjunto de maniobras terapéuticas llevadas a cabo en ambos grupos es parte integral del cuidado estándar proporcionado a nuestros pacientes en la unidad de cuidados intensivos. Además, ambos grupos recibirán todas las intervenciones médicas que normalmente se aplicarían en su atención durante la estancia en la UCI, independientemente de su participación en el estudio. Esto asegura la consistencia en la atención durante el estudio.

El manejo estándar en nuestra UCI implica la atención especializada y continuada de pacientes con enfermedades graves o que requieren monitoreo constante. Estos incluyen:

1. **Vigilancia Continua:** Monitoreo constante de signos vitales como la frecuencia cardíaca, la presión arterial, la frecuencia respiratoria y la saturación de oxígeno para detectar cambios inmediatos en la condición del paciente.
2. **Soporte Vital:** Proporcionar soporte vital avanzado, que puede incluir la administración de oxígeno, ventilación mecánica, medicamentos para mantener la presión arterial y otros tratamientos necesarios para mantener la estabilidad fisiológica.
3. **Control del Dolor y Sedación:** Manejo adecuado del dolor y la sedación para garantizar el confort del paciente y facilitar los procedimientos médicos necesarios.
4. **Prevención y Tratamiento de Infecciones:** Implementación de medidas de control de infecciones para prevenir la propagación de enfermedades, así como el uso de antibióticos y otros tratamientos específicos para combatir infecciones.
5. **Nutrición Especializada:** Proporcionar nutrición enteral o parenteral según las necesidades individuales de los pacientes, especialmente si no pueden alimentarse por vía oral.
6. **Manejo de Fluidos:** Controlar cuidadosamente los niveles de líquidos y electrolitos para mantener el equilibrio hídrico y corregir desequilibrios si es necesario.

7. **Coordinación Multidisciplinaria:** Fomentar la colaboración entre profesionales de la salud, incluyendo médicos, enfermeros, terapeutas, y otros especialistas, para garantizar una atención integral y personalizada.
8. **Comunicación con Familiares:** Mantener una comunicación clara y regular con los familiares del paciente, proporcionando información sobre el estado del paciente y brindando apoyo emocional.
9. **Manejo de Crisis:** Estar preparado para abordar situaciones de emergencia y tomar decisiones rápidas y efectivas en respuesta a cambios agudos en la condición del paciente.

Las intervenciones se realizarán cada día una vez el paciente allá sido incluido en el estudio y se realizarán de acuerdo con el conceso de expertos que recomienda realizar maniobras de movilización cuando el paciente tenga una FIO2 menor o igual de 0.6, una saturación percutánea de oxígeno mayor o igual al 90%, la frecuencia respiratoria sea menor de 30 veces por minuto y tenga una PEEP menor o igual al 10 cmH2O.

Estas maniobras se realizarán hasta el egreso (definido como el momento en el cual el intensivista determina que el paciente puede ser trasladado a hospitalización o se da el alta hospitalaria) del paciente de la UCI.

Intervenciones grupo de fonoaudiología

Fonoaudiología llevará a cabo una evaluación del nivel de sedación según la escala RASS. En el caso de pacientes que no respondan o tengan un puntaje de RASS igual o menor a -2, se llevará a cabo una ***terapia fonoaudiológica pasiva/asistida de tipo propioceptiva.*** Esto implica procedimientos como la alineación craneofacial, la movilización mandibular, la movilización lingual, la movilización cervical, la estimulación de reflejos faríngeos, la activación muscular orofacial, la hidratación de la mucosa oral y la activación muscular supra e infrahioidea.

Por otro lado, en pacientes con un puntaje RASS entre -1 y +1, se aplicará una ***terapia fonoaudiológica activa/asistida de tipo praxis neuromuscular.*** Esto involucra la realización activa de procedimientos como la alineación craneofacial, la movilización mandibular, la movilización lingual, la movilización cervical, la estimulación de reflejos faríngeos, la activación muscular orofacial, la hidratación de

la mucosa oral y la activación muscular activa supra e infrahioidea. Los detalles de cada intervención se encuentran en el anexo 1.

Intervenciones grupos de terapia física

Desde el campo de la fisioterapia, se llevará a cabo una evaluación utilizando la escala RASS. En el caso de pacientes que no respondan o tengan un puntaje de RASS igual o menor a -2, se aplicará ***una intervención pasiva/asistida de tipo propioceptiva***. Esto implica técnicas como la facilitación del reclutamiento de fibras musculares, posicionamiento inhibitorio de reflejos, movilización mioarticular, cargas y aproximaciones articulares, así como la activación de receptores articulares.

En pacientes con un puntaje RASS entre -1 y +1, se realizará ***una intervención activación motora*** utilizando técnicas de facilitación neuromuscular propioceptiva. Además, se aplicará electroestimulación en grupos musculares grandes y músculos accesorios respiratorios y diafragmáticos. Esto se hace con el objetivo de promover la intencionalidad en el movimiento, planificar y ejecutar patrones motores dinámicos que ayuden a desencadenar reacciones de soporte y equilibrio, con el propósito de promover el máximo desarrollo de funcionalidad en pacientes críticamente enfermos. La descripción detallada de la intervención está en el anexo 1.

En los pacientes que ya toleren la intervención con activación motora (RASS entre -1 y +1) se realizaran ejercicios de movilidad en cama, ejercicios de transferencia de cama a silla o de silla a cama, ejercicios de preparación a la marcha y finalmente marcha. La progresión dependerá de la tolerancia y la estabilidad.

Intervenciones grupos de terapia respiratoria

La intervención realizada por el grupo de terapia respiratoria se divide en tres grandes categorías:

- Técnicas de permeabilización de la vía aérea.
- Técnicas de reexpansión pulmonar.
- Técnicas de fortalecimiento muscular respiratorio.

La descripción detallada de la intervención por parte de la terapia respiratoria esta descrita de forma amplia en el anexo 1.

Intervenciones grupos de terapia ocupacional.

Para llevar a cabo este proceso, si el paciente presenta un nivel de conciencia RASS igual o inferior a -3, se realizarán actividades de estimulación multisensorial táctil, propioceptiva, vestibular, gustativa, auditiva y olfativa, asistidas completamente por el terapeuta. Estas actividades deben estar relacionadas con tareas de la vida diaria simuladas e incluir patrones de movimiento de los miembros superiores, con énfasis en patrones primarios como mano-cabeza, mano-boca, mano-homolateral, mano-contralateral y mano-perine, además de cuidar las articulaciones y los pies.

Para los pacientes con un nivel RASS entre -2 y -1, las intervenciones se realizarán de manera pasivo-asistida y se centrarán en estimulación sensorial, junto con retroalimentación cognitiva para reconocer elementos y realizar tareas cotidianas. Se emplearán instrucciones con un máximo de dos comandos, utilizando patrones de movimiento integrales como alcance, agarre, soltar y lanzamientos, con énfasis en planos frontales y laterales en la cama.

Cuando el paciente tiene un nivel RASS de 0 a +1, la intervención se enfoca en la participación activa y autónoma del paciente en actividades, con apoyo mínimo del terapeuta según la tolerancia del paciente. Se hace hincapié en la autonomía en las tareas cotidianas, integrando comandos sensoriales y cognitivos de baja, mediana y alta complejidad, utilizando patrones de movimiento de los miembros superiores trabajados previamente en todos los planos anatómicos, ya sea en la cama o en el borde de la cama, así como en una silla, según el progreso en la rehabilitación.

Para pacientes con un nivel RASS igual o superior a +2, las sesiones terapéuticas se centran en la modulación conductual y ambiental, incluyendo el entorno físico y familiar. Se promueve la conexión con el medio a través de actividades de estimulación multisensorial inhibitoria y estimulación cognitiva orientada a tareas significativas, con asistencia activa en la cama. Además, se educa a la familia sobre la importancia de mantener una orientación continua y se enriquecen los estímulos sensoriales para converger en una tarea común, utilizando estímulos visuales, auditivos, táctiles y propioceptivos (Ver anexo 1).

6.6. Desenlaces

Eficacia

- Desenlace principal: tiempo (días) desde la intubación hasta la extubación.
- Desenlaces secundarios:
    - Días libres de ventilación mecánica hasta el día 28
    - Días de delirium hasta el día 28
    - Tiempo en UCI con delirium hasta el día 28
    - Días de hospitalización con delirium hasta el día 28
    - Días de sedación hasta el día 28
    - Días libres de sedación hasta el día 28
    - Falla de extubación
    - Necesidad de ventilación mecánica no invasiva
- Desenlaces exploratorios:
    - Índice de Barthel al egreso
    - Días de estancia hospitalaria
    - Tiempo de estancia en UCI
    - Mortalidad por cualquier causa hasta los 90 días
    - Tiempo desde la intubación hasta el inicio de la terapia
    - Fuerza muscular medido con la escala Medical research council (MRC)
    - Disfagia a las 72 horas post extubación
    - Disfonía a las 72 horas post extubación
    - Tiempo hasta el inicio de vía oral post extubación
    - Desarrollo de neumonía asociada al cuidado de la salud,
    - Movilidad máxima medida por JH-HLM
    - Fuerza prensil
    - Función cognitiva medida mediante el Montreal Cognitive Assessment (MOCA)
    - Evaluación de funcionalidad en actividades cotidianas hasta el día 28
    - Test rápido de sensibilidad y destreza

Seguridad

- Desenlaces:
    - Número de eventos adversos.
        - Presión arterial alterada

        - Arritmia cardiaca
        - Desaturación de oxigeno
        - Dolor o agitación
        - Remoción de línea invasiva
        - Gastrointestinal (nauseas, vomito o diarrea)
        - Taquipnea
        - Estado neurológico alterado
    - Número de eventos adversos serios
    - Extubación no programada
    - Neumonía asociada al cuidado de la salud
    - Broncoaspiración

6.7. Mediciones

En el marco del protocolo, se llevarán a cabo mediciones en seis momentos clave de la investigación: al ingreso a la unidad de cuidado intensivo, durante la observación en la unidad de cuidado intensivo, al inicio del destete de soporte ventilatorio, posterior a la extubación, al egreso hospitalario y al final de la observación. Además, se realizarán mediciones continuas de desenlaces asociados a la seguridad de la intervención.

Al ingreso, se recopilarán detalles cruciales sobre cada paciente, como el nombre, código identificador, número de historia clínica, sexo y etnia. Asimismo, se evaluarán aspectos relacionados con la salud y la funcionalidad, incluyendo el Índice de Barthel y el Índice de Masa Corporal. La información diagnóstica se complementará con el código CI10, grupo de estudio al que pertenecen (temprana vs. tardía) y la unidad de internado (quirúrgica, neurológica, séptica y respiratoria, cardiovascular o quemados). Se incorporarán puntuaciones de gravedad como el SOFA, APACHE II y SAPS II, junto con datos temporales como la fecha de ingreso, fecha de egreso y el tiempo transcurrido desde la intubación hasta el inicio de la terapia. En cuanto a los parámetros respiratorios, se detallarán variables fundamentales al momento de la intubación, como volumen corriente, frecuencia respiratoria, PEEP, presión meseta, distensibilidad pulmonar estática, presión de conducción, resistencia de la vía aérea e índice PaO2/FIO2.

Durante la fase de observación, se implementará un riguroso proceso de evaluación diaria del paciente para garantizar un registro preciso de su evolución. Se monitorearán variables clave, como los días de ventilación mecánica (registrando la fecha de inicio y finalización), días libres de ventilación mecánica, días de delirium (indicando las fechas de

inicio y finalización), días de hospitalización con delirium, y días de sedación (con detalle de las fechas de inicio y finalización). También se documentará la duración de la ventilación mecánica, la posible necesidad de traqueostomía, la presencia del síndrome de dificultad respiratoria del adulto, y la eventual aparición de neumonía asociada al cuidado de la salud.

En la fase de inicio del destete, se centrará la atención en una serie de parámetros clave para evaluar la capacidad del paciente para prescindir de la ventilación mecánica. Se registrarán datos detallados, como el Delta POCC, que refleja la variación en la capacidad vital en un ciclo de tos, la presión muscular, la P0.1 que representa la presión en los primeros 0.1 segundos de la inspiración, la cantidad de secreciones por tubo orotraqueal, el índice de asincronías, la fuerza inspiratoria negativa (NIF), el porcentaje de fuga, el pico flujo de tos, y mediciones específicas relacionadas con la capacidad diafragmática, como la excursión y el grosor. Estos datos ofrecen una visión integral de la funcionalidad respiratoria durante el proceso de destete, permitiendo una toma de decisiones informada sobre la viabilidad de retirar la ventilación mecánica.

Antes del proceso de extubación, se recopilarán datos críticos para evaluar la preparación del paciente y minimizar riesgos potenciales. Se registrarán el Delta POCC, la presión muscular, la P0.1, la cantidad de secreciones por tubo orotraqueal, el índice de asincronías, la NIF, el porcentaje de fuga, el pico flujo de tos, excursión y el grosor diafragmático. Además, se documentará el OMAHA al momento de la extubación, indicando la presencia o ausencia de este evento.

La decisión de extubar a un paciente, según la Escala de OMAHA+, se fundamenta en una evaluación integral de diversos parámetros clínicos. Cada componente de esta escala ofrece información crucial sobre la capacidad del paciente para tolerar la retirada del soporte ventilatorio. Cuando todos los factores descritos en la Escala de OMAHA+ están presentes, se procede con la extubación.

La aplicación sistemática de la Escala de OMAHA+ está completamente estandarizada en nuestra unidad, formando parte esencial de nuestro protocolo de extubación. Esto garantiza una evaluación exhaustiva y uniforme de los pacientes antes de retirar el soporte ventilatorio. Además, hemos implementado un procedimiento de revisión sistemática del proceso, con el fin de fortalecer la coherencia, precisión y calidad de nuestra práctica clínica.

A continuación se detalla la Escala de OMAHA+:

1. Oxigenación (O): La relación PaO2/FiO2 > 150 , la saturación arterial de oxígeno, > 90% con FIO2 0.4, PEEP ≤ 8 cm H20 e índice de Tobin (fr/Vt) < 105. Cada variables será marcada como si o no.
2. Mecánica Respiratoria (M): volumen corriente exhalado mayor a 5 ml/kg capacidad vital > 10 ml/kg, la presión inspiratoria máxima (MIP) ≤ -20 a 25 cmH20, la frecuencia respiratoria < 35 por minuto y los valores de PIM y PEM.
3. Ácido-Base (A): paCO2 < 50 mmHg, pH > 7.32, ácido láctico > 2, y saturación venosa > 75%.
4. Hemodinámico (H): La presión sistólica > 90 o < 160, la frecuencia cardíaca ≤ 140 por minuto, la presencia de arritmias y dosis de vasopresores (alta >0.2 mcg(kg/min)
5. Vía Aérea (A): antecedente vía aérea difícil y los resultados del test de fuga > 40%.
6. Componente Clínico (+): Factores clínicos adicionales, como la presencia de tos efectiva, el manejo de secreciones, el estado neurológico del paciente, la resolución de la causa subyacente de la intubación y la manifestación de signos clínicos de dificultad respiratoria, también son considerados.

Después de la extubación, se monitorearán cuidadosamente varios indicadores para evaluar la recuperación y la estabilidad del paciente. Se registrará la presencia de disfonía a las 72 horas, la movilidad máxima medida por JH-HLM a las 24 horas y la fuerza prensil en el mismo período. Además, se evaluará la necesidad de ventilación mecánica no invasiva a las 48 horas, indicando si es requerida o no. La posible presencia de disfagia a las 72 horas también se documentará, junto con el momento de inicio de la vía oral, clasificado en dos opciones: 1) entre las 12 y 24 horas, y 2) después de las 24 horas. Se registrará la ocurrencia de falla de extubación a las 48 horas, indicando si ocurrió o no, y se medirá la fuerza muscular utilizando la Escala MRC a las 24 horas para evaluar la capacidad funcional del paciente tras el procedimiento de extubación.

Al concluir la fase de observación, se recopilarán datos esenciales relacionados con la funcionalidad y la estancia del paciente. Se registrará el Índice de Barthel al egreso hospitalario, la función cognitiva medida mediante el Montreal Cognitive Assessment (MOCA) y el test rápido de sensibilidad y destreza; proporcionando una medida detallada de la capacidad funcional y cognitiva alcanzada. Además, se documentarán los días de estancia hospitalaria y el tiempo de permanencia en la Unidad de Cuidados Intensivos (UCI), ofreciendo perspectivas sobre la duración del proceso de recuperación. Se evaluará la

mortalidad, indicando si ocurrió o no durante el periodo de observación. Asimismo, se medirá el estado de independencia funcional mediante las Actividades de la Vida Diaria (ADLs) al egreso hospitalario y al egreso de la UCI, brindando una evaluación integral del impacto de la atención y los procedimientos en la autonomía del paciente (ver Anexo 3 y 4).

Dentro del protocolo, la inclusión de variables de seguridad como la alteración de la presión arterial, arritmias cardíacas y desaturación de oxígeno destaca la importancia de la vigilancia continua de la salud cardiovascular y respiratoria de los pacientes. Estas variables sirven como marcadores clave para identificar posibles complicaciones durante la intervención o tratamiento médico. La detección temprana de alteraciones en la presión arterial brinda insights cruciales sobre la perfusión y la función circulatoria. La monitorización de arritmias cardíacas busca identificar irregularidades en el ritmo cardíaco, mientras que la observación de la desaturación de oxígeno alerta sobre posibles problemas en la oxigenación tisular. Estas medidas de seguridad, integradas en el protocolo, contribuyen a la evaluación exhaustiva y al manejo proactivo de cualquier cambio en la condición del paciente, respaldando así la seguridad y el bienestar durante el proceso clínico.

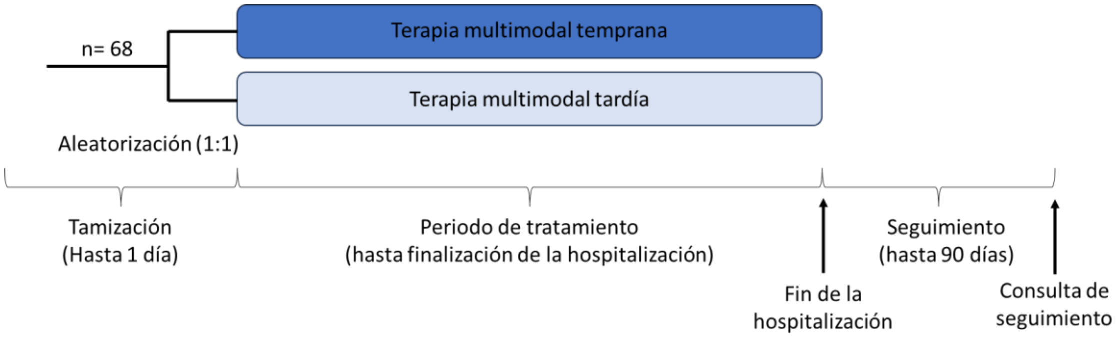

Figura 1. Diagrama del diseño del estudio

Interrupción de la terapia

La intervención se interrumpirá en cualquiera de los siguientes contextos clínicos:

- El paciente egresa de la UCI antes de cumplir 24 horas con soporte ventilatorio invasivo por tubo orotraqueal
- Muerte del paciente durante su estancia en UCI (hasta 28 días)
- El médico tratante determina que considerando el mejor interés del paciente es mejor interrumpir su participación en el estudio
- Retiro del consentimiento informado por parte del paciente o responsable legal

Posterior a la interrupción del tratamiento, el manejo médico del paciente seguirá acorde a los tratamientos y terapias estandarizadas de la unidad de cuidado intensivo. El tratamiento puede ser interrumpido temporalmente debido a razones médicas que incluyen, pero no se limitan a procedimientos quirúrgicos, estudios de radiología, estabilización hemodinámica del paciente. La terapia multimodal se reiniciará tan pronto como sea posible.

6.8 Pérdidas y Manejo de Datos Perdidos:

- Frecuencia y Distribución: Se registraron pérdidas durante el seguimiento de los participantes en ambos grupos del estudio. De [número total de participantes] inicialmente asignados, [número de participantes perdidos] se perdieron durante el periodo de estudio.
- Razones para las Pérdidas:
    - Retiro voluntario del consentimiento por parte del participante.
    - Eventos adversos no relacionados con el tratamiento en estudio.
    - Incumplimiento con el protocolo del estudio.
    - Transferencia a otras instalaciones médicas.
- Comparación entre Grupos: Se llevará a cabo un análisis comparativo entre el grupo control y de intervención, con el objetivo de valorar las distribución equitativa de pérdidas.
- Características de los Participantes Perdidos: Se compararán las características demográficas y clínicas iniciales de los participantes que completaron el estudio con aquellos que se perdieron durante el seguimiento, con el objetivo de valorar si las perdidas introdujeron sesgos sustanciales.
- Impacto en el Análisis: Se realizará un análisis de sensibilidad para evaluar el impacto potencial de las pérdidas en los resultados del estudio. Se presentarán resultados tanto con la inclusión como con la exclusión de los participantes perdidos.
- Manejo Estadístico: En el análisis estadístico principal, se utilizará el enfoque de intención de tratar, incluyendo a todos los participantes según su asignación original, independientemente de su cumplimiento o pérdida de seguimiento. Realizaremos además un análisis de sensibilidad basado en un análisis por protocolo, ya que podría brindarnos una medida del efecto del destete bajo condiciones ideales de adherencia al tratamiento.

- Reemplazo de Participantes Perdidos:
    - Consideración Ética: Antes de considerar el reemplazo de participantes perdidos, se realizará una evaluación ética de la justificación para tal acción. Se considerará si el reemplazo es éticamente aceptable y si su implementación respeta los derechos y bienestar de los participantes.
    - Métodos de Imputación: En caso de considerar el reemplazo de participantes perdidos, se utilizarán métodos de imputación estadísticamente sólidos y bien fundamentados. Se explorarán opciones como la imputación por la media, LOCF u otros métodos más avanzados como la imputación múltiple.
    - Transparencia y Documentación: Se proporcionará una descripción detallada de los métodos de imputación utilizados, incluyendo las razones para la elección de un método específico. Esto se incluirá en los informes finales y en la comunicación con el Comité de Ética.
    - Informe al Comité de Ética: En el informe al Comité de Ética, se describirán detalladamente las pérdidas de participantes y cualquier decisión relacionada con el reemplazo de datos faltantes. Se subrayará la transparencia en el manejo de pérdidas y la consistencia con el protocolo original del estudio.
    - Consideración de Expertos: Se buscará la opinión de expertos en estadísticas y ética en investigación para asegurar que cualquier decisión relacionada con el manejo de pérdidas y el reemplazo de participantes perdidos sea fundamentada y ética.

6.9. Criterios de adherencia al protocolo:

La adherencia al protocolo será evaluada en varios aspectos cruciales y se categorizará como alta o baja. Los pacientes con baja adherencia al protocolo serán excluidos del análisis de sensibilidad por protocolo intención, pero serán incluidos en el análisis por intención de tratar.

- Retiro del paciente: Se considerará baja adherencia cuando un paciente solicite voluntariamente su retiro del consentimiento, experimente un evento adverso no relacionado con el tratamiento en estudio o sea transferido a otras instalaciones médicas.
- Incumplimiento con el protocolo del estudio: Se clasificará como baja adherencia cuando a un paciente no se le pueda realizar el 80% de las intervenciones requeridas según el protocolo.

6.10. Procedimientos de seguimiento y seguridad:

- **Falta de Diferencia Significativa en la Duración de la Ventilación Mecánica**: Si, al finalizar el estudio y tras el análisis de los datos, no se observa una diferencia estadísticamente significativa en la duración media de la ventilación mecánica entre el grupo de terapia temprana y el grupo de terapia tardía, el ensayo podría considerarse un fracaso en términos de demostrar la eficacia de la terapia temprana para reducir los días de ventilación mecánica.
- **Altas Tasas de Desviación del Protocolo**: Si más del 20% de los participantes en uno o ambos grupos no cumple adecuadamente con el protocolo del estudio, esto podría afectar la validez de los resultados y llevar al fracaso del ensayo. ¿
- **Problemas de Seguridad o Toxicidad Inaceptable**: Si se observa una tasa inesperadamente alta de eventos adversos graves en el grupo de terapia temprana versus el tardío, el ensayo podría considerarse un fracaso debido a preocupaciones de seguridad.
- **Baja Tasa de Reclutamiento o Retención de Participantes**.
- **Interferencia Externa o Cambios en la Práctica Clínica**: Si ocurren cambios significativos en la práctica clínica estándar durante el transcurso del estudio que afectan la interpretación de los resultados, el ensayo podría considerarse un fracaso. Por ejemplo, si se introduce un nuevo tratamiento o procedimiento que impacta en la duración de la ventilación mecánica independientemente de la terapia asignada en el ensayo.

6.11. Recolección de la información

Descripción de variables:

**Variable**	**Definición**	**Tipo de variable**	**Escala/Unidad de medida o categorías**
Edad	Años cumplidos	Cuantitativa<br>Discreta	Años
Peso	Peso	Cuantitativa<br>Continua	Peso en kilogramos
Sexo	Sexo	Cualitativa Nominal	Femenino o masculino
Etnia	Etnia con la que se identifica el paciente	Cualitativa Nominal	Afrocolombiano<br>ROM<br>Indígena<br>Raizal<br>Ninguna
Diálisis	Necesidad de terapia de reemplazo renal	Categórica binominal	0: No<br>1: Si
Soporte con noradrenalina	Necesidad de soporte con noradrenalina	Categórica binominal	0: No<br>1: Si

Dosis máxima de noradrenalina	Dosis en mcg/kg/min	Cuantitativa continúa	Mcg/kg/min
Soporte inotrópico	Necesidad de soporte inotrópico	Categórica binominal	0: No<br>1: Si
Soporte con vasopresina	Necesidad de soporte con noradrenalina	Categórica binominal	0: No<br>1: Si
Dosis de vasopresina	Dosis en UI/min	Cuantitativa continúa	UI/min
Índice de Barthel	El Índice de Barthel es una escala utilizada para medir el grado de independencia funcional de una persona en actividades de la vida diaria (AVD)<br>Dos puntos de valoración RASS -3 o -2, RASS 0 y al egreso de UCI	Cuantitativa Discreta	Sera de 0 si el paciente fallece.
Índice de masa corporal	Es la relación entre el peso y la altura del paciente	Cuantitativa<br>Continua	Kilogramos/Metro cuadrado
Grupo	Grupo al que fue asignado el paciente	Cualitativa Nominal	1: temprana

			2: tardía
Diagnostico	Diagnostico principal	Cualitativa Nominal	Código diagnostico CI10.
Adherencia al protocolo	Grado de adherencia al protocolo	Categórica binomial	1: baja<br>2: alta
Tipo de falla respiratoria	Desarrollo de falla respiratoria hipoxémica (PaO2<60 mmHg) o hipercápnica (PCO2>45mmHg)	Categórica binomial	1: hipoxémica<br>2: hipercápnica.
Tipo de paciente	Patología principal que llevo a la necesidad de ventilación mecánica invasiva.	Cualitativa Nominal	1 = quirúrgica<br>2= neurológica.<br>3= séptica/respiratoria.<br>4=cardiovascular.<br>5=quemados<br>6= respiratoria.
Sequential Organ Failure Assessment Score<br>(SOFA)	Sistema de evaluación de la aparición y evolución del Fallo Multiorgánico en enfermos de UCI	Cuantitativa Discreta	Número

Acute Physiology and Chronic Health disease Classification System II (APACHE II)	Clasificación fisiológica de enfermedades agudas y crónicas	Cuantitativa Discreta	Número
Simplified Acute Physiology Score II (SAPS II)	sistema de evaluación de la gravedad de la enfermedad	Cuantitativa Discreta	Número
Volume corriente	Cantidad de volumen administrado en cada respiración	Cuantitativa continua	Número
Frecuencia respiratoria	Frecuencia respiratoria	Cuantitativa Discreta	Número
Presión al final de la expiración (PEEP)	Presión al final de la expiración (PEEP)	Cuantitativa continua	Número
Presión meseta	Equivalente a la presión alveolar en ausencia de flujo	Cuantitativa Continúa.	Centímetros de H2O
Presión de conducción	Presión de conducción	Cuantitativa Continua	Centímetros de H2O

Distensibilidad estática	Cambio de volumen dividido el cambio de presión	Cuantitativa Continua	ml/cmH20
Resistencia de vía aérea	la resistencia del tracto respiratorio al flujo de aire durante la inspiración y la espiración	Cuantitativa Continua	Numero
Índice PaO2/FIO2 post-intubación	Relación oxigenación pulmonar y FIO2	Cuantitativa Continua	Número
Presión neumotaponador inicio VMI, inicio asistencia ventilatoria y weaning	Ajuste de balón neumotaponador a perfusión traqueal	Cuantitativa Continua	Centímetros de H2O
Delta POCC inicio esfuerzo inspiratorio y weaning	Esfuerzo inspiratorio no voluntario	Cuantitativa Continua	Centímetros de H2O
Presión muscular en weaning (Pmus)	Magnitud de fuerza voluntaria	Cuantitativa Continua	Centímetros de H2O
P0.1	Impulso respiratorio neural	Cuantitativa Continua	Centímetros de H2O

Movilización de secreciones	Movilización de secreciones en el tubo orotraqueal	Cualitativa Nominal	**SI:** Valoración por terapeuta respiratorio de la presencia de abundantes secreciones traqueobronquiales **NO**: Valoración subjetiva por terapeuta respiratorio de la ausencia de abundantes secreciones traqueobronquiales
Índice de asincronías	Numero de asincronías presenciadas en un tiempo determinado	Cuantitativo Discreto	Porcentaje
NIF en weaning medido por terapia respiratoria	Esfuerzo inspiratorio negativo máximo	Cuantitativo Continua	Centímetros de H2O
NIF en weaning medido por fisioterapia	Esfuerzo inspiratorio negativo máximo	Cuantitativo Continua	Centímetros de H2O
% FUGA en weaning	% de perdida de volumen corriente al desinsuflar el neumotaponador	Cuantitativa Discreta	Porcentaje

Pico flujo de tos	Flujo espiratorio máximo en un intento de esfuerzo tusígeno	Cuantitativa Continua	medición flujo espiratorio máximo en curva flujo -tiempo L/min
Medición excursión diafragmática	Es el conjunto de la fuerza diafragmática combinada con presiones intratorácicas e intraabdominales adecuadas	Cuantitativa Continua	Centímetros
Medición % grosor diafragmático	Espesor de diafragma	Cuantitativa Continua	TF= (TEI-TEE)/TEE<br>Milímetros.
Días necesarios muerte	Días necesarios muerte	Cuantitativa Discreta	Número
Días para el inicio de soporte ventilatorio	Días que fueron necesarios desde el ingreso a la unidad de cuidado intensivo y el uso de ventilación mecánica invasiva	Cuantitativa Discreta	Días
Días desde el inicio de soporte ventilatorio al inicio del destete	Días desde el inicio de soporte ventilatorio (intubación) al inicio del destete	Cuantitativa Discreta	Días
Días de ventilación mecánica	Días de ventilación mecánica invasiva	Cuantitativa Discreta	Días

Días libres de ventilación mecánica	28 días menos los días de ventilación	Cuantitativa Discreta	Días<br>Se definirá como 0 si el paciente muere o tiene más de 28 días ventilado.
Delirium	Para que se considere que un paciente tiene delirium según el CAM-ICU, deben estar presentes tanto el primer criterio (alteración aguda del estado mental) como al menos uno de los otros tres criterios (incapacidad para mantener la atención sostenida, desorganización del pensamiento o cambios en el nivel de conciencia fluctuantes).	Cualitativa Nominal	1. Alteración aguda del estado mental (+/-).<br>2. Incapacidad para mantener la atención sostenida (+/-).<br>3. Desorganización del pensamiento (+/-).<br>4. Cambios en el nivel de conciencia fluctuantes (+/-)
Días de delirium	Días con presencia de delirium	Cuantitativa Discreta	Días
Días de sedación	Días con sedación	Cuantitativa Discreta	Días
Días libres de sedación	28 días menos días de sedación	Cuantitativa Discreta	Días

			Se definirá como 0 si el paciente muere.
Índice de Barthel al egreso hospitalario	El Índice de Barthel es una escala utilizada para medir el grado de independencia funcional de una persona en actividades de la vida diaria (AVD)	Cuantitativa Discreta	Sera de 0 si el paciente fallece.
Días de estancia hospitalaria	Días de estancia hospitalaria	Cuantitativa Discreta	Días
Días de estancia UCI	Días de estancia UCI	Cuantitativa Discreta	Días
Mortalidad	Muerte	Cualitativa Nominal	Si o No
Tiempo desde la intubación hasta el inicio de la terapia	Tiempo desde la intubación hasta el inicio de la terapia	Cuantitativa Discreta	Días
Fuerza muscular con la escala Medical research council	Evaluación de la fuerza muscular en paciente critico	Cualitativa Ordinal	5, normal: movimiento completo contra resistencia total<br>4+, buena (+): movimiento completo contra gravedad y fuerte resistencia

			4, buena (–): movimiento completo contra gravedad y resistencia mínima<br><br>3+, regular (+): movimiento completo solo contra gravedad<br><br>3, regular (-): movimiento parcial solo contra gravedad<br><br>2, escasa: movimiento eliminado la gravedad<br><br>1 mínima: contracción muscular visible sin movimiento<br><br>0 ausente: parálisis total.
Disfagia a las 72 horas de extubación	Disfagia a las 72 horas de extubación	Cualitativa Nominal	Si o No
Tiempo de inicio de vía oral.	Tiempo en que se inicia la vía oral después de la extubación.	Cualitativa Nominal	1: 12-24 horas<br><br>2: > 24 horas
Neumonía asociada al cuidado de la salud	El paciente desarrollo neumonía asociada al cuidado de la salud	Cualitativa nominal	0 = No<br><br>1 = Si

	durante la estancia en UCI		
Escala de movilidad máxima a las 24 horas.	Movilidad máxima medida por la escala de Johns Hopkins (JH-HLM)	Cualitativa Ordinal	8: camina más de 250 pies.<br>7: Caminas más de 25 pies<br>6: camina más de 10 paso.<br>5: está de pie más de un minuto.<br>4: se puede mover a la silla.<br>3: puede estar sentado al borde de la cama.<br>2: puede voltearse en la cama.<br>1: Solo puede estar acostado.<br><br>Sera de 0 si el paciente fallece.
Destreza Manual y velocidad de procesamiento psicomotor	El evaluado realiza 20 giros de 180° a la moneda colombiana de $200 pesos modelo nuevo; (semejante a la de 25	Cualitativo Nominal	0: Integra<br>1: Alterada

	centavos EE.UU.) lo más rápido posible, usando primer, segundo y tercer dedo únicamente, se espera que la persona realice mínimo 15 segundos en mano dominante y 19 segundos en no dominante<br>Test de rotación de la moneda		
Valoración de la integridad Sensorial	Se realiza tamizaje de la sensibilidad (superficial y profunda) a través de valoración de la sensación cutánea o táctil, propiocepción, y estereognosia (forma, tamaño y textura) en términos identificación discriminación y localización del estímulo.<br><br>Tamizaje de sensibilidad sugerido por literatura	Cualitativo Nominal	0: Integra<br>1: Alterada
Valoración cualitativa del	Mediante evaluación estandarizada, en	Cualitativo Nominal	0: Normal

procesamiento cognitivo	actividad dirigida que evalúa dominios de visoespacial/ejecutiva, denominación, atención, lenguaje, memoria, abstracción y orientación.<br><br>Se empleará la versión que corresponda al paciente asi:<br>Estándar: para personas con niveles de escolaridad bachiller y superiores.<br>• Básico: para personas con analfabetismo o baja escolaridad<br>Blind: para personas con alteración significativa de la función visual<br>Evaluación de procesamiento cognitivo (Montreal Cognitive Assessment)		1: Alterado
Valoración cuantitativa del	Mediante evaluación estandarizada, en actividad dirigida que	Cuantitativa Discreta	Numérico

procesamiento cognitivo	evalúa dominios de visoespacial/ejecutiva, denominación, atención, lenguaje, memoria, abstracción y orientación.<br><br>Se empleará la versión que corresponda al paciente asi:<br>Estándar: para personas con niveles de escolaridad bachiller y superiores.<br>• Básico: para personas con analfabetismo o baja escolaridad<br>Blind: para personas con alteración significativa de la función visual<br>Evaluación de procesamiento cognitivo (Montreal Cognitive Assessment)		
Evaluación de funcionalidad en actividades cotidianas	Se valorará el nivel de autonomía e independencia del paciente en	Cualitativo Ordinal	7: Independencia completa

	actividades cotidianas básicas de la vida diaria determinando el nivel de funcionalidad en estas, apoyado con la observación directa, historia clínica, referencia del personal de enfermería y familiar/cuidador.<br>Functional Independence Measure (FIM).		6: Independencia modificada<br>5: Supervisado<br>4: Asistencia mínima<br>3: Asistencia moderada<br>2: Asistencia máxima<br>1: Asistencia total
Fuerza prensil al momento de la extubación	Fuerza prensil de la mano dominante dentro de 24 horas posterior a la extubación	Cuantitativa Continua	Kilogramos<br>Sera de 0 si el paciente fallece.
Extubación fallida	Incapacidad de respirar espontáneamente en las primeras 48 horas después del retiro de la vía aérea artificial y el paciente es incapaz de mantener una vía aérea permeable	Cualitativa Nominal	Si o No
VMNI POS extubación	Soporte ventilatorio no invasivo pos-	Cualitativa Nominal	NO = 0<br>SI = 1

	extubación dentro de 48 horas		
Ventilación mecánica prolongada	Ventilación mecánica invasiva mayor a 14 días	Cualitativa Nominal	Si o No
Traqueostomía	Necesidad de uso de cánula de traqueostomía.	Cualitativa Nominal	Si o No
Síndrome de dificulta respiratoria del adulto	Infiltrados alveolares bilaterales con una PAFI menor de 300.	Cualitativa Nominal	Si o No
Disfonía a las 72 horas.	Presencia de disfonía a las 72 horas después de la extubación.	Cualitativa Nominal	0: No<br>1: Si
**OMAHA+**	El uso de la Escala de OMAHA+ durante el proceso de retiro del soporte ventilatorio para tomar la decisión de extubación	Cualitativa Nominal	NO = 0<br>SI = 1
Alteración de la presión arterial	El paciente experimenta cambios significativos de la tensión arterial durante la realización de las terapias	Cualitativa nominal	0 = No<br>1 = Hipotensión sin requerimiento de manejo médico

			2 = Hipotensión con requerimiento de manejo médico<br>3 = Hipertensión sin requerimiento de manejo médico<br>4 = Hipertensión con requerimiento de manejo médico
Arritmias cardiacas	El paciente presento una arritmia cardiaca durante la realización de la terapia	Cualitativa nominal	0 = No<br>1 = Arritmia cardiaca sin requerimiento de manejo médico<br>2 = Arritmia cardiaca con requerimiento de manejo médico
Desaturación de oxigeno	El paciente se desaturo durante la realización de la terapia	Cualitativa nominal	0 = No<br>1 = Desaturación de oxígeno sin requerimiento de manejo médico<br>2 = Desaturación de oxígeno con requerimiento de manejo médico
Dolor o agitación	El paciente presento dolor o agitación	Cualitativa nominal	0 = No

	durante la realización de la terapia		1 = Dolor o agitación sin requerimiento de manejo médico<br>2 = Dolor o agitación con requerimiento de manejo médico
Remoción de la línea invasiva	El paciente se retiró accidentalmente o de forma no programada una línea invasiva durante la realización de la terapia	Cualitativa nominal	0 = No<br>1 = Si
Taquipnea	El paciente presento taquipnea durante la realización de la terapia	Cualitativa nominal	0 = No<br>1 = Taquipnea sin requerimiento de manejo médico<br>2 = Taquipnea con requerimiento de manejo médico
Deterioro neurológico	El paciente presento deterioro neurológico durante la realización de la terapia	Cualitativa nominal	0 = No<br>1 = Deterioro neurológico sin requerimiento de manejo médico<br>2 = Deterioro neurológico con requerimiento de manejo médico

Extubación no programada	El paciente se extubo de forma no programada durante la estancia en UCI	Cualitativa nominal	0 = No<br>1 = Si
Broncoaspiración	El paciente broncoaspiro durante la estancia en UCI	Cualitativa nominal	0 = No<br>1 = Si

Procedimientos para la recolección de la información:

Los datos serán recopilados por el personal clínico, terapeutas y asistentes de investigación de la unidad de cuidado intensivo, todos familiarizados con el estudio y las variables definidas previamente. Las intervenciones y variables recopiladas por los terapeutas se detallan en el Plan Operativo del Ensayo Clínico (Anexo 1). La recopilación de datos tendrá lugar en el tercer y cuarto piso de la Unidad de Cuidado Intensivo de la FSFB. Se proporcionará capacitación a todos los investigadores sobre la recopilación de datos y los momentos específicos para completar el instrumento de recopilación de datos. Además, se evaluará el conocimiento del protocolo mediante un examen, que se considerará aprobado con una puntuación superior a 4, y se brindará retroalimentación para abordar posibles deficiencias. Las variables medidas por los terapeutas se registrarán diariamente desde el inicio de la intervención. Las mediciones se realizarán en cinco momentos clave de la investigación: al ingreso, durante la observación en la unidad de cuidado intensivo, al iniciar el destete del soporte ventilatorio, antes de la extubación, después de la extubación y al concluir la observación. Finalmente, se realizará un seguimiento a los 90 días posteriores a la intubación endotraqueal, durante el cual se contactará telefónicamente a los pacientes o a sus representantes legales para evaluar la mortalidad.

Para garantizar la calidad de los datos, se implementarán las siguientes estrategias:

- Validación de datos durante la entrada: Se realizará una verificación de los formatos, rangos y coherencia lógica de los datos al momento de ingresarlos.

- Doble entrada de datos: Dos investigadores ingresarán de manera independiente los mismos datos, y luego se compararán para identificar discrepancias. Cualquier discrepancia encontrada será investigada y corregida.
- Verificación de consistencia y coherencia: Se llevarán a cabo controles para garantizar que los datos no presenten información contradictoria o incoherente en los registros.
- Comprobación de valores extremos: Se identificarán y verificarán los valores extremos o atípicos en los datos, para asegurar su validez.
- Control de calidad en etapas posteriores: Se realizarán verificaciones cruzadas, se revisarán muestras aleatorias de los resultados y se utilizarán métodos de validación externa siempre que sea posible.

Estas estrategias se implementarán con el fin de asegurar la calidad y confiabilidad de los datos recolectados en el estudio.

Instrumento de recolección de datos:

Los investigadores clínicos serán los encargados de recopilarla información en RedCap (ver anexo 5). RedCap proporciona un entorno seguro para el almacenamiento y gestión de datos clínicos, garantizando la privacidad y confidencialidad de la información. Esta transferencia a RedCap permitirá una mejor organización y acceso a los datos, facilitando su análisis y posterior uso en el estudio.

6.12. Calidad de datos: error y sesgos

- Sesgo de asignación: Ocurre cuando hay una selección inapropiada o sesgada de los participantes para los grupos de tratamiento y control. Para minimizar este sesgo, se utiliza la asignación aleatoria para garantizar que los participantes sean asignados de manera equitativa a los diferentes grupos.
- Sesgo de cegamiento (ciego): Puede ocurrir cuando los participantes, los investigadores o los evaluadores conocen la asignación del tratamiento y esto puede

influir en su comportamiento o en la evaluación de los resultados. Los ECAs suelen utilizar la asignación al azar cegada, donde los participantes y/o los investigadores no conocen la asignación del tratamiento. Sin embargo, dado las características del protocolo es imposible cegar.

- Sesgo de exclusión: Puede ocurrir cuando se excluyen ciertos participantes del estudio de manera selectiva, lo que puede sesgar los resultados. Este sesgo se manejará con un uso adecuado de los criterios de inclusión y exclusión.
- Sesgo de pérdida de seguimiento: Ocurre cuando los participantes abandonan el estudio o se pierde el seguimiento de ellos de manera desigual entre los grupos de tratamiento y control. La pérdida desigual de participantes puede afectar la validez de los resultados. Para minimizar este sesgo, se realizarán esfuerzos para maximizar la retención de los participantes y analizar los resultados de acuerdo con el principio de “intención de tratar”.
- Sesgo de informe: Se produce cuando los resultados seleccionados o informados en el estudio están influenciados por la preferencia de los investigadores o por la intención de presentar los resultados de una manera favorable. Para evitar este sesgo, se registrará el protocolo del estudio en www.clinicaltrials.gov y se publicará el protocolo en una revista indexada.

6.13. Plan de análisis estadístico

Se realizará una prueba de Shapiro-Wilk para verificar la distribución de las variables cuantitativas, teniendo en cuenta el número de pacientes incluidos. Los datos se presentarán como medias o medianas, acompañadas de su distribución estándar o rango intercuartílico, según corresponda. Las variables categóricas se describirán como frecuencias absolutas y relativas.

Para comparar las variables continuas entre los dos grupos (terapia temprana vs. tardía), se empleará una prueba T o la prueba U de Mann-Whitney, dependiendo de la distribución de los datos. Para las variables categóricas, se utilizará una prueba de $\chi 2$ o la prueba exacta de Fisher, según corresponda.

El objetivo principal del estudio es evaluar la asociación entre el uso de terapia multimodal temprana y los días de ventilación mecánica invasiva. Para responder a esta pregunta, realizaremos un análisis de supervivencia y una regresión Cox. Se creará una variable de riesgo combinando el tiempo de ventilación mecánica invasiva y la extubación (si/no). Luego, se realizará un análisis de regresión Cox univariable con la variable de riesgo como la variable dependiente y el grupo de intervención (temprana vs tardía) como la variable independiente. Asimismo, se generará una curva de supervivencia y se analizarán las diferencias entre los dos grupos mediante una prueba de log-rank.

Para evaluar los factores relacionados con el tiempo de ventilación mecánica, se realizarán modelos de regresión Cox univariable para todas las variables incluidas en el estudio. Para determinar si las variables continuas se pueden evaluar de manera continua o como variables categóricas, se verificará el supuesto de linealidad a través del gráfico de residuos de Martingala. En caso de no cumplirse la linealidad, se estratificará la variable y se definirán puntos de corte según la apariencia del gráfico. Posteriormente, se incluirán aquellas variables con un valor p de significancia estadística (menor a 0.25) y aquellas variables clínicamente importantes (edad, sexo, delirium, etc.) en un modelo de regresión Cox multivariable. Se seleccionará el mejor modelo mediante un proceso de Backward Stepwise basado en los criterios de información de Akaike (AIC) y bayesiano (BIC).

Se evaluarán las variables que podrían actuar como factores de confusión mediante un segundo modelo de regresión múltiple. En este modelo, se retirará la variable potencialmente confusora y se calculará la pendiente o estimador resultante (estimación ajustada). Si existe un cambio mayor al 15%-20%, la variable confusora se incluirá en el modelo final. En caso contrario, se optará por el modelo más parsimonioso.

Para evaluar la interacción entre dos variables, se realizará otro modelo de regresión que incluirá una nueva variable que resulta de multiplicar las dos variables que potencialmente interactúan. Si el coeficiente de regresión de esta nueva variable tiene un nivel de significancia menor a 0.05, se concluye que existe interacción y dicha variable se incluirá en el modelo. Las variables con potencial de interacción se identificarán mediante la evaluación de los factores de confusión. Los investigadores también evaluarán las variables clínicas que puedan ser consideradas como variables de interacción o de confusión a través de la estrategia arriba descrita.

Se evaluará el cumplimiento del supuesto de proporcionalidad de riesgo mediante residuos de Schoenfeld y se analizarán los valores influyentes mediante gráficos de residuos versus observaciones. La multicolinealidad se evaluará a través de las curvas de matriz.

Usaremos modelos de regresión logística para valorar la relación entre nuestras variables dicotómicas, como extubación fallida, VMNI pos extubación, ventilación mecánica prolongada, traqueotomía y disfonía, y las otras variables estudiadas. Las asunciones de un modelo de regresión logística, como la linealidad en el registro de las probabilidades, independencia de errores, homogeneidad de varianza, ausencia de multicolinealidad, ausencia de valores atípicos y proporcionalidad de los efectos, serán evaluadas a través de análisis de residuos y diagnósticos gráficos, como el análisis de residuos de Pearson o el análisis de residuos de deviance.

Se empleará una estrategia de ajuste por multiplicidad en las pruebas estadísticas, como el método de Bonferroni, Holm-Bonferroni u otros métodos que controlen la tasa de falso descubrimiento (FDR), con el fin de mantener la tasa global de error tipo 1 en un nivel aceptable.

Realizaremos una exploración detallada a través de un análisis de sensibilidad de posibles interacciones entre los subgrupos identificados, según escala RASS, según la adherencia al protocolo y otras variables relevantes, con el objetivo de profundizar en la comprensión de cualquier influencia conjunta que pueda afectar los resultados del estudio. En caso de no identificar diferencias estadísticamente significativas mediante los análisis de regresión, se llevará a cabo un análisis pos-

hoc para evaluar si el tamaño de la muestra reclutada fue adecuado para detectar posibles diferencias entre los subgrupos establecidos. Este análisis adicional permitirá ajustar la interpretación de los resultados y proporcionará información valiosa sobre la validez de las conclusiones obtenidas.

Por último, se establecerá un comité independiente de monitoreo de datos para llevar a cabo revisiones intermedias del tamaño de muestra, permitiendo ajustes en función de las variaciones en las tasas de eventos observadas durante el estudio, garantizando así la potencia estadística adecuada a lo largo de su ejecución.

7. Consideraciones éticas

Este Ensayo Clínico Aleatorizado se realizará de acuerdo con los principios éticos internacionales, en consonancia con la Declaración de Helsinki de la Asociación Médica Mundial, en la 29 Asamblea General, Tokio, octubre de 1975 y reformas posteriores, así como las normas sobre Bioética en Investigación para el territorio colombiano (Resolución No. 8430 de 1993, emitida por el Ministerio de Salud y conocido como el "Código de Bioética en Investigación").

De acuerdo con la Resolución Colombiana de 8430 de 1993, este estudio se considera una investigación "con riesgo mayor que el mínimo" dado que este estudio emplea métodos aleatorios de asignación a esquemas terapéuticos. Dada esta categorización se tomará el consentimiento informado del paciente o en caso de no poder otorgar su consentimiento informado, el mismo se solicitará a su representante legal.

Por otro lado, los datos personales de los participantes del estudio estarán protegidos bajo las cláusulas de confidencialidad y tratamiento de datos personales de acuerdo con la resolución 1581 de 2012 de la ley colombiana. Todos los datos del estudio serán incorporados a una base de datos tras ser debidamente anonimizados. El objetivo de esta será garantizar la confidencialidad de los datos incluidos mediante registros codificados, así como permitir un volcado de los mismos al software de análisis estadístico para su posterior

estudio. El proyecto será valorado por el Comité Ético del Hospital Fundación Santa Fe de Bogotá. Los resultados se publicarán en revistas académicas y científicas, preservando su precisión y haciendo referencia a datos globales y no a individuos particulares.

Dado que es un ensayo clínico controlado requiere consentimiento informado (Anexo 2), los investigadores clínicos se encargarán del proceso de explicación y obtención del consentimiento informado posteriormente a la comprobación de los criterios de inclusión y exclusión de los potenciales participantes del estudio. En el caso de los pacientes que tengan compromiso del estado de conciencia o que no puedan ejercer su autonomía, se le explicará al representante legal del paciente el propósito, la intervención, la duración, los beneficios, los riesgos, las medidas de confidencialidad y la información de contacto del estudio. Se explicará también que la participación será voluntaria y sin ningún perjuicio en la calidad de la atención médica en caso de no participar o retirar su consentimiento en cualquier momento del estudio.

Principios de bioética

Beneficencia: El principio de beneficencia se aplica en este estudio de dos formas:

1. Se maximizarán los potenciales beneficios a los pacientes, dado que ambos brazos recibirán terapia multimodal, la cual tiene amplia evidencia a nivel mundial. Así mismo, el ensayo clínico aleatorizado es el tipo de estudio primario con mayor rigor científico que genera el mayor conocimiento aplicable al mejoramiento de los tratamientos y terapias utilizadas actualmente.
2. La monitorización y la supervisión del estudio asegura que los potenciales beneficios superen los riesgos. Se realizarán evaluaciones de seguridad de forma continua para que en caso de detectarse algún peligro para los participantes el estudio sea detenido.

No maleficencia: El estudio está diseñado para minimizar los potenciales peligros o daños a los participantes. Los participantes estarán monitorizados de forma continua, especialmente porque se tratan de pacientes hospitalizados en la Unidad de Cuidado Intensivo. El estudio priorizará la seguridad de los pacientes por encima de los resultados de la investigación por lo que cualquier riesgo innecesario será evitado.

Justicia: El principio de justicia se garantizará de dos formas:

1. Habrá una selección equitativa de los participantes, es decir, no se discriminará ningún grupo. Se seleccionarán a los participantes de forma consistente conforme a los criterios de inclusión y exclusión previamente descritos, los cuales tienen bases científicas y clínicas.
2. El estudio tendrá una distribución equitativa de beneficios y riesgos. Los potenciales beneficios y riesgos serán asignados de forma justa entre los participantes, lo que significa que el acceso a las terapias o conocimiento resultante no será injustamente limitado y que los costos y riesgos de la investigación no recaerán desproporcionadamente sobre una población especifica.

Autonomía: El principio de autonomía es asegurado en este estudio mediante el proceso del consentimiento informado voluntario por parte de los participantes. Los potenciales participantes serán informados completamente con respecto al propósito, los objetivos, la intervención, la duración, los beneficios, los riesgos, las alternativas, las medidas de confidencialidad y la información de contacto del estudio. Ellos, en su autonomía, tienen el derecho a aceptar o declinar su participación en el estudio sin ser coaccionados.

Publicación: nos comprometemos a la publicación completa y transparente de todos los resultados obtenidos, tanto positivos como negativos. Esta práctica se alinea con nuestra dedicación a la integridad científica y al avance del conocimiento en el campo médico. Cada hallazgo, sin importar su naturaleza, será documentado y compartido con la comunidad científica y el público en general, cumpliendo así con nuestro compromiso de contribuir al cuerpo global de conocimiento médico.

8. Alcances

Este estudio propuesto tiene el potencial de fortalecer significativamente la comunidad científica y médica en múltiples aspectos. La investigación planeada no solo busca generar nuevos conocimientos sobre terapia y estrategias de acortamiento de la ventilación mecánica, sino que también tiene como objetivo establecer metodologías innovadoras que puedan ser aplicadas en investigaciones futuras. Además, al enfocarse en rehabilitación, este protocolo tiene el potencial de mejorar las prácticas clínicas actuales y ofrecer nuevas

perspectivas. Es importante destacar que este estudio fomentará la colaboración interdisciplinaria al involucrar a investigadores y profesionales de diversas áreas, lo que promoverá un intercambio de ideas y enfoques que enriquecerá el campo en su conjunto.

Se espera que los resultados de esta investigación se presenten en congresos a nivel nacional e internacional, brindando la oportunidad de compartir los hallazgos con la comunidad científica y los profesionales de la salud. Esta difusión permitirá la discusión y retroalimentación de los resultados obtenidos.

Además, se buscará publicar los resultados en revistas de alto impacto, preferentemente clasificadas como Q1 o Q2. Estas revistas son reconocidas por su riguroso proceso de revisión por pares y su prestigio en el ámbito académico y científico. La publicación en este tipo de revistas proporcionará una mayor visibilidad y alcance a los resultados de la investigación, permitiendo que sean accesibles para otros investigadores y profesionales de la salud en todo el mundo.

El objetivo de compartir los resultados en congresos y publicaciones científicas es fomentar la difusión del conocimiento, contribuir al avance de la investigación en el campo y permitir que los hallazgos de este estudio sean utilizados para mejorar la atención y el tratamiento de los pacientes.

9. Trayectoria de los investigadores

Laura María Castillo es especialista en medicina crítica y cuidado intensivo de la Universidad del Rosario. Actualmente se desempeña como intensivista en el departamento de Medicina Crítica y Cuidado Intensivo de la Fundación Santa Fe de Bogotá como jefe de la sección de sepsis y ventilación de la UCI institucional con estudios relacionados al área.

Jorge Iván Alvarado Sánchez es médico anestesiólogo con maestría en fisiología cuya tesis (relación variabilidad de presión de pulso/variabilidad de volumen sistólico en un modelo de choque por endotoxina porcina) fue Laureada por la Universidad Nacional de Colombia. Cuenta con algunos estudios publicadas en revistas médicas de alto impacto como son Critical Care, Shock, Annals of Intensive Care, Scientific Report, Journal of Intensive Care Medicine, Revista Colombiana De Anestesiología Y Acta Colombiana De Cuidado Intensivo. También ha sido revisor par de algunas revistas de anestesiología y cuidado intensivo de alto impacto como son Critical Care, Scientific Reportr, Journal of Clinical Monitoring

Computing, Intensive Care Medicine Experimental, BMC Anesthesiology, BMJ open, Anaesthesiology Intensive Therapy, entre otros.

La trayectoria del doctor Alvarado puede ser revisada en los siguientes enlaces:

- https://orcid.org/0000-0003-4320-3150.
- https://scienti.minciencias.gov.co/cvlac/visualizador/generarCurriculoCv.do?cod_rh=0002095674#.

10. Cronograma de actividades

Actividades	Mes1	Mes2	Mes3	Mes4	Mes5	Mes6	Mes7
**Planteamiento del proyecto**	X						
**Sometimiento Subdirección estudios clínicos**		X					
**Aprobación comité de Ética**		X					
**Registro de pacientes**			X	X	X	X	
**Análisis estadístico y resultados**						X	
**Escritura artículo científico**						X	X

11. Presupuesto y financiación

Esta investigación no ha recibido, no recibe, ni recibirá financiación de parte de ninguna agencia, institución o patrocinador en los sectores público, comercial u organizaciones sin ánimo de lucro.

RUBROS	FUENTE DE FINANCIACIÓN	TOTAL
Honorarios Asesores	Fondos propios	$ 5000000
Licencias de software adicional	FSFB	$3000000
Administrativos	Fondos propios	$2000000
Publicación	Fondos propios	$1000000
TOTAL		$11000000

12. Referencias

1. Plowman EK, Anderson A, York JD, DiBiase L, Vasilopoulos T, Arnaoutakis G, et al. Dysphagia after cardiac surgery: Prevalence, risk factors, and associated outcomes. Journal of Thoracic and Cardiovascular Surgery. 2021 Feb 1;165(2):737-746.e3.
2. Rousseau AF, Prescott HC, Brett SJ, Weiss B, Azoulay E, Creteur J, et al. Long-term outcomes after critical illness: recent insights. Crit Care. 2021 Dec 1;25(1).
3. Sanger H. How early is early? When should rehabilitation begin in critical illness? ACPRC Journal. 2020 Jun;52.
4. Hodgson CL, Schaller SJ, Nydahl P, Timenetsky KT, Needham DM. Ten strategies to optimize early mobilization and rehabilitation in intensive care. Vol. 25, Critical Care. BioMed Central Ltd; 2021.
5. Zhang L, Hu W, Cai Z, Liu J, Wu J, Deng Y, et al. Early mobilization of critically ill patients in the intensive care unit: A systematic review and meta-analysis. PLoS One. 2019 Oct 1;14(10).

6. Hodgson C, Bailey M, Bellomo R, Brickell K, Broadley T, Buhr H, et al. Early Active Mobilization during Mechanical Ventilation in the ICU. New England Journal of Medicine [Internet]. 2022 Nov 10;387(19):1747–58. Available from: http://www.nejm.org/doi/10.1056/NEJMoa2209083

7. Schaller SJ, Anstey M, Blobner M, Edrich T, Grabitz SD, Gradwohl-Matis I, et al. Early, goal-directed mobilisation in the surgical intensive care unit: a randomised controlled trial. The Lancet. 2016 Oct 1;388(10052):1377–88.

8. Koestenberger M, Neuwersch S, Hoefner E, Breschan C, Weissmann H, Stettner H, et al. A Pilot Study of Pharyngeal Electrical Stimulation for Orally Intubated ICU Patients with Dysphagia. Neurocrit Care. 2020 Apr 1;32(2):532–8.

9. See KC, Peng SY, Phua J, Sum CL, Concepcion J. Nurse-performed screening for postextubation dysphagia: A retrospective cohort study in critically ill medical patients. Crit Care. 2016 Oct 12;20(1).

10. Zuercher P, Moret CS, Dziewas R, Schefold JC. Dysphagia in the intensive care unit: Epidemiology, mechanisms, and clinical management. Vol. 23, Critical Care. BioMed Central Ltd.; 2019.

11. Zuercher P, Dziewas R, Schefold JC. Dysphagia in the intensive care unit: a (multidisciplinary) call to action. Vol. 46, Intensive Care Medicine. Springer; 2020. p. 554–6.

12. Schefold JC, Berger D, Zürcher P, Lensch M, Perren A, Jakob SM, et al. Dysphagia in mechanically ventilated ICU patients (Dynamics): A prospective observational trial. Crit Care Med. 2017;45(12):2061–9.

13. Troll C, Trapl-Grundschober M, Teuschl Y, Cerrito A, Compte MG, Siegemund M. A bedside swallowing screen for the identification of post-extubation dysphagia on the intensive care unit – validation of the Gugging Swallowing Screen (GUSS)—ICU. BMC Anesthesiol. 2023 Dec 1;23(1).

14. Borders JC, Fink D, Levitt JE, McKeehan J, McNally E, Rubio A, et al. Relationship Between Laryngeal Sensation, Length of Intubation, and Aspiration in Patients with Acute Respiratory Failure. Dysphagia. 2019 Aug 15;34(4):521–8.

15. Su H, Hsiao TY, Ku SC, Wang TG, Lee JJ, Tzeng WC, et al. Tongue Weakness and Somatosensory Disturbance Following Oral Endotracheal Extubation. Dysphagia. 2015 Apr 28;30(2):188–95.

16. Brodsky MB, Pandian V, Needham DM. Post-extubation dysphagia: a problem needing multidisciplinary efforts. Intensive Care Med. 2020 Jan 1;46(1):93–6.

17. Houzé MH, Deye N, Mateo J, Mégarbane B, Bizouard F, Baud FJ, et al. Predictors of extubation failure related to aspiration and/or excessive upper airway secretions. Respir Care. 2020 Apr 1;65(4):475–81.

18. McRae J, Montgomery E, Garstang Z, Cleary E. The role of speech and language therapists in the intensive care unit. J Intensive Care Soc. 2020 Nov 1;21(4):344–8.

19. Omura K, Komine A, Yanagigawa M, Chiba N, Osada M. Frequency and outcome of post-extubation dysphagia using nurse-performed swallowing screening protocol. Nurs Crit Care. 2019 Mar 1;24(2):70–5.

20. Hodgson CL, Stiller K, Needham DM, Tipping CJ, Harrold M, Baldwin CE, et al. Expert consensus and recommendations on safety criteria for active mobilization of mechanically ventilated critically ill adults. Crit Care. 2014 Dec 4;18(6).

21. Deemer K, Myhre B, Oviatt S, Parsons M, Watson M, Zjadewicz K, et al. Occupational therapist-guided cognitive interventions in critically ill patients: a feasibility randomized controlled trial. Canadian Journal of Anesthesia [Internet]. 2023;70:139–50. Available from: https://doi.org/10.1007/s12630-

22. Twose P, Jones U, Bharal M, Bruce J, Firshman P, Highfield J, et al. Exploration of therapists' views of practice within critical care. BMJ Open Respir Res. 2021 Nov 8;8(1).

23. Prohaska CC, Sottile PD, Nordon-Craft A, Gallagher MD, Burnham EL, Clark BJ, et al. Patterns of utilization and effects of hospital-specific factors on physical, occupational, and speech therapy for critically ill patients with acute respiratory failure in the USA: Results of a 5-year sample. Crit Care. 2019 May 16;23(1).

24. Clarissa C, Salisbury L, Rodgers S, Kean S. Early mobilisation in mechanically ventilated patients: A systematic integrative review of definitions and activities. Vol. 7, Journal of Intensive Care. BioMed Central Ltd.; 2019.

25. Hickmann CE, Castanares-Zapatero D, Bialais E, Dugernier J, Tordeur A, Colmant L, et al. Teamwork enables high level of early mobilization in critically ill patients. Ann Intensive Care. 2016 Dec 1;6(1).

26. Van Willigen Z, Collings N, Richardson D, Cusack R. Quality improvement: The delivery of true early mobilisation in an intensive care unit. BMJ Qualitive Improvement Programme [Internet]. 2016; Available from: http://bmjopenquality.bmj.com/

27. Miranda Rocha AR, Martinez BP, Maldaner da Silva VZ, Forgiarini Junior LA. Early mobilization: Why, what for and how? Vol. 41, Medicina Intensiva. Ediciones Doyma, S.L.; 2017. p. 429–36.

28. Hodgson CL, Capell E, Tipping CJ. Early Mobilization of Patients in Intensive Care: Organization, Communication and Safety Factors that Influence Translation into Clinical Practice. Vol. 22, Critical Care. BioMed Central Ltd.; 2018.

29. Lynch YT, Clark BJ, Macht M, White SD, Taylor H, Wimbish T, et al. The accuracy of the bedside swallowing evaluation for detecting aspiration in survivors of acute respiratory failure. J Crit Care. 2017 Jun 1;39:143–8.

30. Dziewas R, Stellato R, van der Tweel I, Walther E, Werner CJ, Braun T, et al. Pharyngeal electrical stimulation for early decannulation in tracheotomised patients with neurogenic dysphagia after stroke (PHAST-TRAC): a prospective, single-blinded, randomised trial. Lancet Neurol. 2018 Oct 1;17(10):849–59.

31. Zuercher P, Schenk N V., Moret C, Berger D, Abegglen R, Schefold JC. Risk Factors for Dysphagia in ICU Patients After Invasive Mechanical Ventilation. Chest. 2020 Nov 1;158(5):1983–91.

32. Schweickert WD, Pohlman MC, Pohlman AS, Nigos C, Pawlik AJ, Esbrook CL, et al. Early physical and occupational therapy in mechanically ventilated, critically ill patients: a randomised controlled trial. The Lancet. 2009 May;373(9678):1874–82.

33. Ding N, Zhang Z, Zhang C, Yao L, Yang L, Jiang B, et al. What is the optimum time for initiation of early mobilization in mechanically ventilated patients? A network meta-analysis. PLoS One. 2019 Oct 7;14(10):e0223151.

ANEXO 1

Intervenciones correspondientes a cada terapia

Fundación Santa Fe de Bogotá	**Fundación Santa Fe de Bogotá**<br>**Nivel 1**: Apoyo Diagnóstico y Terapéutico<br>**Nivel 2**: Neumología<br>**Nivel 3**: Terapia Respiratoria<br>**Estandar Operacional**: Ventilación Mecánica	**Código**	AMB-TR-EO1-007
		**Fecha**	2021-01-28
		**Versión**	Vigente: 5.0

Estratégico	**Misional**	Apoyo

Objetivo

- Llevar un cierto volumen de gas a los pulmones, para que en los alvéolos se produzca el intercambio gaseoso
  - Reducir el trabajo respiratorio del paciente
  - Dar soporte a la función respiratoria hasta la reversión total o parcial de la causa

Alcance

Desde la calibración del equipo (VENTILADOR), instalacion y hasta la extubacion del paciente, logrando que respire por sus propios medios

CONDICIONES/RECURSOS NECESARIOS

1. CONDICIONES / RECURSOS NECESARIOS
• **Personal asistencial:**<br>• Medico Intensivista<br>• Terapeuta Respiratoria<br>• Unidades de Cuidado Intensivo<br>• Fuente de aire<br>• Fuente de oxigeno<br>• Fuente electrica<br>• Ventilador<br>• Circuito desechable

RESULTADOS ESPERADOS

2. RESULTADOS ESPERADOS
• Mantener niveles apropiados de PO_2 y PCO_2<br>• Descanso musculatura respiratoria<br>• Mantenimiento intercambio gaseoso

- Aumento oxigenacion arterial
- Reduccion trabajo respiratorio

RIESGO (S) / CÓMO NEUTRALIZARLO (S)

3. RIESGO (S)	COMO NEUTRALIZARLO (S)
1. Barotrauma.	1. Minimizar volumen minuto<br>Volumen corrientes bajos<br>Limitar presion maxima de insuflación<br>Reducir obstruccion bronquial
2. Neumonia asociada a la Ventilacion Mecanica	2. Extubacion temprana<br>Cabecera a 30 grados<br>Lavados bucales con clorexidina<br>Utilización de filtros bacterianos, sondas de succión cerrada<br>Lavado de manos
3. Toxicidad por oxigeno	3. Reducir Fraccion inspirada de oxigeno<br>Aplicacion de PEEP
4. Atrapamiento Aereo	4.Reducir obstruccion bronquial<br>Disminuir volumen minuto<br>Aumentar Tiempo espiratorio
5. Atelectasias	5. Aumentar volumen corriente<br>Aumentar PEEP

DESCRIPCION DE LA ACTIVIDADES

4. DESCRIPCIÓN DE LAS ACTIVIDADES

1. En la unidad de cuidado intensivo debe tener ventilador de reserva (con sticker verde), confirmando su calibración.
2. Calibre el ventilador con su respectivo circuito antes de instalarselo al paciente
3. Aliste el equipo con los parámetros básicos ventila torios y abra la hoja o formato de ventilación mecánica, anote datos del paciente y parámetros con que alista el ventilador según orden medica.
4. Coloque el sistema cerrado de succión y el filtro bacterial. Dispositivos que deben ser remplazados cada 24 a 48 horas según prescripción del proveedor.
5. Coloque el ventilador al paciente, verifique el acople del paciente al ventilador y la estabilidad del paciente. Reporte y confirme con el médico los parámetros y estabilidad del paciente.
6. Anote cualquier cambio en el formato de ventilacion mecanica y en la historia clinica. Mida la presion del neumotaponador del Tubo orotraqueal, Tubo nasotraqueal o canula de traqueostomia que se debe realizar en cada turno y registre en el formato de ventilacion mecanica.
7. Todos los días al comenzar cada turno (mañana, tarde y noche) , se debe limpiar la pantalla , el teclado del ventilador y la superficie con una toalla de cloro organico (pedir los implementos a la operaria del aseo de cada una de las unidades). Se

debe colocar guantes de examen limpios, empezar el proceso de limpieza de arriba hacia abajo y de izquierda a derecha, si es necesario cambie la toalla las veces que crea necesario. Al terminar deseche la toalla y guantes en caneca roja de solidos, realice lavado de manos, registre el procedimiento en el formato de control de ventiladores.

9. Siga la técnica adecuada para retirar la ventilación mecánica (estándar extubacion AMB-NEUM 01-014)

NOTA: Los ventiladores serán desinfectados, armados, revisados y calibrados en el servicio de Neumología (sotano dos).
La marcación se hará según estado del mismo:
Sticker VERDE= Listo para su uso
Sticker AMARILLO= Sin calibrar
Sticker ROJO= NO apto para usar, dañado, pendiente revisión tecnica
Todos los ventiladores deben estar cubiertos por bolsa plastica transparente

Lleve el control permanente de los parámetros y cambios realizados a los parametros del paciente, todo cambio o control deben ser registrados en el formato de control de ventilacion mecanica y en la historia clinica

Los circuitos de paciente son **DESECHABLES**, previo se instala en el ventilador, se calibra y se coloca al paciente . Luego se pide a la farmacia para reponerlo a la reserva.

El circuito se deja toda la estancia del paciente a no ser que visiblemente se vea sucio o con material organico; solicitelo a farmacia con cargo al paciente y desechar en caneca roja de solidos dentro de la habitacion del paciente, no olvide anotar este cambio en el formato de control de ventilacion mecanica
Preparaciones: Alistar insumos y ventilador, calibrar el ventilador
Recomendaciones: Paciente entubado o traqueostomizado
Controles: Se realiza control de parametros ventilatorios una vez por turno o si se realiza cambios en algun parametro se registra en la hoja de control del ventilador

RESPONSABLE, FRECUENCIA Y LUGAR

RESPONSABLE FRECUENCIA Y LUGAR
1 – 12 **Responsable**. Terapeutas Respiratorios<br>**Frecuencia**: Según Orden medica<br>**Lugar**: Urgencias, Unidades de Cuidado Intensivo

Fecha de Emisión

2008-02-01

COMPONENTE TERAPIA RESPIRATORIA

Coordinadoras:

- ❖ Ana Gabriela López Rubio, email: aglopez904@gmail.com
- ❖ Diana Carolina Ortiz Moreno, email: carolinao.21@hotmail.com

Justificación

La labor del terapeuta respiratorio ha crecido exponencialmente en la última década como eslabón del grupo multidisciplinario de la Unidad de Cuidado Intensivo siendo un elemento fundamental en cuanto a pensamiento crítico se trata, anticipándose a los sucesos en la práctica clínica siendo un consejero leal del médico intensivista a la hora de solucionar problemas que sean de carácter respiratorio y ventilatorio.

Después de los acontecimientos ocurridos en la pandemia, la labor del terapeuta respiratorio en el ejercicio de la terapia multimodal ha impactado de forma óptima en la práctica profesional cotidiana, desarrollando protocolos en muchos ámbitos como la oxigenoterapia convencional, manejo integral del paciente ventilado, retiro del ventilador mecánico en la extubación y el tratamiento de morbilidades respiratorias las cuales se encuentran en auge cada día debido a la contaminación del medio ambiente (biomasa), el uso de tabaco y enfermedades ocupacionales, permitiendo llevar una directriz del cuidado integral a estos pacientes, siendo parte de la promoción y prevención de enfermedades respiratorias con la educación a pacientes y familias.

Siendo parte de la cadena que forma la terapia multimodal, administrando de forma racional y controlada el oxígeno necesario en la realización de todos los procesos vitales del cuerpo humano, siendo profesionales con inteligencia sensorial, siendo un apoyo diagnóstico, monitoria y rehabilitación de la mecánica pulmonar y hemodinámica utilizando los sentidos, desde la visión para evaluar las curvas del ventilador mecánico y diagnosticar patologías obstructivas y restrictivas guiando el cuidado del pulmón minuto a minuto, la audición para

encontrar ruidos adventicios pulmonares orientando el tratamiento a seguir, hasta la aplicación de la palpación en la kinesiterapia del tórax, con el objetivo de eliminar secreciones alojadas en el pulmón y fortalecimiento de los músculos de la respiración.

Iniciando desde el minuto de instaurada la ventilación mecánica invasiva, pasando por el transcurso diario hasta el día del destete ventilatorio, el éxito de las variables determinantes de la retirada del soporte dependen de la monitoria estricta de la mecánica ventilatoria, la prevención de la neumonía asociada a ventilación mecánica y el proceso de weaning donde se recobra la respiración espontánea y se evalúa la posibilidad de la retirada del ventilador, es vital, vigilar las posibles causas que pueden llevar a fracaso del destete.

En la monitoria y vigilancia de la mecánica ventilatoria del parénquima pulmonar en paciente en fase aguda donde se encuentra sedado o incluso bajo relajación muscular y con esfuerzo inspiratorio completamente abolido la medición de la distensibilidad estática refleja el volumen pulmonar potencialmente ventilable, un estudio transversal multi-institucional realizado entre los años 2017-2019 evaluaron el fracaso del destete y requerimiento de intubación en las primeras 72 horas posterior a la extubación en función a distensibilidad estática < 50 ml/cm H2O provocando en los pacientes una capacidad inspiratoria disminuida. (1)

El PEF (Pico flujo de tos) de los pacientes con extubaciones fallidas fue significativamente menor que el de aquellos con extubaciones exitosas (64,2 L/min vs 81,9 L/min). Los pacientes con extubaciones fallidas permanecieron más tiempo en la UCI que aquellos con extubaciones exitosas (11,7 vs 5,3 días). Aquellos con un pico flujo de tos ≤ 60 L/min la probabilidad es 5 veces mayor de falla a la extubación y 19 veces más probabilidad de fallecer durante la estancia hospitalaria. (2)

La disfunción diafragmática y la debilidad adquirida en la UCI impactan en el éxito o fracaso en el destete de la ventilación mecánica según mediciones guiados por evaluación ultrasonográfica del diafragma: excursión y medición de grosor diafragmático; así como la fuerza muscular según escala MRC en una prueba de respiración espontánea (PVE) durante 30 minutos, es así como un estudio evaluó estas variables encontrando que la debilidad adquirida en la UCI se relacionaba de forma directa con destete difícil y prolongado, mayor estancia en UCI y prolongación de la ventilación mecánica. (3)

En el proceso del destete ventilatorio es un término que se utiliza para describir el proceso gradual de disminución del soporte ventilatorio, descenso de parámetros de forma progresiva hasta que el paciente pueda iniciar la respiración con requerimiento de mínimo soporte ventilatorio. Existe una clasificación del destete ventilatorio dividido según PVE: Destete simple, como la primera prueba de respiración espontanea exitosa. Destete difícil, PVE fallida en el primer o tercer intento o requerido PVE posterior a 7 días en ventilación mecánica y Destete prolongado, el cual el paciente presenta PVE exitosa en un tiempo mayor a 7 días bajo soporte ventilatorio.

Existen varios factores de riesgo de fracaso en la extubación:

- Fracaso de 2 o más pruebas de Ventilación espontanea
- Insuficiencia cardiaca crónica
- Tos débil
- Riesgo de estridor pos extubación

- Edad mayor a 65 años
- $PaCO_2$ > 45 mmHg posterior a la extubación. (4)

se ha demostrado recientemente que la prueba de respiración espontánea (PVE) en PSV durante 30 minutos resultó en una tasa de extubación significativamente exitosa en relación a prueba de Tubo en T durante 2 horas, dado que una prueba menos exigente vs. un tiempo mucho más largo puede sobreestimar el esfuerzo inspiratorio posterior a la extubación. (5)

La vigilancia hemodinámica también es crucial en relación al éxito o fracaso en la extubación y es la relación lineal entre la dosis de soporte vasopresor y el riesgo de falla a la extubación. Dosis de vasopresor > 0.1 mcg/kg/min se ha asociado a riesgo de intubación en relación al balance de líquidos y presiones de llenado, ya que al tener dosis bajas de soporte vasopresor el balance urinario va a tender a estar negativo para evitar la sobrecarga de volumen y subsecuente edema pulmonar. (6)

La prueba de fuga del manguito de neumotaponador en la anticipación del riesgo de estridor post extubación el cual se asocia a edema laríngeo hace parte de la gama de test necesarios, dado que si la prueba de fuga es <45% es imprescindible el inicio de corticoterapia endovenosa previa y posterior repetición de prueba para precaver si es necesario la administración de corticoides nebulizados. (7)

Constituyendo así, un rol insustituible en el grupo de terapia multimodal.

Alcance

Pacientes seleccionados para el proyecto de investigación: “Asociación entre terapia multimodal temprana y días de ventilación mecánica en la unidad de cuidado intensivos de la Fundación Santa Fe de Bogotá: un ensayo clínico controlado aleatorizado (SECEC-2023-129).

Población objeto

Criterios de inclusión

- Pacientes hospitalizados en la unidad de cuidado intensivo adulto de la Fundación Santa Fe de Bogotá que requieran ventilación mecánica invasiva a través de un tubo endotraqueal durante un periodo mayor de 72 horas y que se espere que continue por al menos 24 horas.
- Índice de Barthel mayor o igual a 70.

Criterios de Exclusión:

- Pacientes que requieran ventilación mecánica invasiva a través de una cánula de traqueostomía o tubo nasotraqueal.
- Pacientes a los que se realizó algún tipo de cirugía de cabeza y cuello.
- Paro cardiaco.

- Quemadura de la vía aérea.
- Quemaduras con una extensión mayor al 50% del área corporal.
- Paciente con enfermedad pulmonar obstructiva crónica.
- Pacientes remitidos de otra institución.
- Enfermedades desmielinizantes o de la placa neuromuscular.
- Pacientes requieran relajación neuromuscular.
- Pacientes con expectativa de vida menor a 180 días
- Pacientes que por criterio médico no se beneficiarían de tratamiento multimodal
- Pacientes que no ingresan a UCI por primera vez
- Pacientes que participan en otros ensayos clínicos de rehabilitación

Metodología

Recursos:

Humanos:

Terapeutas respiratorios

Materiales:

-DISPOSITIVOS E INSUMOS PARA LA SUCCIÓN:

INSUMO	REGISTRO INVIMA	LOTE
Manómetro de succión: Vacutron® Continuous Suction Regulator	NO APLICA	NO APLICA
1 Canister de 2000 cc marca Biolife	NO APLICA	NO APLICA
1 Receptal con válvula de 2000 cc marca Biolife	2017DM-0017348	10100923
2 tubos de succión siliconados	2021DM-0008052 R1	1044923
Sondas de succión de 12 French		
Cánula de aspiración desechable Yankauer	2019DMM0005249R1	2208011896
Sonda Succión cerrada Tubo endotraqueal 14 FR.	2018DM-0018691	20027825

-DISPOSITIVOS E INSUMOS PARA EL VENTILADOR MECÁNICO

INSUMO	REGISTRO INVIMA	LOTE
Circuito desechable adulto para el ventilador mecánico	2014DM-0012091	2022060001
Filtro intercambiador de calor y humedad HMEF (Humidificación pasiva)	2018DM-0018409	32222070
Sujetador tubo Anchor Fast	2021DM 0023253	2H252
AMBU PVC adulto	2018DM-0018376	2104282A / 2012031A
Mascara ventilación mecánica no invasiva talla M	20100DM-0007932	1450304
Mascara ventilación mecánica no invasiva talla L	20100DM-0007932	1450304

-DISPOSITIVOS E INSUMOS PARA TERAPIA RESPIRATORIA

INSUMO	REGISTRO INVIMA	LOTE
Incentivo Respiratorio	2020DM-0021287	10830821
Cough Assist Philips Respironics E70	NO APLICA	NO APLICA
Circuito asistente de tos	2018DM00182293	32302952
Mascara facial talla 5	2016DM-0014955	2023020001
Kit Micronebulizacion adulto	2018DM-0018538	32305306

-DISPOSITIVOS DE OXIGENOTERAPIA

INSUMO	REGISTRO INVIMA	LOTE
Canula nasal adulto	2022DM-0009342-R1	23S3100

Humidificador	2017DM-0000413-R1	10160523
Mascara Venturi	2017DM-000410R1	10160222
Mascara de no Reinhalacion	2016DM-0015089	21210618050
Mascara Simple	2017DM0000410R1	10220823
Circuito AIRVO 2	2014DM-0011418	2102557742
Agua esteril de 3000 ml	2020DM-011991-R3	SB23IB6
Tubo conector de Oxigeno	2016DM-0015089	20230505077
Interfase Canula alto flujo TALLA M	2014DM-0011418	2101649851
Interfase Canula alto flujo TALLA L	2014DM-0011418	2101451544

**No olvide que de acuerdo con el ventilador que utilice, se debe garantizar la correcta calibración del equipo, previo a la instauración en paciente.

NOMBRE VENTILADOR	MARCA
PB 980	Medtronic
PB840	Medtronic
SV800/ SV600	Mindray
AVEA	Vyaire Care Fusion
CARESCAPE R860	General Electric
Engstrom	General Electric
Hamilton S1	EMCO
Evita V500	Drager
Servo U	Maquet

**Tenga en cuenta que los ventiladores Engström y los Carescape R860 se deben calibrar con todos los insumos que se van a utilizar con el paciente: Filtro bacteriano/viral espiratorio – Circuito desechable – cubeta de medición de CO2 – Líneas de análisis y muestreo – Filtro HMEF – Sonda de succión cerrada. Los demás ventiladores se calibran solo con circuito desechable de ventilación mecánica.

Intervención

Técnicas de permeabilización de la vía aérea:

1. Manejo de secreciones en ventilación mecánica

1.1. *Succión abierta y succión cerrada:* existen dos métodos para realizar la succión de secreciones: (a) Sistema de Succión Cerrado: Se utiliza un circuito cerrado que permite succionar al paciente sin desconectar la ventilación mecánica. La sonda de succión está protegida por una camisa de plástico. El proceso implica introducir el catéter dentro del tubo endotraqueal y empujar la sonda hasta deslizar la camisa de plástico que recubre la sonda. La aspiración se aplica utilizando el pulgar y el índice mientras se retira el catéter. (b) *Sistema de Succión Abierto:* En este método, el catéter de succión se introduce cuando se desconecta temporalmente la ventilación mecánica. (8)

1.2. Ayuda Tusígena para Reducir Dolores Posquirúrgicos: esta intervención se enfoca en el manejo de la tos y tiene como objetivo reducir el dolor asociado con los ejercicios de tos. La fijación de la incisión quirúrgica juega un papel crucial en este proceso. La fijación se logra mediante el uso de las manos del paciente o almohadas estratégicamente colocadas. Esta práctica tiene el propósito de disminuir la presión intratorácica o intraabdominal en el contexto de procedimientos quirúrgicos. Al reducir la presión, se mitiga el dolor y el malestar al llevar a cabo ejercicios que fomentan la tos.

1.3. Técnica de Espiración Forzada o Aceleración de Flujos Espiratorios: esta técnica se emplea como una manera de modular el flujo espiratorio de manera rápida y forzada. Su mecanismo radica en el drenaje de las secreciones ubicadas en las vías aéreas mediasy centrales. La compresión dinámica se logra a través de una espiración forzada, lo que desplaza el punto de igual presión hacia regiones proximales. Este cambio incrementa el flujo espiratorio, lo que a su vez facilita el desplazamiento de las secreciones bronquiales y su eventual eliminación. El objetivo de esta técnica es favorecer la limpieza de las vías respiratorias, asegurando que las secreciones no queden estancadas en las áreas centrales y medias del árbol bronquial. (9)

1.4. Técnicas de Limpieza Bronquial Utilizando Flujos Espiratorios

1.4.1. Técnicas Espiratorias Lentas en Pacientes sin Ventilación Mecánica (ELPr): esta técnica se emplea cuando el paciente presenta secreciones en las vías aéreas medias. Consiste en realizar espiraciones lentas y controladas con la glotis abierta, de manera similar a la acción de hacer vaho en un espejo. El objetivo es desplazar las secreciones desde las vías aéreas medias hacia bronquios más proximales y la tráquea. Al mantener una espiración lenta y sin obstáculos estructurales en la salida del aire, las secreciones son arrastradas con mayor facilidad, contribuyendo a la limpieza de las vías respiratorias.

1.4.2. Técnicas Espiratorias en Pacientes Bajo Ventilación Mecánica: En pacientes bajo ventilación mecánica, el concepto de "bias flow" (flujo de corriente) se vuelve relevante. La diferencia entre el pico de flujo inspiratorio (PFI) y el pico de flujo espiratorio (PEF) determina la dirección del movimiento de las secreciones. Un aumento del bias flow inspiratorio moviliza las secreciones hacia el interior de los pulmones, mientras que un bias flow espiratorio las direcciona hacia la tráquea. Para lograr una diferencia PFE - PFI > 33 l/min, es necesario llevar a cabo los siguientes pasos de manera transitoria (hasta un máximo de 30 minutos): (a) Aumentar el volumen corriente en un rango de 50-150%

respecto al valor inicial. Se debe tener en cuenta que la presión pico no debe superar los 40 cm H2O y la presión meseta no debe exceder los 30 cm H2O. (b) Reducir la frecuencia respiratoria de manera que se mantenga el volumen minuto basal. (c) Disminuir el flujo inspiratorio a 20 litros/minuto. Si el paciente está en modo controlado por presión, aumentar el tiempo inspiratorio; si está en modo de presión de soporte, reducir el porcentaje de ciclado. (d) Estos ajustes se mantienen de manera transitoria durante un máximo de 30 minutos, después de lo cual se restablecen los parámetros a sus valores previo. (10)

1.5. Tos Dirigida: La tos dirigida implica un esfuerzo de tos voluntario con el propósito de aumentar la velocidad del flujo de aire durante la expulsión. Una tos eficaz debería generar un pico de flujo espiratorio (PEF) tusígeno superior a 160 l/min. Esta técnica tiene como objetivo imitar la efectividad de una tos espontánea, promoviendo una eliminación más eficiente de las secreciones bronquiales.

1.6. Espiración Lenta Prolongada (ELPr): la ELPr es una técnica pasiva y manual que modula el flujo espiratorio de manera lenta. Su objetivo principal es lograr un volumen espiratorio mayor que el obtenido durante una exhalación normal. La maniobra consiste en prolongar y completar la fase espiratoria normal, evitando así el colapso bronquial. Al prolongar esta fase, se facilita una depuración efectiva del árbol bronquial periférico, contribuyendo a la eliminación de secreciones.

1.7. Asistente de Tos o Cough Assist: el asistente de tos, también conocido como Cough Assist, es un dispositivo diseñado para simular una tos normal y asistir en el proceso. Este dispositivo reemplaza o ayuda en dos de las tres fases de la tos. Durante la faseinspiratoria, genera presión positiva que permite la insuflación de los pulmones. A continuación, se produce un cambio rápido a presión negativa durante la fase espiratoria para eliminar el aire insuflado, permitiendo la movilización y eliminación de las secreciones.El Cough Assist mide el pico de flujo de la tos (PFT). Se ha observado que el valor mínimonecesario para lograr una higiene óptima de las secreciones es de 160 L/min. Un pacientecon un pico de flujo de tos menor a 270 l/min necesita asistencia mecánica para la tos. Este soporte proporciona un apoyo adicional a los grupos musculares comprometidos en el proceso de tos.(11)

2. Técnicas de re expansión pulmonar

2.1. Ejercicios Diafragmáticos en Pacientes sin Ventilación Mecánica: los ejercicios diafragmáticos se enfocan en mejorar la función del diafragma durante el proceso respiratorio. En la respiración normal, durante la inspiración, la parte superior de la pared abdominal se proyecta hacia adelante debido al descenso del diafragma, mientras que en la espiración, el abdomen se desplaza hacia atrás. La técnica consiste en los siguientes pasos: (a) Inspiración profunda por la nariz, dirigiendo el aire hacia el abdomen. (b) Sostener la respiración durante aproximadamente 3 segundos. (c) Realizar una espiración lenta y prolongada a través de los labios fruncidos, retrayendo el abdomen.

Durante la ejecución, la mano del terapeuta respiratorio guía el movimiento del aire. La posición del paciente influye en la movilización específica del diafragma: (a) Decúbito dorsal: Se enfoca en la porción posterior del diafragma. (b) Decúbito lateral derecho: Se dirige al hemidiafragma derecho. (c) Decúbito lateral izquierdo: Se dirige al hemidiafragma izquierdo.

2.2. Ejercicios de Ventilación Dirigida Abdomino-Diafragmática: esta técnica apunta a mejorar la ventilación en las bases pulmonares. El terapeuta respiratorio se sitúa al lado del paciente y guía el ejercicio de la siguiente manera: (a) Inspiración lenta a través de la nariz. (b) Elevación simultánea del abdomen durante la inspiración. (c) Con la espiración, se contrae el abdomen hacia adentro.

El terapeuta proporciona apoyo constante y guía durante todo el ejercicio.

2.3. Incentivo Respiratorio: el incentivo respiratorio es un dispositivo de entrenamiento que utiliza retroalimentación visual para promover inspiraciones máximas sostenidas. Su objetivo es mejorar la función respiratoria, aumentando la presión intratorácica negativa y expandiendo los alvéolos al inhalar profundamente. Este dispositivo contribuye a: mejorar la ventilación pulmonar, aumentar la expansión de la pared torácica, reducir la pérdida de la función pulmonar y sus complicaciones asociadas.

El dispositivo de incentivo consiste en una boquilla conectada a un sistema de flujo inspiratorio con tres niveles de flujo: 600 ml/s, 900 ml/s y 1200 ml/s. El paciente inhala por la boquilla, el aire fluye a través del dispositivo y desplaza las esferas según el flujo inspiratorio. Una tasa de flujo de 1200 ml/s indica un flujo máximo alcanzado. (12)

3. Técnicas de fortalecimiento muscular respiratorio

3.1. Entrenamiento musculas diafragmático en paciente ventilado por medio de trigger por presión: El trigger del ventilador mecánico, es el mecanismo con el que el ventilador es capaz de detectar el esfuerzo respiratorio del paciente. La sensibilidad puede establecerse por presión o por flujo. En la sensibilidad por presión, el esfuerzo inspiratorio del paciente produce una caída programada, la sensibilidad por presión, se ajustará al 20% de la primera NIF registrada en el paciente, asegurando una frecuencia respiratoria entre 20 y 30 respiraciones/minuto y el volumen tidal de 4 a 6 ml / kg. Realizando 2 sesiones por día, iniciando con 5 min y aumentando 5 min por sesión hasta completar 30 min. Si una paciente toleró 30 min del entrenamiento muscular inspiratorio, La próxima sesión se realiza con el aumento de la sensibilidad de disparo por 10% de la NIF inicial durante 5 min. (13)

APARTADO DE PROFUNDIZACIÓN

- **VMNI POS EXTUBACIÓN:** La ventilación mecánica no invasiva es un recurso sencillo y plausible en este contexto, ya que ha demostrado evitar la intubación en los siguientes escenarios:

    1. Como medida profiláctica: en pacientes que han tenido PVE con destete prolongado o difícil que tienen alto riesgo de reintubación. Ejemplo: pacientes con Ventilación Mecánica prolongada, SDRA, EPOC, Falla cardiaca
    2. Tratamiento y mantenimiento: pacientes que fueron extubados y desarrollaron insuficiencia respiratoria aguda en las primeras 48 horas pos extubación.

- **INCENTIVO RESPIRATORIO (IR):**

El incentivo respiratorio es un dispositivo que promueve las respiraciones lentas y profundas es simple y fácil de manejar, teniendo como objetivo principal el prevenir y tratar la atelectasia en pacientes que se encuentran predispuestos a respiraciones superficiales que provoquen zonas pequeñas de colapso o atelectasias pasivas ya sea por reposo o posquirúrgicas útil en pacientes con enfermedad neuromuscular.

Los incentivos respiratorios disponibles en el mercado de clasifican en dos tipos: los incentivos con desplazamiento de volumen y los incentivos dependientes de flujo, los primeros miden la cantidad de volumen de aire desplazado en un esfuerzo inspiratorio y los segundos miden el flujo inspiratorio de forma cualitativa que realiza el paciente en una inspiración profunda. La eficacia del incentivo respiratorio va de la mano de dos condiciones:

1. Educación y supervisión adecuadas en la realización y ejecución de la técnica.

2. Complementar el incentivo respiratorio con ejercicios respiratorios ya que la evidencia clínica ha demostrado la eficacia de la combinación con ejercicios de ventilación dirigida y fortalecimiento diafragmático.

En este estudio utilizaremos el incentivo respiratorio flujo dependiente, el cual consta de una boquilla y un tubo corrugado conectado a un recolector formado por tres tubos donde se encuentran 3 esferas de plástico livianas. A medida que el paciente realiza el esfuerzo inspiratorio por la boca se crea una presión negativa dentro de los tubos lo que permite que se eleven. Este, está diseñado para que el número de esferas y el nivel de elevación DEPENDA DE LA MAGNITUD DEL FLUJO INSPIRATORIO. La meta de flujo que debe ser alcanzado elevando las 3 esferas es de 1200 ml/s, siendo la primera esfera: 600 ml/s, la segunda esfera: 900 ml/s y la tercera esfera arriba mencionada: 1200 ml/s.

- **COUGH ASSIST POS EXTUBACIÓN**: *La directriz para iniciar dispositivo de asistente de tos será dirigido según el pico flujo de tos medido previo a extubación.*

El Cough-Assist es una asistencia mecánica a la tos. Pertenece a las máquinas de insuflación/exsuflación (MI-E) pulmonar mecánica (Mechanical Insufflation Exsufflation). Es un dispositivo eléctrico-mecánico portátil que favorece y reproduce el mecanismo de la tos fisiológica. Interviene desde el inicio hasta el fin del ciclo de la tos. En efecto, consiste a una ayuda a la inspiración en presión positiva (hiperinsuflación), seguido inmediatamente por una espiración forzada en presión negativa (hiperexsuflación). Por lo tanto, este dispositivo simula la tos fisiológica, y proporciona ciclos respiratorios los cuales alternan presiones positivas, pausas inspiratorias, y cambios abruptos a presiones negativas. El objetivo es mejorar la eficacia a la tos, facilitando así la movilización de las secreciones en el árbol bronquial, y después su expectoración.

Indicaciones

-El Cough-Assist está indicado con pacientes que no pueden toser ni eliminar las secreciones de forma efectiva debido a una reducción del flujo espiratorio máximo, o cuando el pico de flujo de tos en situación inestable sea inferior a 270 l/min.

-Fibrosis quística, bronquiectasias y atelectasias por obstrucción de secreciones.

-La literatura describe con frecuencia su uso en las enfermedades neuromusculares, pero es interesante en cualquier tipo de paciente que tiene una debilidad general, como puede ser el caso de los pacientes en cuidados intensivos o en post-operatorio.

-Al inicio, fue desarrollado para ayudar a pacientes con deterioro de la tos fisiológica y, actualmente, se considera una de las técnicas más eficaces para eliminar secreciones de las vías respiratorias en pacientes con enfermedades neuromusculares.

Contraindicaciones

El Cough-Assist está contra indicado en caso de:

Neumotórax no tratado o susceptibilidad al neumotórax

Hipotensión y criterios hemodinámicos inestables

Neumomediastino

Hemoptisis importante

Bullas enfisematosas

Inestabilidad de la vía aérea

Crisis de broncoespasmo

Laringomalacia Severa

Ejecución

Se utilizará el dispositivo Cough-Assist E70 de la marca Philips insuflador y exuflador mecánico

– El objetivo es realizar una serie de 4 a 6 ciclos seguidos de un descanso dependiendo de la condición del paciente.

– Los primeros 2-3 ciclos son ciclos de hiperinsuflación y de exhalación durante los cuales el paciente debe lograr a un aumento del flujo espiratorio.

– Los últimos 2-3 ciclos son ciclos de hiperinsuflaciones seguido por un esfuerzo activo de la tos por parte del paciente durante la exuflación.

– La serie se puede renovar de 4 a 6 veces o se puede intercalar con una técnica de desobstrucción periférica.

– La duración de la sesión depende de la obstrucción del paciente y su estado de fatiga.

Programación cough assist

Aunque los valores de los parámetros del Cough-Assist dependen totalmente de los pacientes, los parámetros ajustables son el nivel de presión y los tiempos de aplicaciones.

En la última versión del Cough-Assist E70, las presiones positivas y negativas pueden ser ajustadas entre +70 y -70 cm H2O.

Es preferible empezar la sesión con presiones débiles (entre +15 y +20 cm H2O, y -20 a -25 H2O) para acostumbrar el paciente.

Más altas son las presiones, mayores son los movimientos del aire.

Encontramos en la literatura buenos resultados con bajas presiones (+ 30 a - 30 cm H2O)

De manera general, +40/-40cm H2O ya son suficiente para producir tos asistida bien tolerada en la mayoría de los pacientes restrictivos. También, se puede ajustar los tiempos de inspiración, espiración y de pausa (0 a 5 segundos). El tiempo inspiratorio varía entre 2 y 3 segundos, mientras que el tiempo espiratorio entre 3 y 4 segundos. (32)

• ***TERAPIA CONVENCIONAL:*** *Se denomina al abordaje completo por parte de Terapia respiratoria llevando a cabo las siguientes intervenciones:*

- Incentivo respiratorio
- Ejercicios respiratorios con ventilación dirigida, es decir, respiracionesprofundas redirigidas a los diferentes segmentos pulmonares a nivel apical, medio-lateral y basal.
- Reacondicionamiento diafragmático con ejercicios de fortalecimiento del principal músculo de la respiración.
- Ejercicios de tos: enseñar al paciente a toser de forma eficaz, posterior a ejercicios de ventilación dirigida. Con el objetivo de Limpiar el árbol bronquial.

Los sistemas de oxigenoterapia se seleccionan teniendo en cuenta las necesidades y circunstancias clínicas del paciente (29):

**DISPOSITIVO**	**PROS**	**CONTRAS**
**Cánula Nasal (FIO2 23-36%)**	Confortable<br>Bajo costo<br>El paciente puede hablar y comer con tranquilidad	FIO2 limitada e irreal por ser dispositivo de bajo flujo<br>Depende de la ventilación minuto/ apertura oral
**Mascara facial simple (FIO2 35-60%)**	Bajo costo<br>FIO2 independiente de la apertura oral	No es confortable<br>Riesgo de hipercapnia con flujos < 5 litros/minuto<br>El paciente no puede comer ni hablar con tranquilidad

**Mascara Venturi (FIO2 24-50%)**	Reduce el riesgo de hiperoxia e hipercapnia<br>Escasa formación de aerosoles	Experiencia profesional necesaria<br>Los colores de los Venturi están ajustados según fabricante<br>La máscara impide hablar y comer con tranquilidad
**Máscara con reservorio (FIO2 60-90%)**	Flujos de oxígeno altos<br>Muy útil en situaciones de emergencia	Riesgo de hipercapnia con flujos < 5 litros/min<br>Poco confortable
**Cánula de alto flujo (FIO2 21-100%)**	FIO2 estable<br>Muy confortable<br>Lavado de CO2 en el espacio muerto anatómico	Monitoria estricta<br>Alto costo

Intervención terapia respiratoria

Acorde con el modelo de atención de Terapia Respiratoria en la UCI del Hospital Universitario de la Fundación Santafé de Bogotá (FSFB) un paciente inicia intervención cuando el médico tratante solicita interconsulta a Terapia respiratoria. Los terapeutas respiratorios realizan la evaluación e inician el plan de tratamiento.

En el marco del proyecto SECEC-2023-129, habrá dos grupos de intervención: terapia multimodal temprana y terapia multimodal tardía. Con respecto a la primera, esta se instaurará en las primeras 24 horas posteriores a la intubación de un paciente en UCI. La segunda se instaurará conforme al modelo de atención de terapia respiratoria en paciente en cuidado crítico de la Fundación Santa Fe de Bogotá. Es decir, cuando el médico tratante solicita interconsulta por Terapia Respiratoria.

Independientemente del grupo en el cual sea asignado el paciente, se realizarán 3 sesiones (en el turno de la mañana, tarde y noche) los siete días de la semana, con una duración por sesión:

Dividiéndose en 3 grandes categorías:

- Técnicas de permeabilización de la vía aérea
- Técnicas de re expansión pulmonar
- Técnicas de fortalecimiento muscular respiratorio

**INTERVENCIÓN**	**DURACIÓN DE LA SESIÓN**

PACIENTE BAJO VENTILACIÓN MECANICA INVASIVA	
Succión de secreciones por tubo endotraqueal	3 minutos
Succión de secreciones orofaringeas + higiene de cavidad oral	5 minutos
Técnica de higiene bronquial usando flujos espiratorios en ventilación mecánica	15 minutos
Entrenamiento muscular diafragmático en paciente ventilado por medio de trigger por presión	5 minutos
Tiempo total empleado: ***28 minutos***	
**PACIENTE YA SIN VENTILACIÓN MECÁNICA INVASIVA**	
Ejercicios diafragmáticos en paciente sin ventilación mecánica	5 minutos
Ejercicios de ventilación dirigida	5 minutos
Incentivo respiratorio	3 minutos
Técnicas de espiración lenta prolongada	3 minutos
Técnicas de tos dirigida	3 minutos

Técnicas de espiración forzada	3 minutos
Utilización de asistente de tos o *COUGH ASSIST*	5 minutos
TIEMPO TOTAL EMPLEADO: ***27 minutos***	

RESPONSABLE, FRECUENCIA Y LUGAR

1. Responsable: Terapeuta Respiratorio
2. Frecuencia: Según orden médica y/o criterio del profesional de Terapia Respiratoria
3. Lugar: Unidad de Cuidado Intensivo Adultos (Tercer y cuarto piso)

Referencias Bibliográficas

1. Abplanalp LA, Ionescu F, Calvo-Ayala E, Yu L, Girish ·, Nair B. Static Respiratory System Compliance as a Predictor of Extubation Failure in Patients with Acute Respiratory Failure. Lung [Internet]. 2023 [cited 2023 Jun 26];201:309–14. Available from: https://doi.org/10.1007/s00408-023-00625-7

2. Smina M, Salam A, Khamiees M, Gada P, Amoateng-Adjepong Y, Manthous CA. Cough peak flows and extubation outcomes. Chest [Internet]. 2003 Jul 1 [cited 2023 Jun 20];124(1):262–8. Available from: http://journal.chestnet.org/article/S0012369215360190/fulltext

3. Dres M, Dubé BP, Dubé D, Mayaux J, Delemazure J, Reuter D, et al. Coexistence and Impact of Limb Muscle and Diaphragm Weakness at Time of Liberation from Mechanical Ventilation in Medical Intensive Care Unit Patients. 2017 [cited 2023 Jun 26]; Available from: www.atsjournals.org

4. Zein H, Baratloo A, Negida A, Safari S. Emergency 2016; 4 (2): 65-71 Ventilator Weaning and Spontaneous Breathing Trials; an Educational Review. [cited 2023 Jul 25]; Available from: www.jemerg.com

5. Subirà C, Hernández G, Vázquez A, Rodríguez-Garciá R, González-Castro A, Garciá C, et al. Effect of Pressure Support vs T-Piece Ventilation Strategies During Spontaneous Breathing Trials on Successful Extubation Among Patients Receiving Mechanical Ventilation: A

Randomized Clinical Trial. JAMA [Internet]. 2019 Jun 11 [cited 2023 Jul 25];321(22):2175–82. Available from: https://pubmed.ncbi.nlm.nih.gov/31184740/

6. Zarrabian B, Wunsch H, Stelfox HT, Iwashyna TJ, Gershengorn HB. Liberation from Invasive Mechanical Ventilation with Continued Receipt of Vasopressor Infusions. Am J Respir Crit Care Med [Internet]. 2022 May 1 [cited 2023 Jul 25];205(9):1053–63. Available from: https://pubmed.ncbi.nlm.nih.gov/35107416/

7. Jaber S, Jung B, Chanques G, Bonnet F, Marret E. Effects of steroids on reintubation and post-extubation stridor in adults: meta-analysis of randomised controlled trials. Crit Care [Internet]. 2009 Apr 3 [cited 2023 Jul 25];13(2):R49. Available from: /pmc/articles/PMC2689493/

8. SISTEMAS DE ASPIRACIÓN DE SECRECIONES CERRADOS: INDICACIONES Y CUIDADOS. CLOSED SECRETION SUCTIONING SYSTEM: INDICATIONS AND CARE.

9. Roldan Lysbeth, Sarmiento Piedad. Terapia Respiratoria para profesionales todo lo que debes saber. Vol. 1. Bogotá: Distribuna Editorial Medica; 2020. 185–187 p.

10. Volpe MS, Guimarães FS, Morais CC. Airway Clearance Techniques for Mechanically Ventilated Patients: Insights for Optimization. 2020;

11. Herrero Maria Victoria. Pressures used and peak cough flow during mechanical cough assistance. Revista Cubana de Medicina Militar. 2020 Mar;

12. Zeng P, Lin Y, Chen Y, Tan G. Effects of incentive spirometry respiratory trainer device on lung recruitment in non-intubated mechanical ventilation moderate ARDS patients: A retrospective study. Heliyon. 2023 May;9(5):e16073.

13. Acosta Ortega Adriana. PROPUESTA DE UN PROTOCOLO PARA EL ENTRENAMIENTO MUSCULAR RESPIRATORIO AL INTERIOR DE LAS UNIDADES DE CUIDADO INTENSIVOS. FASE II. Universidad Iberoamericana; 2018.

14. Poor H. Respiratory Mechanics. In: Basics of Mechanical Ventilation. Cham: Springer International Publishing; 2018. p. 1–10.

15. Amato MBP, Meade MO, Slutsky AS, Brochard L, Costa ELV, Schoenfeld DA, et al. Driving Pressure and Survival in the Acute Respiratory Distress Syndrome. New England Journal of Medicine [Internet]. 2015 Feb 19 [cited 2023 Jul 31];372(8):747–55. Available from: https://www.nejm.org/doi/10.1056/NEJMsa1410639

16. Mora Carpio AL, Mora JI. Ventilator Management. 2023.

17. Cardozo SL, Sanabria O. Índices de oxigenación: más allá de la PaO2/FiO2 como herramienta ideal. Acta Colombiana de Cuidado Intensivo. 2022 Jul 1;22(3):227–36.

18. Jiménez Duran DP, Cruz Mosquera FE, Arango Arango AC, Ávila Ovalle IJ. Medición del neumotaponador y su influencia en la prevención de complicaciones laringotraqueales: a propósito de un caso de intubación prolongada (56 días). Acta Colombiana de Cuidado Intensivo. 2018 Jan 1;18(1):66–9.

19. Haudebourg AF, Moncomble E, Lesimple A, Delamaire F, Louis B, Mekontso Dessap A, et al. A novel method for assessment of airway opening pressure without the need for low-flow insufflation. Crit Care. 2023 Jul 7;27(1):273.

20. Bertoni M, Telias I, Urner M, Long M, Del Sorbo L, Fan E, et al. A novel non-invasive method to detect excessively high respiratory effort and dynamic transpulmonary driving pressure during mechanical ventilation. Crit Care. 2019 Dec 6;23(1):346.

21. Grassi A, Telias I, Bellani G. Monitoring the Patient During Assisted Ventilation. In: Mechanical Ventilation from Pathophysiology to Clinical Evidence. Cham: Springer International Publishing; 2022. p. 61–73.

22. Domínguez Cherit G, Rivero Sigarroa E, Vidal Mayo J de J, Mercado Velázquez P, Nicolás Martínez EL. Impacto de las asincronías en el pronóstico del paciente bajo ventilación mecánica invasiva. Medicina Crítica. 2020;34(5):273–8.

23. Jubran A. Advances in Respiratory Monitoring During Mechanical Ventilation. Chest. 1999 Nov;116(5):1416–25.

24. Garzon Posada Natalia. Desenlaces de la escala Omaha+ en los pacientes en cuidado intensivo en la fundación santa fe de Bogotá. Repositorio Universidad del Rosario. 2016;

25. Salam A, Tilluckdharry L, Amoateng-Adjepong Y, Manthous CA. Neurologic status, cough, secretions and extubation outcomes. Intensive Care Med. 2004 Jul 4;30(7):1334–9.

26. Matamis D, Soilemezi E, Tsagourias M, Akoumianaki E, Dimassi S, Boroli F, et al. Sonographic evaluation of the diaphragm in critically ill patients. Technique and clinical applications. Intensive Care Med. 2013 May 24;39(5):801–10.

27. Gómez Grande ML, González Bellido V, Olguin G, Rodríguez H. Manejo de las secreciones pulmonares en el paciente crítico. Enferm Intensiva. 2010 Apr;21(2):74–82.

28. Smailes ST, McVicar AJ, Martin R. Cough strength, secretions and extubation outcome in burn patients who have passed a spontaneous breathing trial. Burns. 2013 Mar;39(2):236–42.

29. Gottlieb J, Capetian P, Hamsen U, Janssens U, Karagiannidis C, Kluge S, et al. German S3 Guideline: Oxygen Therapy in the Acute Care of Adult Patients. Respiration. 2022;101(2):214–52.

30. Fajardo-Campoverdi A, González-Castro A, Adasme-Jeria R, Roncalli-Rocha A, Ibarra M, Chica-Meza C, et al. Mechanical ventilator liberation protocol. Recommendation based on review of the evidence. Journal of Mechanical Ventilation. 2023 Mar 15;4(1):31–41.

31. Cairo JM. Mosby’s respiratory care equipment. 11th ed. San Luis; 2021.

32. Chatwin M, Simonds AK. Long-Term Mechanical Insufflation-Exsufflation Cough Assistance in Neuromuscular Disease: Patterns of Use and Lessons for Application. Respir Care. 2020 Feb;65(2):135–43.

COMPONENTE TERAPIA OCUPACIONAL

Coordinadoras:

- ❖ Marisol Murillo; email: marisol.murillo@fsfb.org.co
- ❖ Sara Stefania Martínez; email: sahara.martinez@fsfb.org.co

Justificación

Los procesos de intervención de Terapia Ocupacional se direccionan en evitar y mitigar el impacto de los procesos patológicos agudos en la autonomía e independencia del paciente en sus actividades cotidianas significativas de acuerdo a su historia y rol ocupacional, asi como evitar deterioros futuros en componentes neurocognitivos, sensitivomotores y psicosociales (Ares Senra et al., 2014); en las unidades de cuidado intensivo se enfocaran en brindar estimulación multisensorial (énfasis en inputs táctil, propioceptivos, visuales, olfativos y auditivos), estimulación cognitiva y fomento de participación en actividades significativas de la vida diaria a partir de la información recepcionada por el familiar, paciente y/o cuidador y la reportada en la historia clínica mediados por actividades con significado (Celis et al., 2014).

Para ello si el paciente se encuentra en un RASS menor o igual a -3 se realizará actividad de estimulación multisensorial de tipo táctil, propioceptiva, vestibular, gustativo, auditiva y olfativa asistida completamente por el terapeuta que guarden una correlación con tarea de la vida diaria simulada incorporando patrones motores de miembros superiores (énfasis en patrones primarios mano-cabeza, mano-boca, mano-homolateral, mano-contralateral y mano-perine) empleados en dichas tareas asi como el cuidado y protección articular y de piel; se ajustará la intervención de manera unimodal y posteriormente multimodal según tolerancia del paciente en cama; adicionalmente al finalizar cada sesión se garantizara posicionamiento de los segmentos apendiculares (miembros superiores e inferiores) en

ángulos funcionales de reposo que generen confort y evitar contracturas o edema. En caso de presencia de familiar se educa y retroalimenta el que acción terapéutica se hizo y los objetivos de la misma, así mismo se brinda recomendaciones para brindar acercamiento al paciente y participar mínimamente en co-cuidado con énfasis en estímulos táctiles (acicalado cuidado de piel) y auditivos (con voz, audios de máximo 2 minutos de duración continua)

Para RASS entre -2 y -1 los procesos de intervención se realizaran con actividades de manera pasivo-asistido e involucran adicional al estimulo sensorial se hace énfasis en procesos de retroalimentación cognitiva de reconocimiento de elementos y generación de tareas cotidianas con seguimiento instruccional de máximos dos comandos con uso de patrones integrales de movimiento de alcance, agarre, soltar voluntario o rudimentario y lanzamientos con o sin propulsión énfasis en planos frontales y laterales medios en cama.

Cuando el paciente se encuentra en un RASS de 0 y +1, la intervención se centra en participación del paciente en actividades de manera activa o autónoma con mínimos apoyos por parte del terapeuta según tolerancia del paciente con mayor énfasis en la autonomía en tareas cotidianas e integración de comandos sensoriales y cognitivos de baja/mediana/alta complejidad según evolución y respuestas del paciente que vincula ejecución de patrones de miembros superiores (primarios, secundarios e integrales) previamente trabajados en todos los planos anatómicos tanto en cama/a borde de cama, como en silla según avance de equipo de rehabilitación. Se educa al paciente y familia frente a la importancia de promover autonomía en actividades de autocuidado y búsqueda de herramientas de comunicación con uso de pictogramas o actos grafomotores con tablero, brindando apoyo según evolución del paciente y avance del proceso terapéutico.

Cuando el paciente se encuentra en un RASS superior o igual a +2 las sesionesterapéuticas se enfocan en modulación conductual y ambiental (físico y familia) y fomentode conexión con el medio mediante actividades de estimulación multisensorial de tipo inhibitoria sumado a estimulación cognitiva direccionados a orientación y participación en tareas significativas del paciente de manera activo-asistida en cama. Educación a familiar frente a procesos de orientación continua con enriquecimiento de estímulos sensoriales queconverjan en una misma tarea de tipo visual, auditivo, táctil y propioceptivo.

Se aclara que todo proceso educativo y recomendaciones se retroalimentaran de manera teórico-practico paralelo al proceso de intervención.

Alcance

Pacientes seleccionados para el proyecto de investigación: “Asociación entre terapia multimodal temprana y días de ventilación mecánica en la unidad de cuidado intensivos de la Fundación Santa Fe de Bogotá: un ensayo clínico controlado aleatorizado (SECEC-2023-129).

Población objeto

Criterios de inclusión

- Pacientes hospitalizados en la unidad de cuidado intensivo adulto de la Fundación Santa Fe de Bogotá que requieran ventilación mecánica invasiva a través de un tubo endotraqueal durante un periodo mayor de 72 horas y que se espere que continue por al menos 24 horas.
- Índice de Barthel mayor o igual a 70.

Criterios de Exclusión:

- Pacientes que requieran ventilación mecánica invasiva a través de una cánula de traqueostomía o tubo nasotraqueal.
- Pacientes a los que se realizó algún tipo de cirugía de cabeza y cuello.
- Paro cardiaco.
- Quemadura de la vía aérea.
- Quemaduras con una extensión mayor al 50% del área corporal.
- Paciente con enfermedad pulmonar obstructiva crónica.
- Pacientes remitidos de otra institución.
- Enfermedades desmielinizantes o de la placa neuromuscular.
- Pacientes requieran relajación neuromuscular.
- Pacientes con expectativa de vida menor a 180 días
- Pacientes que por criterio médico no se beneficiarían de tratamiento multimodal
- Pacientes que no ingresan a UCI por primera vez
- Pacientes que participan en otros ensayos clínicos de rehabilitación

Metodología

Recurso humano:

Terapeutas Ocupacionales

Material:

- Computadores
- Historia Clínica
- Valoración: Observación directa y escalas
- Kit de estimulación multisensorial, material didáctico cognitivo y motor fino y utensilios y elementos de co-cuidado.

Intervención

Estimulación sensorial:

El terapeuta realizara la presentación de estímulos organizados al paciente por espacios mínimos de 20 a 30 segundos de tipo táctil (texturas, tacto ligero, tacto-presión y cepilleo), propioceptivo (tracción/compresión, vibración y asistencia de patrones motores), auditivo (música, voz familiar/sonidos aislados y audios), vestibular (movimientos dirigidos cefálicos que acompañan la localización de otros estímulos), olfativo (fragancias u olores cotidianos),

gustativo (correlacionados a inputs olfativos y visuales coordinados con fonoaudiologia) y visuales (formas, colores, imágenes cotidianas y fotografías) iniciando de manera unimodal o unisensorial por sesión y según tolerancia del paciente incrementar cantidad de estímulos orientados o inmersos en una actividad cotidiana con elementos significativos para el paciente según historia ocupacional del mismo. (Ares Senra et al., 2014; Celis- Rodríguez et al., 2013; Costigan et al., 2019; Hellweg, 2012; Martínez-Leiva et al., 2020, 2020, 2020; Sanz et al., 2004; Silva Barbosa & Da Silva Reis, 2017; Weinreich et al., 2017)

Estrategias de Confort

Posicionamiento en reposo de los diferentes segmentos corporales apendiculares con apoyos articulares para evitar sobreesfuerzos osteotendinosos y/o contracturas, dolor y edema con énfasis en miembro superiores e inferiores a nivel distal manteniendo ángulos que generan confort o posicionamiento neutro y apoyo externo a estabilización de segmentos articulares en los diferentes cambios posicionales fomentando aproximación de miembros superiores a línea media y eliminar puntos de presión, uso de ortesis o aditamentos en caso de requerirse, fomento de conciencia corporal y modificaciones medioambientales en términos sensoriales. (Ares Senra et al., 2014; Celis et al., 2014; Celis-Rodríguez et al., 2013; Martínez-Leiva et al., 2020; Moreno-Chaparro et al., 2017; Provancha-Romeo et al., 2019)

Estimulación Cognitiva:

Actividades semiestructuradas según el nivel de sedación del paciente (Álvarez et al., 2017; Celis-Rodríguez et al., 2013; Costigan et al., 2019; Martínez-Leiva et al., 2020; Sheard et al., 2022, 2022; Silva Barbosa & Da Silva Reis, 2017, 2017; Weinreich et al., 2017) por niveles de complejidad así:

- Baja: reconocimiento de elementos presentados o búsqueda de los mismos según instrucciones de un comando, descripción de características percibidas, uso cotidiano a partir de diálogos con respuestas afirmativas o negativas y de selección; reconocimiento de familiares y escenario hospitalario así como elementos o dispositivos en su habitación y su cuerpo para la supervivencia.

- Mediana: actividades que demandan razonamiento básico, funciones atencionales, mnésicas, visoespaciales a través del juego o desafíos cognitivos con 2 o 3 comandos y doble tareas, generalmente con manipulación de elementos según el avance en patrones motores y destrezas manipulativas y con apoyo visual continuo o alternante según necesidad del paciente.

- Alta: Actividades que implican razonamiento abstracto, análisis de información y resolución de desafíos con uso de funciones cognitivas básicas con mayor grado de complejidad según reserva cognitiva del paciente, actividades cotidianas y roles que desempeñaba previo a la hospitalización actual.

Funcionalización de patrones motores de miembros superiores

El terapeuta incorpora educación y entrenamiento para recuperación o mantenimiento de movilidad funcional (AOTA, 2020; Celis et al., 2014; Celis-Rodríguez et al., 2013; Hellweg, 2012; Martínez-Leiva et al., 2020; Rapolthy-Beck et al., 2021, 2022; Schweickert et al., 2009; Silva Barbosa & Da Silva Reis, 2017; Weinreich et al., 2017) en especial patrones motores de miembros superiores requeridos en actividades cotidianas en especial acciones de autocuidado tales como alimentación, baño, acicalado y vestido (superior e inferior), los cuales son:

Patrones primarios: mano-boca, mano-cabeza, mano-homolateral, mano-contralateral, mano periné.

Patrones secundarios: mano-rodilla, mano-pie, mano-cintura y mano-espalda.

Patrones integrales: Alcances (plano sagital y coronal anterior/posterior medio, superior e inferior), soltar (rudimentario o con precisión), lanzar (con y sin propulsión) y agarres (palmares, Penta, tri y bidigitales).

Así mismo se continua con la mejora de destrezas manipulativas en donde intervienen procesos de coordinación sensitivomotor y cognitiva, asi como modulación de fuerza prensil énfasis en la disminución de compensaciones, planeación y organización y coordinación en cada praxia motora necesaria en las actividades aumentando la complejidad por planos anatómicos, partiendo de una ejecución en plano medio, inferior y superior según evolución y tolerancia del paciente.

Promoción de autonomía en Actividades de la vida diaria

El terapeuta promueve el mayor grado de autonomía e independencia en las actividades cotidianas del paciente tanto en autocuidado como en actividades instrumentales y de ocio y esparcimiento en donde se integran las demandas cognitivas, sensoriales y ejecución de patrones motores de miembros superiores y destrezas manipulativas, para ello se estructura cada sesión en fases y tareas con ejemplificación y asistencia odireccionamiento motor inicial, ejecución por parte del paciente y retroalimentación continua de tipo visual, verbal y en espejo para ajustes y adaptaciones que el paciente deba realizar a favor o en contra de gravedad según evolución y tolerancia del paciente, incluye cambios posicionales necesarios para la realización de las tareas a desarrollar durante la sesión.(Álvarez et al., 2017; Hellweg, 2012; López, 2010; Martínez-Leiva et al., 2020; Moreno-Chaparro et al., 2017; Rapolthy-Beck et al., 2021, 2022; Schweickert et al., 2009; Sheard et al., 2022, 2022; Silva Barbosa & Da Silva Reis, 2017; Weinreich et al., 2017)

Educación al paciente y la familia y/o cuidadores

Proceso de orientación continua de manera practica con el objetivo de garantizar una participación activa, garantizar una mayor adherencia del paciente y su familia en el proceso terapéutico y garantizar un co-cuidado seguro, según el nivel de sedación del paciente al inicio y finalización de cada intervención se explica brindan recomendaciones pertinentes a cada etapa del proceso de recuperación funcional en línea con las actividades

realizadas durante la sesión y acordes a la capacidad funcional del paciente según su evolución clínica, de tal forma que se disminuya la sobre asistencia en tareas cotidianas y se fomente la participación ocupacional del paciente. (Ares Senra et al., 2014; Celis-Rodríguez et al., 2013; López, 2010; Martínez-Leiva et al., 2020; Rapolthy-Beck et al., 2022)

Intervención terapia ocupacional

Acorde con el modelo de atención de Terapia Ocupacional en la UCI del Hospital Universitario de la Fundación Santafé de Bogotá (FSFB) un paciente inicia intervención cuando el médico tratante solicita interconsulta a Terapia Ocupacional. Los terapeutas ocupacionales realizan la evaluación e inician el plan de tratamiento.

En el marco del proyecto SECEC-2023-129, habrá dos grupos de intervención: terapia multimodal temprana y terapia multimodal tardía. Con respecto a la primera, esta se instaurará en las primeras 24 horas posteriores a la intubación de un paciente en UCI. La segunda se instaurará conforme al manejo estándar por parte de terapia ocupacional en cuidado crítico de la Fundación Santa Fe de Bogotá. Es decir, cuando el médico tratante solicita interconsulta por Terapia Ocupacional.

Independientemente del grupo en el cual sea asignado el paciente, se realizarán 1 sesión al día durante RASS – o igual a -3; cuando inicia el weaning RASS -2 a -1 o superior a +1 se realizarán 2 sesiones diarias distribuidas en mañana y tarde los siete días de la semana, con una duración por sesión de 30 minutos

Por favor escribir cuánto dura cada sesión y si la duración varía en función de las características de los pacientes.

RESPONSABLE, FRECUENCIA Y LUGAR

1. Responsable: Terapeuta Ocupacional
2. Frecuencia: Según orden médica y/o criterio del profesional de Terapia Ocupacional
3. Lugar: Unidad de Cuidado Intensivo Adultos (Tercer y cuarto piso)

Referencias Bibliográficas

Álvarez, E. A., Garrido, M. A., Tobar, E. A., Prieto, S. A., Vergara, S. O., Briceño, C. D., & González, F. J. (2017). Occupational therapy for delirium management in elderly patients without mechanical ventilation in an intensive care unit: A pilot randomized

clinical trial. *Journal of Critical Care*, *37*, 85-90. https://doi.org/10.1016/j.jcrc.2016.09.002

AOTA. (2020). *Marco de Trabajo para la Practica de Terapia Ocupacional: Dominio y practica 4 Ed*. AOTA 2020 CUARTA EDICION - Marco de Trabajo para la Practica de Terapia Ocupacional: Dominio y - Studocu. https://www.studocu.com/es/document/universidad-de-burgos/la-terapia-ocupacional-en-los-trastornos-cognitivos/aota-2020-cuarta-edicion/17349125

Ares Senra, L., Diaz-Mor Prieto, C., & Huerta Mareca, R. (2014). Terapia Ocupacional en pacientes ingresados en UCI con daño neurologico y estados de minima conciencia. *TOG. REVISTA TERAPIA OCUPACIONAL GALICIA*, *19*. file:///C:/Users/57310/Downloads/Dialnet-TerapiaOcupacionalEnPacientesIngresadosEnUciConDan-4710536%20(2).pdf

Celis, F., Gálvez, C., Moretti, C., Navarrete, E., Rovengo, M., & Torrent, V. (2014). Terapia ocupacional y paciente crítico. *Revista Chilena de Terapia Ocupacional*, *14*(1), 101. https://doi.org/10.5354/0719-5346.2014.32395

Celis-Rodríguez, E., Birchenall, C., de la Cal, M. Á., Castorena Arellano, G., Hernández, A., Ceraso, D., Díaz Cortés, J. C., Dueñas Castell, C., Jimenez, E. J., Meza, J. C., Muñoz Martínez, T., Sosa García, J. O., Pacheco Tovar, C., Pálizas, F., Pardo Oviedo, J. M., Pinilla, D.-I., Raffán-Sanabria, F., Raimondi, N., Righy Shinotsuka, C., … Rubiano, S. (2013). Guía de práctica clínica basada en la evidencia para el manejo de la sedoanalgesia en el paciente adulto críticamente enfermo. *Medicina Intensiva*, *37*(8), 519-574. https://doi.org/10.1016/j.medin.2013.04.001

Costigan, F. A., Duffett, M., Harris, J. E., Baptiste, S., & Kho, M. E. (2019). Occupational Therapy in the ICU: A Scoping Review of 221 Documents. *Critical Care Medicine*, *47*(12), e1014-e1021. https://doi.org/10.1097/CCM.0000000000003999

Hellweg, S. (2012). Effectiveness of Physiotherapy and Occupational Therapy after Traumatic Brain Injury in the Intensive Care Unit. *Critical Care Research and Practice*, *2012*, 1-5. https://doi.org/10.1155/2012/768456

López, B. P. (2010). *Terapia Ocupacional aplicada al Daño Cerebral Adquirido*. Ed. Médica Panamericana.

Martínez-Leiva, J., Parra-Montañez, G., & Segura-Esquivel, J. (2020). Acciones y beneficios que proporciona la intervención del Terapeuta Ocupacional en la Unidad de Cuidados Intensivos de adultos. *Revista Terapéutica*, *14*(1), 11-23. https://doi.org/10.33967/rt.v14i1.94

Mendoza, J. E., Apostolos, G. T., Humphreys, J. D., Hanna-Pladdy, B., & O'Bryant, S. E. (2009). Coin Rotation Task (CRT): A New Test of Motor Dexterity. *Archives of Clinical Neuropsychology*, *24*(3), 287-292. https://doi.org/10.1093/arclin/acp030

Moreno-Chaparro, J., Cubillos-Mesa, C., & Duarte-Torres, S. C. (2017). Terapia ocupacional en unidad de cuidados intensivos. *Revista de la Facultad de Medicina*, *65*(2), 291-296. https://doi.org/10.15446/revfacmed.v65n2.59342

Provancha-Romeo, A. F., Hoffman, A. L., Malcolm, M. P., Coatsworth, J. D., Laxton, L. R., Freeman, K. M., & Schmid, A. A. (2019). Mind-body interventions utilized by an occupational therapist in a medical intensive care unit: An exploratory case study. *Work*, *63*(2), 191-197. https://doi.org/10.3233/WOR-192920

Rapolthy-Beck, A., Fleming, J., & Turpin, M. (2022). Occupational therapy service provision in adult intensive care units in Australia: A survey of workload practices, interventions nd barriers. *Australian Occupational Therapy Journal*, *69*, 316-330.

Rapolthy-Beck, A., Fleming, J., Turpin, M., Sosnowski, K., Dullaway, S., & White, H. (2021). A comparison of standard occupational therapy versus early enhanced occupation-based therapy in a medical/surgical intensive care unit: Study protocol

for a single site feasibility trial (EFFORT-ICU). *Pilot and Feasibility Studies*, *7*(1), 51. https://doi.org/10.1186/s40814-021-00795-2

Sánchez, D. P., & Mora, L. T. O. (s. f.). *EVALUACIÓN DE LA INTEGRIDAD SENSORIAL*.

Sanz, S., De Pobes, A., Bové, M., Tàsies, S., Andrés, B., Noguera, A., Soriano, M., & Roig, M. (2004). Terapia Ocupacional en el estado vegetativo y de mínima conciencia: Estimulación sensorial. *Mapfre Medicina*, *15*, 112-117.

Schweickert, W. D., Pohlman, M. C., Pohlman, A. S., Nigos, C., Pawlik, A. J., Esbrook, C. L., Spears, L., Miller, M., Franczyk, M., Deprizio, D., Schmidt, G. A., Bowman, A., Barr, R., McCallister, K. E., Hall, J. B., & Kress, J. P. (2009). *Early physical and occupational therapy in mechanically ventilated, critically ill patients: A randomised controlled trial*. *373*.

Sheard, K. L., Lape, J. E., & Weissberg, K. (2022). Occupational Therapy-Led Delirium Management in Long-Term Acute Care: A Pilot. *Physical & Occupational Therapy In Geriatrics*, *40*(4), 376-391. https://doi.org/10.1080/02703181.2022.2043983

Silva Barbosa, F. D., & Da Silva Reis, M. C. (2017). O papel da Terapia Ocupacional nas unidades de terapia intensiva—Uma revisão da literatura/ The role of occupational therapy in intensive care units—A literature review. *Revista Interinstitucional Brasileira de Terapia Ocupacional - REVISBRATO*, *1*(2), 221-239. https://doi.org/10.47222/2526-3544.rbto4753

Weinreich, M., Herman, J., Dickason, S., & Mayo, H. (2017). Occupational Therapy in the Intensive Care Unit: A Systematic Review. *Occupational Therapy In Health Care*, *31*(3), 205-213. https://doi.org/10.1080/07380577.2017.1340690

COMPONENTE FISIOTERAPIA

Coordinadoras:

- Catherine Lissell Arévalo Guerrero, email: Catherine.arevalo@fsfb.org.co
- Paula Andrea Barreto Garzón, email: paula.barreto@fsfb.org.co

Justificación

Mientras que, en décadas previas, menos del 50% de pacientes con lesión pulmonar aguda que requerían intubación orotraqueal recibían una valoración de la deglución durante su hospitalización, (19) en la actualidad se ha incorporado cada vez más los grupos de rehabilitación multidisciplinaria. (7,20–22) En un estudio realizado en el transcurso de 5 años en Estados Unidos, que incluyo 264.137 pacientes con necesidad de ventilación mecánica, se encontró que los pacientes reciben terapia física, ocupacional y del lenguaje de manera variable. A pesar de la concientización sobre la movilización de estos pacientes, la rehabilitación no empieza tan temprano cómo es posible. Sólo en el 24% de los pacientes se instauró la terapia física el mismo día de la ventilación mecánica. (23) Hay menos información disponible en la literatura, pero en este estudio, sólo el 12.2% de pacientes bajo ventilación mecánica recibió terapia ocupacional mientras que el 33% recibió orden de terapia de lenguaje. (23)

La terapia de rehabilitación varía significativamente entre regiones geográficas, pero en general, puede involucrar además del equipo médico, profesionales de terapia física, terapia respiratoria, terapia del lenguaje o fonoaudiología, terapia ocupacional y enfermería. En países sin disponibilidad directa de terapias especializadas bien sea por cuestiones de formación o por disponibilidad de recursos, el grupo de enfermería o de terapia física asume el rol de terapia ocupacional, respiratoria y del lenguaje (20–24)

La rehabilitación física es crucial en pacientes que ingresan a la unidad de cuidado intensivo ya que 20-50% de pacientes críticamente enfermos experimentan algún grado de debilidad.

En la actualidad, la movilización realizada en las unidades de cuidado intensivo es aceptada como una herramienta terapéutica con el potencial de prevenir o atenuar deterioro funcional en estos pacientes. Sin embargo, el momento ideal para el inicio de esta estrategia ha sido ampliamente debatido. (3,5)

La movilización temprana se ha propuesto como una política atractiva en este grupo de pacientes por parte de algunos autores. Pues en algunos estudios ha demostrado adecuada tolerancia con menos incidencia de delirium, más días libres de ventilación mecánica y mejores desenlaces funcionales al alta hospitalaria. (2,25–27)

Sin embargo, su beneficio ha sido ampliamente debatido, debido a que algunos estudios no han demostrado mejoría de desenlaces, algunos incluso han reportado aumeno en eventos adversos. Esto se ha atribuido a múltiples irregularidades al momento de comparar los estudios: principalmente, hay una falta de estandarización del concepto de temprano, que en muchos casos ni siquiera es definido. Tampoco hay consenso en la terapia establecida, el comparador o la “terapia usual”, que varía ampliamente dependiendo de las políticas institucionales, los criterios de inclusión y exclusión, ni la frecuencia de las intervenciones y los desenlaces. (3) Otro factor que puede contribuir a la falta de mejoría en los desenlaces es la heterogeneidad de los pacientes, tanto en su línea de base, como en su patología y en la respuesta a las intervenciones propuestas en la unidad. (3)

En un estudio reciente publicado en el New England Journal of Medicine (6) en el que se recogieron 750 pacientes en 49 hospitales de 6 países, y que fueron aleatorizados para movilización temprana o cuidado usual, no hubo diferencia en mortalidad a 180 días ni en estancia hospitalaria ni en desenlaces secundarios como días de ventilación mecánica y días fuera de la UCI. Hubo en cambio un aumento en efectos adversos y efectos adversos serios en el grupo de movilización temprana. Los autores reportan como limitaciones, un mayor nivel de movilización en el grupo de control respecto a estudios previos, barreras para la movilización que pudieron haber limitado el poder estadístico para detectar diferencias entre grupos, y sesgo de vigilancia en el grupo de movilización temprana. (6)

En un metaanálisis y revisión sistemática de la literatura publicada en 2019 que contó con 23 ensayos clínicos controlados aleatorizados, se concluyó que independientemente de las diferentes técnicas y periodos de movilización utilizados, la movilización temprana de los pacientes críticamente enfermos aumento el número de personas capaces de ponerse de pie, y el número de días libres de ventilación mecánica durante la hospitalización, una menor incidencia de debilidad asociada al cuidado intensivo, aumento en la distancia caminada al egreso hospitalario . No hubo en este estudio diferencias significativas en cuanto a mortalidad a 28 días o eventos adversos. (5)

Dentro de los tipos de intervención a considerar incluyen la movilización activa funcional, ergometría cíclica en cama, estimulación muscular eléctrica (con o sin ejercicios activos o pasivos), tablas de inclinación, entre otros. (4) Si bien los ejercicios pasivos no se encuentran formalmente dentro del proceso de rehabilitación, pues no se ha demostrado que aumenten la fuerza o la resistencia muscular. (3) Frecuentemente preceden el inicio de las maniobras activas y son los más utilizados en pacientes bajo efectos de sedación profunda cuya condición clínica lo permite. (7,24,26,27)

La terapia debe ser en la medida de lo posible individualizada para la condición del paciente. Sin embargo, Hickman et al encontraron sólo 5 contraindicaciones para establecer la movilización temprana, que en su estudio fue definido como iniciada en las primeras 24 horas de estancia en UCI: Infarto agudo de miocardio, sangrado activo, aumento de la presión intracraneana y fractura de pelvis inestable. (25) En este grupo de pacientes, los

parámetros hemodinámicos no fueron casi afectados por la terapia, causando su descontinuación en sólo 0.8% de las actividades, principalmente por hipotensión o arritmias. (25)

Una forma para determinar el riesgo/beneficio de aplicar la movilización temprana a pacientes en la unidad de cuidado intensivo fue desarrollada por un grupo multidisciplinario y es ampliamente utilizado en la actualidad. Consiste en la semaforización según el riesgo, de manera que se les otorga categoría verde a pacientes que presentan un bajo riesgo de eventos adversos, es decir, el beneficio sobrepasa las consecuencias potenciales de seguridad. Amarillo representa un riesgo de evento adverso, y en este grupo de pacientes debe haber una discusión de las precauciones a tener en cuenta y las contraindicaciones para la movilización temprana. La categoría roja implica un mayor riesgo de evento adverso, y en estos pacientes no se realiza movilización temprana a menos que sea autorizado por el equipo médico responsable. Si bien esta categoría no constituye una contraindicación, es una advertencia de que en este caso, los riesgos podrían superar los beneficios.(20)

Diferentes guías clínicas apoyan el uso de un bundle para reducir o acortar el tiempo de rehabilitación y la incidencia de delirium. El ABCDEF incluye la evaluación, prevención y manejo de dolor; ensayos de despertar y de retiro de la ventilación mecánica invasiva, la evaluación, prevención y manejo del delirium, la movilización temprana y el ejercicio, y el involucrar y favorecer el ambiente familiar de los pacientes). (21)

Aún en equipos donde se promueve la rehabilitación temprana, los estudios han encontrado múltiples barreras tanto para movilizar a los pacientes en ventilación mecánica, como para mantener la dosis o el tiempo propuesto de intervención. (28)

En la práctica, ofrecer movilización temprana puede ser difícil, ya que requiere tiempo adicional, profesional y material especializado, y un abordaje en equipo coordinado. (26)

Dentro de las barreras para la movilización temprana se han encontrado: Las relacionadas con el paciente, que pueden ser signos y síntomas o condiciones como inestabilidad hemodinámica o respiratoria; las barreras estructurales, como recurso humano y de equipo o técnicos; las barreras relacionadas con la cultura de la UCI, incluyendo hábitos y actitudes arraigadas en cada institución y limitaciones relacionadas con el proceso, falta de coordinación, ausencia de roles y reglas que determinen y distribuyan las tareas y responsabilidades de manera adecuada. (27)

Una de las barreras o de las razones más frecuentes para evitar que se cumplan los protocolos de movilización son los altos niveles de sedación. Los niveles adecuados de reducción y adecuación de la sedo analgesia para permitir la participación del paciente en ejercicios es importante para los desenlaces favorables. (27) Otros factores importantes son el adecuado manejo del dolor y el reconocimiento y manejo temprano del delirium. (27)

El objetivo de recuperación debe establecerse tan temprano como sea posible para cada paciente. Y con él, se deben implementar estrategias para mejorar la implementación de la rehabilitación integral, dentro de las que es importante destacar la identificación de barreras y factores facilitadores; la creación de equipos multidisciplinarios con líderes que permita mejorar la comunicación, la educación y entrenamiento. (3)

El criterio de la seguridad del paciente es una barrera frecuentemente reportada. Con la movilización hay un aumento en el riesgo del retiro de medidas invasivas como sondas, tubos, catéteres y equipos y es una creencia común entre médicos y enfermeras que limita el rol de la fisioterapia y compromete la implementación de protocolos de movilización

temprana. Este riego sin embargo se ha demostrado despreciable cuando el programa se realiza por personal capacitado y calificado para ello. (27)

Para mejorar la preocupación cultural en la UCI, un estudia aleatorizado multicéntrico internacional, implementó el establecimiento de los objetivos de movilización durante la revista médica diaria, con ello lograron alcanzarlos en 89% de los días en el grupo de intervención. En este estudio, se logró estancia en uci más corta y hospitalaria, mejoría en la movilidad funcional al alta hospitalaria. (7)

Alcance

Pacientes seleccionados para el proyecto de investigación: "Asociación entre terapia multimodal temprana y días de ventilación mecánica en la unidad de cuidado intensivos de la Fundación Santa Fe de Bogotá: un ensayo clínico controlado aleatorizado (SECEC-2023-129).

Población objeto

Criterios de inclusión

- Pacientes hospitalizados en la unidad de cuidado intensivo adulto de la Fundación Santa Fe de Bogotá que requieran ventilación mecánica invasiva a través de un tubo endotraqueal durante un periodo mayor de 72 horas y que se espere que continue por al menos 24 horas.
- Índice de Barthel mayor o igual a 70.

Criterios de Exclusión:

- Pacientes que requieran ventilación mecánica invasiva a través de una cánula de traqueostomía o tubo nasotraqueal.
- Pacientes a los que se realizó algún tipo de cirugía de cabeza y cuello.
- Paro cardiaco.
- Quemadura de la vía aérea.
- Quemaduras con una extensión mayor al 50% del área corporal.
- Paciente con enfermedad pulmonar obstructiva crónica.
- Pacientes remitidos de otra institución.
- Enfermedades desmielinizantes o de la placa neuromuscular.
- Pacientes requieran relajación neuromuscular.
- Pacientes con expectativa de vida menor a 180 días
- Pacientes que por criterio médico no se beneficiarían de tratamiento multimodal
- Pacientes que no ingresan a UCI por primera vez
- Pacientes que participan en otros ensayos clínicos de rehabilitación

Metodología

Recursos:
Materiales:

- Guantes – tapabocas
- Bandas elásticas (Therabant)
- Pesas ajustables.
- Caminadores
- Sillas neurológicas
- Aceite
- Paquetes fríos y calientes
- Equipo de Electroterapia y ultrasonido.
- Balón terapéutico de diferente peso
- Cicloergometro
- Bastón
- Odómetro
- Dinamómetro

Intervención

1. Técnicas de reclutamiento de fibras musculares:

Se utilizarán métodos que faciliten la activación y reclutamiento neuromuscular favoreciendo procesos de planeamiento y control motor desarrollando patrones de tipo coordinativos, posturales, promoviendo el máximo de funcionalidad.

Técnica facilitación neuromuscular propioceptiva con patrones bilaterales simétricos y asimétricos para aumento de fuerza de las cuatro extremidades:

a) Iniciación rítmica: utiliza movimientos rítmicos como una alternativa para mejorar el inicio y la velocidad del movimiento de un miembro o del cuerpo, se inicia con un movimiento pasivo en todo el rango de amplitud progresando a un movimiento activo asistido, posterior a ello el fisioterapeuta aplica resistencia al movimiento activo para finalizar con un movimiento realizado totalmente por el paciente. Se utiliza para iniciar un movimiento, mejorar su coordinación, normalizar la velocidad, ayudar al paciente a relajarse y enseñar el movimiento. Está indicada en pacientes que tienen dificultad para iniciar el movimiento, movimientos lentos o rápidos, movimientos sin coordinación y sin ritmo, para regularizar o normalizar el tono muscular y para relajar la musculatura.

b) Combinación de isotónicos: Permite estabilizar un segmento corporal por medio de la combinación de contracciones musculares concéntricas y excéntricas sin llegar a inhibir grupos musculares. Se debe aplicar resistencia al rango de movimiento del paciente, pero al llegar al final del movimiento se demanda una contracción que permita estabilizar la posición, se finaliza con una contracción excéntrica. En esta técnica los objetivos son activar el control del movimiento, coordinación, aumentar amplitud de movimiento activa, fortalecer, entrenamiento funcional en el control excéntrico de movimiento.

c) Inversión de antagonistas: estas a su vez se subdividen en:

• Inversiones dinámicas: se basan en el principio de inducción sucesiva, durante la realización del movimiento se aplica resistencia continua, al aproximarse al final del recorrido articular se demanda un cambio repentino en el movimiento activo evitando

tiempo de reposo o relajación. Los objetivos radican en aumentar el rango de movilidad activa, fuerza y resistencia, desarrollar coordinación, evitar o disminuir fatiga y tono muscular. Técnica indicada en pacientes que cuentan con disminución de rango de movimiento activo, debilidad de músculos agonistas, dificultad para cambiar el sentido al movimiento, fatiga muscular y relajación de músculos hipertónicos.

• Inversiones de estabilización: utiliza contracciones musculares isotónicas contra resistencia y por medio de tracción o aproximación brindar estabilidad articular, en este caso cuando el paciente brinda una respuesta positiva a la resistencia inmediatamente la fuerza de resistencia cambia en sentido contrario. Sus principales objetivos son el conseguir el aumento de estabilidad, equilibrio, fuerza muscular, coordinación entre agonistas y antagonistas. Indicada en pacientes con estabilidad disminuida, debilidad, incapacidad para contraer músculos isométricamente y requiere resistencia en dirección única.

d) Estabilización rítmica: contracciones isométricas contra fuerza de resistencia progresiva sin el propósito de generar movimiento o cambio de posición. Los objetivos de esta técnica están encaminados a aumentar rangos de movilidad activos y pasivos, fuerza, estabilidad y equilibrio y disminuir el dolor. Está indicada en casos de limitación de rango de movimiento, dolor, inestabilidad articular, debilidad en músculos antagonistas y alteraciones de equilibrio. Contraindicada en pacientes que no siguen órdenes, dificultad en comprensión de lenguaje o disfunción cerebral, promueve la estabilización de áreas corporales por medio de contracciones isométricas que demandan sinergia de grupos musculares, con ello la respuesta excitatoria.

e) Estiramiento repetido: que a su vez se divide en:

• Estiramiento repetido desde el inicio del recorrido: activación de los reflejos de estiramiento, al iniciar el patrón de movimiento los músculos se someten a tensión por elongación.

• Estiramiento repetido durante el recorrido: de caso contrario este utiliza la activación de reflejo de estiramiento al estar el músculo sometido a tensión debido a una contracción. Dentro de los objetivos de ambas variantes está el facilitar la iniciación del movimiento, aumentar rango de movimiento activo y fuerza, evitar o disminuir la fatiga y guiar el movimiento en dirección correcta.

f) Contracción – relajación: que a su vez se divide en:

• Tratamiento directo: el movimiento debe ser guiado hasta el final del rango de movimiento activo conservado por el paciente, en donde se utiliza una contracción máxima resistida de los músculos antagonistas seguida de relajación y con ello un aumento de amplitud de movimiento. El objetivo busca aumentar el rango de movimiento pasivo.

• Tratamiento indirecto: la técnica se beneficia de la contracción de músculos agonistas. Está indicado cuando la contracción muscular es dolorosa o débil.

g) Mantener – relajar (hold relax): que a su vez se dividen en:

• Tratamiento directo: se debe colocar la articulación hasta el final del rango de movimiento para aplicar fuerza de resistencia y demandar una contracción isométrica resistida sin intención de movimiento, para posteriormente relajarse de forma gradual. Se utiliza para aumentar rango de movimiento pasivo y disminuir dolor.

• Tratamiento indirecto: al realizar el movimiento durante el recorrido articular se aplica resistencia a músculos distales al área corporal o segmento doloroso.

h) Repetición Proporciona: secuenciación de aprendizaje motor a través de la reiteración de una actividad, por medio de su ejecución sincrónica y continua. Expone la posición final del movimiento y evalúa la capacidad del paciente al mantener una contracción muscular

2. técnica facilitación neuromuscular propioceptiva con patrones bilaterales asimétricos para aumento de fuerza en músculos respiratorios: Ambas extremidades se dirigen a patrones de movimiento opuestos. Por ejemplo, la extremidad derecha se mueve en flexión – aducción, pero la izquierda se mueve en flexión – aducción.

a) Postdescarga es la percepción de aumento de fuerza muscular al finalizar la realización de una contracción muscular sostenida.

b) Sumación temporal se refiere a una serie de estímulos de baja intensidad durante un periodo de tiempo que genera un estímulo con mayor intensidad y con el genera una respuesta de excitación motriz.

c) Sumación espacial refiere que la utilización de estímulos sincronizados de baja intensidad en diversas áreas del cuerpo reforzándose unos con otros, origina una respuesta.

d) La irradiación es la capacidad de transmisión de un estímulo generado en un segmento corporal específico a otro para obtener una respuesta contráctil.

e) La inducción sucesiva se beneficia del aumento de excitabilidad de músculos antagonistas convirtiéndose en el origen de facilitación de músculos agonistas débiles.

f) La inervación recíproca establece que para conseguir una respuesta inhibitoria de músculos antagonistas se deben realizar contracciones de músculos agonistas contra fuerza de resistencia. (

Entrenamiento musculas diafragmático en paciente ventilado por medio de trigger por presión en aplicación con electroestimulación: El trigger del ventilador mecánico, es el mecanismo con el que el ventilador es capaz de detectar el esfuerzo respiratorio del paciente. La sensibilidad puede establecerse por presión o por flujo. En la sensibilidad por presión, el esfuerzo inspiratorio del paciente produce una caída programada, la sensibilidad por presión, se ajustará al 20% de la primera NIF registrada en el paciente, asegurando una frecuencia respiratoria entre 20 y 30 respiraciones/minuto y el volumen tidal de 4 a 6 ml / kg. Realizando 2 sesiones por día, iniciando con 5 min y aumentando 5 min por sesión hasta completar 30 min. Si una paciente toleró 30 min del entrenamiento muscular inspiratorio, La próxima sesión se realiza con el aumento de la sensibilidad de disparo por 10% de la NIF inicial durante 5 min.

3. Aplicación de electro estimulación:

Electroterapia interferencial (IFT): técnica que consiste en la estimulación eléctrica de baja frecuencia para alivio del dolor, estimulación muscular, estimulación muscular

eléctrica EMS: técnica utilizada para genera impulsos que estimulen las neuronas motoras y causen contracción muscular.

Se realizara la aplicación de corriente fijo y móvil a los siguientes grupos musculares

- Músculos que participan en el proceso de inspiración:
- Diafragma esternal costal Lumbar
- Intercostales externos (11 pares)
- Accesorios
- Intercostales íntimos
- Escalenos anterior medio y posterior
- Músculos que participan en el proceso de espiración
- Intercostales internos (11 pares)
- accesorios
- Recto del abdomen
- Oblicuo interno, externo
- Transverso del abdomen

Intervención fisioterapéutica

Acorde con el modelo de atención de Fisioterapia en la UCI del Hospital Universitario de la Fundación Santafé de Bogotá (FSFB) un paciente inicia intervención cuando el médico tratante solicita interconsulta a Medicina Física y Rehabilitación, (Medico Fisiatra) quienes plantean indicaciones a seguir por parte de Fisioterapia. Los Fisioterapeutas realizan la evaluación e inician el plan de tratamiento.

En el marco del proyecto SECEC-2023-129, habrá dos grupos de intervención: terapia multimodal temprana y terapia multimodal tardía. Con respecto a la primera, esta se instaurará en las primeras 24 horas posteriores a la intubación de un paciente en UCI. La segunda se instaurará conforme al protocolo de Intervención por Fisioterapia en paciente en cuidado crítico de la Fundación Santa Fe de Bogotá (Código: AMB-REHF-PT-020). Es decir, cuando el médico tratante solicita interconsulta por Medicina Física y Rehabilitación.

Independientemente del grupo en el cual sea asignado el paciente, se realizarán dos sesiones (mañana y tarde) los siete días de la semana, con una duración por sesión:

Sesiones de 25 minutos:

- Pacientes que por su condición clínica se encuentran con soporte ventilatorio, soporte inotrópico, sedo-analgesia, terapia reemplazo renal, los cuales requieren de supervisión continua y mayores periodos de recuperación entre un ejercicio y otro.

Sesiones de 50 minutos:

- Sesiones en casos donde se requiera involucrar la deambulación de un paciente con ventilación mecánica invasiva o no invasiva (movilización de varios equipos y la coordinación del equipo multidisciplinario)
- Pacientes con compromiso pulmonar que requieren de periodos de descanso largos durante la sesión de la terapia.
- Pacientes que requieren entrenamiento en marcha con o sin ayuda externa.

La intervención fisioterapéutica se llevará a cabo teniendo en cuenta el protocolo de Intervención por Fisioterapia en paciente en cuidado crítico de la Fundación Santa Fe de Bogotá (Código: AMB-REHF-PT-020).

RESPONSABLE, FRECUENCIA Y LUGAR

1. Responsable: Fisioterapeuta
2. Frecuencia: Según orden médica y/o criterio del profesional de Terapia Física
3. Lugar: Unidad de Cuidado Intensivo Adultos (Tercer y cuarto piso)

COMPONENTE FONOAUDIOLOGÍA

Coordinadoras:

- Miguel Leonardo Pulido Bobadilla, email: miguel.pulido@fsfb.org.co
- Diana Marcela Melo Rojas, email: dianammelo1992@gmail.com

Justificación

El rol de la terapia del lenguaje o fonoaudiología cobra cada vez mayor importancia en el cuidado intensivo, pues Similar a la movilización temprana, la terapia de lenguaje debería involucrarse en la valoración temprana de pacientes en UCI, pues el reconocimiento de la disfunción laríngea y los trastornos deglutorios pueden minimizar el riesgo de disfagia orofaríngea y aspiración.

Los terapeutas del lenguaje pueden proveer apoyo para los pacientes críticamente enfermos con condiciones agudas ya sean neurológicas o de otra etiología médica: trauma, hemorragias, tumores, lesiones de columna, condiciones respiratorias, así como pacientes en postoperatorios complejos de intervenciones neurológicas, cardiaca y de cirugía general. Estas pueden afectar habilidades del lenguaje motor, la deglución, la tos y la voz.

El apoyo temprano de la terapia del lenguaje ayuda a facilitar la comunicación exitosa de los pacientes en cuidado intensivo con el equipo médico y con sus familiares, desarrollando programas de intervención especializadas para cada condición y trabajando con los pacientes desde el punto de vista funcional y de su patología. Esto a su vez mejor su bienestar psicosocial, su compromiso con el cuidado del día a día y la toma de decisiones consentidas. Además, las alteraciones en la comunicación se han asociado a delirium, un problema emergente en unidades de cuidado intensivo.

El estudio videoflurosocópico de la deglución (VFS) y la evaluación endoscópica de la deglución (FEES) son necesarios para el diagnóstico certero de un trastorno deglutorio,

pues la aspiración es una complicación devastadora que puede ocurrir de manera silente hasta en un 30-44% de pacientes y no puede ser diagnosticada con evaluaciones a la cabecera del paciente. Dentro de los factores asociados a procesos aspirativos se encuentra la neumonía, sondas de alimentación y traqueostomía, estancias en UCI prolongadas y aumento en la mortalidad hospitalaria.

La FEES a diferencia de VFS es una herramienta portátil, con el beneficio adicional de que provee una vista directa de la dinámica faríngea y laríngea, así como de la anatomía y el manejo de secreciones por parte del paciente, por lo que puede apoyar decisiones clínicas y frecuentemente destaca dificultades que pueden después ser resueltas. El uso de FEES permite hacer recomendaciones farmacológicas para el manejo de secreciones excesivas, así como intervenciones terapéuticas para fortalecer la base de la lengua, la constricción faríngea y el rango de movimiento laríngeo por lo que en el momento y en conjunto con VFS son considerados el Gold-Standard para la detección de trastornos deglutorios.

Sin embargo, el costo, en ocasiones la negativa de los pacientes, las dificultades para el traslado, y el requerimiento de personal y equipos especializados limitan la aplicación de estas herramientas en todos los pacientes posterior a la extubación.

La habilidad de diagnosticar aspiración de manera fácil y acertada podría minimizar las complicaciones de la aspiración, limitando el retardo innecesario del inicio de la vía oral en pacientes de UCI. Por ello se han diseñado múltiples protocolos especializados y herramientas para identificar pacientes con trastorno deglutorio establecido o riesgo de desarrollarlo, con el objetivo de adoptar un abordaje preventivo que permita reducir complicaciones y desenlaces deletéreos.

Dentro de estas, una combinación entre la prueba de deglución de agua y una evaluación de la deglución a la cabecera del paciente es la única estrategia que ha sido validada para la identificación de disfagia post extubación en pacientes sobrevivientes de falla respiratoria aguda. El test de deglución de Gugging es una herramienta fácil de aplicar por diferentes terapeutas o enfermeras, y que permite una evaluación graduada de la capacidad de deglución del paciente, permitiendo recomendaciones nutricionales.

En un estudio reciente el GUSS-ICU se comparó con FEES, demostrando 89-92% de sensibilidad y 67-89% especificidad para detectar pacientes con disfagia, comparable a los resultados obtenidos en pacientes con accidente cerebrovascular. La variabilidad confiabilidad inter observador fue buena. Esta herramienta tiene como ventaja su simplicidad y el uso de varias consistencias de alimentos, lo que le permite proveer recomendaciones dietarías para los pacientes en contexto post extubación.

Además de las maniobras de fortalecimiento de la musculatura orofaríngea con ejercicios, recientemente se ha propuesto la estimulación eléctrica a este nivel. En algunos estudios ha demostrado mejorar la reorganización de la corteza motora relacionada con la deglución, facilitar la activación de vías cortico bulbares y aumentar los niveles salivares de neurotransmisores asociados a la deglución, como la sustancia P. En el estudio PHAST-TRAC se valoró la estimulación eléctrica faríngea en pacientes con accidente cerebrovascular traqueostomizados con disfagia neurogénica, encontrando que el uso de esta estrategia conllevó a un mayor número de pacientes listos para decanulación, así como menor prevalencia de neumonía, sin aumento en las complicaciones.

En la última década se ha destacado el valor y la experticia de los terapeutas que apoyan el cuidado de pacientes en la unidad de cuidado intensivo. Cada profesión relacionada con la terapia ofrece una amplia variedad de experiencia, conocimiento habilidades y experticia que contribuyen de diferentes formas a la rehabilitación temprana, óptima e integral de los pacientes en cuidado intensivo. Los objetivos de rehabilitación deben estar individualizados para cada paciente y la terapia debe formularse según dichos objetivos. Debe hacerse énfasis en un trabajo interdisciplinario colaborativo, entendiendo el rol y responsabilidades de cada profesional.

Alcance

Pacientes seleccionados para el proyecto de investigación: “Asociación entre terapia multimodal temprana y días de ventilación mecánica en la unidad de cuidado intensivos de la Fundación Santa Fe de Bogotá: un ensayo clínico controlado aleatorizado (SECEC-2023-129).

Población objeto

Criterios de inclusión

- Pacientes hospitalizados en la unidad de cuidado intensivo adulto de la Fundación Santa Fe de Bogotá que requieran ventilación mecánica invasiva a través de un tubo endotraqueal durante un periodo mayor de 72 horas y que se espere que continue por al menos 24 horas.
- Índice de Barthel mayor o igual a 70.

Criterios de Exclusión:

- Pacientes que requieran ventilación mecánica invasiva a través de una cánula de traqueostomía o tubo nasotraqueal.
- Pacientes a los que se realizó algún tipo de cirugía de cabeza y cuello.
- Paro cardiaco.
- Quemadura de la vía aérea.
- Quemaduras con una extensión mayor al 50% del área corporal.
- Paciente con enfermedad pulmonar obstructiva crónica.
- Pacientes remitidos de otra institución.
- Enfermedades desmielinizantes o de la placa neuromuscular.
- Pacientes requieran relajación neuromuscular.
- Pacientes con expectativa de vida menor a 180 días
- Pacientes que por criterio médico no se beneficiarían de tratamiento multimodal
- Pacientes que no ingresan a UCI por primera vez
- Pacientes que participan en otros ensayos clínicos de rehabilitación

Metodología

Recurso humano:

Profesionales en Fonoaudiología

Material:

- Computadores
- Historia Clínica
- Mesa
- Pruebas definidas para valoración

Intervención

Terapia fonoaudiológica pasiva/asistida (propioceptiva):

Terapia fonoaudiológica activa/asistida (praxis neuromuscular)

Se implementa la escala de fuerza muscular modificada (Medical Research Council) Se iniciará realizando valoración de cualidades musculares orofaciales. Se valorará músculos buccinadores, maceteros, cadena muscular labial y lingual:

1. Se realiza valoración de fuerza y movilidad cervical.
2. Valoración de posicionamiento de maxilar inferior para verificación de fuerza de cierre y/o permanencia de apertura oral en reposo.
3. Valoración de sensibilidad superficial facial en tercio medio, superior e inferior bilateral.
4. Se realiza palpación de grupos musculares orofaciales para verificar cualidades de tono muscular.
5. Se solicita al paciente la realización de movimientos práxicos simples para valoración de rangos de movilidad, alcance y fuerza.
6. Se valorarán en ejecución contra-gravedad, luego en resistencia y posteriormente en contra-resistencia.
7. De acuerdo con los resultados se realizará:

- Clasificación dentro de la Escala de Fuerza Muscular Medical Research Council / Modificada.

También se tiene en cuenta la tendencia a la fatigabilidad ante la repetición

Flexión, inclinación lateral y rotación del cuello

- Flexión cerval superior: Trabaja los músculos largos de la cabeza, recto anterior de la cabeza, músculos suprahioideos (flexión de la columna cervical superior), músculos infrahioideos (estabiliza el hioides)
- Flexión cervical inferior: Trabaja el largo del cuello, platisma, escaleno anterior, esternocleidomastoideo
- Rotación: Contralateral (todos los escalenos), esternocleidomastoideo, Homolateral: Largo de la cabeza y el cuello, recto anterior de la cabeza
- Inclinación lateral: Largo del cuello, todos los escalenos y el esternocleidomastoideo

Extensión, inclinación lateral, rotación del cuello

- Extensión cervical superior: Iliocostal y longísimo de la cabeza, oblicuo de la cabeza (superior e inferior), recto posterior de la cabeza (mayor y menor), semiespinoso y espinoso de la cabeza, trapecio

- Extensión cervical inferior: Iliocostal del cuello, longísimo y esplenio del cuello, multífidos y rotadores, semiespinoso y esplenio del cuello, trapecio
- Rotación: Contralateral: multífidos y rotadores, semiespinoso de la cabeza, trapecio superior, Homolateral: oblicuo inferior de la cabeza, esplenios del cuello y de la cabeza
- Inclinación lateral: Iliocostal del cuello, intertransversos (cervical), longísimo de la cabeza, oblicuo superior de la cabeza, esplenio del cuello y de la cabeza, trapecio

El movimiento de la mandíbula se asocia con el movimiento de la cabeza sobre el cuello, la apertura de la boca y la flexión cervical superior se refuerzan mutuamente, al igual que el cierre de la boca y la extensión cervical superior
La irradiación desde los patrones de flexión del cuello facilita la flexión del cuello, la elongación del tronco y la inclinación lateral del tronco.

El paciente debe estar sentado, ya que es funcional para el movimiento y la estabilidad del cuello, los músculos flexores en esta posición tienen que ser lo suficientemente fuerte para levantar la cabeza contra la acción de la gravedad:

Intervención fisioterapéutica

Acorde con el modelo de atención de Fonoaudiología en la UCI del Hospital Universitario de la Fundación Santafé de Bogotá (FSFB) un paciente inicia intervención cuando el médico tratante solicita interconsulta a Fonoaudiología. Los Fonoaudiólogos realizan la evaluación e inician el plan de tratamiento.

En el marco del proyecto SECEC-2023-129, habrá dos grupos de intervención: terapia multimodal temprana y terapia multimodal tardía. Con respecto a la primera, esta se instaurará en las primeras 24 horas posteriores a la intubación de un paciente en UCI. La segunda se instaurará conforme al modelo de atención por Fonoaudiología en paciente en cuidado crítico de la Fundación Santa Fe de Bogotá. Es decir, cuando el médico tratante solicita interconsulta por Fonoaudiología.

Independientemente del grupo en el cual sea asignado el paciente, se realizarán 2 sesiones (mañana y tarde) los siete días de la semana, con una duración por sesión de 15 a 30 minutos dependiendo de la escala RASS del paciente.

RESPONSABLE, FRECUENCIA Y LUGAR

1. Responsable: Fonoaudiólogo
2. Frecuencia: Según orden médica y/o criterio del profesional de Fonoaudiología

3. Lugar: Unidad de Cuidado Intensivo Adultos (Tercer y cuarto piso)

FUNDACIÓN SANTA FE DE BOGOTÁ
COMITÉ CORPORATIVO DE ÉTICA
E INVESTIGACIÓN

22 ABR 2024

APROBADO

Firma: Dr. Klaus Mieth A.
Presidente

ANEXO 2 Fundación Santa Fe de Bogotá

Asociación entre terapia multimodal temprana y días de ventilación mecánica en la unidad de cuidado intensivos de la Fundación Santa Fe de Bogotá: un ensayo clínico controlado aleatorizado.

**Título del estudio**	Asociación entre terapia multimodal temprana y días de ventilación mecánica en la unidad de cuidado intensivos de la Fundación Santa Fe de Bogotá: un ensayo clínico controlado aleatorizado.
**Nombre del Investigador Principal**	Jorge Iván Alvarado Sánchez<br>Laura María Castillo Morales
**Teléfono de contacto con el investigador**	6030303 ext. 5889
**Nombre de la Institución de Investigación**	Fundación Santa Fe de Bogotá
**Dirección de la Institución de Investigación**	Calle 119A No.7-49, Cuarto piso torre de expansión UCI adultos
**Versión y fecha del consentimiento**	Versión 2.0 del 05 de abril de 2024
**Número codificado del participante**	

La Fundación Santa Fe de Bogotá (FSFB) y el Departamento de Medicina Crítica y Cuidado Intensivo lo están invitando a participar / están invitando a su familiar a participar como voluntario en un proyecto en el que se compara la terapia multimodal (terapia física, ocupacional, respiratoria y fonoaudiología) temprana frente a la terapia multimodal tardía en pacientes con ventilación mecánica invasiva en la Unidad de Cuidado Intensivo de la FSFB.

Este documento de Consentimiento Informado le proporcionará la información necesaria para ayudarle a decidir a usted y/o su familiar sobre su participación en el estudio. Por favor lea atentamente la información. Si cualquier parte de este documento no le resulta claro o si tiene alguna pregunta o desea solicitar información adicional, no dude en pedirla en cualquier momento a alguno de los miembros del equipo de estudio, quienes se mencionan al final de este documento.

1. **NATURALEZA Y PROPÓSITO DEL ESTUDIO:** Este estudio tiene como objetivo evaluar la diferencia en días de ventilación mecánica invasiva entre la terapia multimodal temprana y la terapia multimodal tardía (manejo estándar) en la

FUNDACIÓN SANTA FE DE BOGOTÁ
COMITÉ CORPORATIVO DE ÉTICA
E INVESTIGACIÓN
22 ABR 2024
APROBADO
Firma: Dr. Klaus Mieth A.
Presidente

unidad de cuidado intensivo adulto de la Fundación Santa Fe de Bogotá. La ventilación mecánica es un tratamiento utilizado cuando una persona tiene dificultades para respirar por sí misma. Se trata de una maquina especializada similar a un "pulmón, pero fuera del cuerpo" que ayuda a una persona a respirar al inflar y desinflar sus pulmones de manera controlada. Este proceso se realiza a través de un tubo colocado en la garganta (también conocido como tubo endotraqueal) o mediante una máscara que se coloca sobre la boca y la nariz.

2. **¿QUIÉN PUEDE PARTICIPAR?:** Para participar usted / su familiar debe cumplir con los siguientes criterios:

    - Personas mayores de 18 años
    - El participante debe estar hospitalizado en la Unidad de Cuidado Intensivo de la Fundación Santa Fe de Bogotá
    - El participante debe requerir ventilación mecánica invasiva por más de 24 horas (un día) posterior al ingreso en la Unidad.
    - El participante debe ser una persona con alta capacidad funcional, la cual será medida mediante una herramienta conocida como índice de Barthel (la cual es una escala que se usa por personal de salud para objetivar que tan funcional es una persona en su día a día y puntua de 0 a 100). Para esta investigación, el índice debe ser mayor a 70 puntos (siendo 100 una independencia total para las tareas de la vida diaria y 0 una dependencia total de la persona a los cuidados de un tercero).

3. **TRATAMIENTOS ALTERNATIVOS VENTAJOSOS PARA EL SUJETO:** El médico tratante determinara si hay otros tratamientos de los que se pueda beneficiar el paciente o si el paciente no requiere todas las intervenciones de la terapia multimodal, sino un subgrupo de ellas. Su participación en este estudio contribuirá al avance del conocimiento médico en este campo.

4. **DURACIÓN ESPERADA DE PARTICIPACIÓN Y NÚMERO DE SUJETOS:** Su participación durará 90 días a partir del momento en que requiere ventilación mecánica invasiva. Comprendemos que probablemente el tiempo de ventilación mecánica no sea de 90 días y que puede que ya se encuentre en casa con su familia, por lo que el seguimiento que está previsto para este estudio será telefónico por lo que no tendrá que trasladarse nuevamente a la institución para ello. El número total de participantes será de 74.

5. **PROCEDIMIENTOS DEL ESTUDIO:** Existen dos grupos en este proyecto: un grupo de terapia multidisciplinaria temprana, la cual se define como el conjunto de maniobras especializadas que realiza el grupo de fisioterapia, fonoaudiología, terapia respiratoria y terapia ocupacional a partir de las primeras 24 horas que se realiza la intubación y se inicia la ventilación mecánica. El segundo grupo corresponde a la terapia multidisciplinar tardía, la cual consiste en las mismas

FUNDACIÓN SANTA FE DE BOGOTÁ
COMITÉ CORPORATIVO DE ÉTICA
E INVESTIGACIÓN

22 ABR 2024

APROBADO

Firma: Dr. Klaus Mieth A.
Presidente

intervenciones que el primer grupo, no obstante, el tiempo de inicio será a partir de las 72 horas que se realiza la intubación y se inicie la ventilación mecánica. Se realizará un monitoreo continuo del estado suyo o de su familiar como se hace de rutina en la UCI. La decisión de retirar la terapia multidisciplinar será tomada por el equipo médico a cargo en caso tal que estas terapias no sean en el mejor interés suyo o de su familiar.

6. **MANEJO DE MUESTRAS BIOLÓGICAS OBTENIDAS:** No se tomarán muestras biológicas específicamente para este proyecto. Todas las muestras biológicas que se tomen corresponderán al tratamiento habitual de los pacientes de la Unidad de Cuidado Intensivo concertado con el grupo médico tratante.

7. **¿QUÉ SE ESPERA DE SU PARTICIPACIÓN?:** Se espera que participe y colabore en el desarrollo del estudio, siguiendo las instrucciones del equipo médico.

8. **¿QUÉ PASARÁ AL FINAL DEL ESTUDIO?:** Al final del estudio, se le proporcionará información detallada sobre los resultados si así lo desea. También tendrá la oportunidad de discutir cualquier pregunta o inquietud que pueda tener. En este informe de consentimiento informado podrá marcar si desea conocer esta información. En caso tal que marque "SI", se le será comunicada a usted o a su familiar por medio telefónico o por correo electrónico una vez finalice el estudio.

9. **POSIBLES EFECTOS ADVERSOS:** Existen posibles riesgos asociados con la participación en el estudio, incluyendo efectos secundarios de las diferentes terapias y posibles incomodidades. Los efectos adversos conocidos son:

   - **Alteración de la presión arterial:** algunos participantes podrían experimentar una disminución o aumento temporal de la presión arterial durante la realización de las terapias, lo que podría causar mareos o desmayos.

   - **Arritmias Cardiacas (cambios en el ritmo normal del corazón):** en raras ocasiones, los participantes podrían experimentar cambios en el ritmo cardíaco, lo que podría causar palpitaciones o sensación de irregularidad en los latidos del corazón.

   - **Desaturación de oxígeno (disminución de la cantidad de oxigeno que se transporta en el cuerpo):** en algunos casos, los participantes podrían experimentar disminución de la cantidad de oxígeno en la sangre, lo que podría resultar en confusión, mareos o sensación de ahogo y fatiga.

   - **Dolor o agitación (hiperactividad corporal o una sensación de desespero):** algunos participantes podrían experimentar dolor o

FUNDACIÓN SANTA FE DE BOGOTÁ
COMITÉ CORPORATIVO DE ETICA
E INVESTIGACIÓN
22 ABR 2024
APROBADO
Firma: Dr. Klaus Mieth A.
Presidente

agitación debido a la intensidad o condición de base del participante el cual puede aumentar al realizar las actividades correspondientes a cada terapia.

- **Remoción de línea invasiva (como venas o arterias canalizadas para colocación de líquidos o medicamentos):** aunque es poco probable, algunas personas podrían removerse accidentalmente líneas invasivas (líquidos endovenosos, nutrición parenteral, bombas de infusión). Esto implicaría que sería necesario volver a establecer estas líneas invasivas.

- **Taquipnea (aumento de la frecuencia en la que se respira):** dado que estas terapias implican actividad física, se espera que haya un aumento de la frecuencia respiratoria. En algunos casos, los participantes podrían experimentar sensación de ahogo o fatiga debido al aumento del esfuerzo físico.

- **Deterioro neurológico:** en casos excepcionales, los participantes podrían experimentar cambios en la función cerebral, lo que podría resultar en confusión, mareos persistentes o dificultades para hablar.

- **Otros efectos secundarios:** además de los mencionados, pueden ocurrir otros efectos secundarios no previstos debido a la complejidad de los tratamientos médicos y la condición de base del paciente. Estos serán monitoreados cuidadosamente y tratados según sea necesario.

10. **RIESGOS Y BENEFICIOS:** Los riesgos incluyen posibles efectos secundarios de los procedimientos médicos y las terapias explicados en el punto 9. Los beneficios incluyen contribuir al avance del conocimiento médico y posiblemente mejorar el tratamiento de pacientes con ventilación mecánica invasiva en el futuro.

11. **INFORMACIÓN NUEVA DEL ESTUDIO:** Su médico del estudio le comunicará oportunamente toda información nueva obtenida durante el estudio que pueda afectar su voluntad de continuar participando. Cuando se le comunique esta información nueva, se le pedirá que firme y feche un nuevo formulario de consentimiento, si acepta continuar en el estudio.

12. **QUÉ MÁS NECESITA SABER ANTES DE DECIDIR PARTICIPAR:** usted recibirá una copia de este formato de Consentimiento Informado, consérvela en un lugar seguro y utilícela como información y referencia durante todo el desarrollo

FUNDACIÓN SANTA FE DE BOGOTÁ
COMITÉ CORPORATIVO DE ÉTICA
E INVESTIGACIÓN

22 ABR 2024

APROBADO

Firma: Dr. Klaus Mieth A.
Presidente

del estudio. Esta investigación se llevará acabo de acuerdo con la resolución 8430 de 1993 y 2378 de 2008 del Ministerio de Salud colombiano. Este documento fue revisado y aprobado por el Comité Corporativo de Ética en Investigación y cumple con todos los requerimientos metodológicos y éticos para ser desarrollado.

Ni usted ni el Departamento de Medicina Crítica y Cuidado Intensivo recibirán compensación económica por participar en este estudio.

13. **PUEDEN EXISTIR RAZONES POR LAS CUALES USTED NO PUEDA PARTICIPAR:** su participación en este estudio es absolutamente voluntaria. No está obligado a participar y puede retirar su participación en cualquier momento sin que esto implique sanciones ni la pérdida de los beneficios a los que tiene derecho. Si decide abandonar el estudio antes de la última visita del estudio, infórmelo al médico del estudio y siga sus instrucciones.

14. **CUBRIMIENTO DE EVENTOS ADVERSOS / COMPENSACIÓN:** dado que la aplicación de la terapia multimodal hace parte de nuestra práctica médica habitual, las complicaciones relacionadas con ellas no requieren una póliza adicional debido a que se asocian directamente con la terapia y la condición médica de base. Al firmar este consentimiento usted no renuncia a ninguno de sus derechos legales que pudiese corresponderle en caso de que se produzca algún daño, y que se acredite que el mismo se haya producido como consecuencia directa de la terapia multimodal y su participación en el estudio. El investigador empeñaraátodos sus esfuerzos para evitar que se produzca algún daño.

15. **QUÉ OCURRIRÁ SI DECIDE NO PARTICIPAR O SI CAMBIA DE IDEA:** La participación en este estudio es totalmente voluntaria, usted no está obligado a participar, puede retirarse en cualquier momento sin justificar su decisión, sin sufrir ninguna sanción o detrimento en la atención por parte de su médico ni de la Institución o puede también ser retirado por su investigador por alguna razón que él le explicará, pero en cualquiera de los dos casos se le realizarán pruebas o procedimientos para terminar su participación de manera ordenada.

16. **CONFIDENCIALIDAD Y PRIVACIDAD DE DATOS:** El investigador asegurará la confidencialidad de su historia clínica, en la cual no se identificará al sujeto, se mantendrá la confidencialidad de la información relacionada con su privacidad, utilizando códigos hasta donde las leyes y regulaciones lo permitan y no serán

FUNDACIÓN SANTA FE DE BOGOTÁ
COMITÉ CORPORATIVO DE ÉTICA
E INVESTIGACIÓN

22 ABR 2024

APROBADO

Firma: Dr. Klaus Mieth A.
Presidente

accesibles públicamente. Los datos obtenidos podrán ser consultados por autoridades sanitarias, autoridades de salud nacional Instituto Nacional de medicamentos y Alimentos- INVIMA- y Comité de Ética en Investigación.

El centro del estudio registrará la información personal básica sobre usted, como su nombre, información de contacto, sexo, estatura, peso y etnia, así como también información sobre sus antecedentes médicos y los datos clínicos recopilados acerca de su participación en el estudio. Todo el personal con acceso a sus registros está obligado a respetar su confidencialidad en todo momento.

Para garantizar su privacidad, no se incluirá su nombre ni ninguna otra información que lo identifique directamente en los registros entregados para los fines de la investigación. Los únicos que podrán vincular este código con su nombre son el médico del estudio y el personal autorizado, quienes podrán hacerlo mediante una lista que se conservará de forma segura en el centro de investigación.

Sus datos codificados serán analizados por los investigadores del Departamento de Medicina Crítica y Cuidado Intensivo para actividades relacionadas con el estudio. Los datos se transferirán a una base de datos informática y se procesarán para permitir que los resultados de este estudio se analicen, informen y publiquen. Al publicar los resultados del estudio, se seguirá manteniendo la confidencialidad de su identidad. En virtud de la Ley de protección de datos en Colombia 1581 de 2012, el Centro de investigación sera responsable de garantizar la protección de su información personal. En el caso de transferir sus datos a otros países en los que las leyes no proporcionen el mismo grado de garantías y derechos en materia de protección de datos que las leyes de Colombia, los datos serán anonimizados antes de la transferencia.

Usted tiene derecho a revisar la información personal, a solicitar cambios. Si decide retirarse del estudio, los datos recopilados hasta ese momento podrán seguir procesándose, junto con otros datos recopilados como parte del estudio.

17. QUIÉNES PUEDEN CONTESTAR SUS PREGUNTAS:

En caso de presentar dudas respecto al proyecto, se puede contactar al Departamento de Medicina Crítica y Cuidado Intensivo de la Fundación Santa Fe de Bogotá:

Investigadores principales: Dr. Jorge Iván Alvarado Sánchez, Dra. Laura María Castillo
Telefono: (601) 6030303 Ext. 5889

FUNDACIÓN SANTA FE DE BOGOTÁ
COMITÉ CORPORATIVO DE ÉTICA
E INVESTIGACIÓN

22 ABR 2024

APROBADO

Firma: Dr. Klaus Mieth A.
Presidente

Dirección: Carrera 7 # 117 – 15, Cuarto piso torre de expansión Unidad de Cuidados Intensivos – Adultos)

El Comité Corporativo de Ética en Investigación de la Fundación Santa Fe de Bogotá han revisado y aprobado este proyecto.
Si usted tiene alguna duda o si cree que sus derechos han sido vulnerados, puede comunicarse con el Comité Corporativo de Ética en Investigación de la Fundación Santa Fe de Bogotá:

Nombre del presidente: Dr. Klaus Willy Mieth Alviar
Telefono: 6030303 Ext 5402
Correo: comiteinvestigativo@fsfb.org.co
Dirección: Calle 119ª # 7 – 49

18. **PUBLICACIÓN DE RESULTADOS:** Los resultados del estudio, ya sean positivos, negativos o inconclusos, serán publicados por el Departamento de Medicina Crítica y Cuidado Intensivo de la Fundación Santa Fe de Bogotá de acuerdo con las normativas éticas y legales.

19. **DECLARACIÓN DE CONSENTIMIENTO INFORMADO:**

Yo: ________________________________ con tipo de documento: cédula de ciudadanía ( ), cédula de extranjería ( ), pasaporte ( ), No. ______________ en calidad de paciente ( ) o representante legal ( ) de:______________________ con tipo de documento: cédula de ciudadanía ( ), cédula de extranjería ( ), pasaporte ( ) No.____________ declaró que, al firmar este consentimiento informado, certifico todos los siguientes puntos:

- He leído (o me han leído) este formulario de consentimiento informado en su totalidad y he recibido explicaciones sobre lo que me van a hacer y lo que se me pide que haga. He tenido la oportunidad de hacer preguntas y entiendo que puedo hacer otras preguntas sobre este estudio en cualquier momento.

- He recibido una copia de este formulario de Informe de Consentimiento que puedo guardar como referencia.

- Acepto que mi información personal confidencial esté disponible para que la revisen: el Grupo de Investigación del Departamento de Medicina Crítica y Cuidado Intensivo o cualquier autoridad de salud, institución o entidad

FUNDACION SANTA FE DE BOGOTÁ
COMITÉ CORPORATIVO DE ÉTICA
E INVESTIGACIÓN

22 ABR 2024

APROBADO

Firma: Dr. Klaus Mieth A.
Presidente

gubernamental asignada a esta tarea en este país o en otro país donde la terapia multimodal temprana se esté considerando para su aprobación o, si corresponde, por el Comité de Revisión Institucional o el Comité de Ética.

- Autorizo que el investigador tenga acceso a los registros médicos del hospital en cualquier momento durante el periodo del estudio.
- Autorizo al investigador a procesar mis datos del estudio y a transferirlos si es necesario.
- Entiendo que todos los datos personales serán codificados y/o anonimizadas.
- Entiendo que tengo la libertad de retirarme del estudio en cualquier momento, sin justificar mi decisión y sin afectar la atención médica que reciba, o que también el investigador puede retirarme por alguna razón en protección a mi seguridad
- Entiendo que los resultados cualesquiera que sean serán publicados por el Departamento de Medicina Crítica y Cuidado Intensivo de la Fundación Santa Fe de Bogotá.
- Comprendo que se me informará sobre cualquier información nueva que pudiera afectar mi voluntad de seguir participando en este estudio.
- Acepto voluntariamente participar en este estudio.
- Deseo que se me comuniquen mis resultados / los resultados de mi familiar

  SI __ NO __

  Telefono / celular: ____

  Correo electrónico: ____

Nombres y apellidos del participante:_________________________

Documento de identificación:____________________________

Firma del participante:_________________

Fecha: ____/____/____, Hora: ____:____

Fundación Santa Fe de Bogotá

FUNDACIÓN SANTA FE DE BOGOTÁ
COMITÉ CORPORATIVO DE ÉTICA
E INVESTIGACIÓN

22 ABR 2024

APROBADO

Firma: Dr. Klaus Mieth A.
Presidente

Dirección de residencia:______________________________

Nombre del representante: ____

Documento identificación: ____

Firma del representante:______________________

Fecha: ____/____/____, Hora: ____:____

Dirección: ____________________________

Parentesco: ________________

Firma:________________________________

Fecha: ____ /____ /____ Hora: __:__

Nombre del testigo No. 1: ____

Documento identificación:______________________________

Dirección: ____

Parentesco: ____

Firma:_________________________

Fecha: __ /__ /__ Hora: _:__

Nombre del testigo No. 2: ____

Documento identificación: ____

Dirección: ____

Relación con el paciente: ____

Firma: _________________________

Fundación Santa Fe de Bogotá

FUNDACIÓN SANTA FE DE BOGOTÁ
COMITÉ CORPORATIVO DE ÉTICA
E INVESTIGACIÓN

22 ABR 2024

APROBADO

Firma: Dr. Klaus Mieth A.
Presidente

Fecha: ____ /____ /____ Hora: __:__

Por la presente certifico que he informado detalladamente a esta/s persona/s sobre el proyecto.

Si alguna información adicional surge durante el proyecto, de modo que pudiera afectar al consentimiento dado por el representante, le informaré de manera oportuna.

Nombre del investigador:________________________________

Documento identificación: ______________________________

Firma del investigador:____________________

Fecha: ____/____ /____ Hora: __:__

Firma recibido copia del consentimiento informado

____

Nombre: Fecha: __/__ /__ Hora: __:__

Anexo 3

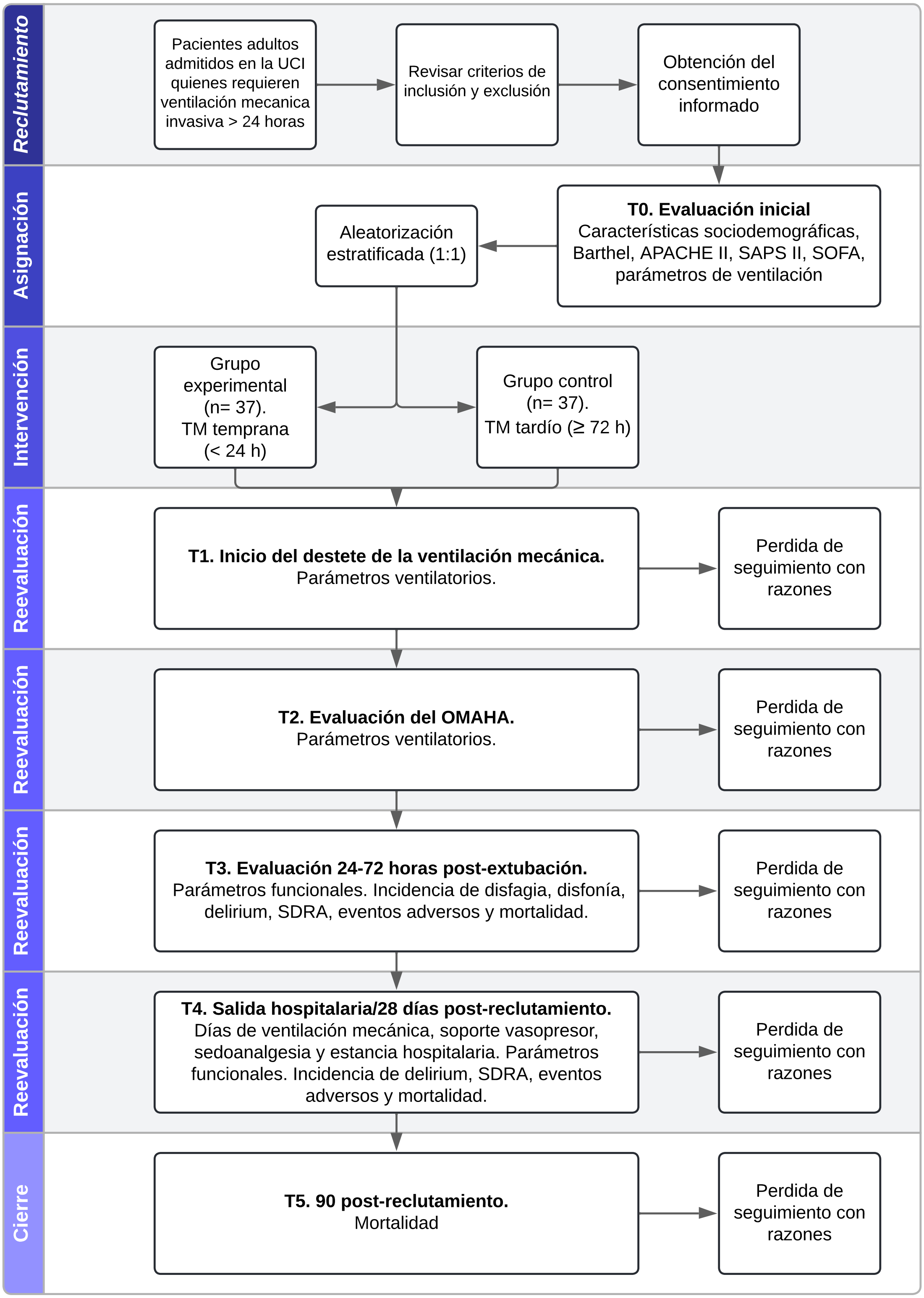

Reclutamiento
Pacientes adultos admitidos en la UCI quienes requieren ventilación mecanica invasiva > 24 horas
Revisar criterios de inclusión y exclusión
Obtención del consentimiento informado
Asignación
T0. Evaluación inicial
Características sociodemográficas, Barthel, APACHE II, SAPS II, SOFA, parámetros de ventilación
Aleatorización estratificada (1:1)
Intervención
Grupo experimental (n= 37). TM temprana (< 24 h)
Grupo control (n= 37). TM tardío (≥ 72 h)
Reevaluación
T1. Inicio del destete de la ventilación mecánica.
Parámetros ventilatorios.
Perdida de seguimiento con razones
Reevaluación
T2. Evaluación del OMAHA.
Parámetros ventilatorios.
Perdida de seguimiento con razones
Reevaluación
T3. Evaluación 24-72 horas post-extubación.
Parámetros funcionales. Incidencia de disfagia, disfonía, delirium, SDRA, eventos adversos y mortalidad.
Perdida de seguimiento con razones
Reevaluación
T4. Salida hospitalaria/28 días post-reclutamiento.
Días de ventilación mecánica, soporte vasopresor, sedoanalgesia y estancia hospitalaria. Parámetros funcionales. Incidencia de delirium, SDRA, eventos adversos y mortalidad.
Perdida de seguimiento con razones
Cierre
T5. 90 post-reclutamiento.
Mortalidad
Perdida de seguimiento con razones

ANEXO 4

Tiempo de medidas de variables

Inicio del estudio	Inicio del destete	Finalización del destete	Final de observación
• Edad<br>• Peso<br>• Sexo<br>• Etnia<br>• Índice de Barthel<br>• Índice de Masa Corporal<br>• Diagnóstico<br>• Grupo al que pertenecen (Temprana vs tardía)<br>• Diagnóstico<br>• Unidad de internado<br>• Puntuación SOFA<br>• Puntuación APACHE II<br>• Puntuación SAPS II<br>• Volumen corriente<br>• Frecuencia respiratoria<br>• PEEP<br>• Presión meseta<br>• Distensibilidad pulmonar estática<br>• Presión de conducción<br>• Resistencia de la vía aérea<br>• Índice PaO2/FIO2	• Delta POCC<br>• Presión muscular<br>• P0.1 (Presión en 0.1 segundos)<br>• Cantidad de secreciones por tubo orotraqueal<br>• Índice de asincronías<br>• NIF<br>• Porcentaje de fuga (% fuga)<br>• Pico flujo de tos<br>• Medición de excursión diafragmática<br>• Medición de porcentaje de grosor diafragmático	• Delta POCC<br>• Presión muscular<br>• P0.1<br>• Cantidad de secreciones por tubo orotraqueal<br>• Índice de asincronías<br>• NIF<br>• Porcentaje de fuga (% fuga)<br>• Pico flujo de tos<br>• Medición de excursión diafragmática<br>• Medición de porcentaje de grosor diafragmático	• Días de ventilación mecánica<br>• Días libres de ventilación mecánica<br>• Días de delirium<br>• Días de hospitalización con delirium<br>• Días de sedación<br>• Días libres de sedación<br>• Mortalidad<br>• Índice de Barthel al egreso<br>• Días de estancia hospitalaria<br>• Tiempo de estancia en UCI<br>• Mortalidad<br>• Tiempo desde la intubación hasta el inicio de la terapia<br>• Estado de independencia funcional medido por ADLs al egreso hospitalario

			• Estado de independencia funcional medido por ADLs al egreso de la UCI<br>• Fuerza muscular medido con la escala Medical Research Council (MRC)<br>• Disfagia a las 72 horas<br>• Inicio de vía oral<br>• Presencia de neumonía asociada al cuidado de la salud<br>• Movilidad máxima medida por JH-HLM<br>• Fuerza prensil<br>• Falla de extubación<br>• Necesidad de ventilación mecánica no invasiva<br>• Ventilación mecánica prolongada<br>• Traqueostomía<br>• Síndrome de dificultad respiratoria del adulto<br>• Disfonía a las 72 horas<br>• OMAHA al momento de la extubación

ANEXO 5

Formato de recolección de datos

Datos al ingreso:

Datos del Paciente:

- Nombre del Paciente: ____________________
- Código: ______________ (1,2,3...)
- Número de Historia Clínica: ________________
- Sexo _______________
- Etnia ____________ (Afrocolombiano, ROM, Indígena, Raizal, Ninguna)
- Índice de Barthel _____________
- Índice de Masa Corporal __________________
- Diagnóstico ____________ Código diagnostico CI10.
- Grupo al que pertenecen ______________ (Temprana vs tardía)
- Unidad de internado___________ (1 = Quirúrgica, 2 = Neurológica, 3 = Séptica y Respiratoria, 4 = Cardiovascular, 5 = Quemados)
- Puntuación SOFA ___________
- Puntuación APACHE II ____________
- Puntuación SAPS II _______________
- Fecha de Ingreso: ________________
- Fecha de Egreso: ________________
- Tiempo desde la Intubación hasta el Inicio de la Terapia: ________________

Parámetros Respiratorios:

Variables al momento de la intubación, estos datos serán facilitados por terapia respiratoria.

- Volumen Corriente: ________________
- Frecuencia Respiratoria: ________________
- PEEP (Presión Positiva al Final de la Espiración): ________________
- Presión Meseta: ________________
- Distensibilidad Pulmonar Estática: ________________
- Presión de Conducción: ________________
- Resistencia de la Vía Aérea: ________________
- Índice PaO2/FIO2: ________________

Durante observación:

Para registrar de forma adecuada estos datos se debe pasar todos los días a valorar el paciente.

- Días de Ventilación Mecánica: _______________________________(fecha de inicio-fecha de finalización)

- Días Libres de Ventilación Mecánica: ________________ (calculado el final de la observación)
- Días de Delirium: ____(fecha de inicio- fecha de finalización)
- Días de Hospitalización con Delirium: _______(calculado el final de la observación)
- Días de Sedación: ____________ (fecha de inicio- fecha de finalización)
- Días Libres de Sedación: _______________(calculado el final de la observación)
- Ventilación Mecánica Prolongada: _______________(Si/No)
- Traqueostomía: ______ (Si/No).
- Síndrome de Dificultad Respiratoria del Adulto: _____ (Si/No)
- Presencia de Neumonía Asociada al Cuidado de la Salud: ________ (Si/No)
- Necesidad de diálisis: ________ (Si/No)
- Soporte con noradrenalina________ (Si/No)
- Soporte con vasopresina ________ (Si/No)
- Soporte con inotrópico ________ (Si/No)
- Dosis máxima de noradrenalina_______(mcg/kg/min)
- Dosis máxima de vasopresina ______(UI/min)

Datos inicio de destete.

El inicio del destete será determinado por terapeuta.

- Delta POCC ____ (cmH20)
- Presión muscular _____(cmH20)
- P0.1 (Presión en 0.1 segundos) ____(cmH20)
- Cantidad de secreciones por tubo orotraqueal____(Si/No)
- Índice de asincronías___(%)
- NIF ____(cmH20)
- Porcentaje de fuga ____ (% fuga)
- Pico flujo de tos ___(L/min)
- Medición de excursión diafragmática____ (cm)
- Medición de porcentaje de grosor diafragmático ____(cm)

Datos previos a extubación:

- Delta POCC ____ (cmH20)
- Presión muscular _____(cmH20)
- P0.1 (Presión en 0.1 segundos) ____(cmH20)
- Cantidad de secreciones por tubo orotraqueal____(Si/No)
- Índice de asincronías___(%)
- NIF ____(cmH20)
- Porcentaje de fuga ____ (% fuga)
- Pico flujo de tos ___(L/min)

- Medición de excursión diafragmática____ (cm)
- Medición de porcentaje de grosor diafragmático ____(cm)
- OMAHA al Momento de la Extubación: _______________(+/-)

Posterior a extubación:

- Disfonía a las 72 Horas: _______________(Si/No)
- Movilidad Máxima Medida por JH-HLM a las 24 horas: _______________
- Fuerza Prensil a las 24 horas : _______________
- Necesidad de Ventilación Mecánica No Invasiva a las 48 horas: __________(Si/No)
- Disfagia a las 72 Horas: _______________(Si/No)
- Inicio de Vía Oral: _______________ (1: 12-24 horas, 2> 24 horas).
- Falla de Extubación a las 48 horas: _______________(Si/No)
- Fuerza Muscular Medido con la Escala MRC a las 24 horas: _______________

Final de observación:

Funcionalidad y Estancia:

- Índice de Barthel al Egreso hospitalario: ________________
- Función cognitiva medido por MOCA: ________
- Test rápido de sensibilidad y destreza: _______
- Días de Estancia Hospitalaria: __________ (día en el que el intensivista informa traslado a piso).
- Tiempo de Estancia en UCI: _____________________(días).
- Mortalidad: _________________________(si/no)
- Estado de Independencia Funcional Medido por ADLs al Egreso Hospitalario: _______________
- Estado de Independencia Funcional Medido por ADLs al Egreso de la UCI: _______________

Variables de seguridad:

- Desenlaces:
  - Número de eventos adversos.
    - Presión arterial alterada________ (Si/No)(disminución de PAM > 15%)
    - Arritmia cardiaca ________ (Si/No)
    - Desaturación de oxígeno________ (Si/No)(disminución menor al 80%)
    - Dolor o agitación ________ (Si/No)
    - Remoción de línea invasiva ________ (Si/No)

- Gastrointestinal (nauseas, vomito o diarrea) ________ (Si/No)
- Taquipnea________ (Si/No)
- Estado neurológico alterado________ (Si/No)

- Número de eventos adversos serios_______
- Extubación no programada ________ (Si/No)
- Neumonía asociada al cuidado de la salud ________ (Si/No)
- Broncoaspiración________ (Si/No)
