# Supplementary material for: Efficacy and safety of the early implementation of a multimodal rehabilitation program in mechanically ventilated patients: A randomized clinical trial protocol
Source: PLoS One. 2025 May 19;20(5):e0324335. doi: 10.1371/journal.pone.0324335 (PMC12088510; doi:10.1371/journal.pone.0324335)
Supplement: S2 File — (PDF) [file pone.0324335.s002.pdf]

Association between Early Multimodal Therapy and Days of Mechanical Ventilation in the Intensive Care Unit of Fundación Santa Fe de Bogotá: A Randomized Controlled Clinical Trial

- **Principal Investigators:**

  Jorge Iván Alvarado Sánchez
  Médico Anestesiólogo
  Departamento Medicina Crítica y Cuidado Intensivo
  Fundación Santa Fe de Bogotá
  E-mail: Jorge.alvarado@fsfb.org.co

- **Laura María Castillo Morales**
  Departamento Medicina Crítica y Cuidado Intensivo
  Fundación Santa Fe de Bogotá
  E-mail: laura.castillo@fsfb.org.co

Co-investigadors:

- **Catherine Lissell Arévalo Guerrero**
  Instituto de Medicina del Ejercicio y rehabilitación
  Fundación Santa Fe de Bogotá
  E-mail: Catherine.arevalo@fsfb.org.co

- **Miguel Leonardo Pulido Bobadilla**
  Instituto de Medicina del Ejercicio y rehabilitación
  Fundación Santa Fe de Bogotá
  E-mail: miguel.pulido@fsfb.org.co

- **Diana Marcela Melo Rojas**
  Instituto de Medicina del Ejercicio y rehabilitación
  Fundación Santa Fe de Bogotá
  E-mail**:** dianammelo1992@gmail.com

- **Ana Gabriela López Rubio**
  Neumología
  Fundación Santa Fe de Bogotá
  E-mail: aglopez904@gmail.com

- **Diana Carolina Ortiz Moreno**
  Neumología

Fundación Santa Fe de Bogotá
E-mail: carolinao.21@hotmail.com

- **Paula Andrea Barreto Garzón**
  Instituto de Medicina del Ejercicio y rehabilitación
  Fundación Santa Fe de Bogotá
  E-mail: paula.barreto@fsfb.org.co

- **Marisol Murillo**
  Instituto de Medicina física y rehabilitación
  Fundación Santa Fe de Bogotá
  E-mail: marisol.murillo@fsfb.org.co

- **Sara Stefania Martínez**
  Instituto de Medicina física y rehabilitación
  Fundación Santa Fe de Bogotá
  E-mail: sahara.martinez@fsfb.org.co

Research assistants:

- **María Valentina Stozitzky Ríos**
  Magister en epidemiología
  Departamento Medicina Crítica y Cuidado Intensivo
  Fundación Santa Fe de Bogotá
  E-mail: valentinastoziztky@gmail.com

- **Andrea Valentina Montañez Nariño**
  Médico general
  Departamento de Medicina Crítica y Cuidado intensivo
  Fundación Santa Fe de Bogotá
  E-mail: Montanez.andreav@gmail.com

- **Andrés Felipe Mora Salamanca**
  Magister en Epidemiología
  Departamento Medicina Crítica y Cuidado Intensivo
  Fundación Santa Fe de Bogotá
  E-mail: af.mora1963@uniandes.edu.co

Content

1. Summary 4

2. Problem Statement and Justification 8
3. Research question 9
4. Theoretical Framework and State-of-the-Art 12
5. Objectives 19
6. Methodology 20
6.1. Study design 20
6.2. Population 21
6.3. Sample size 22
6.4. Sampling and Randomization 24
6.5. Intervention 24
6.6. Outcomes 28
6.7. Measurements 29
6.8 Loses and Handling of Missing data 32
6.9. Protocol Adherence Criteria 33
6.10. Follow-up and Safety Procedures 34
6.11. Data Collection 34
6.12. Data quality: error and bias 50
6.13. Statistical Analysis Plan 51
7. Ethical Consideration 52
8. Scope 54
9. Researchers´ background 55
10. Activity Schedule 56
11. Budget and Funding 56
12. References 57

1. Summary

**QUALIFICATION**	Association between early multimodal therapy and days of mechanical ventilation in the intensive care unit of the Santa Fe Foundation in Bogotá: a randomized controlled clinical trial.
**SPONSOR**	No
**FINANCING ORGANIZATION**	No. The study does not have external or internal financing.
**RESEARCH QUESTION**	Does the use of early multimodal therapy reduce days of invasive mechanical ventilation in adult patients compared to late multimodal therapy?
**GENERAL OBJECTIVE**	To evaluate the difference in days of invasive mechanical ventilation between early multimodal therapy and late multimodal therapy in the adult intensive care unit of the Santa Fe Foundation in Bogotá.
**SPECIFIC OBJECTIVES**	To describe the sociodemographic and clinical characteristics of patients with invasive mechanical ventilation in the Adult Intensive Care Unit of the Santa Fe Foundation in Bogotá.<br><br>To evaluate the effectiveness of early multimodal therapy versus late multimodal therapy in terms of ventilator-free days and other secondary outcomes.<br><br>Establish the sociodemographic and clinical factors that are associated with the type of therapy (early or late) and with the primary outcome (days free of mechanical ventilation) and secondary outcomes.<br><br>To evaluate the safety of early multimodal therapy versus late multimodal therapy in terms of adverse events, serious adverse events, episodes of aspiration, and healthcare-associated pneumonia.<br><br>To evaluate the impact of the different previously defined subgroups, such as the type of respiratory failure (hypoxemic or

	hypercapnic) and the type of patients (surgical, respiratory, burned, cardiovascular), on the results of the main outcome.
**NUMBER OF SUBJECTS**	37 for each group and 74 in total.
**SUBJECT SELECTION CRITERIA**	All adult patients admitted to the intensive care unit after approval by the ethics committee who enter or require invasive mechanical ventilation.
**DURATION OF THE SUBJECT'S PARTICIPATION AND DURATION OF THE STUDY**	One year.
**STATISTICS, PRIMARY PLAN OF ANALYSIS**	A Shapiro-Wilk test will be performed to verify the distribution of the quantitative variables, considering the number of patients included. Data will be presented as means or medians, accompanied by their standard distribution or interquartile range, as appropriate. Categorical variables will be described as absolute and relative frequencies.<br><br>To compare continuous variables between the two groups (early vs. late therapy), a T-test or the Mann-Whitney U test will be used, depending on the distribution of the data. For categorical variables, a $\chi 2$ test or Fisher's exact test will be used, as appropriate.<br><br>The primary objective of the study is to evaluate the association between the use of early multimodal therapy and days of invasive mechanical ventilation. To answer this question, we will perform a survival analysis and Cox regression. A risk variable will be created by combining the time of invasive mechanical

	ventilation and extubation (yes/no). Then, a univariate Cox regression analysis will be performed with the risk variable as the dependent variable and the intervention group (early vs late) as the independent variable. Likewise, a survival curve will be generated and the differences between the two groups will be analyzed using a log- rank test.<br><br>To evaluate factors related to mechanical ventilation time, univariable Cox regression models will be performed for all variables included in the study. To determine whether continuous variables can be evaluated continuously or as categorical variables, the linearity assumption will be verified through the Martingale residual plot. If linearity is not met, the variable will be stratified, and cut-off points will be defined according to the appearance of the graph. Subsequently, those variables with a p-value of statistical significance (less than 0.25) and those clinically important variables (age, sex, delirium, etc.) will be included in a multivariable Cox regression model. The best model will be selected through a Backward process Stepwise based on Akaike (AIC) and Bayesian (BIC) information criteria.<br><br>Variables that could act as confounding factors will be evaluated using a second multiple-regression model. In this model, the potentially confounding variable will be removed, and the resulting slope or estimator (adjusted estimate) will be calculated. If there is a change greater than 15%-20%, the confounding variable will be included in the final model. Otherwise, the most parsimonious model will be chosen.<br><br>To evaluate the interaction between two variables, another regression model will be carried out that will include a new variable that results from multiplying the two variables that

	potentially interact. If the regression coefficient of this new variable has a significance level of less than 0.05, it is concluded that there is an interaction and said variable will be included in the model. Variables with interaction potential will be identified by assessing confounding factors. The investigators will also evaluate clinical variables that may be considered as interaction or confounding variables through the strategy described above.<br><br>Compliance with the assumption of risk proportionality will be evaluated using Schoenfeld residuals and influential values will be analyzed using graphs of residuals versus observations. Multicollinearity will be evaluated through matrix curves.<br><br>We will use logistic regression models to assess the relationship between our dichotomous variables, such as failed extubation, post- extubation NIV, prolonged mechanical ventilation, tracheostomy and dysphonia, and the other variables studied. The assumptions of a logistic regression model, such as linearity in the log of probabilities, independence of errors, homogeneity of variance, absence of multicollinearity, absence of outliers, and proportionality of effects, will be evaluated through residual analysis and graphical diagnostics, such as Pearson residual analysis or deviance residual analysis.<br><br>A multiplicity adjustment strategy will be used in statistical tests, such as the Bonferroni method, Holm-Bonferroni, or other methods that control the false discovery rate (FDR), to maintain the overall type 1 error rate at an acceptable level.<br><br>We will carry out a detailed exploration through a sensitivity analysis of possible interactions between the identified subgroups, according to the RASS scale, according to adherence

	to the protocol and other relevant variables, to deepen the understanding of any joint influence that may affect the results. If statistically significant differences are not identified through the regression analyses, a post-hoc analysis will be carried out to evaluate whether the size of the recruited sample was adequate to detect possible differences between the established subgroups. This additional analysis will allow the interpretation of the results to be adjusted and will provide valuable information on the validity of the conclusions obtained.<br><br>Finally, an independent data monitoring committee will be established to conduct interim sample size reviews, allowing for adjustments based on variations in event rates observed during the study, thus ensuring adequate statistical power throughout the study.

2. Problem Statement and Justification

Patients under mechanical ventilation (MV) in the intensive care unit (ICU) exhibit high hospital mortality rates, ranging from 23% to 51%. This mortality is linked to the success of weaning and the incidence of nosocomial infections. Prolonged MV can increase complications in critically ill patients, extending immobility and raising the risk of ventilator-associated pneumonia, further restricting patients. It is argued that early mobility improves MV-related outcomes, such as ventilator duration and ventilator-free days, breaking the vicious cycle of prolonged MV and immobilization. Additionally, early rehabilitation is associated with reduced morbidity and mortality, as well as complications of the disease, ICU length of stay, hospital stay, and rehospitalization rate. Therefore, weaning from MV through early rehabilitation is crucial to improving the prognosis of ICU patients.

Despite the growing evidence on the utility of early rehabilitation for critically ill patients, programs and interest in outcomes vary among studies. Given this diversity, healthcare professionals must assess whether different programs have a similar therapeutic effect and ensure that the prescribed rehabilitation provides benefits consistent with previous literature. Besides the heterogeneity among programs, different countries and institutions face barriers to implementation, such as a shortage of trained rehabilitation personnel and a cultural gap in the ICU. In cases of personnel

shortages and settings with conservative ICU ethics, early rehabilitation is adapted to a sustainable level, including modified programs or less frequent visits.

Regarding post-intensive care syndrome (PICS), it was established as a syndrome encompassing new or worsened limitations in physical, cognitive, or mental health status that persist beyond acute hospitalization. The goal is to initiate improvements for ICU survivors and their families throughout the continuum of care. Since the creation of PICS in 2010, observational studies have evaluated factors associated with this syndrome; however, few intervention studies have addressed its prevention. Physical therapy with early rehabilitation is considered integral to the multidisciplinary management of ICU patients. Although exercise has been shown to improve strength and function in other populations, reducing inflammation and affecting oxidative stress, a systematic review on the effectiveness of early rehabilitation to prevent PICS in ICU patients has not yet been conducted. The objective of this systematic review is to evaluate the effectiveness of early rehabilitative interventions in preventing PICS in ICU patients.

To our current knowledge, no studies have evaluated the impact and safety of early (within 24 hours) physical therapy, respiratory therapy, speech therapy, and occupational therapy (multimodal therapy). Therefore, our objective is to assess whether multimodal therapy can reduce the days of invasive mechanical ventilation in critically ill patients. This question is relevant given the growing interest in rehabilitation and improving outcomes in these patients. By addressing this question, we hope to contribute to the knowledge of best practices for the care of critically ill patients and improve their long-term outcomes.

3. Research question

Does the use of early multimodal therapy reduce the days of invasive mechanical ventilation in adult patients compared to late multimodal therapy?

PICOT strategy

Population	Adults (≥ 18 years) hospitalized in the adult intensive care unit of the Santa Fe Foundation in Bogotá who require invasive mechanical ventilation through an endotracheal tube for a period greater than 24 hours
Intervention	Early multimodal therapy (<24 hours from intubation) is defined as the set of therapeutic maneuvers performed by the physiotherapy, speech therapy, respiratory therapy, and occupational therapy group at the time

	orotracheal intubation is performed. The intervention will be carried out exclusively during the stay in the ICU.
Comparator	Late multimodal therapy is defined as the initiation of multimodal therapy 3 days or more after the start of mechanical ventilation. The comparator differs from the study intervention only in the moment (time from orotracheal intubation to the start of multimodal therapy) of initiation of therapy.
Outcomes	Effectiveness<br>Primary outcome: Number of days with invasive mechanical ventilation in the ICU<br>Secondary outcomes:<br>• Days free of mechanical ventilation until day 28<br>• Days of delirium until day 28<br>• Time in ICU with delirium until day 28<br>• Days of hospitalization with delirium until day 28<br>• Sedation days until day 28<br>• Sedation-free days until day 28<br>• Barthel index at discharge<br>• Days of hospital stay<br>• Length of stay in ICU<br>• Mortality from any cause up to 90 days<br>• Time from intubation to start of therapy<br>• Muscle strength measured with the medical research scale council (MRC)<br>• Dysphagia at 72 hours<br>• Time until initiation of oral administration<br>• Development of healthcare-associated pneumonia,<br>• Maximum mobility measured by JH-HLM<br>• Grip strength<br>• Extubation failure

	• Need for non-invasive mechanical ventilation<br>• Cognitive function is measured using the Montreal Cognitive Assessment (MOCA)<br>• Quick sensitivity and dexterity test<br>Security<br>Outcomes<br>• Number of adverse events<br>○ Altered blood pressure<br>○ Cardiac arrhythmia<br>○ oxygen desaturation<br>○ Pain or agitation<br>○ Invasive line removal<br>○ Gastrointestinal (nausea, vomiting, or diarrhea)<br>○ tachypnea<br>○ Altered neurological status<br>• Number of serious adverse events<br>• Unscheduled extubation<br>• Healthcare-associated pneumonia<br>• Bronchoaspiration
Time	Screening: Verification of the inclusion and exclusion criteria of patients will take a maximum of 24 hours after endotracheal intubation.<br>Intervention: For both study groups, multimodal therapy will be applied exclusively while the patient is in the ICU.<br>Follow-up:<br>At the beginning of the study, the variables described in point 6.6 will be measured.<br>At the time of weaning from ventilatory support, the following variables will be measured: delta POCC, muscle pressure, P0.1, number of secretions per orotracheal tube, asynchrony index, NIF, % leak, peak cough flow, measurement of diaphragmatic excursion,

	measurement percentage of diaphragmatic thickness, and tracheobronchial secretions.<br><br>On the day of hospital discharge, the variables recorded in point 6.6 will be measured.<br>The last measurement of variables will be carried out 90 days after the patient is intubated by point 6.6.

4. Theoretical Framework and State-of-the-Art

In recent decades, there has been an increase in the demand for intensive care units (ICUs) worldwide, accompanied by the admission of older patients with more complex pathologies, previously considered untreatable. Medical, social, and technological advances have allowed for increased survival of patients admitted to ICUs, with the possibility of offering and providing support to various vital organs. (1–3) In the United Kingdom, the number of patients admitted to ICUs nearly doubled between 2009 and 2018. (3) The increase in ICU stays and readmission requires the persistent use of invasive measures and interventions in these patients. It is estimated that the aging population will continue to grow, along with the need for ICU beds and care. (1–3)

While the criteria for ICU admission have been extended, allowing care for a larger number of patients and reducing their mortality, the advent of new technologies and the use of invasive measures, particularly invasive mechanical ventilation, also bring inherent risks. Consequently, ICU patients, even after recovering from the acute pathology, have higher morbidity and mortality and experience deterioration in multiple aspects of their lives, which can persist for years after their hospital stay. (3) This has led to growing concern about adequately rehabilitating these patients and ensuring their overall recovery, including social, physical, psychological, and quality of life aspects. (2)

The terms **ICU-acquired weakness (ICU-AW)** and **post-intensive care syndrome (PICS)** have emerged to encompass the set of physical, mental, and neurocognitive alterations widely described in ICU patients. Among these, the loss of muscle mass and function secondary to myopathy and neuropathy due to alterations in ion channels involved in nerve conduction, as well as primary distal axonal degeneration of both motor and sensory neurons with consequent denervation atrophy, are highlighted. There is also a muscular component related to the decoupling between excitation and contraction and functional mitochondrial capacity, ultimately leading to the loss of muscle regeneration capacity. (2,4) Muscle weakness in these patients affects functional capacity, delays

recovery, impedes weaning from mechanical ventilation, increases costs, and reduces the quality of life of survivors. (5)

Multiple factors can contribute to the development of PICS, including metabolic and neuroendocrine processes directly related to the critical condition of these patients. However, other factors have been documented that may be at least partially induced by medical interventions, procedures, and medications implemented in the ICU, making them potentially preventable or modifiable. (2,6,7)

Swallowing disorders are common in patients with PICS, with prevalence varying in different studies depending on the chosen population, study design, and diagnostic criteria used, ranging from 3-70%. (1,8–10) It is estimated that more than one in six patients admitted in an emergency condition and at least one in ten medical-surgical patients are affected by post-extubation dysphagia. (11)

In some cases, dysphagia may resolve or significantly improve within days, while in other cases, it may persist for long periods. (12) Specifically in the postoperative context, the incidence of dysphagia has been reported to be around 18.3%, with up to 80% persisting at ICU discharge and more than 60% persisting until hospital discharge. (10) Additionally, postoperative dysphagia has been established as an independent predictor of 28- and 90-day mortality. (12) Therefore, the early use of strategies for recognizing post-extubation dysphagia and regular and periodic reevaluation in the ICU should be a standard of care. (13)

Swallowing disorders are closely associated with post-extubation aspiration events, which have been persistently linked to deleterious outcomes for patients, including increased morbidity and mortality, longer hospital and ICU stays, the need for re-intubation, and pneumonia, among others. (1)

Safe and efficient swallowing is a complex process that requires the coordination of various structures; and the execution of both cranial and spinal nerves to convert respiratory impulses into digestive ones and vice versa. It also involves structures of the upper aerodigestive tract, where the closure of the laryngeal vestibule is crucial, which must be performed in a short period, leaving very little margin for error. (1)

The mechanisms of postoperative dysphagia can be multifactorial, involving decreased respiratory capacity due to reduced respiratory and bulbar reserve, deconditioning, and disuse atrophy; the presence of cognitive disorders associated with the use of sedation and medications acting on the central nervous system, either by directly compromising the state of consciousness or by intervening in feedback systems of nerve conduction, ultimately affecting the ability to execute and coordinate precise movements for safe and efficient swallowing; mechanical trauma caused by some invasive measures such as the orotracheal tube or feeding tubes, either at the time of insertion or removal, which increases with prolonged use, inappropriate size selection (oversizing), and frequent traumatic maneuvers in acute contexts. (1,9) Overinflation of the endotracheal tube cuff can compress the arytenoid cartilages or the recurrent laryngeal nerve and dislocate the cricothyroid and/or cricoarytenoid joint, affecting the closure of the laryngeal vestibule. (1,14) Another cause of

mechanical trauma that is particularly important in cardiovascular surgery patients is the frequent use of transesophageal echocardiography. (1)

The duration of intubation influences the period of disuse of the bulbar musculature for both speech and swallowing, producing in some cases diffuse atrophy, weakness, and impaired ability to generate the necessary lingual and pharyngeal pressure for bolus propulsion and clearance, putting patients at risk of aspiration. (1,8,10–12) Additionally, endotracheal tubes impede normal swallowing function and active laryngeal elevation, reducing the passive opening of the upper esophageal sphincter, which impedes rapid esophageal passage, contributing to the generation and perpetuation of dysphagia. (10)

In addition to pharyngeal structures, the somatosensory and motor functions of the tongue, which are essential for taste, chewing, swallowing, and speech, can be compromised during ICU stay. (15) This can contribute to dysarthria, dysphonia, dysphagia, chewing disorders, and consequences such as aspiration, pneumonia, dehydration, malnutrition, social withdrawal, and reduced quality of life for these patients. (10,12,15,16)

There are several moments when tongue function can be affected by mechanical ventilation. From the moment of endotracheal tube insertion with a laryngoscope that compresses and deviates the tongue during the procedure, lingual nerve injuries can occur, resulting in loss of sensation. Additionally, immobilization or reduced frequency and range of tongue movement during intubation can decrease its contractile strength. (10,15,16) It has been shown that somatosensory and motor impairment of the tongue occurs regardless of age, sex, smoking history, and patient comorbidities. Compared to a control group, the decrease in strength was more persistent than sensory alteration, which has shown at least partial improvement within 14 days post-extubation. (15)

Post-extubation dysphagia is associated with delayed resumption of oral intake, potentially increasing the risks of dehydration and malnutrition in patients who are often in hypercatabolic processes, which is particularly important in the elderly population, who are often frail and have limited functional reserve. (1,9) Other factors associated with swallowing disorders in ICU patients include gastroesophageal reflux and desynchrony between breathing and swallowing processes. (10)

Extubation failure, usually defined as the need for re-intubation within 24-72 hours following planned extubation, leads to a worse prognosis, increased mortality, and hospital stay, and can occur in 2-25% of patients. Causes include swallowing dysfunction, ineffective cough, upper airway obstruction, and excessive secretions in the airway. (17) In a related study of patients in a medical-surgical ICU, intubated for more than 6 days, it was found that the gag reflex is a determinant for extubation failure related to aspiration or excessive secretions in the airway. (17) Both frailty and generalized weakness have been associated with impaired ability to generate an effective cough to expel tracheal material. (1)

The growing concern to minimize the impact of medical interventions on the overall recovery process of patients has led to a cultural shift in ICUs, where it was previously more common to find most patients deeply sedated and on invasive mechanical ventilation, sometimes for prolonged periods, and today, fostering an increasingly conducive environment for rehabilitation. (18)

Physical therapy and early mobilization

While in previous decades, less than 50% of patients with acute lung injury requiring orotracheal intubation received a swallowing assessment during their hospitalization, (19) nowadays, multidisciplinary rehabilitation groups have increasingly been incorporated. (7,20–22) In a study conducted over 5 years in the United States, which included 264,137 patients requiring mechanical ventilation, it was found that patients received physical, occupational, and speech therapy variably. Despite awareness of mobilizing these patients, rehabilitation does not start as early as possible. Only 24% of patients received physical therapy on the same day as mechanical ventilation. (23) There is less information available in the literature, but in this study, only 12.2% of patients under mechanical ventilation received occupational therapy, while 33% received speech therapy orders. (23)

Rehabilitation therapy varies significantly between geographic regions, but generally, it can involve, in addition to the medical team, professionals in physical therapy, respiratory therapy, speech therapy, occupational therapy, and nursing. In countries without direct availability of specialized therapies, either due to training issues or resource availability, the nursing or physical therapy team assumes the role of occupational, respiratory, and speech therapy. (20–24)

Physical rehabilitation is crucial for patients admitted to the intensive care unit as 20-50% of critically ill patients experience some degree of weakness. Currently, mobilization performed in intensive care units is accepted as a therapeutic tool with the potential to prevent or mitigate functional deterioration in these patients. However, the ideal timing for initiating this strategy has been widely debated. (3,5)

Early mobilization has been proposed as an attractive policy for this group of patients by some authors. In some studies, it has shown adequate tolerance with less incidence of delirium, more ventilator-free days, and better functional outcomes at hospital discharge. (2,25–27)

However, its benefit has been widely debated, as some studies have not shown improved outcomes, and some have even reported an increase in adverse events. This has been attributed to multiple

irregularities when comparing studies: primarily, there is a lack of standardization of the concept of early, which in many cases is not even defined. There is also no consensus on the established therapy, the comparator, or "usual therapy," which varies widely depending on institutional policies, inclusion and exclusion criteria, the frequency of interventions, and outcomes. (3) Another factor that may contribute to the lack of improvement in outcomes is the heterogeneity of patients, both in their baseline condition, their pathology, and their response to the proposed interventions in the unit. (3)

In a recent study published in the New England Journal of Medicine (6) that included 750 patients in 49 hospitals across 6 countries, and who were randomized to early mobilization or usual care, there was no difference in 180-day mortality, hospital stay, or secondary outcomes such as days of mechanical ventilation and days out of the ICU. However, there was an increase in adverse events and serious adverse events in the early mobilization group. The authors report limitations such as a higher level of mobilization in the control group compared to previous studies, barriers to mobilization that may have limited the statistical power to detect differences between groups, and surveillance bias in the early mobilization group. (6)

In a meta-analysis and systematic review of the literature published in 2019, which included 23 randomized controlled trials, it was concluded that regardless of the different techniques and periods of mobilization used, early mobilization of critically ill patients increased the number of people able to stand, the number of ventilator-free days during hospitalization, reduced the incidence of intensive care unit-acquired weakness, and increased the walking distance at hospital discharge. There were no significant differences in 28-day mortality or adverse events in this study. (5)

Types of interventions to consider include active functional mobilization, in-bed cycling ergometry, electrical muscle stimulation (with or without active or passive exercises), and tilt tables, among others. (4) While passive exercises are not formally part of the rehabilitation process, as they have not been shown to increase muscle strength or endurance, (3) they often precede the initiation of active maneuvers and are most used in patients under deep sedation whose clinical condition allows it. (7,24,26,27)

Therapy should be individualized to the patient's condition as much as possible. However, Hickman et al. found only 5 contraindications for establishing early mobilization, which in their study was defined as initiated within the first 24 hours of ICU stay: acute myocardial infarction, active bleeding, increased intracranial pressure, and unstable pelvic fracture. (25) In this group of patients, hemodynamic parameters were hardly affected by therapy, causing discontinuation in only 0.8% of activities, mainly due to hypotension or arrhythmias. (25)

A method to determine the risk/benefit of applying early mobilization to patients in the intensive care unit was developed by a multidisciplinary group and is widely used today. It consists of traffic light coding according to risk, where green is assigned to patients with a low risk of adverse events, meaning the benefit outweighs the potential safety consequences. Yellow represents a risk of adverse events, and in this group of patients, there should be a discussion of precautions and contraindications for early mobilization. The red category implies a higher risk of adverse events, and in these patients, early mobilization is not performed unless authorized by the responsible medical team. While this category does not constitute a contraindication, it is a warning that, in this case, the risks may outweigh the benefits. (20)

Different clinical guidelines support the use of a bundle to reduce or shorten rehabilitation time and the incidence of delirium. The ABCDEF bundle includes the assessment, prevention, and management of pain; awakening and breathing trials, the assessment, prevention, and management of delirium, early mobilization and exercise, and involving and favoring the patient's family environment. (21)

Even in teams that promote early rehabilitation, studies have found multiple barriers to mobilizing patients on mechanical ventilation and maintaining the proposed dose or intervention time. (28)

In practice, offering early mobilization can be challenging as it requires additional time, specialized professionals and materials, and a coordinated team approach. (26)

Barriers to early mobilization include patient-related factors, such as signs and symptoms or conditions like hemodynamic or respiratory instability; structural barriers, such as human and equipment resources or technical issues; barriers related to ICU culture, including ingrained habits and attitudes in each institution, and process-related limitations, lack of coordination, absence of roles and rules that adequately determine and distribute tasks and responsibilities. (27)

One of the most frequent barriers or reasons for not adhering to mobilization protocols is high levels of sedation. Adequate reduction and adjustment of sedation to allow patient participation in exercises is important for favorable outcomes. (27) Other important factors are proper pain management and early recognition and management of delirium. (27)

The recovery goal should be established as early as possible for each patient. Along with it, strategies should be implemented to improve the implementation of comprehensive rehabilitation, including identifying barriers and facilitating factors; and creating multidisciplinary teams with leaders to improve communication, education, and training. (3)

Patient safety criteria are a frequently reported barrier. With mobilization, there is an increased risk of removing invasive measures such as catheters, tubes, and equipment, and it is a common belief among doctors and nurses that limits the role of physical therapy and compromises the implementation of early mobilization protocols. However, this risk is negligible when the program is carried out by trained and qualified personnel. (27)

To improve cultural concerns in the ICU, an international multicenter randomized study implemented the establishment of mobilization goals during the daily medical rounds, achieving them on 89% of days in the intervention group. In this study, shorter ICU and hospital stays were achieved, and functional mobility at hospital discharge improved. (7)

Speech therapy

The role of speech therapy or speech-language pathology is becoming increasingly important in intensive care, as like early mobilization, speech therapy should be involved in the early assessment of ICU patients. Early recognition of laryngeal dysfunction and swallowing disorders can minimize the risk of oropharyngeal dysphagia and aspiration. (18) Speech therapists can provide support for critically ill patients with acute conditions, whether neurological or of other medical etiologies: trauma, hemorrhages, tumors, spinal injuries, respiratory conditions, as well as patients in complex postoperative states from neurological, cardiac, and general surgery interventions. These conditions can affect motor speech skills, swallowing, coughing, and voice. (18) Early support from speech therapy helps facilitate successful communication between ICU patients and the medical team and their families, developing specialized intervention programs for each condition and working with patients from a functional and pathological perspective. This, in turn, improves their psychosocial well-being, engagement in day-to-day care, and informed decision-making. Additionally, communication disorders have been associated with delirium, an emerging problem in intensive care units. (18,23) The video fluoroscopic swallowing study (VFSS) and the fiberoptic endoscopic evaluation of swallowing (FEES) are necessary for the accurate diagnosis of swallowing disorders, as aspiration is a devastating complication that can occur silently in 30-44% of patients and cannot be diagnosed with bedside evaluations. Factors associated with aspiration processes include pneumonia, feeding tubes, tracheostomy, prolonged ICU stays, and increased hospital mortality. (29) FEES, unlike VFSS, is a portable tool with the additional benefit of providing a direct view of pharyngeal and laryngeal dynamics, as well as the patient's anatomy and secretion management, thus supporting clinical decisions and often highlighting difficulties that can later be resolved. (18) The use of FEES allows for pharmacological recommendations for managing excessive secretions, as well as therapeutic interventions to strengthen the tongue base, pharyngeal constriction, and laryngeal range of motion, making it, along with VFSS, the gold standard for detecting swallowing disorders. (18,29) However, cost, occasional patient refusal, transportation difficulties, and the requirement for specialized personnel and equipment limit the application of these tools in all patients post-extubation. (18,19) The ability to easily and accurately diagnose aspiration could minimize the complications of aspiration, limiting unnecessary delays in initiating oral intake in ICU patients. Therefore, multiple specialized protocols and tools have been designed to identify patients with established or at-risk swallowing disorders, aiming to adopt a preventive approach to reduce complications and deleterious outcomes. (18,19) Among these, a combination of the water swallow test and a bedside swallowing evaluation is the only strategy validated for identifying post-extubation dysphagia in survivors of acute respiratory failure. (13,29) The Gugging Swallowing Screen (GUSS) is an easy-to-apply tool by different therapists or nurses, allowing for a graded assessment of the patient's swallowing capacity and providing nutritional recommendations. (13) In

a recent study, the GUSS-ICU was compared with FEES, demonstrating 89-92% sensitivity and 67-89% specificity for detecting patients with dysphagia, comparable to results obtained in stroke patients. The inter-observer reliability was good. This tool has the advantage of simplicity and the use of various food consistencies, allowing for dietary recommendations for patients in the post-extubation context. In addition to strengthening oropharyngeal musculature with exercises, electrical stimulation at this level has recently been proposed. Some studies have shown it improves the reorganization of the motor cortex related to swallowing, facilitates the activation of corticobulbar pathways, and increases salivary levels of neurotransmitters associated with swallowing, such as substance P. (8) The PHAST-TRAC study evaluated pharyngeal electrical stimulation in tracheostomized stroke patients with neurogenic dysphagia, finding that this strategy led to a higher number of patients ready for decannulation, as well as a lower prevalence of pneumonia, without an increase in complications. (30) In the last decade, the value and expertise of therapists supporting the care of patients in the intensive care unit have been highlighted. (22) Each therapy-related profession offers a wide range of experience, knowledge, skills, and expertise that contribute in different ways to the early, optimal, and comprehensive rehabilitation of ICU patients. Rehabilitation goals should be individualized for each patient, and therapy should be formulated according to these goals. Emphasis should be placed on collaborative interdisciplinary work and understanding the role and responsibilities of each professional. (10,12,18,22,23,31)

5. Objectives

General Objective:

To evaluate the difference in days of invasive mechanical ventilation between early multimodal therapy and late multimodal therapy in the adult intensive care unit at Fundación Santa Fe de Bogotá.

Specific Objectives:

- To describe the sociodemographic and clinical characteristics of patients with invasive mechanical ventilation in the Neurological Intensive Care Unit at Fundación Santa Fe de Bogotá.
- To evaluate the efficacy of early multimodal therapy versus late multimodal therapy in terms of ventilator-free days and other secondary outcomes.

- To establish the sociodemographic and clinical factors associated with the type of therapy (early or late) the primary outcome (ventilator-free days) and secondary outcomes.
- To evaluate the safety of early multimodal therapy versus late multimodal therapy in terms of adverse events, serious adverse events, episodes of broncho-aspiration, and healthcare-associated pneumonia.
- To evaluate the impact of different predefined subgroups, such as the type of respiratory failure (hypoxemic or hypercapnic) and the type of patient (surgical, respiratory, burn, cardiovascular), on the primary outcome results.

Hypotheses*:*

Alternative Hypothesis: Early multimodal therapy significantly reduces the number of days of invasive mechanical ventilation compared to late multimodal therapy in intubated patients in the intensive care unit.

Null Hypothesis: There are no statistically significant differences in the number of days of invasive mechanical ventilation when comparing early multimodal therapy to late multimodal therapy in intubated patients in the intensive care unit.

6. Methodology

6.1. Study design

A phase III, randomized, controlled clinical trial will be conducted at a single center, with two parallel groups of patients. Patients who meet the inclusion criteria will be assigned, through stratified randomization by sex and age group, to one of the two study groups at a 1:1 ratio. Allocation concealment will be achieved using opaque, sealed, and sequentially numbered envelopes. In the first group, called the "early arm" (EA), patients who have been intubated in the intensive care unit or the emergency and/or surgery rooms will be included, and they will receive multimodal therapy early (<24 hours). In the second group, called the "late arm," multimodal therapy will also be applied, but it will start at 72 hours of mechanical ventilation. Due to the methodological characteristics of the study, it will not be possible to blind the participants or the researchers regarding the group to which they belong. However, the healthcare professionals who will perform the final evaluation on day 90 of follow-up will not be informed about the intervention group the participant was in. Similarly, the statisticians and epidemiologists who will analyze the data will be blinded to the intervention performed in each group. Finally, the study will be analyzed by intention to treat.

6.2. Population

Inclusion Criteria: Must meet all the following criteria:

Adult patients (over 18 years old)

Patients hospitalized in the adult intensive care unit at Fundación Santa Fe de Bogotá who require invasive mechanical ventilation through an endotracheal tube for a period longer than 24 hours.

Barthel Index greater than or equal to 70

Exclusion Criteria:

Patients requiring invasive mechanical ventilation through a tracheostomy cannula or nasotracheal tube

Patients who have undergone any type of head and neck surgery

Cardiac arrest

Airway burns

Burns covering more than 50% of the body surface area

Patients with chronic obstructive pulmonary disease (COPD)

Patients referred from another institution

Demyelinating diseases or neuromuscular junction disorders

Patients requiring neuromuscular relaxation

Patients with a life expectancy of less than 180 days

Patients who, by medical criteria, would not benefit from multimodal treatment

Patients not admitted to the ICU for the first time

Patients participating in other rehabilitation clinical trials

Patients with liver or kidney transplants

Patients who have undergone cytoreductive surgery (Sugar-Baker)

Evaluation and Patient Withdrawal Criteria:

Changes in Participant's Health Status: If a participant develops health conditions that could affect the validity of the results, such as acute myocardial infarctions, pulmonary thromboembolism, intracerebral hemorrhages, cardiac dysfunction (requiring inotropic support), and multiple surgical washouts.

Lack of Compliance: If a participant does not meet the study requirements or does not follow the protocol guidelines, they may be withdrawn to maintain data integrity.

Participant Decisions: A participant has the right to withdraw from the study at any time for any reason, without penalty.

Interim Results: If significant results are discovered during the study that make it ethically necessary to stop the study to protect participants.

Study Completion Criteria: Once sufficient data have been collected to answer the research questions, the remaining participants may be withdrawn.

6.3. Sample size

Based on the study published by Schweickert et al. where they found that the mean number of days of mechanical ventilation in patients with usual physical therapy was 6.1 ± 1.4 days and in early therapy it was 3.4 ± 1.25 days (32), for an alpha error of 0.05 and a statistical power of 0.8 is considered the following formula:

Next, we will calculate the effect size (ES) using the difference in means between the groups and the standard deviations:

$$ES = \frac{|\ \mu 1 - \mu 2\ |}{\sqrt{DE1^2 - DE2^2/_2}}$$

$$ES = \frac{|\ 6.1 - 3.4\ |}{\sqrt{1.40^2 - 1.25^2/_2}}$$

$$ES = \frac{2.7}{\sqrt{3.52/_2}}$$

$$ES = 2.03$$

With the effect size calculated (ES ≈ 0.4463), we can use the formula for the sample size mentioned earlier:

$$n = \frac{2\ (\frac{\mathrm{Z\alpha}}{2} + Z\beta)^2\ .\ (\sigma 1^2 +\ \sigma 2^2)}{\mathrm{ES}^2}$$

Where $\frac{\mathrm{Z\alpha}}{2}$ is the critical value corresponding to the alpha significance level /2 to α = 0.025, $\frac{\mathrm{Z\alpha}}{2}$ ≈ 2.81

and $Z\beta$ is the critical value corresponding to the statistical power beta to β = 0.90, $Z\beta$ ≈ 1.28

The other values we have are the same:

- Mean of group 1 (μ1) = 6.1 days
- Standard deviation of group 1 (σ1) = 1.4 days
- Mean of group 2 (μ2) = 3.4 days
- Standard deviation of group 2 (σ2) = 1.25 days

Effect size (ES) ≈ 20.03 (previously calculated)

Substituting these values into the formula:

$$n = \frac{2\ (2.81 + 1.28)^2\ .\ ((1.40)^2 +\ (1.25)^2)}{(2.03)^2}$$

$$n = \frac{2\ (4.09)^2\ .\ (1.96 + 1.56)}{4.13}$$

$$n = \frac{2\ (16.72)\ .\ (3.52)}{4.13}$$

$$n = \frac{(35.35).(3.52)}{4.14}$$

$$n = 30.29$$

Rounding:

$$n = 31 \text{ by each group}$$

To the estimated 31 patients per arm, an extra 20% (6 patients per arm) is added to minimize the loss of statistical power in case patients withdraw from the study or there are missing data.

The sample size has also been determined based on the magnitude of the expected effect of mobilization within 48-72 hours of mechanical ventilation, as suggested in the reference study (33), which justifies our choice to evaluate early mobilization at 24 hours versus 72 hours after mechanical ventilation in our study.

6.4. Sampling and Randomization

Patients who meet the inclusion criteria will be assigned, through stratified randomization by sex and age group (under 65 years and over 65 years), to one of the two study groups at a 1:1 ratio (sampling stratified randomized). The number of resulting strata corresponds to 4. Randomization will be applied to each stratum. This type of sampling and randomization reduces possible imbalances and increases statistical power, considering that the number of participants per arm is 37. Allocation concealment will be carried out using sealed and sequentially numbered opaque envelopes, which will be in the Intensive Care Unit office on the fourth floor of the expansion building. A record of the person who opened the envelope will be kept by Good Clinical Practice guidelines.

6.5. Intervention

Early multimodal therapy is defined as the set of therapeutic maneuvers performed by the physiotherapy, speech therapy, respiratory therapy, and occupational therapy team within 24 hours of orotracheal intubation. In contrast, late multimodal therapy is characterized by the same type of maneuvers performed by the same disciplinary group at 72 hours of orotracheal intubation.

Multimodal therapy will be carried out according to the results of the Richmond Agitation-Sedation Scale (RASS). All personnel involved in our study will receive specific training in the accurate assessment of this scale, to reduce intra- and inter-evaluator variability in the application of the intervention.

It is clarified that the set of therapeutic maneuvers carried out in both groups is an integral part of the standard care provided to our patients in the intensive care unit. Additionally, both groups will receive all the medical interventions that would normally be applied during their ICU stay, regardless of their participation in the study. This ensures consistency in care during the study.

Standard management in our ICU involves specialized and continuous care for patients with severe illnesses or those requiring constant monitoring. These include:

Continuous Monitoring: Continuous monitoring of vital signs such as heart rate, blood pressure, respiratory rate, and oxygen saturation to detect immediate changes in the patient's condition.

Life Support: Providing advanced life support, which may include oxygen administration, mechanical ventilation, medications to maintain blood pressure, and other treatments necessary to maintain physiological stability.

Pain and Sedation Management: Adequate management of pain and sedation to ensure patient comfort and facilitate necessary medical procedures.

Infection Prevention and Treatment: Implementation of infection control measures to prevent the spread of diseases, as well as the use of antibiotics and other specific treatments to combat infections.

Specialized Nutrition: Providing enteral or parenteral nutrition according to the individual needs of patients, especially if they cannot feed orally.

Fluid Management: Carefully controlling fluid and electrolyte levels to maintain fluid balance and correct imbalances if necessary.

Multidisciplinary Coordination: Promoting collaboration among healthcare professionals, including doctors, nurses, therapists, and other specialists, to ensure comprehensive and personalized care.

Communication with Family Members: Maintaining clear and regular communication with the patient's family, providing information about the patient's condition, and offering emotional support.

Crisis Management: Being prepared to address emergencies and make quick and effective decisions in response to acute changes in the patient's condition.

Interventions will be performed daily once the patient has been included in the study and will be carried out according to the expert consensus recommending mobilization maneuvers when the patient has an FIO2 less than or equal to 0.6, a percutaneous oxygen saturation greater than or equal to 90%, a respiratory rate less than 30 breaths per minute, and a PEEP less than or equal to 10 cmH2O.

These maneuvers will be performed until the patient is discharged (defined as the moment when the intensivist determines that the patient can be transferred to hospitalization or discharged from the hospital) from the ICU.

Speech Therapy Group Interventions

The speech therapy group will assess the sedation level using the Richmond Agitation-Sedation Scale (RASS). For patients who are unresponsive or have a RASS score of -2 or lower, passive/assisted proprioceptive speech therapy will be carried out. This involves procedures such as craniofacial alignment, mandibular mobilization, lingual mobilization, cervical mobilization, pharyngeal reflex stimulation, orofacial muscle activation, oral mucosa hydration, and supra- and infrahyoid muscle activation.

On the other hand, for patients with a RASS score between -1 and +1, active/assisted neuromuscular praxis speech therapy will be applied. This involves the active performance of procedures such as craniofacial alignment, mandibular mobilization, lingual mobilization, cervical mobilization, pharyngeal reflex stimulation, orofacial muscle activation, oral mucosa hydration, and active supra- and infrahyoid muscle activation. The details of each intervention are provided in Annex 1.

Physical Therapy Group Interventions

From the field of physical therapy, an assessment will be conducted using the Richmond Agitation-Sedation Scale (RASS). For patients who are unresponsive or have a RASS score of -2 or lower, a passive/assisted proprioceptive intervention will be applied. This involves techniques such as facilitation of muscle fiber recruitment, inhibitory reflex positioning, particular mobilization, joint loading, and approximation, as well as activation of joint receptors.

For patients with a RASS score between -1 and +1, motor activation intervention using proprioceptive neuromuscular facilitation techniques will be performed. Additionally, electrostimulation will be applied to large muscle groups and accessory respiratory and diaphragmatic muscles. This is done to promote intentional movement and plan and execute dynamic motor patterns that help trigger support and balance reactions, to promote maximum functional development in critically ill patients. A detailed description of the intervention is provided in Annex 1.

For patients who can tolerate motor activation intervention (RASS between -1 and +1), bed mobility exercises, transfer exercises from bed to chair or chair to bed, pre-gait exercises, and finally gait training will be performed. The progression will depend on the patient's tolerance and stability.

Respiratory therapy group interventions

The intervention performed by the respiratory therapy group is divided into three main categories:

Airway Clearance Techniques: Methods to ensure the airway remains clear of obstructions, such as mucus or foreign bodies.

Lung Re-expansion Techniques: Procedures aimed at improving lung expansion and preventing atelectasis.

Respiratory Muscle Strengthening Techniques: Exercises and interventions designed to enhance the strength and endurance of the respiratory muscles.

The detailed description of the interventions performed by the respiratory therapy group is comprehensively described in Annex 1.

Occupational therapy group interventions

To carry out this process, if the patient presents a level of consciousness with a RASS score of -3 or lower, multisensory stimulation activities will be performed. These activities include tactile, proprioceptive, vestibular, gustatory, auditory, and olfactory stimulation, fully assisted by the therapist. These activities should be related to simulated daily life tasks and include upper limb movement patterns, with an emphasis on primary patterns such as hand-to-head, hand-to-mouth, homolateral hand, contralateral hand, and hand-to-perineum, in addition to caring for the joints and feet.

For patients with a RASS score between -2 and -1, interventions will be performed in a passive-assisted manner and will focus on sensory stimulation, along with cognitive feedback to recognize elements and perform daily tasks. Instructions will be given with a maximum of two commands, using integral movement patterns such as reaching, grasping, releasing, and throwing, with an emphasis on frontal and lateral planes in the bed.

When the patient has a RASS score of 0 to +1, the intervention focuses on the patient's active and autonomous participation in activities, with minimal support from the therapist according to the patient's tolerance. Emphasis is placed on autonomy in daily tasks, integrating sensory and cognitive commands of low, medium, and high complexity, using upper limb movement patterns previously worked on in all anatomical planes, either in bed, at the edge of the bed, or in a chair, depending on the progress in rehabilitation.

For patients with a RASS score of +2 or higher, therapeutic sessions focus on behavioral and environmental modulation, including the physical and family environment. Connection with the environment is promoted through inhibitory multisensory stimulation activities and cognitive stimulation oriented towards meaningful tasks, with active assistance in bed. Additionally, the family is educated on the importance of maintaining continuous orientation, and sensory stimuli are enriched to converge on a common task, using visual, auditory, tactile, and proprioceptive stimuli (see Annex 1).

6.6. Outcomes

Effectiveness

Main outcome: time (days) from intubation to extubating.

● Secondary outcomes:
○ Days free of mechanical ventilation until day 28
○ Days of delirium until day 28
○ Time in ICU with delirium until day 28
○ Days of hospitalization with delirium until day 28
○ Days of sedation until day 28
○ Sedation-free days until day 28
○ Extubating failure
○ Need for non-invasive mechanical ventilation

● Exploratory outcomes:
○ Barthel index at discharge
○ Days of hospital stay
○ Length of stay in ICU
○ Mortality from any cause up to 90 days
○ Time from intubation to start of therapy
○ Muscular strength measured with the Medical Research Council (MRC) scale
○ Dysphagia 72 hours after extubation
○ Dysphonia 72 hours after extubation
○ Time until initiation of oral administration post-extubation
○ Development of healthcare-associated pneumonia,
○ Maximum mobility measured by JH-HLM
○ Grip strength
○ Cognitive function measured using the Montreal Cognitive Assessment (MOCA)
○ Evaluation of functionality in daily activities until day 28
○ Quick sensitivity and dexterity test

Safety

● Outcomes:
○ Number of adverse events.
■ Altered blood pressure
■ Cardiac arrhythmia
■ Oxygen desaturation
■ Pain or agitation
■ Invasive line removal

- ■ Gastrointestinal (nausea, vomiting or diarrhea)
- ■ Tachypnea
- ■ Altered neurological status
- ○ Number of serious adverse events
- ○ Unscheduled extubation
- ○ Healthcare-associated pneumonia
- ○ Bronchoaspiration

6.7. Measurements

Within the framework of the protocol, measurements will be conducted at six key moments of the research: upon admission to the intensive care unit, during observation in the intensive care unit, at the start of ventilatory support weaning, post-extubation, upon hospital discharge, and at the end of the observation period. Additionally, continuous measurements of outcomes associated with the safety of the intervention will be performed.

Upon admission, crucial details about each patient will be collected, such as name, identifier code, medical record number, sex, and ethnicity. Health and functionality aspects will also be evaluated, including the Barthel Index and Body Mass Index. Diagnostic information will be complemented with the ICD-10 code, study group (early vs. late), and the unit of admission (surgical, neurological, septic and respiratory, cardiovascular, or burn unit). Severity scores such as SOFA, APACHE II, and SAPS II will be incorporated, along with temporal data such as admission date, discharge date, and the time elapsed from intubation to the start of therapy. Regarding respiratory parameters, fundamental variables at the time of intubation will be detailed, such as tidal volume, respiratory rate, PEEP, plateau pressure, static lung compliance, driving pressure, airway resistance, and the PaO2/FIO2 ratio.

During the observation phase, a rigorous daily patient evaluation process will be implemented to ensure accurate recording of their progress. Key variables will be monitored, such as days on mechanical ventilation (recording start and end dates), ventilator-free days, days with delirium (indicating start and end dates), days of hospitalization with delirium, and days of sedation (with details of start and end dates). The duration of mechanical ventilation, the possible need for tracheostomy, the presence of adult respiratory distress syndrome, and the eventual occurrence of healthcare-associated pneumonia will also be documented.

In the weaning initiation phase, attention will focus on a series of key parameters to assess the patient's ability to discontinue mechanical ventilation. Detailed data will be recorded, such as the Delta POCC, reflecting the variation in vital capacity during a cough cycle, muscle pressure, P0.1 representing the pressure in the first 0.1 seconds of inspiration, the amount of secretions through the orotracheal tube, the asynchrony index, negative inspiratory force (NIF), leak percentage, peak cough flow, and specific measurements related to diaphragmatic capacity, such as excursion and thickness. These data provide a comprehensive view of respiratory functionality during the weaning

process, allowing informed decision-making about the feasibility of withdrawing mechanical ventilation.

Before the extubation process, critical data will be collected to assess the patient's readiness and minimize potential risks. The Delta POCC, muscle pressure, P0.1, the number of secretions through the orotracheal tube, the asynchrony index, NIF, leak percentage, peak cough flow, diaphragmatic excursion, and thickness will be recorded. Additionally, the OMAHA score at the time of extubation will be documented, indicating the presence or absence of this event.

The decision to extubate a patient, according to the OMAHA+ Scale, is based on a comprehensive evaluation of various clinical parameters. Each component of this scale provides crucial information about the patient's ability to tolerate the withdrawal of ventilatory support. When all factors described in the OMAHA+ Scale are present, extubation is performed.

The systematic application of the OMAHA+ Scale is fully standardized in our unit, forming an essential part of our extubation protocol. This ensures a thorough and uniform evaluation of patients before withdrawing ventilatory support. Additionally, we have implemented a systematic review procedure of the process to strengthen the consistency, accuracy, and quality of our clinical practice.

The OMAHA+ Scale is detailed below:

1. Oxygenation (O): The PaO_2/FiO_2 ratio > 150, arterial oxygen saturation > 90% with FIO_2 0.4, PEEP ≤ 8 cm H20 and Tobin's index (fr/Vt) < 105. Each variable will be marked as yes or no.

2. Respiratory Mechanics (M): exhaled tidal volume greater than 5 ml/kg vital capacity > 10 ml/kg, maximum inspiratory pressure (MIP) ≤ -20 to 25 cmH20, respiratory rate < 35 per minute, and values of PIM and PEM.

3. Acid-Base (A): $paCO_2$ < 50 mmHg, pH > 7.32, lactic acid > 2, and venous saturation > 75%.

4. Hemodynamic (H): Systolic pressure > 90 or < 160, heart rate ≤ 140 per minute, the presence of arrhythmias and doses of vasopressors (high > 0.2 mcg (kg/min)

5. Airway (A): history of difficult airway and leak test results > 40%.

6. Clinical Component (+): Additional clinical factors, such as the presence of effective cough, management of secretions, the patient's neurological status, resolution of the underlying cause of intubation, and the manifestation of clinical signs of respiratory distress, as well were considered.

After extubation, several indicators will be carefully monitored to assess the patient's recovery and stability. The presence of dysphonia at 72 hours, maximum mobility measured by JH-HLM at 24 hours, and grip strength within the same period will be recorded. Additionally, the need for non-invasive mechanical ventilation at 48 hours will be evaluated, indicating whether it is required or not. The possible presence of dysphagia at 72 hours will also be documented, along with the timing of oral intake initiation, classified into two options: 1) between 12 and 24 hours, and 2) after 24 hours. The occurrence of extubation failure at 48 hours will be recorded, indicating whether

it occurred or not, and muscle strength will be measured using the MRC Scale at 24 hours to assess the patient's functional capacity after the extubation procedure.

After the observation phase, essential data related to the patient's functionality and hospital stay will be collected. The Barthel Index at hospital discharge, cognitive function measured by the Montreal Cognitive Assessment (MOCA), and the quick sensitivity and dexterity test will be recorded, providing a detailed measure of the functional and cognitive capacity achieved. Additionally, the days of hospital stay and the length of stay in the Intensive Care Unit (ICU) will be documented, offering insights into the duration of the recovery process. Mortality will be evaluated, indicating whether it occurred during the observation period. Furthermore, functional independence will be measured using Activities of Daily Living (ADLs) at hospital discharge and ICU discharge, providing a comprehensive assessment of the impact of care and procedures on the patient's autonomy (see Annex 3 and 4).

Within the protocol, the inclusion of safety variables such as blood pressure alteration, cardiac arrhythmias, and oxygen desaturation highlights the importance of continuous monitoring of cardiovascular and respiratory health in patients. These variables serve as key markers to identify potential complications during the intervention or medical treatment. Early detection of blood pressure alterations provides crucial insights into perfusion and circulatory function. Monitoring cardiac arrhythmias aims to identify irregularities in heart rhythm while observing oxygen desaturation alerts to potential issues in tissue oxygenation. These safety measures, integrated into the protocol, contribute to a comprehensive evaluation and proactive management of any changes in the patient's condition, thereby supporting safety and well-being during the clinical process.

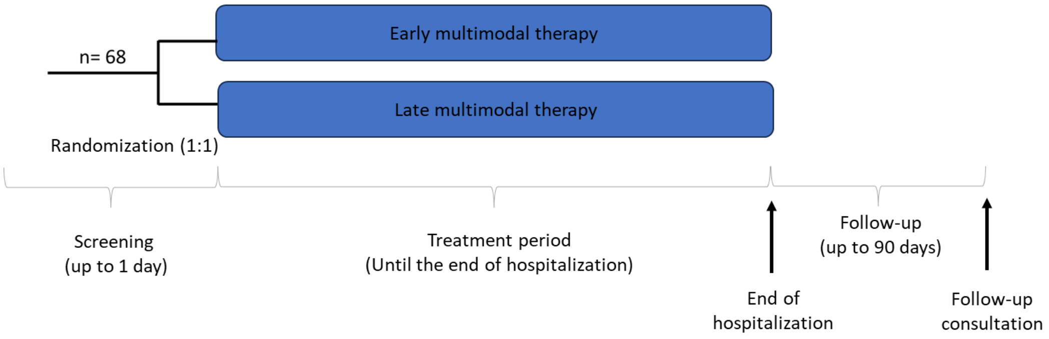

Figure 1. Study design diagram

Discontinuation of therapy

The intervention will be interrupted in any of the following clinical contexts:

• The patient leaves the ICU before completing 24 hours with invasive ventilatory support through an orotracheal tube.

• Death of the patient during their stay in the ICU (up to 28 days)

• The treating physician determines that considering the best interest of the patient it is best to discontinue her participation in the study

• Withdrawal of informed consent by the patient or legal representative

Following the interruption of treatment, the medical management of the patient will continue with the standardized treatments and therapies of the intensive care unit. Treatment may be temporarily interrupted due to medical reasons, which include but are not limited to surgical procedures, radiology studies, and hemodynamic stabilization of the patient. Multimodal therapy will be resumed as soon as possible.

6.8 Loses and Handling of Missing data

• Frequency and Distribution: Losses during follow-up were recorded for participants in both study groups. Of [total number of participants] initially assigned, [number of participants lost] were lost during the study period.

• Reasons for Losses:

o Voluntary withdrawal of consent by the participant.

o Adverse events not related to the study treatment.

o Failure to comply with the study protocol.

o Transfer to other medical facilities.

• Comparison between Groups: A comparative analysis will be carried out between the control and intervention groups, to assess the equitable distribution of losses.

• Characteristics of Lost Participants: The baseline demographic and clinical characteristics of participants who completed the study will be compared with those who were lost to follow, to assess whether the losses introduced substantial biases.

• Impact on Analysis: A sensitivity analysis will be performed to evaluate the potential impact of losses on the study results. Results will be presented with both the inclusion and exclusion of missing participants.

• Statistical Management: In the main statistical analysis, the intention-to-treat approach will be used, including all participants according to their original assignment, regardless of their compliance or loss to follow-up. We will also perform a sensitivity analysis based on a per-protocol analysis since it could provide us with a measure of the effect of weaning under ideal treatment adherence conditions.

• Replacement of Lost Participants:

o Ethical Consideration: Before considering the replacement of lost participants, an ethical evaluation of the justification for such action will be conducted. It will be considered whether the replacement is ethically acceptable and whether its implementation respects the rights and well-being of the participants.

o Imputation Methods: If replacement of lost participants is considered, statistically sound and well-founded imputation methods will be used. Options such as imputation by the mean, LOCF, or other more advanced methods such as multiple imputation will be explored.

o Transparency and Documentation: A detailed description of the imputation methods used will be provided, including the reasons for choosing a specific method. This will be included in the final reports and communication with the Ethics Committee.

o Report to the Ethics Committee: The report to the Ethics Committee will describe in detail any participant losses and any decisions related to the replacement of missing data. Transparency in loss management and consistency with the original study protocol will be emphasized.

o Expert Consideration: The opinion of experts in statistics and research ethics will be sought to ensure that any decisions related to loss management and replacement of lost participants are informed and ethical.

6.9. Protocol Adherence Criteria

Adherence to the protocol will be evaluated in several crucial aspects and will be categorized as high or low. Patients with low adherence to the protocol will be excluded from the sensitivity analysis by intention protocol but will be included in the analysis by intention to treat.

• Patient withdrawal: Low adherence will be considered when a patient voluntarily requests withdrawal of consent, experiences an adverse event unrelated to the study treatment, or is transferred to other medical facilities.

• Non-compliance with the study protocol: It will be classified as low adherence when a patient cannot receive 80% of the interventions required according to the protocol.

6.10. Follow-up and Safety Procedures

• **Lack of Significant Difference in the Duration of Mechanical Ventilation:** If at the end of the study and after analysis of the data, there is no statistically significant difference in the mean duration of mechanical ventilation between the early therapy group and the late therapy group, the trial could be considered a failure in terms of demonstrating the effectiveness of early therapy in reducing days on mechanical ventilation.

• **High Rates of Protocol Deviation:** If more than 20% of participants in one or both groups do not adequately comply with the study protocol, this could affect the validity of the results and lead to trial failure.

• **Safety Concerns or Unacceptable Toxicity:** If an unexpectedly high rate of serious adverse events is observed in the early versus late therapy group, the trial could be considered a failure due to safety concerns.

• **Low Participant Recruitment or Retention Rate.**

• **External Interference or Changes in Clinical Practice:** If significant changes in standard clinical practice occur during the study that affect the interpretation of the results, the trial could be considered a failure. For example, if a new treatment or procedure is introduced that impacts the duration of mechanical ventilation regardless of the therapy assigned in the trial.

6.11. Data Collection

Variable description:

Variable	Definition	Variable type	Scale/Unit of measurement or categories
Age	Years old	Quantitative<br>Discreet	Years
Weight	Weight	Quantitative<br>Keep going	Weight in kilograms
Sex	Sex	Qualitative Nominal	Female or male
Ethnicity	Ethnicity with which the patient identifies	Qualitative Nominal	Afro-Colombian<br>ROM<br>Indigenous<br>Raizal<br>None
Dialysis	Need for renal replacement therapy	Binomial Categorical	0: No<br>1: Yes
Norepinephrine support	Need for norepinephrine support	Binomial Categorical	0: No<br>1: Yes
Maximum dose of norepinephrine	Dose in mcg/kg/min	Quantitative continues	Mcg /kg/min
Inotropic support	Need for inotropic support	Binomial Categorical	0: No<br>1: Yes

Vasopressin support	Need for norepinephrine support	Binomial Categorical	0: No<br>1: Yes
Vasopressin dosage	Dose in IU/min	Quantitative continues	IU/min
Barthel index	The Barthel Index is a scale used to measure a person's degree of functional independence in activities of daily living (ADL).<br>Two RASS assessment points -3 or -2, RASS 0, and at ICU discharge	Discrete Quantitative	It will be 0 if the patient dies.
body mass index	It is the relationship between the patient's weight and height	Quantitative<br>Keep going	Kilograms/Square meter
Cluster	The group to which the patient was assigned	Qualitative Nominal	1: early<br>2: late
Diagnosis	Principal diagnostic	Qualitative Nominal	Diagnostic code CI10.
Protocol Adherence	Degree of adherence to the protocol	Binomial Categorical	1: low<br>2: high
Type of respiratory failure	Development of hypoxemic ($PaO2<60$ mmHg) or hypercapnic ($PCO2>45$mmHg) respiratory failure	Binomial Categorical	1: hypoxemic<br>2: hypercapnic

Patient type	Main pathology that led to the need for invasive mechanical ventilation.	Qualitative Nominal	1 = surgical<br>2= neurological<br>3= septic/respiratory<br>4=cardiovascular<br>5=burned<br>6= respiratory
Sequential Organ Failure Assessment Score<br>(COUCH)	System for evaluating the appearance and evolution of Multiorgan Failure in ICU patients	Discrete Quantitative	Number
Acute Physiology and Chronic Health disease Classification System II<br>(APACHE II)	Physiological classification of acute and chronic diseases	Discrete Quantitative	Number
Simplified Acute Physiology Score II<br>(SAPS II)	disease severity assessment system	Discrete Quantitative	Number
Tidal volume	Amount of volume delivered with each breath	Continuous quantitative	Number
Breathing frequency	Breathing frequency	Discrete Quantitative	Number
End expiratory pressure (PEEP)	End expiratory pressure (PEEP)	Continuous quantitative	Number

Plateau pressure	Equivalent to alveolar pressure in the absence of flow	Quantitative Continued.	Centimeters of H2O
Driving pressure	Driving pressure	Continuous Quantitative	Centimeters of H2O
Static compliance	Volume change divided by pressure change	Continuous Quantitative	ml/cmH20
Airway resistance	the resistance of the respiratory tract to airflow during inspiration and expiration	Continuous Quantitative	Number
Post-intubation PaO2/FIO2 index	Pulmonary oxygenation and FIO2 relationship	Continuous Quantitative	Number
Pneumotaponator pressure start IMV, start ventilatory assistance and weaning	Adjustment of pneumatic tamponade balloon to tracheal perfusion	Continuous Quantitative	Centimeters of H2O
Delta POCC initiation of inspiratory effort and weaning	Non-voluntary inspiratory effort	Continuous Quantitative	Centimeters of H2O
Muscle pressure in weaning (Pmus)	Voluntary Force Magnitude	Continuous Quantitative	Centimeters of H2O
P0.1	Neural respiratory drive	Continuous Quantitative	Centimeters of H2O

Mobilization of secretions	Mobilization of secretions in the orotracheal tube	Qualitative Nominal	**YES:** Assessment by a respiratory therapist of the presence of abundant tracheobronchial secretions<br>**NO**: Subjective assessment by a respiratory therapist of the absence of abundant tracheobronchial secretions
Asynchrony index	Number of asynchronies witnessed in each time	Discrete Quantitative	Percentage
NIF in weaning measured by respiratory therapy	Maximum negative inspiratory effort	Quantitative Continuous	Centimeters of H2O
NIF in weaning measured by physiotherapy	Maximum negative inspiratory effort	Quantitative Continuous	Centimeters of H2O
% LEAK in weaning	% loss of tidal volume when deflating the pneumotaponator	Discrete Quantitative	Percentage
Peak cough flow	Peak expiratory flow during a cough effort attempt	Continuous Quantitative	maximum expiratory flow measurement in flow-time curve L/min
Diaphragmatic excursion measurement	It is the set of diaphragmatic forces combined with adequate intrathoracic and intra-abdominal pressures.	Continuous Quantitative	Centimeters

Diaphragmatic thickness % measurement	Diaphragm thickness	Continuous Quantitative	TF= (TEI- TEE)/ TEE<br>Millimeters
Days necessary death	Days necessary death	Discrete Quantitative	Number
Days to start ventilatory support	Days that were necessary from admission to the intensive care unit and the use of invasive mechanical ventilation	Discrete Quantitative	Days
Days from the start of ventilatory support to the start of weaning	Days from the start of ventilatory support (intubation) to the start of weaning	Discrete Quantitative	Days
Days of mechanical ventilation	Days of invasive mechanical ventilation	Discrete Quantitative	Days
Days free of mechanical ventilation	28 days minus ventilation days	Discrete Quantitative	Days<br>It will be defined as 0 if the patient dies or has been ventilated for more than 28 days.
Delirium	For a patient to be considered to have delirium according to the CAM-ICU, both the first criterion (acute alteration in mental status) and at least one of the other three criteria (inability to	Qualitative Nominal	1. Acute alteration of mental status (+/-).<br>2. Inability to maintain sustained attention (+/-).<br>3. Disorganization of thought (+/-).

	maintain sustained attention, disorganization of thought, or fluctuating changes in level of consciousness).		4. Fluctuating changes in level of consciousness (+/-)
Days of delirium	Days with the presence of delirium	Discrete Quantitative	Days
Sedation days	Days with sedation	Discrete Quantitative	Days
Sedation-free days	28 days fewer days of sedation	Discrete Quantitative	Days<br>It will be defined as 0 if the patient dies.
Barthel index at hospital discharge	The Barthel Index is a scale used to measure a person's degree of functional independence in activities of daily living (ADL).	Discrete Quantitative	It will be 0 if the patient dies.
Days of hospital stay	Days of hospital stay	Discrete Quantitative	Days
Days of ICU stay	Days of ICU stay	Discrete Quantitative	Days
Mortality	Death	Qualitative Nominal	Yes or no
Time from intubation to start of therapy	Time from intubation to start of therapy	Discrete Quantitative	Days

Muscle strength with the medical research scale council	Evaluation of muscle strength in critically ill patients	Qualitative Ordinal	5, normal: full movement against full resistance<br>4+, good (+): full movement against gravity and strong resistance<br>4, good (–): full movement against gravity and minimal resistance<br>3+, regular (+): full movement against gravity only<br>3, regular (-): partial movement only against gravity<br>2, sparse: movement eliminated gravity<br>1 minimal: visible muscle contraction without movement<br>0 absent: total paralysis.
Dysphagia 72 hours after extubation	Dysphagia 72 hours after extubation	Qualitative Nominal	Yes or no
Oral start time.	Time in which the oral route is started after extubation.	Qualitative Nominal	1: 12-24 hours<br>2: > 24 hours
Healthcare-associated pneumonia	The patient developed healthcare-associated pneumonia during his stay in the ICU.	Nominal qualitative	0 = No<br>1 = Yes

Maximum mobility scale at 24 hours.	Maximum mobility measured by the Johns Hopkins scale (JH-HLM)	Qualitative Ordinal	8: Walk more than 250 feet.<br>7: You walk more than 25 feet<br>6: walk more than 10 steps.<br>5: Stands for more than a minute.<br>4: can be moved to the chair.<br>3: may be sitting on the edge of the bed.<br>2: can turn over in bed.<br>1: He can only be lying down.<br><br>It will be 0 if the patient dies.
Manual dexterity and psychomotor processing speed	The evaluated person makes 20 180° turns to the new model Colombian coin of 200 pesos; (similar to that of 25 US cents) as quickly as possible, using first, second, and third fingers only, the person is expected to perform a minimum of 15 seconds in the dominant hand and 19 seconds in the non-dominant hand<br>Coin rotation test	Qualitative Nominal	0: Integrate<br>1: Altered
Assessment of Sensory Integrity	Screening of sensitivity (superficial and deep) is	Qualitative Nominal	0: Integrate<br>1: Altered

	carried out through the assessment of cutaneous or tactile sensation, proprioception, and stereognosis (shape, size, and texture) in terms of identification, discrimination, and location of the stimulus.<br><br>Sensitivity screening suggested by the literature		
Qualitative assessment of cognitive processing	Through standardized evaluation, in a directed activity that evaluates domains of isospatial/executive vision, naming, attention, language, memory, abstraction, and orientation.<br><br>The version that corresponds to the patient will be used like this:<br>Standard: for people with high school education levels and above.<br>• Basic: for people with illiteracy or low education<br>Blind: for people with significant impairment of visual function	Qualitative Nominal	0: Normal<br>1: Altered

	processing assessment (Montreal Cognitive Assessment)		
Quantitative assessment of cognitive processing	Through standardized evaluation, in a directed activity that evaluates domains of isospatial/executive vision, naming, attention, language, memory, abstraction, and orientation.<br><br>The version that corresponds to the patient will be used like this:<br>Standard: for people with high school education levels and above.<br>• Basic: for people with illiteracy or low education<br>Blind: for people with significant impairment of visual function<br>Cognitive Processing Assessment (Montreal Cognitive Assessment)	Discrete Quantitative	Numeric
Evaluation of functionality in daily activities	The patient's level of autonomy and independence will be assessed in basic daily activities of daily living, determining the level of functionality in these, supported by direct observation, clinical	Qualitative Ordinal	7: Complete independence<br>6: Modified Independence<br>5: Supervised<br>4: Minimum attendance<br>3: Moderate assistance<br>2: Maximum assistance

	history, and reference from nursing staff and family/caregiver.<br>Functional Independence Measure (FIM).		1: Full attendance
Grip strength at the time of extubation	Grip strength of the dominant hand within 24 hours after extubation	Continuous Quantitative	Kilograms<br>It will be 0 if the patient dies.
Failed extubation	Inability to breathe spontaneously in the first 48 hours after removal of the artificial airway and the patient is unable to maintain a patent airway	Qualitative Nominal	Yes or no
NIV POS extubation	Post-extubation non-invasive ventilatory support within 48 hours	Qualitative Nominal	NOT = 0<br>IF = 1
Prolonged mechanical ventilation	Invasive mechanical ventilation longer than 14 days	Qualitative Nominal	Yes or no
Tracheostomy	Need to use a tracheostomy tube.	Qualitative Nominal	Yes or no
Adult respiratory distress syndrome	Bilateral alveolar infiltrates with a PAFI less than 300.	Qualitative Nominal	Yes or no

Dysphonia at 72 hours.	Presence of dysphonia 72 hours after extubation.	Qualitative Nominal	0: No<br>1: Yes
**OMAHA+**	The use of the OMAHA+ Scale during the process of withdrawing ventilatory support to make the extubation decision	Qualitative Nominal	NOT = 0<br>Yes = 1
Alteration in blood pressure	The patient experiences significant changes in blood pressure during the therapies	Nominal qualitative	0 = No<br>1 = Hypotension without the requirement for medical management<br>2 = Hypotension requiring medical management<br>3 = Hypertension without requiring medical management<br>4 = Hypertension requiring medical management
Cardiac arrhythmias	The patient had a cardiac arrhythmia during therapy.	Nominal qualitative	0 = No<br>1 = Cardiac arrhythmia without a requirement for medical management<br>2 = Cardiac arrhythmia requiring medical management
oxygen desaturation	The patient became desaturated during therapy	Nominal qualitative	0 = No<br>1 = Oxygen desaturation without requirement for medical management

			2 = Oxygen desaturation requiring medical management
Pain or agitation	The patient experienced pain or agitation during therapy.	Nominal qualitative	0 = No<br>1 = Pain or agitation without requiring medical management<br>2 = Pain or agitation requiring medical management
Invasive line removal	The patient accidentally or unscheduled removed an invasive line during therapy	Nominal qualitative	0 = No<br>1 = Yes
tachypnea	The patient presented tachypnea during therapy.	Nominal qualitative	0 = No<br>1=Tachypnea without the requirement for medical management<br>2=Tachypnea requiring medical management
Neurological impairment	The patient presented with neurological deterioration during therapy.	Nominal qualitative	0 = No<br>1=Neurological deterioration without the requirement for medical management<br>2=Neurological deterioration requiring medical management
Unscheduled extubation	The patient was extubated unscheduled during the ICU stay.	Nominal qualitative	0 = No<br>1 = Yes

Bronchoaspiration	The patient aspirated bronchodilators during the ICU stay	Nominal qualitative	0 = No<br>1 = Yes

Procedures for Data Collection

Data will be collected by clinical staff, therapists, and research assistants from the intensive care unit, all of whom are familiar with the study and the predefined variables. The interventions and variables collected by the therapists are detailed in the Clinical Trial Operational Plan (Annex 1). Data collection will take place on the third and fourth floors of the Intensive Care Unit at FSFB. Training will be provided to all investigators on data collection and the specific times to complete the data collection instrument. Additionally, knowledge of the protocol will be assessed through an exam, which will be considered passed with a score above 4, and feedback will be provided to address any deficiencies. The variables measured by the therapists will be recorded daily from the start of the intervention. Measurements will be taken at five key points in the research: upon admission, during observation in the intensive care unit, at the start of weaning from ventilatory support, before and after extubation, and at the end of observation. Finally, a follow-up will be conducted 90 days after endotracheal intubation, during which patients or their legal representatives will be contacted by phone to assess mortality.

To ensure data quality, the following strategies will be implemented:

Data Validation During Entry: Verification of formats, ranges, and logical consistency of the data will be performed at the time of entry.

Double Data Entry: Two investigators will independently enter the same data, and then compare them to identify discrepancies. Any discrepancies found will be investigated and corrected.

Consistency and Coherence Verification: Checks will be carried out to ensure that the data do not present contradictory or incoherent information in the records.

Extreme Value Checking: Extreme or atypical values in the data will be identified and verified to ensure their validity.

Quality Control in Later Stages: Cross-checks will be performed, random samples of the results will be reviewed, and external validation methods will be used whenever possible.

These strategies will be implemented to ensure the quality and reliability of the data collected in the study.

Data collection instrument

The clinical investigators will be responsible for collecting the information in RedCap (see Annex 5). RedCap provides a secure environment for the storage and management of clinical data, ensuring the privacy and confidentiality of the information. This transfer to RedCap will allow for better organization and access to the data, facilitating its analysis and subsequent use in the study.

6.12. Data quality: error and bias

Allocation Bias: This occurs when there is an inappropriate or biased selection of participants for the treatment and control groups. To minimize this bias, random allocation is used to ensure that participants are equitably assigned to the different groups.

Blinding Bias: This can occur when participants, researchers, or evaluators know the treatment assignment, which can influence their behavior or the evaluation of outcomes. Randomized Controlled Trials (RCTs) often use blinded random allocation, where participants and/or researchers do not know the treatment assignment. However, given the characteristics of the protocol, blinding is impossible.

Exclusion Bias: This can occur when certain participants are selectively excluded from the study, which can bias the results. This bias will be managed by appropriately using inclusion and exclusion criteria.

Loss to Follow-Up Bias: This occurs when participants drop out of the study or are lost to follow-up unequally between the treatment and control groups. Unequal loss of participants can affect the validity of the results. To minimize this bias, efforts will be made to maximize participant retention and analyze the results according to the "intention-to-treat" principle.

Reporting Bias: This occurs when the results selected or reported in the study are influenced by the researchers' preferences or the intention to present the results favorably. To avoid this bias, the study protocol will be registered at www.clinicaltrials.gov and published in an indexed journal.

6.13. Statistical Analysis Plan

A Shapiro-Wilk test will be conducted to verify the distribution of quantitative variables, considering the number of patients included. Data will be presented as means or medians, accompanied by their standard deviation or interquartile range, as appropriate. Categorical variables will be described as absolute and relative frequencies.

To compare continuous variables between the two groups (early vs. late therapy), a T-test or the Mann-Whitney U test will be employed, depending on the distribution of the data. For categorical variables, a $\chi 2$ test or Fisher's exact test will be used, as appropriate.

The primary objective of the study is to evaluate the association between the use of early multimodal therapy and the days of invasive mechanical ventilation. To address this question, we will perform a survival analysis and a Cox regression. A risk variable will be created by combining the time of invasive mechanical ventilation and extubation (yes/no). Then, a univariable Cox regression analysis will be performed with the risk variable as the dependent variable and the intervention group (early vs. late) as the independent variable. Additionally, a survival curve will be generated, and differences between the two groups will be analyzed using a log-rank test.

To evaluate factors related to the time of mechanical ventilation, univariable Cox regression models will be performed for all variables included in the study. To determine whether continuous variables can be evaluated continuously or as categorical variables, the assumption of linearity will be checked through the Martingale residual plot. If linearity is not met, the variable will be stratified, and cut-off points will be defined according to the appearance of the plot. Subsequently, those variables with a statistically significant p-value (less than 0.25) and clinically important variables (age, sex, delirium, etc.) will be included in a multivariable Cox regression model. The best model will be selected through a Backward Stepwise process based on Akaike (AIC) and Bayesian (BIC) information criteria.

Variables that could act as confounding factors will be evaluated through a second multiple regression model. In this model, the potentially confounding variable will be removed, and the resulting slope or estimator (adjusted estimate) will be calculated. If there is a change greater than 15%-20%, the confounding variable will be included in the final model. Otherwise, the most parsimonious model will be chosen.

To evaluate the interaction between two variables, another regression model will be performed, including a new variable resulting from multiplying the two potentially interacting variables. If the regression coefficient of this new variable has a significance level of less than 0.05, it is concluded that there is an interaction, and this variable will be included in the model. Variables with potential interaction will be identified by evaluating confounding factors. Researchers will also evaluate clinical variables that may be considered as interaction or confounding variables through the above-described strategy.

The assumption of proportional hazard will be evaluated using Schoenfeld residuals, and influential values will be analyzed through residuals versus observations plots. Multicollinearity will be assessed through matrix curves.

Logistic regression models will be used to assess the relationship between our dichotomous variables, such as failed extubation, post-extubation NIV, prolonged mechanical ventilation, tracheostomy, and dysphonia, and the other studied variables. The assumptions of a logistic regression model, such as linearity in the log of the odds, independence of errors, homogeneity of variance, absence of multicollinearity, absence of outliers, and proportionality of effects, will be evaluated through residual analysis and graphical diagnostics, such as Pearson residual analysis or deviance residual analysis.

A multiplicity adjustment strategy will be employed in statistical tests, such as the Bonferroni method, Holm-Bonferroni, or other methods that control the false discovery rate (FDR), to maintain the overall type 1 error rate at an acceptable level.

A detailed exploration will be conducted through a sensitivity analysis of possible interactions between identified subgroups, according to the RASS scale, protocol adherence, and other relevant variables, to deepen the understanding of any joint influence that may affect the study results. If no statistically significant differences are identified through regression analyses, a post-hoc analysis will be conducted to assess whether the recruited sample size was adequate to detect possible differences between the established subgroups. This additional analysis will allow adjusting the interpretation of the results and provide valuable information on the validity of the obtained conclusions.

Finally, an independent data monitoring committee will be established to conduct interim reviews of the sample size, allowing adjustments based on variations in event rates observed during the study, thus ensuring adequate statistical power throughout its execution.

7. Ethical Consideration

This Randomized Clinical Trial will be conducted by international ethical principles, in line with the Declaration of Helsinki of the World Medical Association, at the 29th General Assembly, Tokyo, October 1975, and subsequent amendments, as well as the Bioethics standards for research in Colombia (Resolution No. 8430 of 1993, issued by the Ministry of Health and known as the "Bioethics Code in Research").

According to Colombian Resolution 8430 of 1993, this study is considered research "with greater than minimal risk" since it employs random methods of assignment to therapeutic schemes. Given

this categorization, informed consent will be obtained from the patient, or if the patient is unable to provide informed consent, it will be requested from their legal representative.

Furthermore, the personal data of the study participants will be protected under the confidentiality and personal data treatment clauses by Resolution 1581 of 2012 of Colombian law. All study data will be incorporated into a database after being properly anonymized. The objective of this is to ensure the confidentiality of the included data through coded records, as well as to allow their transfer to statistical analysis software for subsequent study. The project will be evaluated by the Ethics Committee of the Fundación Santa Fe de Bogotá Hospital. The results will be published in academic and scientific journals, preserving their accuracy and referring to global data rather than individual participants.

Since this is a controlled clinical trial, informed consent is required (Annex 2). The clinical investigators will be responsible for the process of explaining and obtaining informed consent after verifying the inclusion and exclusion criteria of potential study participants. In the case of patients with impaired consciousness or who cannot exercise their autonomy, the purpose, intervention, duration, benefits, risks, confidentiality measures, and contact information of the study will be explained to the patient's legal representative. It will also be explained that participation is voluntary and without any detriment to the quality of medical care in case of non-participation or withdrawal of consent at any time during the study.

Principles of Bioethics

Beneficence

The principle of beneficence is applied in this study in two ways:

1. The potential benefits to the patients will be maximized, as both arms will receive multimodal therapy, which has extensive evidence worldwide. Additionally, the randomized clinical trial is the primary study type with the highest scientific rigor, generating the most applicable knowledge for improving current treatments and therapies.
2. The monitoring and supervision of the study ensure that the potential benefits outweigh the risks. Continuous safety evaluations will be conducted so that if any danger to the participants is detected, the study will be halted.

Non-Maleficence: The study is designed to minimize potential dangers or harm to the participants. Participants will be continuously monitored, especially since they are hospitalized patients in the Intensive Care Unit. The study will prioritize patient safety over research outcomes, ensuring that any unnecessary risks are avoided.

Justice: The principle of justice will be guaranteed in two ways:

1. There will be an equitable selection of participants, that is, no group will be discriminated against. Participants will be selected consistently according to the inclusion and exclusion criteria previously described, which have scientific and clinical bases.
2. The study will have an equitable distribution of benefits and risks. Potential benefits and risks will be allocated fairly among participants, meaning that access to the therapies or resulting knowledge will not be unfairly limited and that the costs and risks of the research will not fall disproportionately on a specific population.

Autonomy: The principle of autonomy is ensured in this study through the process of voluntary informed consent by the participants. Potential participants will be fully informed regarding the purpose, objectives, intervention, duration, benefits, risks, alternatives, confidentiality measures, and contact information of the study. They, in their autonomy, have the right to accept or decline their participation in the study without being coerced.

Publication: We are committed to the complete and transparent publication of all obtained results, both positive and negative. This practice aligns with our dedication to scientific integrity and the advancement of knowledge in the medical field. Every finding, regardless of its nature, will be documented and shared with the scientific community and the public, thus fulfilling our commitment to contribute to the global body of medical knowledge.

8. Scope

This proposed study has the potential to significantly strengthen the scientific and medical community in multiple aspects. The planned research not only aims to generate new knowledge about therapy and strategies for shortening mechanical ventilation but also seeks to establish innovative methodologies that can be applied in future research. Additionally, by focusing on rehabilitation, this protocol has the potential to improve current clinical practices and offer new perspectives. It is important to highlight that this study will foster interdisciplinary collaboration by involving researchers and professionals from various fields, promoting an exchange of ideas and approaches that will enrich the field.

The results of this research are expected to be presented at national and international conferences, providing an opportunity to share the findings with the scientific community and healthcare professionals. This dissemination will allow for discussion and feedback on the obtained results.

Furthermore, efforts will be made to publish the results in high-impact journals, preferably classified as Q1 or Q2. These journals are recognized for their rigorous peer-review process and their prestige

in the academic and scientific community. Publishing in such journals will provide greater visibility and reach for the research results, making them accessible to other researchers and healthcare professionals worldwide.

The objective of sharing the results in conferences and scientific publications is to promote the dissemination of knowledge, contribute to the advancement of research in the field, and enable the findings of this study to be used to improve patient care and treatment.

9. Researchers´ background

Laura María Castillo is a specialist in critical care medicine from the Universidad del Rosario. She currently serves as an intensivist in the Department of Critical Care Medicine at the Fundación Santa Fe de Bogotá, where she is the head of the sepsis and ventilation section of the institutional ICU, with studies related to the field.

Jorge Iván Alvarado Sánchez is an anesthesiologist with a master's degree in physiology, whose thesis (relationship between pulse pressure variability/stroke volume variability in a porcine endotoxin shock model) was awarded honors by the Universidad Nacional de Colombia. He has several studies published in high-impact medical journals such as Critical Care, Shock, Annals of Intensive Care, Scientific Reports, Journal of Intensive Care Medicine, Revista Colombiana De Anestesiología, and Acta Colombiana De Cuidado Intensivo. He has also been a peer reviewer for several high-impact anesthesiology and critical care journals, including Critical Care, Scientific Reports, Journal of Clinical Monitoring and Computing, Intensive Care Medicine Experimental, BMC Anesthesiology, BMJ Open, Anaesthesiology Intensive Therapy, among others.

Dr. Alvarado's career can be reviewed at the following:

- Links:https://orcid.org/0000-0003-4320-3150.
- https://scienti.minciencias.gov.co/cvlac/visualizador/generarCurriculoCv.do?cod_rh=0002095674#.

10. Activity Schedule

Activities	Month1	Month2	Month3	Month4	Month5	Month6	Month7
**Project Approach**	x						
**Submission Subdirectorate clinical studies**		x					
**Ethics committee approval**		x					
**Patient Registration**			x	x	x	x	
**Statistical analysis and results**						x	
**Writing scientific article**						x	x

11. Budget and Funding

This research has not received, does not receive, and will not receive funding from any agency, institution, or sponsor in the public, commercial, or non-profit sectors.

ITEMS	FUNDING SOURCE	TOTAL
Advisory Fees	Own funds	$5000000

Additional software licenses	FSFB	$3000000
Administrative	Own funds	$2000000
Publication	Own funds	$1000000
TOTAL		$11000000

12. References

1. Plowman EK, Anderson A, York JD, DiBiase L, Vasilopoulos T, Arnaoutakis G, et al. Dysphagia after cardiac surgery: Prevalence, risk factors, and associated outcomes. Journal of Thoracic and Cardiovascular Surgery. 2021 Feb 1;165(2):737-746.e3.
2. Rousseau AF, Prescott HC, Brett SJ, Weiss B, Azoulay E, Creteur J, et al. Long-term outcomes after critical illness: recent insights. Crit Care. 2021 Dec 1;25(1).
3. Sanger H. How early is early? When should rehabilitation begin in critical illness? ACPRC Journal. 2020 Jun;52.
4. Hodgson CL, Schaller SJ, Nydahl P, Timenetsky KT, Needham DM. Ten strategies to optimize early mobilization and rehabilitation in intensive care. Vol. 25, Critical Care. BioMed Central Ltd; 2021.
5. Zhang L, Hu W, Cai Z, Liu J, Wu J, Deng Y, et al. Early mobilization of critically ill patients in the intensive care unit: A systematic review and meta-analysis. PLoS One. 2019 Oct 1;14(10).
6. Hodgson C, Bailey M, Bellomo R, Brickell K, Broadley T, Buhr H, et al. Early Active Mobilization during Mechanical Ventilation in the ICU. New England Journal of Medicine [Internet]. 2022 Nov 10;387(19):1747–58. Available from: http://www.nejm.org/doi/10.1056/NEJMoa2209083
7. Schaller SJ, Anstey M, Blobner M, Edrich T, Grabitz SD, Gradwohl-Matis I, et al. Early, goal-directed mobilisation in the surgical intensive care unit: a randomised controlled trial. The Lancet. 2016 Oct 1;388(10052):1377–88.

8. Koestenberger M, Neuwersch S, Hoefner E, Breschan C, Weissmann H, Stettner H, et al. A Pilot Study of Pharyngeal Electrical Stimulation for Orally Intubated ICU Patients with Dysphagia. Neurocrit Care. 2020 Apr 1;32(2):532–8.

9. See KC, Peng SY, Phua J, Sum CL, Concepcion J. Nurse-performed screening for postextubation dysphagia: A retrospective cohort study in critically ill medical patients. Crit Care. 2016 Oct 12;20(1).

10. Zuercher P, Moret CS, Dziewas R, Schefold JC. Dysphagia in the intensive care unit: Epidemiology, mechanisms, and clinical management. Vol. 23, Critical Care. BioMed Central Ltd.; 2019.

11. Zuercher P, Dziewas R, Schefold JC. Dysphagia in the intensive care unit: a (multidisciplinary) call to action. Vol. 46, Intensive Care Medicine. Springer; 2020. p. 554–6.

12. Schefold JC, Berger D, Zürcher P, Lensch M, Perren A, Jakob SM, et al. Dysphagia in mechanically ventilated ICU patients (Dynamics): A prospective observational trial. Crit Care Med. 2017;45(12):2061–9.

13. Troll C, Trapl-Grundschober M, Teuschl Y, Cerrito A, Compte MG, Siegemund M. A bedside swallowing screen for the identification of post-extubation dysphagia on the intensive care unit – validation of the Gugging Swallowing Screen (GUSS)—ICU. BMC Anesthesiol. 2023 Dec 1;23(1).

14. Borders JC, Fink D, Levitt JE, McKeehan J, McNally E, Rubio A, et al. Relationship Between Laryngeal Sensation, Length of Intubation, and Aspiration in Patients with Acute Respiratory Failure. Dysphagia. 2019 Aug 15;34(4):521–8.

15. Su H, Hsiao TY, Ku SC, Wang TG, Lee JJ, Tzeng WC, et al. Tongue Weakness and Somatosensory Disturbance Following Oral Endotracheal Extubation. Dysphagia. 2015 Apr 28;30(2):188–95.

16. Brodsky MB, Pandian V, Needham DM. Post-extubation dysphagia: a problem needing multidisciplinary efforts. Intensive Care Med. 2020 Jan 1;46(1):93–6.

17. Houzé MH, Deye N, Mateo J, Mégarbane B, Bizouard F, Baud FJ, et al. Predictors of extubation failure related to aspiration and/or excessive upper airway secretions. Respir Care. 2020 Apr 1;65(4):475–81.

18. McRae J, Montgomery E, Garstang Z, Cleary E. The role of speech and language therapists in the intensive care unit. J Intensive Care Soc. 2020 Nov 1;21(4):344–8.

19. Omura K, Komine A, Yanagigawa M, Chiba N, Osada M. Frequency and outcome of post-extubation dysphagia using nurse-performed swallowing screening protocol. Nurs Crit Care. 2019 Mar 1;24(2):70–5.

20. Hodgson CL, Stiller K, Needham DM, Tipping CJ, Harrold M, Baldwin CE, et al. Expert consensus and recommendations on safety criteria for active mobilization of mechanically ventilated critically ill adults. Crit Care. 2014 Dec 4;18(6).

21. Deemer K, Myhre B, Oviatt S, Parsons M, Watson M, Zjadewicz K, et al. Occupational therapist-guided cognitive interventions in critically ill patients: a feasibility randomized controlled trial. Canadian Journal of Anesthesia [Internet]. 2023;70:139–50. Available from: https://doi.org/10.1007/s12630-

22. Twose P, Jones U, Bharal M, Bruce J, Firshman P, Highfield J, et al. Exploration of therapists' views of practice within critical care. BMJ Open Respir Res. 2021 Nov 8;8(1).

23. Prohaska CC, Sottile PD, Nordon-Craft A, Gallagher MD, Burnham EL, Clark BJ, et al. Patterns of utilization and effects of hospital-specific factors on physical, occupational, and speech therapy for critically ill patients with acute respiratory failure in the USA: Results of a 5-year sample. Crit Care. 2019 May 16;23(1).

24. Clarissa C, Salisbury L, Rodgers S, Kean S. Early mobilisation in mechanically ventilated patients: A systematic integrative review of definitions and activities. Vol. 7, Journal of Intensive Care. BioMed Central Ltd.; 2019.

25. Hickmann CE, Castanares-Zapatero D, Bialais E, Dugernier J, Tordeur A, Colmant L, et al. Teamwork enables high level of early mobilization in critically ill patients. Ann Intensive Care. 2016 Dec 1;6(1).

26. Van Willigen Z, Collings N, Richardson D, Cusack R. Quality improvement: The delivery of true early mobilisation in an intensive care unit. BMJ Qualitive Improvement Programme [Internet]. 2016; Available from: http://bmjopenquality.bmj.com/

27. Miranda Rocha AR, Martinez BP, Maldaner da Silva VZ, Forgiarini Junior LA. Early mobilization: Why, what for and how? Vol. 41, Medicina Intensiva. Ediciones Doyma, S.L.; 2017. p. 429–36.

28. Hodgson CL, Capell E, Tipping CJ. Early Mobilization of Patients in Intensive Care: Organization, Communication and Safety Factors that Influence Translation into Clinical Practice. Vol. 22, Critical Care. BioMed Central Ltd.; 2018.

29. Lynch YT, Clark BJ, Macht M, White SD, Taylor H, Wimbish T, et al. The accuracy of the bedside swallowing evaluation for detecting aspiration in survivors of acute respiratory failure. J Crit Care. 2017 Jun 1;39:143–8.

30. Dziewas R, Stellato R, van der Tweel I, Walther E, Werner CJ, Braun T, et al. Pharyngeal electrical stimulation for early decannulation in tracheotomised patients with neurogenic dysphagia after stroke (PHAST-TRAC): a prospective, single-blinded, randomised trial. Lancet Neurol. 2018 Oct 1;17(10):849–59.

31. Zuercher P, Schenk N V., Moret C, Berger D, Abegglen R, Schefold JC. Risk Factors for Dysphagia in ICU Patients After Invasive Mechanical Ventilation. Chest. 2020 Nov 1;158(5):1983–91.

32. Schweickert WD, Pohlman MC, Pohlman AS, Nigos C, Pawlik AJ, Esbrook CL, et al. Early physical and occupational therapy in mechanically ventilated, critically ill patients: a randomised controlled trial. The Lancet. 2009 May;373(9678):1874–82.

33. Ding N, Zhang Z, Zhang C, Yao L, Yang L, Jiang B, et al. What is the optimum time for initiation of early mobilization in mechanically ventilated patients? A network meta-analysis. PLoS One. 2019 Oct 7;14(10):e0223151.

ANNEX 1

Interventions corresponding to each therapy

Fundación Santa Fe de Bogotá	Fundación Santa Fe de Bogotá<br>Level 1: Diagnostic and Therapeutic Support<br>Level 2: Pulmonology<br>Level 3: Respiratory Therapy<br>Operational Standard: Mechanical Ventilation	**Code**	AMB-TR-EO1-007
		**Date**	2021-01-28
		**Version**	5.0

Strategic	**Missionary**	Support

Objective

- To bring a certain volume of gas to the lungs, so that gas exchange occurs in the alveoli

-To reduce the patient's respiratory effort
-To support respiratory function until the total or partial reversal of the cause

Scope

From the calibration of the equipment (VENTILATOR), installation, and even extubation of the patient, allowing them to breathe on their own.

CONDITIONS/RESOURCES NEEDED

1. CONDITIONS / NECESSARY RESOURCES

Assistant Personnel:

- Intensivist Physician
- Respiratory Therapist
- Intensive Care Units
- Air source
- Oxygen source
- Electrical source
- Ventilator
- Disposable circuit

RESULTADOS ESPERADOS

2. EXPECTED RESULTS

- Maintain appropriate levels of PO2 and PCO2
- Rest respiratory muscles
- Maintain gas Exchange
- Increased arterial oxygenation
- Reduction work of breathing

RISK (AND)/HOW TO NEUTRALIZE THEM)

3. RISK (S)	HOW TO NEUTRALIZE IT (S)
1. Barotrauma.	1. Minimize minute volume<br>Low tidal volumes<br>To limit maximum inflation pressure<br>To reduce bronchial obstruction
2. Pneumonia associated with Mechanical Ventilation	2. Early extubation<br><br>Head of bed at 30 degrees<br>Oral rinses with chlorhexidine<br>Use of bacterial filters closed suction catheters<br>Hand washing
3. Oxygen toxicity<br>4. Air trapping<br>5. Atelectasis	3. Reduce inspired oxygen fraction.<br>Application of PEEP<br><br>4. Reduce bronchial obstruction<br>Decrease minute volume<br>Increase expiratory time<br>Increase tidal volume<br>Increase PEEP

DESCRIPTION OF THE ACTIVITIES

4. DESCRIPTION OF THE ACTIVITIES

1. 1. In the intensive care unit, you must have a reserve ventilator (with a green sticker), confirming its calibration.
2. Calibrate the ventilator with its respective circuit before installing it on the patient.
3. Prepare the equipment with the basic ventilatory parameters and open the mechanical ventilation sheet or form. Note the patient's data and the parameters with which the ventilator is prepared according to the medical order.
4. Place the closed suction system and the bacterial filter. These devices should be replaced every 24 to 48 hours according to the provider's prescription.
5. Connect the ventilator to the patient and verify the patient's connection to the ventilator and the patient's stability. Report and confirm the parameters and the patient's stability with the doctor.
6. Note any changes in the mechanical ventilation form and the clinical history. Measure the pressure of the cuff of the orotracheal tube, nasotracheal tube, or tracheostomy cannula, which should be done every shift and recorded in the

mechanical ventilation form.

7. Every day at the beginning of each shift (morning, afternoon, and night), the screen, keyboard of the ventilator, and surface should be cleaned with an organic chlorine towel (request the supplies from the cleaning operator of each unit). Clean examination gloves should be worn, and the cleaning process should start from top to bottom and from left to right. If necessary, change the towel as many times as needed. Upon completion, dispose of the towel and gloves in the red solid waste bin, perform hand washing, and record the procedure in the ventilator control form.

9. Follow the appropriate technique for removing mechanical ventilation (standard extubation AMB-NEUM 01-014).

NOTE: Ventilators will be disinfected, assembled, checked, and calibrated in the Pulmonology service (basement two). The marking will be done according to the state of the ventilator:
Green Sticker = Ready for use
Yellow Sticker = Not calibrated
Red Sticker = Not suitable for use, damaged, pending technical review All ventilators must be covered with a transparent plastic bag.
Maintain permanent control of the parameters and changes made to the patient's parameters.
All changes or controls must be recorded in the mechanical ventilation control form and the clinical history.

Patient circuits are DISPOSABLE. They are installed on the ventilator, calibrated, and placed on the patient.
Then, they are requested from the pharmacy to replenish the reserve.

The circuit is left for the entire stay of the patient unless it is visibly dirty or with organic material.
Request it from the pharmacy at the patient's expense and dispose of it in the red solid waste bin in the patient's room.
Do not forget to note this change in the mechanical ventilation control form.
Preparations: Prepare supplies and ventilator and calibrate the ventilator.
Recommendations: Intubated or tracheostomized patient.
Controls: Ventilatory parameters are controlled once per shift or if any parameter changes are made and recorded in the ventilator control sheet.

RESPONSIBLE, FREQUENCY AND PLACE

RESPONSIBLE, FREQUENCY AND PLACE
1 – 12 Responsible. Respiratory Therapists<br>Frequency: According to medical<br>order Location: Emergencies, Intensive Care Units Issue

Date

2008-02-01

RESPIRATORY THERAPY COMPONENT

Coordinators:

- Ana Gabriela López Rubio, email: aglopez904@gmail.com
- Diana Carolina Ortiz Moreno, email: carolinao.21@hotmail.com

Justification

The work of the respiratory therapist has grown exponentially in the last decade as a link in the multidisciplinary group of the Intensive Care Unit, being a fundamental element in terms of critical thinking, anticipating events in clinical practice, and being a loyal advisor to the intensive care doctor. When solving problems that are respiratory and ventilatory.

After the events that occurred in the pandemic, the work of the respiratory therapist in the practice of multimodal therapy has optimally impacted daily professional practice, developing protocols in many areas such as conventional oxygen therapy, comprehensive management of the ventilated patient, withdrawal of mechanical ventilator in extubation and treatment of respiratory morbidities which are increasing every day due to environmental pollution (biomass), tobacco use and occupational diseases, allowing a comprehensive care guideline to be provided to these patients, being part of the promotion and prevention of respiratory diseases with education for patients and families.

Being part of the chain that forms multimodal therapy, administering in a rational and controlled manner the oxygen necessary to carry out all the vital processes of the human body, being professional with sensory intelligence, being a diagnostic support, monitoring and rehabilitation of pulmonary mechanics and hemodynamics using the senses, from vision to evaluate the curves of the mechanical ventilator and diagnose obstructive and restrictive pathologies, guiding lung care minute by minute, hearing to find adventitious lung sounds, guiding the treatment to follow, to the application of palpation in chest kinesitherapy, to eliminate secretions lodged in the lung and strengthen the breathing muscles.

Starting from the minute invasive mechanical ventilation is established, passing through the daily course until the day of ventilatory weaning, the success of the variables determining the withdrawal of support depends on strict monitoring of ventilatory mechanics, the prevention of associated pneumonia Mechanical ventilation and the weaning process where spontaneous breathing is recovered and the possibility of removing the ventilator is evaluated, it is vital to monitor the possible causes that may lead to weaning failure.

Diaphragmatic dysfunction and ICU-acquired weakness impact success or failure in weaning from mechanical ventilation according to measurements guided by ultrasonographic evaluation of the diaphragm: excursion and measurement of diaphragmatic thickness; as well as muscle strength according to the MRC scale in a spontaneous breathing test (PVE) for 30 minutes, this is how a study evaluated these variables, finding that weakness acquired in the ICU was directly related to difficult and prolonged weaning, longer stay in ICU and prolongation of mechanical ventilation. (3)

The ventilatory weaning process is a term used to describe the gradual process of decreasing ventilatory support, progressively decreasing parameters until the patient can start breathing with minimal ventilatory support required. There is a classification of ventilatory weaning divided according to PVE: Simple weaning, is the first successful spontaneous breathing test. Difficult weaning failed PVE on the first or third attempt or required PVE after seven days of mechanical ventilation and

prolonged weaning, in which the patient presents successful PVE in a time greater than seven days under ventilatory support.

There are risk factors for extubation failure:

- Failure of two further tests of ventilation spontaneous
- Insufficiency cardiac chronicle
- Cough weak
- Risk of stridor post extubation
- Age > 65 years.
- PaCO2 > Four. Five mmHg later to the extubation. (4)

It has recently been shown that the spontaneous breathing test (SVT) in PSV for 30 minutes resulted in a significantly successful extubation rate relative to the T-Tube test for 2 hours, given that a less demanding test vs. a much longer time may overestimate the inspiratory effort after extubation. (5)

Hemodynamic monitoring is also crucial for extubation success or failure and is the linear relationship between the dose of vasopressor support and the risk of extubation failure. Vasopressor doses > 0.1 mcg/kg/min have been associated with the risk of intubation about fluid balance and filling pressures, since by having low doses of vasopressor support the urinary balance will tend to be negative to avoid overload. Volume and subsequent pulmonary edema. (6)

The leak test of the pneumotaponator cuff in anticipation of the risk of post-extubation stridor which is associated with laryngeal edema is part of the range of necessary tests, given that if the leak test is <45%, the start of corticosteroid therapy is essential. Prior intravenous injection and subsequent repetition of the test to prevent whether the administration of nebulized corticosteroids is necessary. (7)

Thus constituting an irreplaceable role in the multimodal therapy group.

Scope

Selected Patients for the Research Project: "Association between Early Multimodal Therapy and Days of Mechanical Ventilation in the Intensive Care Unit of Fundación Santa Fe de Bogotá: A Randomized Controlled Clinical Trial (SECEC-2023-129)."

Target Population:

Inclusion criteria

- Hospitalized patients in the adult intensive care unit (ICU) of Fundación Santa Fe de Bogotá who require invasive mechanical ventilation via an endotracheal tube for a period greater than 72 hours and are expected to continue for at least 24 hours.
- Patients with a Barthel Index score of $\geq$ 70.

Exclusion Criteria:

- Patients requiring invasive mechanical ventilation through a tracheostomy cannula or nasotracheal tube.
- Patients who have undergone any type of head and neck surgery.
- Cardiac arrest.
- Airway burns.
- Burns cover more than 50% of the body's surface area.
- Patients with chronic obstructive pulmonary disease (COPD).
- Patients transferred from other institutions.
- Patients with demyelinating diseases or neuromuscular junction disorders.
- Patients requiring neuromuscular relaxation.
- Patients with a life expectancy of less than 180 days.
- Patients who, according to medical criteria, would not benefit from multimodal treatment.
- Patients who are not being admitted to the ICU for the first time.
- Patients participating in other clinical rehabilitation trials.

Methodology

Resources:

Humans:

Respiratory therapist's

Materials:

-DEVICES AND SUPPLIES FOR THE SUCTION:

INPUT	RECORD INVIMA	BATCH
Pressure gauge suction: Vacutron® Continuous Suction regulator	NO APPLY	NO APPLY
1 Canister of 2000 cc brand Biolife	NO APPLY	NO APPLY

1 Receptal with 2000 cc valvebrand Biolife	2017DM-0017348	10100923
2 suction tubes silicone	2021DM-0008052 R1	1044923
Suction probes12 French		
Suction cannula disposable Yankauer	2019DMM0005249R1	2208011896
Suction probe closed Tube endotracheal 14 FR.	2018DM-0018691	20027825

-DEVICES AND SUPPLIES FOR THE FAN MECHANIC

INPUT	RECORD INVIMA	BATCH
Disposable circuit adult for him fan mechanic	2014DM-0012091	2022060001
exchanger filter heat and humidity HMEF (Humidificationpassive)	2018DM-0018409	32222070
Anchor tube brafast	2021DM 0023253	2H252
AMBU PVC adult	2018DM-0018376	2104282A / 2012031A
Ventilation mask mechanics No invasive size M	20100DM-0007932	1450304
Ventilation mask non-invasive mechanics size L	20100DM-0007932	1450304

-DEVICES AND SUPPLIES FOR THERAPY RESPIRATORY

INPUT	RECORD INVIMA	BATCH
Incentive Respiratory	2020DM-0021287	10830821
Cough Assist Philips Respironics E70	NO APPLY	NO APPLY
Circuit assistant of cough	2018DM00182293	32302952
Mask facial size 5	2016DM-0014955	2023020001
Micronebulization Kit adult	2018DM-0018538	32305306

-DEVICES OF OXYGEN THERAPY

INPUT	RECORD INVIMA	BATCH
Cannula nasal adult	2022DM-0009342-R1	23S3100

Humidifier	2017DM-0000413-R1	10160523
Mask Venturi	2017DM-000410R1	10160222
Mask of no Rebreathing	2016DM-0015089	21210618050
Mask Simple	2017DM0000410R1	10220823
Circuit AIRVO 2	2014DM-0011418	2102557742
Water sterile of 3000 ml	2020DM-011991-R3	SB23IB6
Connector tube Oxygen	2016DM-0015089	20230505077
High flow cannula interface SIZE M	2014DM-0011418	2101649851
High flow cannula interface SIZE L	2014DM-0011418	2101451544

**Do not forget that depending on the fan you use, the correctcalibration of the equipment, previous to the establishment in patient.

NAME FAN	BRAND
P.B. 980	Medtronic
PB840	Medtronic
SV800/ SV600	Mindray
AVEA	Vyaire care Fusion
CARESCAPE R860	General Electric
Engstrom	General Electric
Hamilton S1	EMCO
Evita V500	Drager
Servo U	Maquet

** Please note that the Engström and Carescape R860 ventilators must be calibrated with all the supplies that will be used with the patient: expiratory bacterial/viral filter, disposable circuit, CO2 measurement chamber, analysis and sampling lines, HMEF filter, closed suction catheter. The other ventilators are calibrated only with the disposable mechanical ventilation circuit.

Intervention

Techniques of permeabilization of the via aerial:

1. **Driving of secretions in ventilation mechanics**

1.1. Open and Closed Suctioning:

There are two methods for performing secretion suctioning:

(a) Closed Suction System: This method uses a closed circuit that allows suctioning the patient without disconnecting mechanical ventilation. The suction catheter is protected by a plastic sleeve. The process involves inserting the catheter into the endotracheal tube and pushing the catheter until the plastic sleeve slides over the catheter. Suction is applied using the thumb and index finger while withdrawing the catheter.

(b) Open Suction System: In this method, the suction catheter is introduced when mechanical ventilation is temporarily disconnected. (8)

1.2. Cough Assistance to Reduce Postoperative Pain

This intervention focuses on managing cough and aims to reduce pain associated with coughing exercises. The fixation of the surgical incision plays a crucial role in this process. Fixation is achieved using the patient's hands or strategically placed pillows. This practice aims to decrease intrathoracic or intra-abdominal pressure in the context of surgical procedures. By reducing pressure, pain, and discomfort are mitigated during exercises that promote coughing.

1.3. Forced Expiration Technique or Expiratory Flow Acceleration

This technique is employed as a way to rapidly and forcefully modulate expiratory flow. Its mechanism lies in the drainage of secretions located in the middle and central airways. Dynamic compression is achieved through forced expiration, which shifts the equal pressure point towards proximal regions. This change increases expiratory flow, thereby facilitating the movement of bronchial secretions and their eventual elimination. The objective of this technique is to promote airway clearance, ensuring that secretions do not remain stagnant in the central and middle areas of the bronchial tree. (9)

1.4. Bronchial Cleaning Techniques Using Expiratory Flows

1.4.1. Slow Expiratory Techniques in Non-Mechanically Ventilated Patients (ELPr)

This technique is employed when the patient has secretions in the mid-airways. It involves performing slow and controlled expirations with an open glottis, similar to the action of fogging up a mirror. The objective is to move the secretions from the mid-airways to more proximal bronchi and the trachea. By maintaining a slow expiration without structural obstructions in the airway exit, the secretions are more easily carried out, contributing to the cleaning of the respiratory airways.

1.4.2. Expiratory Techniques in Mechanically Ventilated Patients

In mechanically ventilated patients, the concept of "bias flow" becomes relevant. The difference between the peak inspiratory flow (PIF) and the peak expiratory flow (PEF) determines the direction of secretion movement. An increase in inspiratory bias flow mobilizes secretions towards the lungs, while an expiratory bias flow directs them towards the trachea. To achieve a PEF - PIF difference > 33 l/min, the following steps should be carried out transiently (for up to a maximum of 30 minutes): (a) Increase Tidal Volume: Increase the tidal volume by 50-150% of the initial value. It is important to ensure that the peak pressure does not exceed 40 cm H2O and the plateau pressure does not exceed 30 cm H2O. (b) Reduce Respiratory Rate: Reduce the respiratory rate to maintain the baseline minute volume. (c) Decrease Inspiratory Flow: Decrease the inspiratory flow to 20 liters/minute. If the patient is in pressure control mode, increase the inspiratory time; if in pressure support mode, reduce the cycling percentage. (d) Transient Adjustments: These adjustments are maintained transiently for a maximum of 30 minutes, after which the parameters are returned to their previous values. (10)

1.5. Directed Cough

Directed cough involves a voluntary coughing effort aimed at increasing the airflow velocity during expulsion. An effective cough should generate a peak expiratory flow (PEF) of more than 160 l/min. This technique aims to mimic the effectiveness of a spontaneous cough, promoting more efficient clearance of bronchial secretions.

1.6. Prolonged Slow Expiration (PSE)

Prolonged Slow Expiration (PSE) is a passive and manual technique that modulates the expiratory flow slowly. Its primary objective is to achieve a greater expiratory volume than that obtained during a normal exhalation. The maneuver involves prolonging and completing the normal expiratory phase, thereby avoiding bronchial collapse. By extending this phase, effective clearance of the peripheral bronchial tree is facilitated, contributing to the elimination of secretions.

1.7. Cough Assist Device

The cough assist device, also known as Cough Assist, is designed to simulate a normal cough and assist in the process. This device replaces or aids in two of the three phases of coughing. During the inspiratory phase, it generates positive pressure to allow lung inflation. Subsequently, a rapid switch to negative pressure occurs during the expiratory phase to expel the insufflated air, facilitating the mobilization and elimination of secretions. The Cough Assist measures the peak cough flow (PCF). It has been observed that the minimum value necessary to achieve optimal secretion hygiene is 160 L/min. A patient with a peak cough flow of less than 270 L/min requires mechanical cough assistance. This support provides additional aid to the muscle groups involved in the coughing process. (11)

2. Lung Re-expansion Techniques

2.1. Diaphragmatic Exercises in Patients without Mechanical Ventilation: diaphragmatic exercises focus on improving the function of the diaphragm during the respiratory process. In normal breathing, during inspiration, the upper part of the abdominal wall projects forward due to the descent of the

diaphragm, while during expiration, the abdomen moves backward. The technique consists of the following steps: (a) Deep inhalation through the nose, directing the air towards the abdomen. (b) Hold your breath for approximately 3 seconds. (c) Perform a slow exhalation through pursed lips, retracting the abdomen.

During execution, the respiratory therapist's hand guides the movement of air. The position of the patient influences the specific mobilization of the diaphragm: (a) Dorsal recumbency: Focuses on the posterior portion of the diaphragm. (b) Right lateral decubitus: It goes to the right hemidiaphragm. (c) Left lateral decubitus: It goes to the left hemidiaphragm.

2.2. Abdomino-Diaphragmatic Directed Ventilation Exercises: this technique aims to improve ventilation at the lung bases. The respiratory therapist stands next to the patient and guides the exercise as follows: (a) Slow inhalation through the nose. (b) Simultaneous elevation of the abdomen during inspiration. (c) With expiration, the abdomen contracts inward.

The therapist provides constant support and guidance throughout the exercise.

23. Respiratory Incentive: The respiratory incentive is a training device that uses visual feedback to promote sustained maximal inspiration. Its objective is to improve respiratory function, increasing negative intrathoracic pressure and expanding the alveoli when inhaling deeply. This device contributes to improving lung ventilation, increasing chest wall expansion, and reducing the loss of lung function and its associated complications.

The incentive device consists of a mouthpiece connected to an inspiratory flow system with three flow levels: 600 ml/s, 900 ml/s, and 1200 ml/s. The patient inhales through the mouthpiece, the air flows through the device, and moves the spheres according to the inspiratory flow. A flow rate of 1200 ml/s indicates a maximum flow achieved. (12)

3. Techniques of strengthening muscular respiratory

3.1. Diaphragmatic Muscle Training in Ventilated Patients Using Pressure Trigger

The trigger mechanism of a mechanical ventilator is designed to detect the patient's respiratory effort. Sensitivity can be set by pressure or flow. In pressure sensitivity, the patient's inspiratory effort causes a programmed drop. The pressure sensitivity is adjusted to 20% of the first Negative Inspiratory Force (NIF) recorded in the patient, ensuring a respiratory rate between 20 and 30 breaths per minute and a tidal volume of 4 to 6 ml/kg. The training involves performing 2 sessions per day, starting with 5 minutes and increasing by 5 minutes per session until reaching 30 minutes.

If a patient tolerates 30 minutes of inspiratory muscle training, the next session is conducted with an increase in trigger sensitivity by 10% of the initial NIF for 5 minutes.

In-Depth Section

NIMV Post-Extubation

Non-invasive mechanical ventilation (NIMV) is a simple and feasible resource in this context, as it has been shown to prevent reintubation in the following scenarios:

As a Prophylactic Measure: In patients who have experienced prolonged or difficult weaning from mechanical ventilation and are at high risk of reintubation. Examples include patients with prolonged mechanical ventilation, ARDS, COPD, and heart failure.

Treatment and Maintenance: In patients who were extubated and developed acute respiratory failure within the first 48 hours post-extubation.

- **Respiratory Incentive (RI)**

The respiratory incentive is a device that promotes slow and deep breaths. It is simple and easy to handle, with the primary goal of preventing and treating atelectasis in patients predisposed to shallow breathing, which can cause small areas of collapse or passive atelectasis, whether due to rest or post-surgical conditions. It is particularly useful in patients with neuromuscular disease.

The respiratory incentives available on the market are classified into two types: volume displacement incentives and flow-dependent incentives. The former measures the amount of air volume displaced during an inspiratory effort, while the latter qualitatively measures the inspiratory flow achieved by the patient during a deep inspiration. The effectiveness of the respiratory incentive depends on two conditions:

1. Proper Education and Supervision: Ensuring the correct performance and execution of the technique.
2. Complementing the Respiratory Incentive with Breathing Exercises: Clinical evidence has demonstrated the efficacy of combining respiratory incentives with targeted ventilation exercises and diaphragmatic strengthening.

In this study, we will use the flow-dependent respiratory incentive, which consists of a mouthpiece and a corrugated tube connected to a collector formed by three tubes containing three lightweight plastic spheres. As the patient performs the inspiratory effort through the mouth, a negative pressure is created within the tubes, allowing the spheres to rise. This device is designed so that the number of spheres and the level of elevation DEPEND ON THE MAGNITUDE OF THE INSPIRATORY FLOW. The flow target to be achieved by elevating the three spheres is 1200 ml/s, with the first sphere representing 600 ml/s, the second sphere representing 900 ml/s, and the third sphere, as mentioned above, representing 1200 ml/s.

- **Cough Assist Post-Extubation**

The guideline for initiating the cough assist device will be directed according to the peak cough flow measured before extubation.

The Cough-Assist is a mechanical assistance device for coughing. It belongs to the category of

mechanical insufflation/insufflation (MI-E) devices. It is a portable electro-mechanical device that facilitates and reproduces the mechanism of physiological coughing. It intervenes from the beginning to the end of the cough cycle. Specifically, it assists with inspiration with positive pressure (hyperinflation), immediately followed by forced expiration with negative pressure (hyperexsufflation). Therefore, this device simulates physiological coughing and provides respiratory cycles that alternate between positive pressures, inspiratory pauses, and abrupt changes to negative pressures. The goal is to improve cough efficacy, thereby facilitating the mobilization of secretions in the bronchial tree and their subsequent expectoration.

Indications

-Cough-Assist is indicated for patients who are unable to cough or effectively clear secretions due to a reduction in peak expiratory flow, or when the peak cough flow in an unstable situation is less than 270 l/min.

-Conditions such as cystic fibrosis, bronchiectasis, and atelectasis due to secretion obstruction.

-Although the literature frequently describes its use in neuromuscular diseases, it is also beneficial for any type of patient with general weakness, such as those in intensive care or post-operative settings.

-Initially developed to assist patients with impaired physiological coughing, it is now considered one of the most effective techniques for clearing respiratory secretions in patients with neuromuscular diseases.

Contraindications

Cough-Assist is contraindicated in the following cases:

Untreated pneumothorax or susceptibility to pneumothorax

Hypotension and unstable hemodynamic criteria

Pneumomediastinum

Significant hemoptysis

Emphysematous bullae

Airway instability

Bronchospasm crisis

Severe laryngomalacia

Execution

The Philips Cough-Assist E70 mechanical insufflator-exsufflator device will be used.

-Objective: Perform a series of 4 to 6 cycles followed by a rest period, depending on the patient's condition.

-Initial Cycles: The first 2-3 cycles are hyperinflation and exhalation cycles during which the patient should achieve an increase in expiratory flow.

-Subsequent Cycles: The last 2-3 cycles are hyperinflation cycles followed by an active coughing effort by the patient during insufflation.

-Repetition: The series can be repeated 4 to 6 times or alternated with a peripheral airway clearance technique.

-Session Duration: The duration of the session depends on the patient's level of obstruction and their state of fatigue.

Cough Assist Programming

Although the parameter values for the cough assist depend entirely on the patients, the adjustable parameters are the pressure levels and application times.

In the latest version of the Cough-Assist E70, the positive and negative pressures can be adjusted between +70 and -70 cm H2O.

It is preferable to start the session with low pressures (between +15 and +20 cm H2O, and -20 to -25 cm H2O) to acclimate the patient.

Higher pressures result in greater air movement.

The literature reports good results with low pressures (+30 to -30 cm H2O).

Generally, +40/-40 cm H2O is sufficient to produce well-tolerated assisted coughing in most restrictive patients. Additionally, the inspiration, expiration, and pause times can be adjusted (0 to 5 seconds). The inspiratory time typically varies between 2 and 3 seconds, while the expiratory time ranges from 3 to 4 seconds. (32)

Conventional Therapy

Conventional therapy refers to the comprehensive approach of respiratory therapy, carrying out the following interventions:

-Respiratory Incentive: Using devices to promote slow and deep breaths.

-Breathing Exercises with Directed Ventilation: Deep breaths redirected to different pulmonary segments at the apical, mid-lateral, and basal levels.

-Diaphragmatic Reconditioning: Strengthening exercises for the main muscle of respiration.

-Cough Exercises: Teach the patient to cough effectively following directed ventilation exercises, to clear the bronchial tree.

Oxygen therapy systems are selected based on the clinical needs and circumstances of the patient. (29) :

DEVICE	PROS	CONS
**Nasal Cannula (FIO2 23-36%)**	Comfortable<br>Low cost<br>The patient can speak and eat with tranquillity	FIO2 is limited and unrealistic by being the device of low-flow<br>Depending on the ventilationminute/ opening oral

**Mask facial simple(FIO2 35-60%)**	Low cost<br>FIO2 independent of the opening oral	No is comfortable<br>Risk of hypercapnia with flows < 5 liters/minute<br>the patient No can eat or talk with tranquillity

**Venturi mask (FIO2 24-fifty%)**	Reduces the risk of hyperoxia and hypercapniaLittle training in aerosol sprays	Professional experience necessary<br>The colors of the Venturiare adjusted according to the maker<br>The mask prevents talking and eat with tranquillity
**Mask with reservoir(FIO2 60-90%)**	High oxygen flows are very useful in situations of emergency	Risk of hypercapnia with flows < 5 liters/min<br>Bit comfortable
**Cannula of high flow (FIO2 21-100%)**	FIO2 stable Very comfortable<br>CO2 washing in the space dead anatomical	Strict monitoring<br>High cost

Respiratory Therapy Intervention

According to the Respiratory Therapy care model in the ICU of the Hospital Universitario de la Fundación Santafé de Bogotá (FSFB), a patient begins intervention when the attending physician requests a consultation with Respiratory Therapy. Respiratory therapists conduct the evaluation and initiate the treatment plan.

As part of the SECEC-2023-129 project, there will be two intervention groups: early multimodal therapy and late multimodal therapy. Early Multimodal Therapy: This will be initiated within the first 24 hours following the intubation of a patient in the ICU. Late Multimodal Therapy: This will be initiated according to the Respiratory Therapy care model for critically ill patients at the Fundación Santa Fe de Bogotá, i.e., when the attending physician requests a consultation with Respiratory Therapy.

Regardless of the group to which the patient is assigned, 3 sessions will be conducted daily (morning, afternoon, and night) seven days a week, with each session lasting:

Dividing into 3 big categories:

- Techniques of permeabilization of the airway
- Techniques of re-expansion pulmonary
- Techniques of strengthening muscular respiratory

INTERVENTION	DURATION OF THE SESSION

PATIENT LOW VENTILATION MECHANICS INVASIVE	
Suction of secretions by tube endotracheal	3 minutes
Suction of secretions oropharyngeal + Hygiene of cavity oral	5 minutes
Bronchial hygiene technique using flows expiratory in ventilation mechanics	15 minutes
muscle training diaphragmatic in a patient ventilatedby half of the trigger by pressure	5 minutes
Time total employee: ***28 minutes***	
**PATIENT ALREADY WITHOUT VENTILATION MECHANICS INVASIVE**	
Exercises diaphragmatic in patients without ventilation mechanics	5 minutes
Exercises of ventilation-directed	5 minutes
Incentive respiratory	3 minutes
Slow expiration techniques prolonged	3 minutes
Techniques of directed cough	3 minutes

Techniques of expiration forced	3 minutes
Utilization of assistance of cough either *COUGH ASSIST*	5 minutes
TIME TOTAL EMPLOYEE: ***27 minutes***	

Responsible Party, Frequency, and Location

Responsible Party: Respiratory Therapist
Frequency: According to medical orders and/or the discretion of the Respiratory Therapy professional
Location: Adult Intensive Care Unit (Third and fourth floors)

References

1. Abplanalp LA, Ionescu F, Calvo-Ayala E, Yu L, Girish ·, Nair B. Static Respiratory System Compliance as a Predictor of Extubation Failure in Patients with Acute Respiratory Failure. Lung [Internet]. 2023 [cited 2023 Jun 26];201:309–14. Available from: https://doi.org/10.1007/s00408-023-00625-7
2. Smina M, Salam A, Khamiees M, Gada P, Amoateng-Adjepong Y, Manthous CA. Cough peak flows and extubation outcomes. Chest [Internet]. 2003 Jul 1 [cited 2023 Jun 20];124(1):262–8. Available from: http://journal.chestnet.org/article/S0012369215360190/fulltext
3. Dres M, Dubé BP, Dubé D, Mayaux J, Delemazure J, Reuter D, et al. Coexistence and Impact of Limb Muscle and Diaphragm Weakness at Time of Liberation from Mechanical Ventilation in Medical Intensive Care Unit Patients. 2017 [cited 2023 Jun 26]; Available from: www.atsjournals.org
4. Zein H, Baratloo A, Negida A, Safari S. Emergency 2016; 4 (2): 65-71 Ventilator Weaning and Spontaneous Breathing Trials; an Educational Review. [cited 2023 Jul 25]; Available from: www.jemerg.com
5. Subirà C, Hernández G, Vázquez A, Rodríguez-Garciá R, González-Castro A, Garciá C, et al. Effect of Pressure Support vs T-Piece Ventilation Strategies During Spontaneous Breathing Trials on Successful Extubation Among Patients Receiving Mechanical Ventilation: A Randomized Clinical Trial. JAMA [Internet]. 2019 Jun 11 [cited 2023 Jul 25];321(22):2175–82. Available from: https://pubmed.ncbi.nlm.nih.gov/31184740/
6. Zarrabian B, Wunsch H, Stelfox HT, Iwashyna TJ, Gershengorn HB. Liberation from Invasive Mechanical Ventilation with Continued Receipt of Vasopressor Infusions. Am J Respir Crit Care Med [Internet]. 2022 May 1 [cited 2023 Jul 25];205(9):1053–63. Available from: https://pubmed.ncbi.nlm.nih.gov/35107416/
7. Jaber S, Jung B, Chanques G, Bonnet F, Marret E. Effects of steroids on reintubation and post-extubation stridor in adults: meta-analysis of randomised controlled trials. Crit Care [Internet]. 2009 Apr 3 [cited 2023 Jul 25];13(2):R49. Available from:

/pmc/articles/PMC2689493/
8. SISTEMAS DE ASPIRACIÓN DE SECRECIONES CERRADOS: INDICACIONES Y CUIDADOS. CLOSED

SECRETION SUCTIONING SYSTEM: INDICATIONS AND CARE.
9. Roldan Lysbeth, Sarmiento Piedad. Terapia Respiratoria para profesionales todo lo que debes saber. Vol. 1. Bogotá: Distribuna Editorial Medica; 2020. 185–187 p.
10. Volpe MS, Guimarães FS, Morais CC. Airway Clearance Techniques for Mechanically Ventilated Patients: Insights for Optimization. 2020;
11. Herrero Maria Victoria. Pressures used and peak cough flow during mechanical cough assistance. Revista Cubana de Medicina Militar. 2020 Mar;
12. Zeng P, Lin Y, Chen Y, Tan G. Effects of incentive spirometry respiratory trainer device on lung recruitment in non-intubated mechanical ventilation moderate ARDS patients: A retrospective study. Heliyon. 2023 May;9(5):e16073.
13. Acosta Ortega Adriana. PROPUESTA DE UN PROTOCOLO PARA EL ENTRENAMIENTO MUSCULAR RESPIRATORIO AL INTERIOR DE LAS UNIDADES DE CUIDADO INTENSIVOS. FASE

II. Universidad Iberoamericana; 2018.
14. Poor H. Respiratory Mechanics. In: Basics of Mechanical Ventilation. Cham: Springer International Publishing; 2018. p. 1–10.
15. Amato MBP, Meade MO, Slutsky AS, Brochard L, Costa ELV, Schoenfeld DA, et al. Driving Pressure and Survival in the Acute Respiratory Distress Syndrome. New England Journal of Medicine [Internet]. 2015 Feb 19 [cited 2023 Jul 31];372(8):747–55. Available from: https://www.nejm.org/doi/10.1056/NEJMsa1410639
16. Mora Carpio AL, Mora JI. Ventilator Management. 2023.
17. Cardozo SL, Sanabria O. Índices de oxigenación: más allá de la PaO2/FiO2 como herramienta ideal. Acta Colombiana de Cuidado Intensivo. 2022 Jul 1;22(3):227–36.
18. Jiménez Duran DP, Cruz Mosquera FE, Arango Arango AC, Ávila Ovalle IJ. Medición del neumotaponador y su influencia en la prevención de complicaciones laringotraqueales: a propósito de un caso de intubación prolongada (56 días). Acta Colombiana de Cuidado Intensivo. 2018 Jan 1;18(1):66–9.

19. Haudebourg AF, Moncomble E, Lesimple A, Delamaire F, Louis B, Mekontso Dessap A, et al. A novel method for assessment of airway opening pressure without the need for low-flow insufflation. Crit Care. 2023 Jul 7;27(1):273.
20. Bertoni M, Telias I, Urner M, Long M, Del Sorbo L, Fan E, et al. A novel non-invasive method to detect excessively high respiratory effort and dynamic transpulmonary driving pressure during mechanical ventilation. Crit Care. 2019 Dec 6;23(1):346.
21. Grassi A, Telias I, Bellani G. Monitoring the Patient During Assisted Ventilation. In: Mechanical Ventilation from Pathophysiology to Clinical Evidence. Cham: Springer International Publishing; 2022. p. 61–73.
22. Domínguez Cherit G, Rivero Sigarroa E, Vidal Mayo J de J, Mercado Velázquez P, Nicolás Martínez EL. Impacto de las asincronías en el pronóstico del paciente bajo ventilación mecánica invasiva. Medicina Crítica. 2020;34(5):273–8.
23. Jubran A. Advances in Respiratory Monitoring During Mechanical Ventilation. Chest. 1999 Nov;116(5):1416–25.
24. Garzon Posada Natalia. Desenlaces de la escala Omaha+ en los pacientes en cuidado intensivo en la fundación santa fe de Bogotá. Repositorio Universidad del Rosario. 2016;
25. Salam A, Tilluckdharry L, Amoateng-Adjepong Y, Manthous CA. Neurologic status, cough, secretions and extubation outcomes. Intensive Care Med. 2004 Jul 4;30(7):1334–9.
26. Matamis D, Soilemezi E, Tsagourias M, Akoumianaki E, Dimassi S, Boroli F, et al. Sonographic evaluation of the diaphragm in critically ill patients. Technique and clinical applications. Intensive Care Med. 2013 May 24;39(5):801–10.
27. Gómez Grande ML, González Bellido V, Olguin G, Rodríguez H. Manejo de las secreciones pulmonares en el paciente crítico. Enferm Intensiva. 2010 Apr;21(2):74–82.

28. Smailes ST, McVicar AJ, Martin R. Cough strength, secretions and extubation outcome in burn patients who have passed a spontaneous breathing trial. Burns. 2013 Mar;39(2):236– 42.
29. Gottlieb J, Capetian P, Hamsen U, Janssens U, Karagiannidis C, Kluge S, et al. German S3 Guideline: Oxygen Therapy in the Acute Care of Adult Patients. Respiration. 2022;101(2):214–52.
30. Fajardo-Campoverdi A, González-Castro A, Adasme-Jeria R, Roncalli-Rocha A, Ibarra M, Chica-Meza C, et al. Mechanical ventilator liberation protocol. Recommendation based on review of the evidence. Journal of Mechanical Ventilation. 2023 Mar 15;4(1):31–41.
31. Cairo JM. Mosby's respiratory care equipment. 11th ed. San Luis; 2021.
32. Chatwin M, Simonds AK. Long-Term Mechanical Insufflation-Exsufflation Cough Assistance in Neuromuscular Disease: Patterns of Use and Lessons for Application. Respir Care. 2020 Feb;65(2):135–43.

Occupational Therapy Component

Coordinators

- Marisol Murillo; email: marisol.murillo@fsfb.org.co
- Sara Stefania Martínez; email: sahara.martinez@fsfb.org.co

Justification

The intervention processes of Occupational Therapy are directed towards preventing and mitigating the impact of acute pathological processes on the patient's autonomy and independence in their significant daily activities according to their history and occupational role, as well as preventing future deterioration in neurocognitive, sensorimotor, and psychosocial components (Ares Senra et al., 2014). In intensive care units, the focus will be on providing multisensory stimulation (emphasizing tactile, proprioceptive, visual, olfactory, and auditory inputs), cognitive stimulation, and promoting participation in meaningful daily life activities based on information received from the family, patient, and/or caregiver and reported in the clinical history, mediated by meaningful activities (Celis et al., 2014).

For patients with a RASS (Richmond Agitation-Sedation Scale) score of -3 or lower, multisensory stimulation activities of tactile, proprioceptive, vestibular, gustatory, auditory, and olfactory types will be performed, fully assisted by the therapist. These activities will correlate with simulated daily life tasks incorporating upper limb motor patterns (emphasizing primary patterns such as hand-head, hand-mouth, hand-homolateral, hand-contralateral, and hand-perineal) used in these tasks, as well as joint and skin care and protection. The intervention will be adjusted in an unimodal manner and subsequently multimodal according to the patient's tolerance in bed. Additionally, at the end of each session, the positioning of the appendicular segments (upper and lower limbs) will be ensured in functional resting angles to provide comfort and prevent contractures or edema. If a family member is present, they will be educated and given feedback on the therapeutic action performed and its objectives. Recommendations will also be provided to approach the patient and minimally participate in co-care, emphasizing tactile (skincare) and auditory (voice, audio clips of a maximum of 2 minutes continuous duration) stimuli.

For patients with RASS scores between -2 and -1, intervention processes will involve passive-assisted activities. In addition to sensory stimulation, emphasis will be placed on cognitive feedback processes for recognizing elements and generating daily tasks with instructional follow-up of a maximum of two commands, using integral movement patterns of reaching, grasping, voluntary or rudimentary releasing, and throwing with or without propulsion, emphasizing mid-frontal and lateral planes in bed.

When the patient has a RASS score of 0 to +1, the intervention will focus on the patient's participation in activities actively or autonomously with minimal support from the therapist according to the patient's tolerance. Greater emphasis will be placed on autonomy in daily tasks and the integration of sensory and cognitive commands of low/medium/high complexity according to the patient's evolution and responses. This includes the execution of upper limb patterns (primary,

secondary, and integral) previously worked on in all anatomical planes, both in bed/at the bedside and in a chair, according to the rehabilitation team's progress. The patient and family will be educated on the importance of promoting autonomy in self-care activities and seeking communication tools using pictograms or graphomotor acts with a board, providing support according to the patient's evolution and therapeutic process progress.

For patients with a RASS score of +2 or higher, therapeutic sessions will focus on behavioral and environmental (physical and family) modulation and fostering a connection with the environment through inhibitory multisensory stimulation activities combined with cognitive stimulation directed towards orientation and participation in the patient's significant tasks in an active-assisted manner in bed. Family members will be educated on continuous orientation processes with enriched sensory stimuli converging on a single task of visual, auditory, tactile, and proprioceptive types. All educational processes and recommendations will be provided in a theoretical-practical manner parallel to the intervention process.

Scope

Patients selected for the research project: "Association between early multimodal therapy and days of mechanical ventilation in the intensive care unit of the Fundación Santa Fe de Bogotá: a randomized controlled clinical trial (SECEC-2023-129)."

Target Population

Inclusion Criteria:

- Patients hospitalized in the adult intensive care unit of the Fundación Santa Fe de Bogotá require invasive mechanical ventilation through an endotracheal tube for more than 72 hours and are expected to continue for at least 24 hours.
- Barthel Index score of 70 or higher.

Exclusion Criteria:

- Patients requiring invasive mechanical ventilation through a tracheostomy or nasotracheal tube.
- Patients who have undergone any type of head and neck surgery.
- Cardiac arrest.
- Airway burn.
- Burns cover more than 50% of the body's surface area.
- Patients with chronic obstructive pulmonary disease (COPD).
- Patients referred from another institution.

- Patients with demyelinating diseases or neuromuscular junction disorders.
- Patients requiring neuromuscular relaxation.
- Patients with a life expectancy of less than 180 days.
- Patients who, according to medical criteria, would not benefit from multimodal treatment.
- Patients not admitted to the ICU for the first time.
- Patients participating in other rehabilitation clinical trials.

Methodology

Human Resources:

Occupational Therapists

Materials:

- Computers
- Clinical History
- Assessment: Direct observation and scales
- Multisensory stimulation kit, cognitive and fine motor didactic material, and co-care utensils and elements.

Intervention

Sensory Stimulation: The therapist will present organized stimuli to the patient for minimum periods of 20 to 30 seconds, including tactile (textures, light touch, touch-pressure, and brushing), proprioceptive (traction/compression, vibration, and motor pattern assistance), auditory (music, familiar voice/isolated sounds, and audio clips), vestibular (directed head movements accompanying the localization of other stimuli), olfactory (fragrances or everyday smells), gustatory (correlated with olfactory and visual inputs coordinated with speech therapy), and visual (shapes, colors, everyday images, and photographs). The session will start unimodal or unisensory, and according to the patient's tolerance, the number of stimuli will be increased, oriented or immersed in a daily activity with significant elements for the patient according to their occupational history (Ares Senra et al., 2014; Celis-Rodríguez et al., 2013; Costigan et al., 2019; Hellweg, 2012; Martínez-Leiva et al., 2020; Sanz et al., 2004; Silva Barbosa & Da Silva Reis, 2017; Weinreich et al., 2017).

Comfort Strategies

Positioning the different appendicular body segments at rest with joint supports to avoid osteotendinous overexertion and/or contractures, pain, and edema, with emphasis on the upper and lower limbs at the distal level, maintaining angles that generate comfort or neutral positioning and external support to stabilize joint segments in different positional changes. This promotes the approximation of the upper limbs to the midline and eliminates pressure points, using orthoses or devices if required, fostering body awareness, and making environmental modifications in sensory

terms (Ares Senra et al., 2014; Celis et al., 2014; Celis-Rodríguez et al., 2013; Martínez-Leiva et al., 2020; Moreno-Chaparro et al., 2017; Provancha-Romeo et al., 2019).

Cognitive Stimulation

Semi-structured activities according to the patient's sedation level (Álvarez et al., 2017; Celis-Rodríguez et al., 2013; Costigan et al., 2019; Martínez-Leiva et al., 2020; Sheard et al., 2022; Silva Barbosa & Da Silva Reis, 2017; Weinreich et al., 2017) by complexity levels as follows:

- Low: Recognition of presented elements or searching for them according to a command, describing perceived characteristics, everyday use through dialogues with affirmative or negative responses and selection; recognition of family members and the hospital setting, as well as elements or devices in their room and on their body for survival.
- Medium: Activities that demand basic reasoning, attentional, mnemonic, and visuospatial functions through games or cognitive challenges with 2 or 3 commands and dual tasks, generally involving manipulation of elements according to progress in motor patterns and manipulative skills, with continuous or alternating visual support as needed.
- High: Activities involving abstract reasoning, information analysis, and solving challenges using basic cognitive functions with a higher degree of complexity according to the patient's cognitive reserve, daily activities, and roles performed before the current hospitalization.

Functionalization of Upper Limb Motor Patterns

The therapist incorporates education and training for the recovery or maintenance of functional mobility (AOTA, 2020; Celis et al., 2014; Celis-Rodríguez et al., 2013; Hellweg, 2012; Martínez-Leiva et al., 2020; Rapolthy-Beck et al., 2021; Schweickert et al., 2009; Silva Barbosa & Da Silva Reis, 2017; Weinreich et al., 2017), especially upper limb motor patterns required in daily activities, particularly self-care actions such as feeding, bathing, grooming, and dressing (upper and lower). These patterns include:

Primary Patterns: Hand-mouth, hand-head, hand-homolateral, hand-contralateral, hand-perineal.

Secondary Patterns: Hand-knee, hand-foot, hand-waist, and hand-back.

Integral Patterns: Reaching (sagittal and coronal planes, anterior/posterior middle, upper, and lower), releasing (rudimentary or precise), throwing (with and without propulsion), and grasping (palmar, penta, tri, and bi-digitals).

Additionally, the improvement of manipulative skills involves processes of sensorimotor and cognitive coordination, as well as grip strength modulation, emphasizing the reduction of compensations, planning, organization, and coordination in each motor praxis necessary for activities, increasing complexity by anatomical planes, starting from execution in the middle, lower, and upper planes according to the patient's evolution and tolerance.

Promotion of Autonomy in Daily Activities

The therapist promotes the highest degree of autonomy and independence in the patient's daily activities, both in self-care and instrumental and leisure activities, integrating cognitive, and sensory

demands, and execution of upper limb motor patterns and manipulative skills. Each session is structured in phases and tasks with exemplification and initial motor direction, patient execution, and continuous visual, verbal, and mirror feedback for adjustments and adaptations the patient needs to make in favor or against gravity according to the patient's evolution and tolerance. This includes necessary positional changes for the tasks to be developed during the session (Álvarez et al., 2017; Hellweg, 2012; López, 2010; Martínez-Leiva et al., 2020; Moreno-Chaparro et al., 2017; Rapolthy-Beck et al., 2021; Schweickert et al., 2009; Sheard et al., 2022; Silva Barbosa & Da Silva Reis, 2017; Weinreich et al., 2017).

Patient and Family/Caregiver Education

Continuous practical orientation to ensure active participation, greater adherence of the patient and their family to the therapeutic process, and safe co-care. According to the patient's sedation level, at the beginning and end of each intervention, relevant recommendations are provided for each stage of the functional recovery process in line with the activities performed during the session and according to the patient's functional capacity as they evolve clinically. This aims to reduce over-assistance in daily tasks and promote the patient's occupational participation (Ares Senra et al., 2014; Celis-Rodríguez et al., 2013; López, 2010; Martínez-Leiva et al., 2020; Rapolthy-Beck et al., 2022).

Occupational Therapy Intervention

According to the Occupational Therapy care model in the ICU of the Hospital Universitario de la Fundación Santafé de Bogotá (FSFB), a patient begins intervention when the attending physician requests a consultation with Occupational Therapy. Occupational therapists conduct the evaluation and initiate the treatment plan.

As part of the SECEC-2023-129 project, there will be two intervention groups: early multimodal therapy and late multimodal therapy. Early Multimodal Therapy and Late Multimodal Therapy: This will be initiated within the first 24 hours following the intubation of a patient in the ICU.

This will be initiated according to the standard management by occupational therapy in critical care at the Fundación Santa Fe de Bogotá, i.e., when the attending physician requests a consultation with Occupational Therapy.

Regardless of the group to which the patient is assigned, 1 session per day will be conducted for patients with a RASS score of -3 or lower. When weaning begins (RASS -2 to -1 or higher than +1), 2 sessions per day will be conducted, distributed in the morning and afternoon, seven days a week, with each session lasting 30 minutes.

Please specify the duration of each session and if the duration varies based on patient characteristics.

RESPONSIBLE, FREQUENCY, AND LOCATION

1. Responsible: Occupational Therapist

2. Frequency: According to medical order and/or the Occupational Therapy professional's criteria
3. Location: Adult Intensive Care Unit (Third and fourth floors)

References

Álvarez, E. A., Garrido, M. A., Tobar, E. A., Prieto, S. A., Vergara, S. O., Briceño, C. D., & González, F. J. (2017). Occupational therapy for delirium management in elderly patients without mechanical ventilation in an intensive care unit: A pilot randomized clinical trial. *Journal of Critical Care*, *37*, 85-90. https://doi.org/10.1016/j.jcrc.2016.09.002

AOTA. (2020). *Marco de Trabajo para la Practica de Terapia Ocupacional: Dominio y practica 4 Ed*. AOTA 2020 CUARTA EDICION - Marco de Trabajo para la Practicade Terapia Ocupacional: Dominio y - Studocu. https://www.studocu.com/es/document/universidad-de-burgos/la-terapia- ocupacional-en-los-trastornos-cognitivos/aota-2020-cuarta-edicion/17349125

Ares Senra, L., Diaz-Mor Prieto, C., & Huerta Mareca, R. (2014). Terapia Ocupacional enpacientes ingresados en UCI con daño neurologico y estados de minima conciencia. *TOG. REVISTA TERAPIA OCUPACIONAL GALICIA*, *19*. file:///C:/Users/57310/Downloads/Dialnet-TerapiaOcupacionalEnPacientesIngresadosEnUciConDan-4710536%20(2).pdf

Celis, F., Gálvez, C., Moretti, C., Navarrete, E., Rovengo, M., & Torrent, V. (2014). Terapia ocupacional y paciente crítico. *Revista Chilena de Terapia Ocupacional*, *14*(1), 101. https://doi.org/10.5354/0719-5346.2014.32395

Celis-Rodríguez, E., Birchenall, C., de la Cal, M. Á., Castorena Arellano, G., Hernández, A., Ceraso, D., Díaz Cortés, J. C., Dueñas Castell, C., Jimenez, E. J., Meza, J. C.,Muñoz Martínez, T., Sosa García, J. O., Pacheco Tovar, C., Pálizas, F., Pardo Oviedo, J. M., Pinilla, D.-I., Raffán-

Sanabria, F., Raimondi, N., Righy Shinotsuka, C., … Rubiano, S. (2013). Guía de práctica clínica basada en la evidencia para elmanejo de la sedoanalgesia en el paciente adulto críticamente enfermo. *MedicinaIntensiva*, *37*(8), 519-574. https://doi.org/10.1016/j.medin.2013.04.001

Costigan, F. A., Duffett, M., Harris, J. E., Baptiste, S., & Kho, M. E. (2019). Occupational Therapy in the ICU: A Scoping Review of 221 Documents. *Critical Care Medicine*,*47*(12), e1014-e1021. https://doi.org/10.1097/CCM.0000000000003999

Hellweg, S. (2012). Effectiveness of Physiotherapy and Occupational Therapy after Traumatic Brain Injury in the Intensive Care Unit. *Critical Care Research and Practice*, *2012*, 1-5. https://doi.org/10.1155/2012/768456

López, B. P. (2010). *Terapia Ocupacional aplicada al Daño Cerebral Adquirido*. Ed. Médica Panamericana.

Martínez-Leiva, J., Parra-Montañez, G., & Segura-Esquivel, J. (2020). Acciones y beneficios que proporciona la intervención del Terapeuta Ocupacional en la Unidad de Cuidados Intensivos de adultos. *Revista Terapéutica*, *14*(1), 11-23. https://doi.org/10.33967/rt.v14i1.94

Mendoza, J. E., Apostolos, G. T., Humphreys, J. D., Hanna-Pladdy, B., & O'Bryant, S. E.(2009). Coin Rotation Task (CRT): A New Test of Motor Dexterity. *Archives of Clinical Neuropsychology*, *24*(3), 287-292. https://doi.org/10.1093/arclin/acp030

Moreno-Chaparro, J., Cubillos-Mesa, C., & Duarte-Torres, S. C. (2017). Terapia ocupacional en unidad de cuidados intensivos. *Revista de la Facultad de Medicina*,*65*(2), 291-296. https://doi.org/10.15446/revfacmed.v65n2.59342

Provancha-Romeo, A. F., Hoffman, A. L., Malcolm, M. P., Coatsworth, J. D., Laxton, L. R.,Freeman, K. M., & Schmid, A. A. (2019). Mind-body interventions utilized by an occupational therapist in a medical intensive care unit: An exploratory case study. *Work*, *63*(2), 191-197. https://doi.org/10.3233/WOR-192920

Rapolthy-Beck, A., Fleming, J., & Turpin, M. (2022). Occupational therapy service provision in adult intensive care units in Australia: A survey of workload practices,interventions nd barriers. *Australian Occupational Therapy Journal*, *69*, 316-330.

Rapolthy-Beck, A., Fleming, J., Turpin, M., Sosnowski, K., Dullaway, S., & White, H. (2021). A comparison of standard occupational therapy versus early enhanced occupation-based therapy in a medical/surgical intensive care unit: Study protocol for a single site feasibility trial (EFFORT-ICU). *Pilot and Feasibility Studies*, *7*(1),51. https://doi.org/10.1186/s40814-021-00795-2

Sánchez, D. P., & Mora, L. T. O. (s. f.). *EVALUACIÓN DE LA INTEGRIDAD SENSORIAL*.

Sanz, S., De Pobes, A., Bové, M., Tàsies, S., Andrés, B., Noguera, A., Soriano, M., &Roig, M. (2004). Terapia Ocupacional en el estado vegetativo y de mínima conciencia: Estimulación sensorial. *Mapfre Medicina*, *15*, 112-117.

Schweickert, W. D., Pohlman, M. C., Pohlman, A. S., Nigos, C., Pawlik, A. J., Esbrook, C.L., Spears, L., Miller, M., Franczyk, M., Deprizio, D., Schmidt, G. A., Bowman, A., Barr, R., McCallister, K. E., Hall, J. B., & Kress, J. P. (2009). *Early physical and occupational therapy in mechanically ventilated, critically ill patients: A randomisedcontrolled trial*. *373*.

Sheard, K. L., Lape, J. E., & Weissberg, K. (2022). Occupational Therapy-Led Delirium Management in Long-Term Acute Care: A Pilot. *Physical & Occupational TherapyIn Geriatrics*, *40*(4), 376-391. https://doi.org/10.1080/02703181.2022.2043983

Silva Barbosa, F. D., & Da Silva Reis, M. C. (2017). O papel da Terapia Ocupacional nas unidades de terapia intensiva—Uma revisão da literatura/ The role of occupationaltherapy in intensive care units—A literature review. *Revista Interinstitucional Brasileira de Terapia Ocupacional - REVISBRATO*, *1*(2), 221-239. https://doi.org/10.47222/2526-3544.rbto4753

Weinreich, M., Herman, J., Dickason, S., & Mayo, H. (2017). Occupational Therapy in theIntensive Care Unit: A Systematic Review. *Occupational Therapy In Health Care*, *31*(3), 205-213. https://doi.org/10.1080/07380577.2017.1340690

Physiotherapy Component

Coordinators:

- Catherine Lissell Arévalo Guerrero, email: Catherine.arevalo@fsfb.org.co
- Paula Andrea Barreto Garzón, email: paula.barreto@fsfb.org.co

Justification

In previous decades, less than 50% of patients with acute lung injury requiring orotracheal intubation received a swallowing assessment during their hospitalization (19). However, multidisciplinary rehabilitation groups have increasingly been incorporated (7,20–22). In a study conducted over 5 years in the United States, which included 264,137 patients requiring mechanical ventilation, it was found that patients received physical, occupational, and speech therapy variably. Despite awareness of mobilizing these patients, rehabilitation does not start as early as possible. Only 24% of patients received physical therapy on the same day as mechanical ventilation initiation (23). There is less information available in the literature, but in this study, only 12.2% of patients under mechanical ventilation received occupational therapy, while 33% received speech therapy orders (23).

Rehabilitation therapy varies significantly between geographic regions but generally involves, in addition to the medical team, professionals in physical therapy, respiratory therapy, speech therapy, occupational therapy, and nursing. In countries without direct availability of specialized therapies, either due to training issues or resource availability, the nursing or physical therapy team assumes the roles of occupational, respiratory, and speech therapy (20–24).

Physical rehabilitation is crucial for patients admitted to the intensive care unit as 20-50% of critically ill patients experience some degree of weakness.

Currently, mobilization in intensive care units is accepted as a therapeutic tool with the potential to prevent or attenuate functional deterioration in these patients. However, the ideal timing for initiating this strategy has been widely debated. (3,5)

Early mobilization has been proposed as an attractive policy for this group of patients by some authors. In some studies, it has shown adequate tolerance with less incidence of delirium, more days free of mechanical ventilation, and better functional outcomes at hospital discharge. (2,25–27)

However, its benefit has been widely debated, as some studies have not shown improved outcomes, and some have even reported an increase in adverse events. This has been attributed to multiple irregularities when comparing studies: primarily, there is a lack of standardization of the concept of "early," which in many cases is not even defined. There is also no consensus on the established therapy, the comparator, or "usual therapy," which varies widely depending on institutional policies, inclusion and exclusion criteria, the frequency of interventions, and outcomes (3). Another factor that may contribute to the lack of improvement in outcomes is the heterogeneity of patients, both in their baseline, pathology, and response to proposed interventions in the unit. (3)

In a recent study published in the New England Journal of Medicine (6), which included 750 patients in 49 hospitals across 6 countries, randomized to early mobilization or usual care, there was no difference in 180-day mortality, hospital stay, or secondary outcomes such as days of mechanical

ventilation and days out of the ICU. However, there was an increase in adverse and serious adverse events in the early mobilization group. The authors report limitations such as a higher level of mobilization in the control group compared to previous studies, barriers to mobilization that may have limited the statistical power to detect differences between groups, and surveillance bias in the early mobilization group. (6).

In a meta-analysis and systematic review of the literature published in 2019, which included 23 randomized controlled trials, it was concluded that regardless of the different techniques and periods of mobilization used, early mobilization of critically ill patients increased the number of people able to stand, the number of days free of mechanical ventilation during hospitalization, reduced the incidence of intensive care unit-associated weakness, and increased the walking distance at hospital discharge. There were no significant differences in 28-day mortality or adverse events in this study. (5)

Types of interventions to consider include functional active mobilization, cyclic bed ergometry, electrical muscle stimulation (with or without active or passive exercises), and tilt tables, among others. (4) While passive exercises are not formally part of the rehabilitation process, as they have not been shown to increase muscle strength or endurance (3), they often precede the initiation of active maneuvers and are most commonly used in patients under deep sedation whose clinical condition allows it. (7,24,26,27)

Therapy should be individualized to the patient's condition as much as possible. However, Hickman et al. found only five contraindications for establishing early mobilization, which in their study was defined as initiated within the first 24 hours of ICU stay: acute myocardial infarction, active bleeding, increased intracranial pressure, and unstable pelvic fracture (25). In this group of patients, hemodynamic parameters were hardly affected by therapy, causing its discontinuation in only 0.8% of activities, mainly due to hypotension or arrhythmias (25).

A method to determine the risk/benefit of applying early mobilization to patients in the intensive care unit was developed by a multidisciplinary group and is widely used today. It consists of risk-based categorization, where green is assigned to patients with a low risk of adverse events, meaning the benefit outweighs potential safety consequences. Yellow represents a risk of adverse events, and in this group of patients, precautions and contraindications for early mobilization should be discussed. Red implies a higher risk of adverse events, and in these patients, early mobilization is not performed unless authorized by the responsible medical team. While this category does not constitute a contraindication, it is a warning that in this case, the risks may outweigh the benefits. (20)

Different clinical guidelines support the use of a bundle to reduce or shorten rehabilitation time and the incidence of delirium. The ABCDEF bundle includes the assessment, prevention, and management of pain; awakening and breathing trials, the assessment, prevention, and management of delirium, early mobilization and exercise, and involving and favoring the patient's family environment. (21)

Even in teams that promote early rehabilitation, studies have found multiple barriers to mobilizing patients on mechanical ventilation and maintaining the proposed dose or intervention time. (26)

Barriers to early mobilization include patient-related factors such as signs and symptoms or conditions like hemodynamic or respiratory instability; structural barriers such as human and equipment resources or technical issues; ICU culture-related barriers, including ingrained habits and attitudes in each institution and process-related limitations, lack of coordination, absence of roles and rules that adequately determine and distribute tasks and responsibilities. (27)

One of the most frequent barriers or reasons for not adhering to mobilization protocols is high levels of sedation. Adequate reduction and adjustment of sedation to allow patient participation in exercises is important for favorable outcomes. (27) Other important factors are proper pain management and early recognition and management of delirium. (27)

The recovery goal should be established as early as possible for each patient. With it, strategies should be implemented to improve the implementation of comprehensive rehabilitation, including identifying barriers and facilitators; and creating multidisciplinary teams with leaders to improve communication, education, and training. (3)

Patient safety is a frequently reported barrier. With mobilization, there is an increased risk of removing invasive measures such as tubes, catheters, and equipment, and it is a common belief among doctors and nurses that limits the role of physiotherapy and compromises the implementation of early mobilization protocols. However, this risk is negligible when the program is carried out by trained and qualified personnel. (27)

To improve cultural concerns in the ICU, an international multicenter randomized study implemented the establishment of mobilization goals during the daily medical rounds, achieving them on 89% of the days in the intervention group. This study achieved shorter ICU and hospital stays and improved functional mobility at hospital discharge. (7)

Scope

Patients selected for the research project: "Association between early multimodal therapy and days of mechanical ventilation in the intensive care unit of the Fundación Santa Fe de Bogotá: a randomized controlled clinical trial (SECEC-2023-129)."

Target Population

Inclusion Criteria:

Patients hospitalized in the adult intensive care unit of the Fundación Santa Fe de Bogotá require invasive mechanical ventilation through an endotracheal tube for more than 72 hours and are expected to continue for at least 24 hours.
Barthel Index score of 70 or higher.

Exclusion Criteria:

- Patients requiring invasive mechanical ventilation through a tracheostomy or nasotracheal tube.
- Patients who have undergone any type of head and neck surgery.
- Cardiac arrest.
- Airway burn.
- Burns cover more than 50% of the body's surface area.
- Patients with chronic obstructive pulmonary disease (COPD).
- Patients referred from another institution.
- Patients with demyelinating diseases or neuromuscular junction disorders.
- Patients requiring neuromuscular relaxation.
- Patients with a life expectancy of less than 180 days.
- Patients who, according to medical criteria, would not benefit from multimodal treatment.
- Patients not admitted to the ICU for the first time.

- Patients participating in other rehabilitation clinical trials.

Methodology

Resources:

Materials:

- Gloves – face masks
- Elastic bands (Theraband)
- Adjustable weights
- Walkers
- Neurological chairs
- Oil
- Cold and hot packs
- Electrotherapy and ultrasound equipment
- Therapeutic balls of different weights
- Cycle ergometer
- Cane
- Odometer
- Dynamometer

Intervention

1. Muscle Fiber Recruitment Techniques:

Methods will be used to facilitate neuromuscular activation and recruitment, promote motor planning and control processes, develop coordinative and postural patterns, and maximize functionality.

Proprioceptive Neuromuscular Facilitation (PNF) Techniques with Bilateral Symmetrical and Asymmetrical Patterns for Increasing Strength in All Four Limbs:

a) Rhythmic Initiation: Uses rhythmic movements as an alternative to improve the initiation and speed of movement of a limb or the body. It starts with passive movement through the full range of motion, progressing to active-assisted movement. The therapist then applies resistance to the active movement, ending with a movement performed entirely by the patient. It is used to initiate movement, improve coordination, normalize speed, help the patient relax, and teach movement. Indicated for patients with difficulty initiating movement, slow or fast movements, uncoordinated and arrhythmic movements, to regularize or normalize muscle tone, and to relax muscles.

b) Combination of Isotonics: Stabilizes a body segment through the combination of concentric and eccentric muscle contractions without inhibiting muscle groups. Resistance is applied throughout the patient's range of motion, but at the end of the movement, a contraction is demanded to stabilize the position, ending with an eccentric contraction. The objectives are to activate movement control, and coordination, increase the active range of motion, strengthen, and provide functional training in eccentric movement control.

c) Reversal of Antagonists: Subdivided into:

- Dynamic Reversals: Based on the principle of successive induction, continuous resistance is

applied during movement. As the joint range approaches its end, a sudden change in active movement is demanded, avoiding rest or relaxation time. Objectives include increasing active range of motion, strength, and endurance, developing coordination, and preventing or reducing fatigue and muscle tone. Indicated for patients with decreased active range of motion, weak agonist muscles, difficulty changing movement direction, muscle fatigue, and relaxation of hypertonic muscles.

- Stabilizing Reversals: Uses isotonic muscle contractions against resistance and through traction or approximation to provide joint stability. When the patient responds positively to resistance, the resistance force immediately changes direction. Objectives include increasing stability, balance, muscle strength, and coordination between agonists and antagonists. Indicated for patients with decreased stability, weakness, inability to contract muscles isometrically, and requiring resistance in one direction.

d) Rhythmic Stabilization: Isometric contractions against progressive resistance without the intention of generating movement or position change. Objectives include increasing active and passive range of motion, strength, stability, balance, and reducing pain. Indicated for cases of limited range of motion, pain, joint instability, antagonist muscle weakness, and balance disorders. Contraindicated in patients who do not follow orders, have language comprehension difficulties, or have brain dysfunction. Promotes stabilization of body areas through isometric contractions demanding muscle group synergy, resulting in an excitatory response.

e) Repeated Stretching: Subdivided into:

- Repeated Stretching from the Beginning of the Range: Activates stretch reflexes, with muscles subjected to tension by elongation at the start of the movement pattern.

- Repeated Stretching During the Range: Activates stretch reflexes with muscles subjected to tension due to contraction. Objectives include facilitating movement initiation, increasing active range of motion and strength, preventing or reducing fatigue, and guiding movement in the correct direction.

f) Contract-Relax: Subdivided into:

- Direct Treatment: Movement is guided to the end of the patient's active range of motion, using maximum resisted contraction of antagonist muscles followed by relaxation, increasing range of motion. The objective is to increase the passive range of motion.

- Indirect Treatment: Benefits from contraction of agonist muscles. Indicated when muscle contraction is painful or weak.

g) Hold-Relax: Subdivided into:

- Direct Treatment: Joint is positioned at the end of the range of motion to apply resistance and demand an isometric contraction without movement intention, followed by gradual relaxation. Used to increase the passive range of motion and reduce pain.
- Indirect Treatment: Resistance is applied to muscles distal to the painful body area or segment during the range of motion.

h) Repetition Provides Motor learning sequencing through the reiteration of an activity, executed

synchronously and continuously. Exposes the final movement position and evaluates the patient's ability to maintain a muscle contraction.

2. Proprioceptive Neuromuscular Facilitation with Bilateral Asymmetrical Patterns for Increasing Respiratory Muscle Strength: Both limbs are directed to opposite movement patterns. For example, the right limb moves in flexion-adduction, while the left moves in flexion-adduction. a) Post-Discharge: Perception of increased muscle strength after sustained muscle contraction. b) Temporal Summation: Series of low-intensity stimuli over time generating a higher intensity stimulus and motor excitation response. c) Spatial Summation: Synchronized low-intensity stimuli in various body areas reinforcing each other, generating a response. d) Irradiation: Transmission of a stimulus generated in a specific body segment to another to obtain a contractile response. e) Successive Induction: Benefits from increased excitability of antagonist muscles, facilitating weak agonist muscles. f) Reciprocal Innervation: Achieving inhibitory response of antagonist muscles through agonist muscle contractions against resistance.

Diaphragmatic Muscle Training in Ventilated Patients Using Pressure Trigger with Electro-Stimulation: The ventilator trigger detects the patient's respiratory effort, with sensitivity set by pressure or flow. Pressure sensitivity is adjusted to 20% of the first NIF recorded in the patient, ensuring a respiratory rate of 20-30 breaths/min and tidal volume of 4-6 ml/kg. Two sessions per day, starting with 5 minutes and increasing by 5 minutes per session until reaching 30 minutes. If the patient tolerates 30 minutes of inspiratory muscle training, the next session increases trigger sensitivity by 10% of the initial NIF for 5 minutes.

3. Electro-Stimulation Application: Interferential Therapy (IFT): Low-frequency electrical stimulation for pain relief and muscle stimulation. Electrical Muscle Stimulation (EMS): Generates impulses to stimulate motor neurons and cause muscle contraction. Fixed and mobile current application to the following muscle groups:

   - Inspiration Muscles:
   - Diaphragm (sternal, costal, lumbar)
   - External intercostals (11 pairs)
   - Accessory muscles
   - Innermost intercostals
   - Anterior, middle, and posterior scalenes
   - Expiration Muscles:
   - Internal intercostals (11 pairs)
   - Accessory muscles
   - Rectus abdominis
   - Internal and external obliques
   - Transversus abdominis

Physiotherapeutic Intervention

According to the Physiotherapy care model in the ICU of the Hospital Universitario de la Fundación Santafé de Bogotá (FSFB), a patient begins intervention when the attending physician requests a consultation with Physical Medicine and Rehabilitation (Physiatrist), who provides indications for Physiotherapy. Physiotherapists conduct the evaluation and initiate the treatment plan.

As part of the SECEC-2023-129 project, there will be two intervention groups: early multimodal therapy and late multimodal therapy. Early multimodal therapy will be initiated within the first 24 hours following the intubation of a patient in the ICU. Late multimodal therapy will be initiated

according to the Physiotherapy Intervention protocol for critically ill patients at the Fundación Santa Fe de Bogotá (Code: AMB-REHF-PT-020), i.e., when the attending physician requests a consultation with Physical Medicine and Rehabilitation.

Regardless of the group to which the patient is assigned, two sessions will be conducted daily (morning and afternoon) seven days a week, with each session lasting:

25-minute sessions:

- Patients with clinical conditions requiring ventilatory support, inotropic support, sedo-analgesia, and renal replacement therapy, require continuous supervision and longer recovery periods between exercises.

50-minute sessions:

- Sessions involving ambulation of a patient with invasive or non-invasive mechanical ventilation (mobilization of multiple equipment and coordination of the multidisciplinary team).
- Patients with pulmonary compromise require long rest periods during therapy sessions.
- Patients requiring gait training with or without external assistance.

The physiotherapeutic intervention will be carried out according to the Physiotherapy Intervention protocol for critically ill patients at the Fundación Santa Fe de Bogotá (Code: AMB-REHF-PT-020).

RESPONSIBLE, FREQUENCY, AND LOCATION

Responsible: Physiotherapist
Frequency: According to medical order and/or Physical Therapy professional's criteria
Location: Adult Intensive Care Unit (Third and fourth floors)

Speech Therapy Component

Coordinators:
- Miguel Leonardo Pulido Bobadilla, email: miguel.pulido@fsfb.org.co
- Diana Marcela Melo Rojas, email: dianammelo1992@gmail.com

Justification

The role of speech therapy is increasingly important in intensive care, similar to early mobilization. Speech therapy should be involved in the early assessment of ICU patients, as recognizing laryngeal dysfunction and swallowing disorders can minimize the risk of oropharyngeal dysphagia and aspiration.

Speech therapists can provide support for critically ill patients with acute conditions, whether neurological or of other medical etiologies: trauma, hemorrhages, tumors, spinal injuries, respiratory conditions, as well as patients in complex postoperative states from neurological, cardiac, and general surgery interventions. These conditions can affect motor speech skills, swallowing, coughing, and voice.

Early support from speech therapy helps facilitate successful communication between ICU patients, the medical team, and their families, developing specialized intervention programs for each condition and working with patients from a functional and pathological perspective. This, in turn, improves their psychosocial well-being, engagement in daily care, and informed decision-making. Additionally, communication disorders have been associated with delirium, an emerging problem in intensive care units.

The videofluoroscopic swallowing study (VFS) and the fiberoptic endoscopic evaluation of swallowing (FEES) are necessary for the accurate diagnosis of swallowing disorders, as aspiration is a devastating complication that can occur silently in 30-44% of patients and cannot be diagnosed with bedside evaluations. Factors associated with aspiration processes include pneumonia, feeding tubes, tracheostomy, prolonged ICU stays, and increased hospital mortality.

FEES, unlike VFS, is a portable tool with the additional benefit of providing a direct view of pharyngeal and laryngeal dynamics, as well as the patient's anatomy and secretion management. It can support clinical decisions and often highlights difficulties that can later be resolved. The use of FEES allows for pharmacological recommendations for managing excessive secretions, as well as therapeutic interventions to strengthen the tongue base, pharyngeal constriction, and laryngeal range of motion. Together with VFS, they are considered the gold standard for detecting swallowing disorders.

However, the cost, occasional patient refusal, transportation difficulties, and the requirement for specialized personnel and equipment limit the application of these tools in all patients post-extubation.

The ability to diagnose aspiration easily and accurately could minimize aspiration complications, limiting unnecessary delays in initiating oral intake in ICU patients. Therefore, multiple specialized protocols and tools have been designed to identify patients with established swallowing disorders or at risk of developing them, aiming to adopt a preventive approach to reduce complications and deleterious outcomes.

Among these, a combination of the water swallow test and a bedside swallowing evaluation is the only strategy validated for identifying post-extubation dysphagia in survivors of acute respiratory failure. The Gugging Swallowing Screen (GUSS) is an easy-to-apply tool by different therapists or nurses, allowing a graded evaluation of the patient's swallowing capacity and providing nutritional recommendations.

In a recent study, the GUSS-ICU was compared with FEES, demonstrating 89-92% sensitivity and 67-89% specificity for detecting patients with dysphagia, comparable to results obtained in stroke patients. The inter-observer reliability was good. This tool's advantage is its simplicity and the use of various food consistencies, allowing dietary recommendations for post-extubation patients.

In addition to oropharyngeal muscle strengthening maneuvers with exercises, electrical stimulation at this level has recently been proposed. Some studies have shown that it improves the reorganization of the motor cortex related to swallowing, facilitates the activation of corticobulbar pathways, and increases salivary levels of neurotransmitters associated with swallowing, such as substance P. The PHAST-TRAC study evaluated pharyngeal electrical stimulation in tracheostomized stroke patients with neurogenic dysphagia, finding that this strategy led to a higher number of patients ready for decannulation and a lower prevalence of pneumonia, without an increase in complications.

In the last decade, the value and expertise of therapists supporting the care of patients in intensive care units have been highlighted. Each therapy-related profession offers a wide range of experience, knowledge, skills, and expertise that contribute in different ways to the early, optimal, and comprehensive rehabilitation of ICU patients. Rehabilitation goals should be individualized for each patient, and therapy should be formulated according to these goals. Emphasis should be placed on collaborative interdisciplinary work, and understanding the role and responsibilities of each professional.

Scope

Patients selected for the research project: "Association between early multimodal therapy and days of mechanical ventilation in the intensive care unit of the Fundación Santa Fe de Bogotá: a randomized controlled clinical trial (SECEC-2023-129)."

Target Population

Inclusion Criteria

- Patients hospitalized in the adult intensive care unit of the Fundación Santa Fe de Bogotá require invasive mechanical ventilation through an endotracheal tube for more than 72 hours and are expected to continue for at least 24 hours.
- Barthel Index score of 70 or higher.

Exclusion Criteria:

- Patients requiring invasive mechanical ventilation through a tracheostomy or nasotracheal tube.
- Patients who have undergone any type of head and neck surgery.
- Cardiac arrest.
- Airway burn.

- Burns cover more than 50% of the body's surface area.
- Patients with chronic obstructive pulmonary disease (COPD).
- Patients referred from another institution.
- Patients with demyelinating diseases or neuromuscular junction disorders.
- Patients requiring neuromuscular relaxation.
- Patients with a life expectancy of less than 180 days.
- Patients who, according to medical criteria, would not benefit from multimodal treatment.
- Patients not admitted to the ICU for the first time.
- Patients participating in other rehabilitation clinical trials.

Methodology

Human Resources:

Speech Therapy Professionals

Materials:

- Computers
- Clinical History
- Table
- Defined tests for assessment

Intervention

Passive/Assisted Speech Therapy (Proprioceptive):

Active/Assisted Speech Therapy (Neuromuscular Praxis)

The modified Medical Research Council (MRC) muscle strength scale will be implemented. The assessment of orofacial muscle qualities will begin. Buccinator, masseter, labial, and lingual muscle chains will be evaluated:

1. Cervical strength and mobility assessment.
2. Assessment of lower jaw positioning to verify closing strength and/or oral opening maintenance at rest.
3. Superficial facial sensitivity assessment in the middle, upper, and lower thirds bilaterally.
4. Palpation of orofacial muscle groups to verify muscle tone qualities.
5. Simple praxis movements will be requested from the patient to assess mobility ranges, reach, and strength.
6. Movements will be evaluated against gravity, then with resistance, and finally against resistance.
7. Based on the results, classification within the modified Medical Research Council muscle strength scale will be performed.

Fatigability tendency with repetition will also be considered.

Neck Flexion, Lateral Inclination, and Rotation:

- Superior Cervical Flexion: Works on the longus capitis, rectus capitis anterior, suprahyoid muscles (flexion of the upper cervical spine), and infrahyoid muscles (stabilizes the hyoid).

- Inferior Cervical Flexion: Works on the longus colli, platysma, anterior scalene, sternocleidomastoid.

- Rotation: Contralateral (all scalenes), sternocleidomastoid, Homolateral: longus capitis and colli, rectus capitis anterior.
- Lateral Inclination: Longus colli, all scalenes, and sternocleidomastoid.

Neck Extension, Lateral Inclination, and Rotation:

- Superior Cervical Extension: Iliocostalis and longissimus capitis, obliquus capitis (superior and inferior), rectus capitis posterior (major and minor), semispinalis and spinalis capitis, trapezius.
- Inferior Cervical Extension: Iliocostalis cervicis, longissimus and splenius cervicis, multifidus and rotatores, semispinalis and splenius cervicis, trapezius.
- Rotation: Contralateral: multifidus and rotatores, semispinalis capitis, upper trapezius, Homolateral: inferior obliquus capitis, splenius cervicis and capitis.
- Lateral Inclination: Iliocostalis cervicis, intertransversarii (cervical), longissimus capitis, superior obliquus capitis, splenius cervicis and capitis, trapezius.

Jaw movement is associated with head movement on the neck, mouth opening, and superior cervical flexion mutually reinforce each other, as do mouth closing and superior cervical extension. Irradiation from neck flexion patterns facilitates neck flexion, trunk elongation, and lateral trunk inclination.

The patient should be seated, as it is functional for neck movement and stability. In this position, the flexor muscles must be strong enough to lift the head against gravity:

Physiotherapeutic Intervention

According to the Speech Therapy care model in the ICU of the Hospital Universitario de la Fundación Santafé de Bogotá (FSFB), a patient begins intervention when the attending physician requests a consultation with Speech Therapy. Speech therapists conduct the evaluation and initiate the treatment plan.

As part of the SECEC-2023-129 project, there will be two intervention groups: early multimodal therapy and late multimodal therapy. Early Multimodal Therapy: This will be initiated within the first 24 hours following the intubation of a patient in the ICU.
Late Multimodal Therapy: This will be initiated according to the Speech Therapy care model for critically ill patients at the Fundación Santa Fe de Bogotá, i.e., when the attending physician requests a consultation with Speech Therapy.

Regardless of the group to which the patient is assigned, 2 sessions will be conducted daily (morning and afternoon) seven days a week, with each session lasting 15 to 30 minutes depending on the patient's RASS score.

Responsible, Frequency, and Location:

1. Responsible: Speech Therapist
2. Frequency: According to medical order and/or Speech Therapy professional's criteria.
3. Location: Adult Intensive Care Unit (Third and fourth floors).

Annex 2

Association between therapy multimodal early and days of ventilation mechanicsin the intensive care unit of the Fundación Santa Fe de Bogotá: A rehearsal clinical checked randomized.

**Qualification of the study**	Association between multimodal therapy early and days of ventilation mechanics in the intensive care unit of the Fundación Santa Fe de Bogotá: a rehearsal clinical checked randomized.
**Name of the Main Investigator**	Jorge Ivan Alvarado Sanchez<br>Laura Maria Castle Morales
**Phone of contact with the investigator**	6030303 ext. 5889
**Name of the Institution of Investigation**	Fundación Santa Fe de Bogotá
**Address of the Institution of Investigation**	Street 119A No.7-49, Room floor tower of expansion ICU Adults
**Version and date of the consent**	Version 2.0 of the 05 April 2024
**Number encoded by the competitor**	

The Fundación Santa Fe de Bogotá (FSFB) and the Department of Critical Care Medicine Intensive Care are inviting you to participate/are inviting your family member to participate as a volunteer in a project that compares multimodal therapy (physical, occupational, respiratory, and speech therapy) versus early therapylate multimodality in patients with invasive mechanical ventilation in the Emergency Unit Careful Intensive of the FSFB.

This Informed Consent document will provide you with the information necessary to help you and/or your family member decide whether to participate in the study. Please read the information carefully. If any part of this document is not clear to you or if you have any questions or would like to request additional information, please do not hesitate to ask any of the members of the study team, who are listed at the end of this document, at any time.

1. **NATURE AND PURPOSE OF THE STUDY:** This study aims to evaluate the difference in days of invasive mechanical ventilation between early multimodal therapy and late multimodal therapy (standard management) in the

adult intensive care unit of the Fundación Santa Fe de Bogotá. Mechanical ventilation is a treatment used when a person has difficulty breathing on their own. It is a specialized machine like a "lung, but outside the body" that helps a person breathe by inflating and deflating their lungs in a controlled manner. This process is done through a tube placed in the throat (also known as an endotracheal tube) or through a mask that fits over the mouth and nose.

2. **WHO CAN PARTICIPATE?** To participate you/your family must meet the following criteria:

- People over 18 years of age
- The participant must be hospitalized in the Intensive Care Unit of the Fundación Santa Fe de Bogotá.
- The participant must require invasive mechanical ventilation for more than 24 hours (one day) after admission to the Unit.
- The participant must be a person with high functional capacity, which will be measured using a tool known as the Barthel index (which is a scale used by health personnel to measure how functional a person is in their daily life). and score from 0 to 100). For this research, the index must be greater than 70 points (100 being total independence for daily life tasks and 0 being total dependence of the person on the care of a third party).

3. **ALTERNATIVE TREATMENTS ADVANTAGEOUS FOR THE SUBJECT:** The treating physician will determine if there are other treatments from which the patient can benefit or if the patient does not require all the interventions of multimodal therapy, but rather a subgroup of them. Its participation in this study will contribute to the advancement of medical knowledge in this field.

4. **EXPECTED DURATION OF PARTICIPATION AND NUMBER OF SUBJECTS:** Your participation will last 90 days from the time you require invasive mechanical ventilation. We understand that the duration of mechanical ventilation is probably not 90 days and that you may already be at home with your family, so the follow-up planned for this study will be by telephone so you will not have to travel to the institution again. for it. The total number of participants will be 74.

5. **STUDY PROCEDURES:** There are two groups in this project: an early multidisciplinary therapy group, which is defined as the set of specialized maneuvers carried out by the physiotherapy, speech therapy, respiratory therapy and occupational therapy group starting in the first 24 hours. hours intubation is performed, and mechanical ventilation begins. The second group corresponds to late multidisciplinary therapy, which consists of the same

interventions as the first group, however, the start time will be 72 hours after intubation is performed and mechanical ventilation begins. Continuous monitoring of your or your family member's condition will be carried out as is routinely done in the ICU. The decision to withdraw multidisciplinary therapy will be made by the medical team in charge in the event that these therapies are not in the best interest of you or your family member.

6. **HANDLING OF BIOLOGICAL SAMPLES OBTAINED:** No biological samples will be taken specifically for this project. All biological samples taken will correspond to the usual treatment of Intensive Care Unit patients agreed with the treating medical group.

7. **WHAT IS EXPECTED FROM YOUR PARTICIPATION?** You are expected to participate and collaborate in the development of the study, following the instructions of the medical team.

8. **WHAT WILL HAPPEN AT THE END OF THE STUDY?** At the end of the study, you will be provided with detailed information about the results if you wish. You will also discuss any questions or concerns you may have. In this informed consent report you can check if you want to know this information. If you check "YES" you or your family member will be notified by telephone or email once the study is completed.

9. **POSSIBLE ADVERSE EFFECTS:** There are possible risks associated with participation in the study, including side effects of the different therapies and possible discomfort. Recognized adverse effects are:

- **Altered blood pressure**: Some participants may experience a temporary decrease or increase in blood pressure while taking the therapies, which could cause dizziness or fainting.

- **Cardiac Arrhythmias (changes in the normal heart rhythm):** In rare cases, participants may experience changes in heart rhythm, which could cause palpitations or a feeling of irregular heartbeats.

- **Oxygen desaturation (decrease in the amount of oxygen carried in the body):** In some cases, participants may experience a decrease in the amount of oxygen in their blood, which could result in confusion, dizziness, or a feeling of shortness of breath and fatigue.

- **Pain or agitation (body hyperactivity or a feeling of hopelessness):** some participants may experience pain or agitation due to the intensity or base condition of the participant which may increase when performing the activities corresponding to each therapy.

- **Removal of invasive lines (such as veins or arteries channeled for placement of fluids or medications):** although unlikely, some people may accidentally remove invasive lines (intravenous fluids, parenteral nutrition, infusion pumps). This would imply that these invasive lines would need to be re-established.

- **Tachypnea (increased breathing rate):** Since these therapies involve physical activity, an increased breathing rate is expected. In some cases, participants may experience shortness of breath or fatigue due to increased physical exertion.

- **Neurological impairment:** In rare cases, participants may experience changes in brain function, which may result in confusion, persistent dizziness, or difficulty speaking.

- **Other side effects:** in addition to those mentioned, other unforeseen side effects may occur due to the complexity of medical treatments and the patient's underlying condition. These will be carefully monitored and treated as necessary.

10. **RISKS AND BENEFITS**: Risks include possible side effects of the medical procedures and therapies explained in point 9. Benefits include contributing to the advancement of medical knowledge and possibly improving the treatment of patients with invasive mechanical ventilation in the future.

11. **NEW STUDY INFORMATION**: Your study doctor will inform you promptly of any new information obtained during the study that may affect your willingness to continue participating. When you are told this new information, you will be asked to sign and date a new consent form if you agree to continue in the study.

12**. WHAT ELSE YOU NEED TO KNOW BEFORE DECIDING TO PARTICIPATE**: you will receive a copy of this Informed Consent form, keep it in a safe place, and use it as information and reference throughout the development of the study. This research will be carried out following resolution 8430 of 1993 and 2378 of 2008 of the Colombian Ministry of Health. This document was reviewed and approved by the Corporate Research Ethics Committee and meets all the methodological and ethical requirements to be developed.

Neither you nor the Department of Critical Care Medicine will receive financial compensation for participating in this study.

13. **THERE MAY BE REASONS WHY YOU CANNOT PARTICIPATE**: Your participation in this study is voluntary. You are not required to participate and may withdraw your participation at any time without penalty or loss of benefits to which you are entitled. If you decide to leave the study before the last study visit, inform the study doctor and follow his or her instructions.

14. **COVERAGE OF ADVERSE EVENTS/COMPENSATION**: since the application of multimodal therapy is part of our normal medical practice, complications related to them do not require an additional policy because they are directly associated with the therapy and the underlying medical condition. By signing this consent, you do not waive any of your legal rights that may apply to you if any damage occurs, and it is proven that it has occurred as a direct consequence of the multimodal therapy and your participation in the study. The investigator will make every effort to prevent any harm from occurring.

15. **WHAT WILL HAPPEN IF YOU DECIDE NOT TO PARTICIPATE OR IF YOU CHANGE YOUR MIND**: Participation in this study is completely voluntary, you are not obliged to participate, and you can withdraw at any time without justifying your decision, without suffering any penalty or detriment to care. Part of your doctor or the Institution or you may also be withdrawn by your researcher for some reason that he will explain to you, but in either case tests or procedures will be performed to end your participation in an orderly manner.

16. **CONFIDENTIALITY AND PRIVACY OF DATA**: The researcher will ensure the confidentiality of your clinical history, in which the subject will not be identified, the confidentiality of the information related to your privacy will be maintained, using codes to the extent permitted by laws and regulations and They will not be publicly accessible. The data obtained may be consulted by health authorities, national health authorities, the National Institute of Medicines and Foods - INVIMA - and the Research Ethics Committee.

    The study center will record basic personal information about you, such as your name, contact information, gender, height, weight, and ethnicity, as well as also information about your medical history and clinical data collected about your participation in the study. All personnel with access to your records are obliged to always respect their confidentiality.

    To ensure your privacy, your name or other information that directly identifies you will not be included in the records provided for research purposes. The only people who will be able to link this code to your name are the study doctor and authorized personnel, who will be able to do so using a list that will be kept securely at the research center.

    Your coded data will be analyzed by researchers in the Department of Critical Care and Intensive Care for study-related activities. The data will be transferred to a computer database and processed to allow the results of this study to be analyzed, reported, and published. When publishing the results of the study, your identity will continue to be kept confidential. Under the Data Protection Law in Colombia 1581 of 2012, the Research Center will be responsible for guaranteeing the protection of your personal information. In the case of transferring your data to other countries where the laws do

not provide the same degree of guarantees and rights regarding data protection as the laws of Colombia, the data will be anonymized before the transfer.

You have the right to review personal information, and to request changes. If you decide to withdraw from the study, the data collected up to that point will continue to be processed, along with other data collected as part of the study.

17. **WHO CAN ANSWER YOUR QUESTIONS:**

If you have questions regarding the project, you can contact the Department of Critical Medicine and Intensive Care of the Fundación Santa Fe de Bogotá:

Principal investigators: Dr. Jorge Iván Alvarado Sánchez, Dr. Laura María Castillo

Telephone: (601) 6030303 Ext. 5889

Address: Carrera 7 # 117 – 15, Fourth floor expansion tower Intensive Care Unit – Adults)

The Corporate Research Ethics Committee of the Fundación Santa Fe de Bogotá has reviewed and approved this project.

If you have any questions or if you believe that your rights have been violated, you can contact the Corporate Research Ethics Committee of the Fundación Santa Fe de Bogotá:

Name of the president: Dr. Klaus Willy Mieth Alviar Telephone: 6030303 Ext 5402

Email: comiteinvestigativo@fsfb.org.co Address: Calle 119ª # 7 – 49

18. **PUBLICATION OF RESULTS:** The results of the study, whether positive, negative, or inconclusive, will be published by the Department of Critical Medicine and Intensive Care of the Fundación Santa Fe de Bogotá following ethical and legal regulations.

19. **DECLARATION OF INFORMED CONSENT**:

I, ______________________ with the type of document: citizenship card ( ), immigration card ( ), passport ( ), No.______________ as patient ( ) or legal representative ( ) of_______________ with document type: citizenship card ( ), card of Immigration ( ), passport ( ) No._____________ Declared that, by signing this informed consent, I certify all the following points:

• I have read (or had read to me) this informed consent form in its entirety and have received explanations about what they are going to do to me and what I am asked to do. I have had the opportunity to ask questions and understand that I may ask other questions about this study at any time.

• I have received a copy of this Consent Report form that I can keep for reference.

• I agree that my confidential personal information is available for review by the Research Group of the Department of Critical Care Medicine and Intensive Care or any health authority, institution, or entity

government assigned to this task in this country or in another country where early multimodal therapy is being considered for approval or, if applicable, by the Institutional Review Board or Ethics Committee.

• I authorize the researcher to have access to the hospital's medical records at any time during the study period.

• I authorize the researcher to process my study data and transfer it if necessary.

• I understand that all personal data will be encrypted and/or anonymized.

• I understand that I am free to withdraw from the study at any time, without justifying my decision and without affecting the medical care I receive, or that the researcher may also withdraw me for any reason to protect my safety.

• I understand that the results, whatever they may be, will be published by the Department of Critical Medicine and Intensive Care of the Fundación Santa Fe de Bogotá.

• I understand that I will be informed of any new information that could affect my willingness to continue participating in this study.

• I voluntarily agree to participate in this study.

• I wish to be informed of my results/my family member's results

YES___ NO ___

Telephone/cell phone: ____

Email: ____

Name of representative: ____

Identity document: ____

Signature of representative: ____

Date: ____/_____/_____

Hour: ____/____

Address: ____

Name of representative: ____

Identity document: ____

Signature of representative: ____________________________________

Date: ____/_____/_____

Hour: ____/____

Relationship: ____

Signature: ____

Date: ____/_____/_____

Hour: ____/____

Name of witness No. 1: ______________________________________

Identity document: ____

Address: ____

Relationship: ____

Signature:____

Date:____/_____/_____

Hour: ____/____

Name of witness No. 2: _______________________________________

Identity document: ____

Address: ____

Relationship: ____

Signature: ____

Date: ____/_____/_____

Hour: ____/____

I hereby certify that I have informed this person(s) in detail about the project. If any additional information arises during the project that could affect the consent given by the representative, I will inform you promptly.

Investigator's name: ____

Identity document: ____

Date: ____/_____/_____

Hour: ____/____

Signature received a copy of the informed consent

Name: ____

Date: _____/______/______

Hour: _____/_____

Annex 3

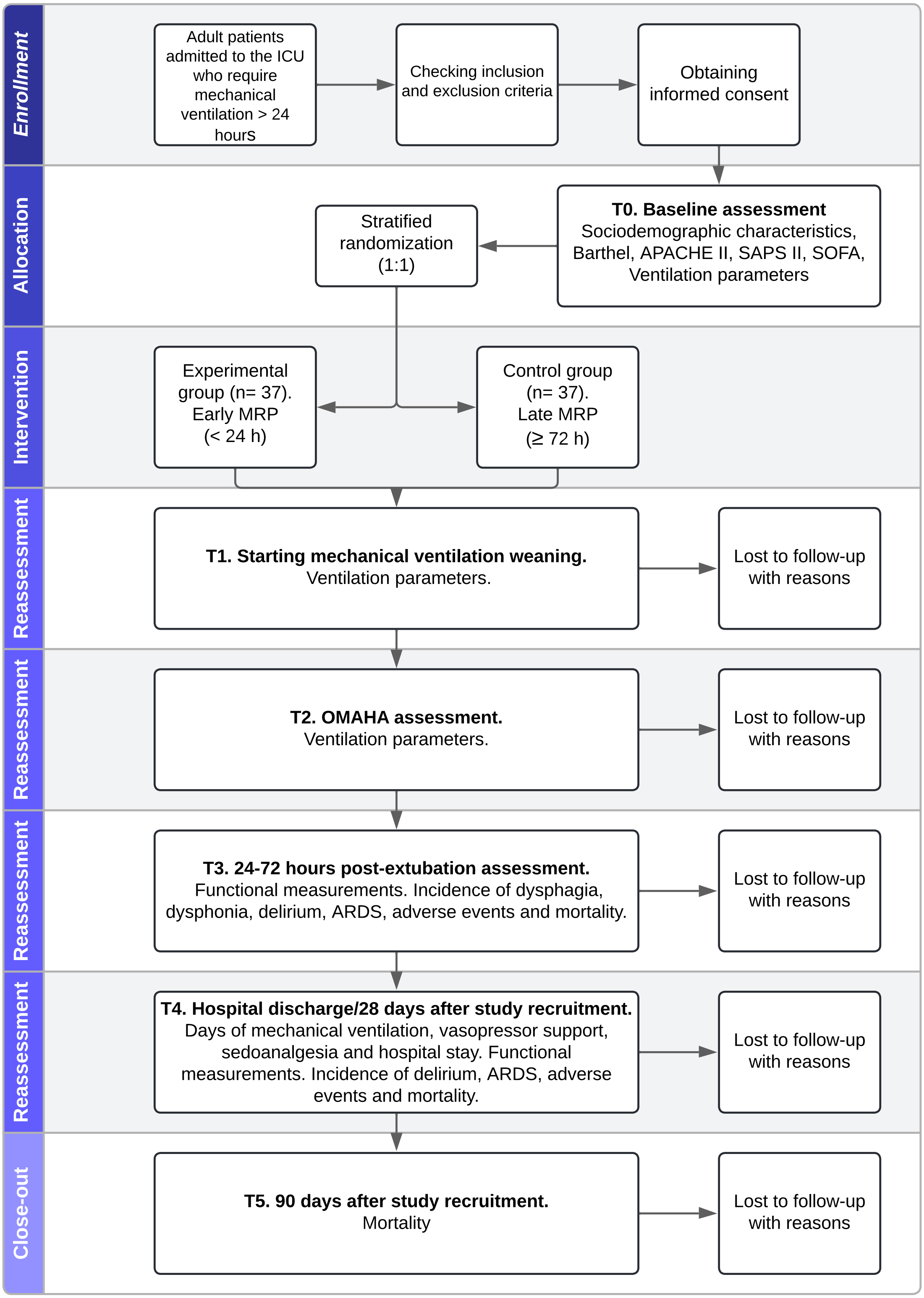

Enrollment
Allocation
Intervention
Reassessment
Reassessment
Reassessment
Reassessment
Close-out
Adult patients admitted to the ICU who require mechanical ventilation > 24 hours
Checking inclusion and exclusion criteria
Obtaining informed consent
T0. Baseline assessment Sociodemographic characteristics, Barthel, APACHE II, SAPS II, SOFA, Ventilation parameters
Stratified randomization (1:1)
Experimental group (n= 37). Early MRP (< 24 h)
Control group (n= 37). Late MRP (≥ 72 h)
T1. Starting mechanical ventilation weaning. Ventilation parameters.
Lost to follow-up with reasons
T2. OMAHA assessment. Ventilation parameters.
Lost to follow-up with reasons
T3. 24-72 hours post-extubation assessment. Functional measurements. Incidence of dysphagia, dysphonia, delirium, ARDS, adverse events and mortality.
Lost to follow-up with reasons
T4. Hospital discharge/28 days after study recruitment. Days of mechanical ventilation, vasopressor support, sedoanalgesia and hospital stay. Functional measurements. Incidence of delirium, ARDS, adverse events and mortality.
Lost to follow-up with reasons
T5. 90 days after study recruitment. Mortality
Lost to follow-up with reasons

ANNEX 4

Time of measures of variables

Start of the study	Start of the weaning	Ending of the weaning	Final of observation
• Age<br>• Weight<br>• Sex<br>• Ethnicity<br>• Index of Barthel<br>• Index of Mass Bodily<br>• Diagnosis<br>• Cluster to the that belong(Early vs late)<br>• Diagnosis<br>• Unit of internship<br>• Punctuation COUCH<br>• Punctuation APACHE II<br>• Punctuation SAPS II<br>• Volume current<br>• Frequency respiratory<br>• PEEP<br>• Pressure plateau<br>• Lung compliancestatic<br>• Pressure of driving<br>• Endurance of the via aerial<br>• Index PaO2/FIO2	• Delta POCC<br>• Pressure muscular<br>• P0.1 (Pressure in 0.1 seconds)<br>• Amount of secretions by tube orotracheal<br>• Index of asynchronies<br>• NIF<br>• Percentage of drain (%drain)<br>• Beak flow of cough<br>• Measurement of excursion diaphragmatic<br>• Measurement of percentageof thickness diaphragmatic	• Delta POCC<br>• Pressure muscular<br>• P0.1<br>• Amount of secretions from tube orotracheal<br>• Index of asynchronies<br>• NIF<br>• Percentage of drain(% drain)<br>• Beak flow of cough<br>• Measuremen t of excursion diaphragmati c<br>• Measurement of percentage of thickness diaphragmatic	• Days of ventilation mechanics<br>• Days free of ventilation mechanics<br>• Days of delirium<br>• Days of hospitalization with delirium<br>• Days of sedation<br>• Days free of sedation<br>• Mortality<br>• Index by Barthel to the egress<br>• Days of stay hospitable<br>• Time of stay in ICU<br>• Mortality<br>• Time from intubation to start of therapy<br>• State of independence function, measured by ADLs to the egress hospitable

			• The state of independencefunction is measured by<br>ADLs to the egress of the ICU<br>• Force muscular measuredwith the scale of Medical<br>Research Council (MRC)<br>• Dysphagia to the 72 hours<br>• Start via oral<br>• The presence of pneumonia, associated with careful health<br>• Mobility maximum extent by JH-HLM<br>• Force prehensile<br>• Failure of extubation<br>• Need of ventilation mechanics No invasive<br>• Mechanic ventilation prolonged<br>• Tracheostomy<br>• Syndrome of difficulty respiratory of the<br>adult<br>• Dysphonia to the 72 hours<br>• OMAHA to the moment of theextubation

ANNEX 5

Format of harvest of data

Data to the income:

Data of the Patient:

- Name of the Patient: ______________________
- Code: ________________(1,2,3...)
- Number of History Clinics: __________________
- Sex_________________
- Ethnicity _________(Afro-Colombian, ROM, Indigenous, Raizal, None)
- Barthel index_________________
- Mass Index Bodily _________________________
- Diagnostic ____________ Code diagnosis CI10.
- Cluster to the that belong______________ (Early vs late)
- Boarding unit _________________(1 = surgical, 2 = neurological, 3 = Septic and Respiratory, 4 = Cardiovascular, 5 = Burns)
- SOFA Score_________________
- APACHE II Score__________________
- Punctuation SAPS II_______________
- Date of Income: __________________
- Date of Egress: __________________
- Time from the Intubation until the Start of the therapy: _________________

Parameters Respiratory:
Variables to the moment of the intubation, these data will be facilitated by respiratory therapy.

- Volume :___________________________
- Frequency:_____________________________
- PEEP (Pressure Positive to the Final of the Expiration): ___________
- Plateau Pressure:________________
- Compliance Pulmonary Static: ____________________
- The pressure of Driving:________________
- Endurance of the Via Air: ___________________
- PaO2/FIO2 index: _________________

During observation:

To register of shape suitable for these data must pass all the days to appreciate the patient.

- Days of Mechanical Ventilation: ______________________________(date of start-end date)

- Days Free from Mechanical Ventilation: _______________(calculated the final of the observation)
- Days of Delirium: ____(start date - end date)
- Days of Hospitalization with Delirium: _______(calculated the final of the observation)
- Sedation Days: ________________(date of start- date of ending)
- Days Free from Sedation: ________________(calculated the final of the observation)
- Prolonged Mechanical Ventilation: ________________(Yes/No)
- Tracheostomy: ________(Yes/No).
- Syndrome of Difficulty Respiratory of the Adult: ______(Yes/No)
- Presence of pneumonia associated with the Careful of health: _____(Yes/No)
- Need dialysis: ________________(Yes/No)
- Medium with norepinephrine _______(Yes/No)
- Support with vasopressin _________(Yes/No)
- Medium with inotrope __________(Yes/No)
- Dose maximum of norepinephrine _____(mcg /kg/min)
- Maximum dose of vasopressin ______(IU/min)

Data start of weaning

The start of the weaning be certain by the therapist

- Delta POCC _____(cmH20)
- Muscle _______________pressure (cmH20)
- P0.1 (Pressure in 0.1 seconds) _____(cmH20)
- Amount of secretions by tube orotracheal ________(Yes/No)
- Index of asynchronies __(%)
- NIF _____(cmH20)
- Percentage of drain ____(% drain)
- Beak flow cough ___(L/min)
- Measurement of excursion diaphragmatic (cm)
- Measurement of percentage of thickness diaphragmatic _(cm)

Data before extubation:

- Delta POCC _____(cmH20)
- Muscle _______________pressure (cmH20)
- P0.1 (Pressure in 0.1 seconds) _____(cmH20)
- Amount of secretions by tube orotracheal ________(Yes/No)
- Index of asynchronies __(%)
- NIF _____(cmH20)
- Percentage of drain ____(% drain)
- Beak flow cough ___(L/min)

- Measurement of excursion diaphragmatic (cm)
- Measurement of percentage of thickness diaphragmatic _(cm)
- OMAHA to the Moment of Extubation: ________________(+/-)

Later to extubation:

- Dysphonia at 72 Hours: ________________(Yes/No)
- Maximum Mobility Extent by JH-HLM at 24 hours: _
- Force Prehensile at 24 hours: _________________
- Need of Ventilation Mechanics No Invasive to the 48 hours: ________(Yes/No)
- Dysphagia to the 72 Hours: _____________(Yes/No)
- Start of Track Oral: ________________(1: 12-24 hours, 2> 24 hours).
- Failure Extubation at 48 hours: ___________________(Yes/No)
- Force Muscular Measured with the scale MRC to the 24 hours: _____________

Final observational:

Functionality and Stay:

- Index by Barthel to the Egress hospitable: ________________
- Function cognitive measured by MOCHA: ______
- Test fast of sensitivity and skill: ______________
- Days of Hospital Stay: __________________(the day when the intensivist informs transfer to the floor).
- Time of Stay in ICU: ______________________________(days).
- Mortality: _____________________________(yes/no)
- State of Independence Functional Measured by ADLs to the Egress Hospitable: ________________
- State of Independence Functional Measured by ADLs to the Egress of the ICU: ________________

Variables of security:

- Outcomes:
    - The number of events adverse.
        - Altered blood pressure __________(Yes/ No) (decrease of PAM > 15%)
        - Cardiac arrhythmia _______(Yes/No)
        - Oxygen desaturation ____________(Yes/ No) (decrease minor at 80%)
        - Pain either agitation_______ (Yes/No)
        - Removal of line invasive _________ (Yes/No)

    - Gastrointestinal (nausea, threw up either diarrhea) ___(Yes/No)
    - Tachypnea ________(Yes/No)
    - State neurological altered ___________(Yes/No)
- Number of events adverse serious__________
- Extubation not scheduled __________(Yes/No)
- Pneumonia associated with careful health _________(Yes/No)
- Bronchoaspiration ________(Yes/No)
