# Supplementary material for: Efficacy and safety of the early implementation of a multimodal rehabilitation program in mechanically ventilated patients: A randomized clinical trial protocol
Source: PLoS One. 2025 May 19;20(5):e0324335. doi: 10.1371/journal.pone.0324335 (PMC12088510; doi:10.1371/journal.pone.0324335)
Supplement: S4 File — (PDF) [file pone.0324335.s004.pdf]

FUNDACIÓN SANTA FE DE BOGOTÁ
COMITÉ CORPORATIVO DE ÉTICA
E INVESTIGACIÓN

22 ABR 2024

APROBADO

Firma: Dr. Klaus Mieth A.
Presidente

Fundación
Santa Fe de Bogotá

Asociación entre terapia multimodal temprana y días de ventilación mecánica en la unidad de cuidado intensivos de la Fundación Santa Fe de Bogotá: un ensayo clínico controlado aleatorizado.

**Título del estudio**	Asociación entre terapia multimodal temprana y días de ventilación mecánica en la unidad de cuidado intensivos de la Fundación Santa Fe de Bogotá: un ensayo clínico controlado aleatorizado.
**Nombre del Investigador Principal**	Jorge Iván Alvarado Sánchez<br>Laura María Castillo Morales
**Teléfono de contacto con el investigador**	6030303 ext. 5889
**Nombre de la Institución de Investigación**	Fundación Santa Fe de Bogotá
**Dirección de la Institución de Investigación**	Calle 119A No.7-49, Cuarto piso torre de expansión UCI adultos
**Versión y fecha del consentimiento**	Versión 2.0 del 05 de abril de 2024
**Número codificado del participante**	

La Fundación Santa Fe de Bogotá (FSFB) y el Departamento de Medicina Crítica y Cuidado Intensivo lo están invitando a participar / están invitando a su familiar a participar como voluntario en un proyecto en el que se compara la terapia multimodal (terapia física, ocupacional, respiratoria y fonoaudiología) temprana frente a la terapia multimodal tardía en pacientes con ventilación mecánica invasiva en la Unidad de Cuidado Intensivo de la FSFB.

Este documento de Consentimiento Informado le proporcionará la información necesaria para ayudarle a decidir a usted y/o su familiar sobre su participación en el estudio. Por favor lea atentamente la información. Si cualquier parte de este documento no le resulta claro o si tiene alguna pregunta o desea solicitar información adicional, no dude en pedirla en cualquier momento a alguno de los miembros del equipo de estudio, quienes se mencionan al final de este documento.

1. **NATURALEZA Y PROPÓSITO DEL ESTUDIO:** Este estudio tiene como objetivo evaluar la diferencia en días de ventilación mecánica invasiva entre la terapia multimodal temprana y la terapia multimodal tardía (manejo estándar) en la

FUNDACIÓN SANTA FE DE BOGOTÁ
COMITÉ CORPORATIVO DE ÉTICA
E INVESTIGACIÓN
22 ABR 2024
APROBADO
Firma: Dr. Klaus Mieth A.
Presidente

unidad de cuidado intensivo adulto de la Fundación Santa Fe de Bogotá. La ventilación mecánica es un tratamiento utilizado cuando una persona tiene dificultades para respirar por sí misma. Se trata de una maquina especializada similar a un "pulmón, pero fuera del cuerpo" que ayuda a una persona a respirar al inflar y desinflar sus pulmones de manera controlada. Este proceso se realiza a través de un tubo colocado en la garganta (también conocido como tubo endotraqueal) o mediante una máscara que se coloca sobre la boca y la nariz.

2. **¿QUIÉN PUEDE PARTICIPAR?:** Para participar usted / su familiar debe cumplir con los siguientes criterios:

   - Personas mayores de 18 años
   - El participante debe estar hospitalizado en la Unidad de Cuidado Intensivo de la Fundación Santa Fe de Bogotá
   - El participante debe requerir ventilación mecánica invasiva por más de 24 horas (un día) posterior al ingreso en la Unidad.
   - El participante debe ser una persona con alta capacidad funcional, la cual será medida mediante una herramienta conocida como índice de Barthel (la cual es una escala que se usa por personal de salud para objetivar que tan funcional es una persona en su día a día y puntua de 0 a 100). Para esta investigación, el índice debe ser mayor a 70 puntos (siendo 100 una independencia total para las tareas de la vida diaria y 0 una dependencia total de la persona a los cuidados de un tercero).

3. **TRATAMIENTOS ALTERNATIVOS VENTAJOSOS PARA EL SUJETO:** El médico tratante determinara si hay otros tratamientos de los que se pueda beneficiar el paciente o si el paciente no requiere todas las intervenciones de la terapia multimodal, sino un subgrupo de ellas. Su participación en este estudio contribuirá al avance del conocimiento médico en este campo.

4. **DURACIÓN ESPERADA DE PARTICIPACIÓN Y NÚMERO DE SUJETOS:** Su participación durará 90 días a partir del momento en que requiere ventilación mecánica invasiva. Comprendemos que probablemente el tiempo de ventilación mecánica no sea de 90 días y que puede que ya se encuentre en casa con su familia, por lo que el seguimiento que está previsto para este estudio será telefónico por lo que no tendrá que trasladarse nuevamente a la institución para ello. El número total de participantes será de 74.

5. **PROCEDIMIENTOS DEL ESTUDIO:** Existen dos grupos en este proyecto: un grupo de terapia multidisciplinaria temprana, la cual se define como el conjunto de maniobras especializadas que realiza el grupo de fisioterapia, fonoaudiología, terapia respiratoria y terapia ocupacional a partir de las primeras 24 horas que se realiza la intubación y se inicia la ventilación mecánica. El segundo grupo corresponde a la terapia multidisciplinar tardía, la cual consiste en las mismas

FUNDACIÓN SANTA FE DE BOGOTÁ
COMITÉ CORPORATIVO DE ÉTICA
E INVESTIGACIÓN

22 ABR 2024

APROBADO

Firma: Dr. Klaus Mieth A.
Presidente

intervenciones que el primer grupo, no obstante, el tiempo de inicio será a partir de las 72 horas que se realiza la intubación y se inicie la ventilación mecánica. Se realizará un monitoreo continuo del estado suyo o de su familiar como se hace de rutina en la UCI. La decisión de retirar la terapia multidisciplinar será tomada por el equipo médico a cargo en caso tal que estas terapias no sean en el mejor interés suyo o de su familiar.

6. **MANEJO DE MUESTRAS BIOLÓGICAS OBTENIDAS:** No se tomarán muestras biológicas específicamente para este proyecto. Todas las muestras biológicas que se tomen corresponderán al tratamiento habitual de los pacientes de la Unidad de Cuidado Intensivo concertado con el grupo médico tratante.

7. **¿QUÉ SE ESPERA DE SU PARTICIPACIÓN?:** Se espera que participe y colabore en el desarrollo del estudio, siguiendo las instrucciones del equipo médico.

8. **¿QUÉ PASARÁ AL FINAL DEL ESTUDIO?:** Al final del estudio, se le proporcionará información detallada sobre los resultados si así lo desea. También tendrá la oportunidad de discutir cualquier pregunta o inquietud que pueda tener. En este informe de consentimiento informado podrá marcar si desea conocer esta información. En caso tal que marque "SI", se le será comunicada a usted o a su familiar por medio telefónico o por correo electrónico una vez finalice el estudio.

9. **POSIBLES EFECTOS ADVERSOS:** Existen posibles riesgos asociados con la participación en el estudio, incluyendo efectos secundarios de las diferentes terapias y posibles incomodidades. Los efectos adversos conocidos son:

   - **Alteración de la presión arterial:** algunos participantes podrían experimentar una disminución o aumento temporal de la presión arterial durante la realización de las terapias, lo que podría causar mareos o desmayos.

   - **Arritmias Cardiacas (cambios en el ritmo normal del corazón):** en raras ocasiones, los participantes podrían experimentar cambios en el ritmo cardíaco, lo que podría causar palpitaciones o sensación de irregularidad en los latidos del corazón.

   - **Desaturación de oxígeno (disminución de la cantidad de oxigeno que se transporta en el cuerpo):** en algunos casos, los participantes podrían experimentar disminución de la cantidad de oxígeno en la sangre, lo que podría resultar en confusión, mareos o sensación de ahogo y fatiga.

   - **Dolor o agitación (hiperactividad corporal o una sensación de desespero):** algunos participantes podrían experimentar dolor o

FUNDACIÓN SANTA FE DE BOGOTÁ
COMITÉ CORPORATIVO DE ETICA
E INVESTIGACIÓN

22 ABR 2024

APROBADO

Firma: Dr. Klaus Mieth A.
Presidente

agitación debido a la intensidad o condición de base del participante el cual puede aumentar al realizar las actividades correspondientes a cada terapia.

- **Remoción de línea invasiva (como venas o arterias canalizadas para colocación de líquidos o medicamentos):** aunque es poco probable, algunas personas podrían removerse accidentalmente líneas invasivas (líquidos endovenosos, nutrición parenteral, bombas de infusión). Esto implicaría que sería necesario volver a establecer estas líneas invasivas.

- **Taquipnea (aumento de la frecuencia en la que se respira):** dado que estas terapias implican actividad física, se espera que haya un aumento de la frecuencia respiratoria. En algunos casos, los participantes podrían experimentar sensación de ahogo o fatiga debido al aumento del esfuerzo físico.

- **Deterioro neurológico:** en casos excepcionales, los participantes podrían experimentar cambios en la función cerebral, lo que podría resultar en confusión, mareos persistentes o dificultades para hablar.

- **Otros efectos secundarios:** además de los mencionados, pueden ocurrir otros efectos secundarios no previstos debido a la complejidad de los tratamientos médicos y la condición de base del paciente. Estos serán monitoreados cuidadosamente y tratados según sea necesario.

10. **RIESGOS Y BENEFICIOS:** Los riesgos incluyen posibles efectos secundarios de los procedimientos médicos y las terapias explicados en el punto 9. Los beneficios incluyen contribuir al avance del conocimiento médico y posiblemente mejorar el tratamiento de pacientes con ventilación mecánica invasiva en el futuro.

11. **INFORMACIÓN NUEVA DEL ESTUDIO:** Su médico del estudio le comunicará oportunamente toda información nueva obtenida durante el estudio que pueda afectar su voluntad de continuar participando. Cuando se le comunique esta información nueva, se le pedirá que firme y feche un nuevo formulario de consentimiento, si acepta continuar en el estudio.

12. **QUÉ MÁS NECESITA SABER ANTES DE DECIDIR PARTICIPAR:** usted recibirá una copia de este formato de Consentimiento Informado, consérvela en un lugar seguro y utilícela como información y referencia durante todo el desarrollo

FUNDACIÓN SANTA FE DE BOGOTÁ
COMITÉ CORPORATIVO DE ÉTICA
E INVESTIGACIÓN
22 ABR 2024
APROBADO
Firma: Dr. Klaus Mieth A.
Presidente

del estudio. Esta investigación se llevará acabo de acuerdo con la resolución 8430 de 1993 y 2378 de 2008 del Ministerio de Salud colombiano. Este documento fue revisado y aprobado por el Comité Corporativo de Ética en Investigación y cumple con todos los requerimientos metodológicos y éticos para ser desarrollado.

Ni usted ni el Departamento de Medicina Crítica y Cuidado Intensivo recibirán compensación económica por participar en este estudio.

13. **PUEDEN EXISTIR RAZONES POR LAS CUALES USTED NO PUEDA PARTICIPAR:** su participación en este estudio es absolutamente voluntaria. No está obligado a participar y puede retirar su participación en cualquier momento sin que esto implique sanciones ni la pérdida de los beneficios a los que tiene derecho. Si decide abandonar el estudio antes de la última visita del estudio, infórmelo al médico del estudio y siga sus instrucciones.

14. **CUBRIMIENTO DE EVENTOS ADVERSOS / COMPENSACIÓN:** dado que la aplicación de la terapia multimodal hace parte de nuestra práctica médica habitual, las complicaciones relacionadas con ellas no requieren una póliza adicional debido a que se asocian directamente con la terapia y la condición médica de base. Al firmar este consentimiento usted no renuncia a ninguno de sus derechos legales que pudiese corresponderle en caso de que se produzca algún daño, y que se acredite que el mismo se haya producido como consecuencia directa de la terapia multimodal y su participación en el estudio. El investigador empeñaraátodos sus esfuerzos para evitar que se produzca algún daño.

15. **QUÉ OCURRIRÁ SI DECIDE NO PARTICIPAR O SI CAMBIA DE IDEA:** La participación en este estudio es totalmente voluntaria, usted no está obligado a participar, puede retirarse en cualquier momento sin justificar su decisión, sin sufrir ninguna sanción o detrimento en la atención por parte de su médico ni de la Institución o puede también ser retirado por su investigador por alguna razón que él le explicará, pero en cualquiera de los dos casos se le realizarán pruebas o procedimientos para terminar su participación de manera ordenada.

16. **CONFIDENCIALIDAD Y PRIVACIDAD DE DATOS:** El investigador asegurará la confidencialidad de su historia clínica, en la cual no se identificará al sujeto, se mantendrá la confidencialidad de la información relacionada con su privacidad, utilizando códigos hasta donde las leyes y regulaciones lo permitan y no serán

FUNDACIÓN SANTA FE DE BOGOTÁ
COMITÉ CORPORATIVO DE ÉTICA
E INVESTIGACIÓN
22 ABR 2024
APROBADO
Firma: Dr. Klaus Mieth A.
Presidente

accesibles públicamente. Los datos obtenidos podrán ser consultados por autoridades sanitarias, autoridades de salud nacional Instituto Nacional de medicamentos y Alimentos- INVIMA- y Comité de Ética en Investigación.

El centro del estudio registrará la información personal básica sobre usted, como su nombre, información de contacto, sexo, estatura, peso y etnia, así como también información sobre sus antecedentes médicos y los datos clínicos recopilados acerca de su participación en el estudio. Todo el personal con acceso a sus registros está obligado a respetar su confidencialidad en todo momento.

Para garantizar su privacidad, no se incluirá su nombre ni ninguna otra información que lo identifique directamente en los registros entregados para los fines de la investigación. Los únicos que podrán vincular este código con su nombre son el médico del estudio y el personal autorizado, quienes podrán hacerlo mediante una lista que se conservará de forma segura en el centro de investigación.

Sus datos codificados serán analizados por los investigadores del Departamento de Medicina Crítica y Cuidado Intensivo para actividades relacionadas con el estudio. Los datos se transferirán a una base de datos informática y se procesarán para permitir que los resultados de este estudio se analicen, informen y publiquen. Al publicar los resultados del estudio, se seguirá manteniendo la confidencialidad de su identidad. En virtud de la Ley de protección de datos en Colombia 1581 de 2012, el Centro de investigación sera responsable de garantizar la protección de su información personal. En el caso de transferir sus datos a otros países en los que las leyes no proporcionen el mismo grado de garantías y derechos en materia de protección de datos que las leyes de Colombia, los datos serán anonimizados antes de la transferencia.

Usted tiene derecho a revisar la información personal, a solicitar cambios. Si decide retirarse del estudio, los datos recopilados hasta ese momento podrán seguir procesándose, junto con otros datos recopilados como parte del estudio.

17. QUIÉNES PUEDEN CONTESTAR SUS PREGUNTAS:

En caso de presentar dudas respecto al proyecto, se puede contactar al Departamento de Medicina Crítica y Cuidado Intensivo de la Fundación Santa Fe de Bogotá:

Investigadores principales: Dr. Jorge Iván Alvarado Sánchez, Dra. Laura María Castillo
Telefono: (601) 6030303 Ext. 5889

FUNDACIÓN SANTA FE DE BOGOTÁ
COMITÉ CORPORATIVO DE ÉTICA
E INVESTIGACIÓN

22 ABR 2024

APROBADO

Firma: Dr. Klaus Mieth A.
Presidente

Fundación
Santa Fe de Bogotá

Dirección: Carrera 7 # 117 – 15, Cuarto piso torre de expansión Unidad de Cuidados Intensivos – Adultos)

El Comité Corporativo de Ética en Investigación de la Fundación Santa Fe de Bogotá han revisado y aprobado este proyecto.
Si usted tiene alguna duda o si cree que sus derechos han sido vulnerados, puede comunicarse con el Comité Corporativo de Ética en Investigación de la Fundación Santa Fe de Bogotá:

Nombre del presidente: Dr. Klaus Willy Mieth Alviar
Telefono: 6030303 Ext 5402
Correo: comiteinvestigativo@fsfb.org.co
Dirección: Calle 119ª # 7 – 49

18. **PUBLICACIÓN DE RESULTADOS:** Los resultados del estudio, ya sean positivos, negativos o inconclusos, serán publicados por el Departamento de Medicina Crítica y Cuidado Intensivo de la Fundación Santa Fe de Bogotá de acuerdo con las normativas éticas y legales.

19. **DECLARACIÓN DE CONSENTIMIENTO INFORMADO:**

Yo: ________________________________ con tipo de documento: cédula de ciudadanía ( ), cédula de extranjería ( ), pasaporte ( ), No. ______________ en calidad de paciente ( ) o representante legal ( ) de:______________________ con tipo de documento: cédula de ciudadanía ( ), cédula de extranjería ( ), pasaporte ( ) No.____________ declaró que, al firmar este consentimiento informado, certifico todos los siguientes puntos:

- He leído (o me han leído) este formulario de consentimiento informado en su totalidad y he recibido explicaciones sobre lo que me van a hacer y lo que se me pide que haga. He tenido la oportunidad de hacer preguntas y entiendo que puedo hacer otras preguntas sobre este estudio en cualquier momento.

- He recibido una copia de este formulario de Informe de Consentimiento que puedo guardar como referencia.

- Acepto que mi información personal confidencial esté disponible para que la revisen: el Grupo de Investigación del Departamento de Medicina Crítica y Cuidado Intensivo o cualquier autoridad de salud, institución o entidad

FUNDACION SANTA FE DE BOGOTÁ
COMITÉ CORPORATIVO DE ÉTICA
E INVESTIGACIÓN

22 ABR 2024

APROBADO

Firma: Dr. Klaus Mieth A.
Presidente

gubernamental asignada a esta tarea en este país o en otro país donde la terapia multimodal temprana se esté considerando para su aprobación o, si corresponde, por el Comité de Revisión Institucional o el Comité de Ética.

- Autorizo que el investigador tenga acceso a los registros médicos del hospital en cualquier momento durante el periodo del estudio.
- Autorizo al investigador a procesar mis datos del estudio y a transferirlos si es necesario.
- Entiendo que todos los datos personales serán codificados y/o anonimizadas.
- Entiendo que tengo la libertad de retirarme del estudio en cualquier momento, sin justificar mi decisión y sin afectar la atención médica que reciba, o que también el investigador puede retirarme por alguna razón en protección a mi seguridad
- Entiendo que los resultados cualesquiera que sean serán publicados por el Departamento de Medicina Crítica y Cuidado Intensivo de la Fundación Santa Fe de Bogotá.
- Comprendo que se me informará sobre cualquier información nueva que pudiera afectar mi voluntad de seguir participando en este estudio.
- Acepto voluntariamente participar en este estudio.
- Deseo que se me comuniquen mis resultados / los resultados de mi familiar

  SI __ NO __

  Telefono / celular: ____

  Correo electrónico: ____

Nombres y apellidos del participante:______________________________

Documento de identificación:______________________________

Firma del participante:____________________

Fecha: ____/____/____, Hora: ____:____

Fundación
Santa Fe de Bogotá

FUNDACIÓN SANTA FE DE BOGOTÁ
COMITÉ CORPORATIVO DE ÉTICA
E INVESTIGACIÓN

22 ABR 2024

APROBADO

Firma: Dr. Klaus Mieth A.
Presidente

Dirección de residencia:______________________________

Nombre del representante: ______________________________

Documento identificación: ______________________________

Firma del representante:______________________

Fecha: ____/____/____, Hora: ____:____

Dirección: ____________________________

Parentesco: ________________

Firma:________________________________

Fecha: ____ /____ /____ Hora: __:__

Nombre del testigo No. 1: ______________________________

Documento identificación:______________________________

Dirección: ______________________________

Parentesco: ______________________________

Firma:_________________________

Fecha: __ /__ /__ Hora: _:__

Nombre del testigo No. 2: ______________________________

Documento identificación: ______________________________

Dirección: ______________________________

Relación con el paciente: ______________________________

Firma: _________________________

Fundación
Santa Fe de Bogotá

FUNDACIÓN SANTA FE DE BOGOTÁ
COMITÉ CORPORATIVO DE ÉTICA
E INVESTIGACIÓN

22 ABR 2024

APROBADO

Firma: Dr. Klaus Mieth A.
Presidente

Fecha: ____ /____ /____ Hora: __:__

Por la presente certifico que he informado detalladamente a esta/s persona/s sobre el proyecto.

Si alguna información adicional surge durante el proyecto, de modo que pudiera afectar al consentimiento dado por el representante, le informaré de manera oportuna.

Nombre del investigador:________________________________

Documento identificación: ______________________________

Firma del investigador:____________________

Fecha: ____/____ /____ Hora: __:__

Firma recibido copia del consentimiento informado
____
Nombre: Fecha: __/__ /__ Hora: __:__
