# Supplementary material for: Efficacy and safety of the early implementation of a multimodal rehabilitation program in mechanically ventilated patients: A randomized clinical trial protocol
Source: PLoS One. 2025 May 19;20(5):e0324335. doi: 10.1371/journal.pone.0324335 (PMC12088510; doi:10.1371/journal.pone.0324335)
Supplement: S5 File — (PDF) [file pone.0324335.s005.pdf]

Association between therapy multimodal early and days of ventilation mechanicsin the intensive care unit of the Fundación Santa Fe de Bogotá: A rehearsal clinical checked randomized.

**Qualification of the study**	Association between multimodal therapy early and days of ventilation mechanics in the intensive care unit of the Fundación Santa Fe de Bogotá: a rehearsal clinical checked randomized.
**Name of the Main Investigator**	Jorge Ivan Alvarado Sanchez<br>Laura Maria Castle Morales
**Phone of contact with the investigator**	6030303 ext. 5889
**Name of the Institution of Investigation**	Fundación Santa Fe de Bogotá
**Address of the Institution of Investigation**	Street 119A No.7-49, Room floor tower of expansion ICU Adults
**Version and date of the consent**	Version 2.0 of the 05 April 2024
**Number encoded by the competitor**	

The Fundación Santa Fe de Bogotá (FSFB) and the Department of Critical Care Medicine Intensive Care are inviting you to participate/are inviting your family member to participate as a volunteer in a project that compares multimodal therapy (physical, occupational, respiratory, and speech therapy) versus early therapylate multimodality in patients with invasive mechanical ventilation in the Emergency Unit Careful Intensive of the FSFB.

This Informed Consent document will provide you with the information necessary to help you and/or your family member decide whether to participate in the study. Please read the information carefully. If any part of this document is not clear to you or if you have any questions or would like to request additional information, please do not hesitate to ask any of the members of the study team, who are listed at the end of this document, at any time.

1. **NATURE AND PURPOSE OF THE STUDY:** This study aims to evaluate the difference in days of invasive mechanical ventilation between early multimodal therapy and late multimodal therapy (standard management) in the

adult intensive care unit of the Fundación Santa Fe de Bogotá. Mechanical ventilation is a treatment used when a person has difficulty breathing on their own. It is a specialized machine like a “lung, but outside the body” that helps a person breathe by inflating and deflating their lungs in a controlled manner. This process is done through a tube placed in the throat (also known as an endotracheal tube) or through a mask that fits over the mouth and nose.

2. **WHO CAN PARTICIPATE?** To participate you/your family must meet the following criteria:

- People over 18 years of age
- The participant must be hospitalized in the Intensive Care Unit of the Fundación Santa Fe de Bogotá.
- The participant must require invasive mechanical ventilation for more than 24 hours (one day) after admission to the Unit.
- The participant must be a person with high functional capacity, which will be measured using a tool known as the Barthel index (which is a scale used by health personnel to measure how functional a person is in their daily life). and score from 0 to 100). For this research, the index must be greater than 70 points (100 being total independence for daily life tasks and 0 being total dependence of the person on the care of a third party).

3. **ALTERNATIVE TREATMENTS ADVANTAGEOUS FOR THE SUBJECT:** The treating physician will determine if there are other treatments from which the patient can benefit or if the patient does not require all the interventions of multimodal therapy, but rather a subgroup of them. Its participation in this study will contribute to the advancement of medical knowledge in this field.

4. **EXPECTED DURATION OF PARTICIPATION AND NUMBER OF SUBJECTS:** Your participation will last 90 days from the time you require invasive mechanical ventilation. We understand that the duration of mechanical ventilation is probably not 90 days and that you may already be at home with your family, so the follow-up planned for this study will be by telephone so you will not have to travel to the institution again. for it. The total number of participants will be 74.

5. **STUDY PROCEDURES:** There are two groups in this project: an early multidisciplinary therapy group, which is defined as the set of specialized maneuvers carried out by the physiotherapy, speech therapy, respiratory therapy and occupational therapy group starting in the first 24 hours. hours intubation is performed, and mechanical ventilation begins. The second group corresponds to late multidisciplinary therapy, which consists of the same

interventions as the first group, however, the start time will be 72 hours after intubation is performed and mechanical ventilation begins. Continuous monitoring of your or your family member's condition will be carried out as is routinely done in the ICU. The decision to withdraw multidisciplinary therapy will be made by the medical team in charge in the event that these therapies are not in the best interest of you or your family member.

6. **HANDLING OF BIOLOGICAL SAMPLES OBTAINED:** No biological samples will be taken specifically for this project. All biological samples taken will correspond to the usual treatment of Intensive Care Unit patients agreed with the treating medical group.

7. **WHAT IS EXPECTED FROM YOUR PARTICIPATION?** You are expected to participate and collaborate in the development of the study, following the instructions of the medical team.

8. **WHAT WILL HAPPEN AT THE END OF THE STUDY?** At the end of the study, you will be provided with detailed information about the results if you wish. You will also discuss any questions or concerns you may have. In this informed consent report you can check if you want to know this information. If you check "YES" you or your family member will be notified by telephone or email once the study is completed.

9. **POSSIBLE ADVERSE EFFECTS:** There are possible risks associated with participation in the study, including side effects of the different therapies and possible discomfort. Recognized adverse effects are:

- **Altered blood pressure**: Some participants may experience a temporary decrease or increase in blood pressure while taking the therapies, which could cause dizziness or fainting.

- **Cardiac Arrhythmias (changes in the normal heart rhythm):** In rare cases, participants may experience changes in heart rhythm, which could cause palpitations or a feeling of irregular heartbeats.

- **Oxygen desaturation (decrease in the amount of oxygen carried in the body):** In some cases, participants may experience a decrease in the amount of oxygen in their blood, which could result in confusion, dizziness, or a feeling of shortness of breath and fatigue.

- **Pain or agitation (body hyperactivity or a feeling of hopelessness):** some participants may experience pain or agitation due to the intensity or base condition of the participant which may increase when performing the activities corresponding to each therapy.

- **Removal of invasive lines (such as veins or arteries channeled for placement of fluids or medications):** although unlikely, some people may accidentally remove invasive lines (intravenous fluids, parenteral nutrition, infusion pumps). This would imply that these invasive lines would need to be re-established.

- **Tachypnea (increased breathing rate):** Since these therapies involve physical activity, an increased breathing rate is expected. In some cases, participants may experience shortness of breath or fatigue due to increased physical exertion.

- **Neurological impairment:** In rare cases, participants may experience changes in brain function, which may result in confusion, persistent dizziness, or difficulty speaking.

- **Other side effects:** in addition to those mentioned, other unforeseen side effects may occur due to the complexity of medical treatments and the patient's underlying condition. These will be carefully monitored and treated as necessary.

10. **RISKS AND BENEFITS**: Risks include possible side effects of the medical procedures and therapies explained in point 9. Benefits include contributing to the advancement of medical knowledge and possibly improving the treatment of patients with invasive mechanical ventilation in the future.

11. **NEW STUDY INFORMATION**: Your study doctor will inform you promptly of any new information obtained during the study that may affect your willingness to continue participating. When you are told this new information, you will be asked to sign and date a new consent form if you agree to continue in the study.

12. **WHAT ELSE YOU NEED TO KNOW BEFORE DECIDING TO PARTICIPATE**: you will receive a copy of this Informed Consent form, keep it in a safe place, and use it as information and reference throughout the development of the study. This research will be carried out following resolution 8430 of 1993 and 2378 of 2008 of the Colombian Ministry of Health. This document was reviewed and approved by the Corporate Research Ethics Committee and meets all the methodological and ethical requirements to be developed.

Neither you nor the Department of Critical Care Medicine will receive financial compensation for participating in this study.

13. **THERE MAY BE REASONS WHY YOU CANNOT PARTICIPATE**: Your participation in this study is voluntary. You are not required to participate and may withdraw your participation at any time without penalty or loss of benefits to which you are entitled. If you decide to leave the study before the last study visit, inform the study doctor and follow his or her instructions.

14. **COVERAGE OF ADVERSE EVENTS/COMPENSATION**: since the application of multimodal therapy is part of our normal medical practice, complications related to them do not require an additional policy because they are directly associated with the therapy and the underlying medical condition. By signing this consent, you do not waive any of your legal rights that may apply to you if any damage occurs, and it is proven that it has occurred as a direct consequence of the multimodal therapy and your participation in the study. The investigator will make every effort to prevent any harm from occurring.

15. **WHAT WILL HAPPEN IF YOU DECIDE NOT TO PARTICIPATE OR IF YOU CHANGE YOUR MIND**: Participation in this study is completely voluntary, you are not obliged to participate, and you can withdraw at any time without justifying your decision, without suffering any penalty or detriment to care. Part of your doctor or the Institution or you may also be withdrawn by your researcher for some reason that he will explain to you, but in either case tests or procedures will be performed to end your participation in an orderly manner.

16. **CONFIDENTIALITY AND PRIVACY OF DATA**: The researcher will ensure the confidentiality of your clinical history, in which the subject will not be identified, the confidentiality of the information related to your privacy will be maintained, using codes to the extent permitted by laws and regulations and They will not be publicly accessible. The data obtained may be consulted by health authorities, national health authorities, the National Institute of Medicines and Foods - INVIMA - and the Research Ethics Committee.

    The study center will record basic personal information about you, such as your name, contact information, gender, height, weight, and ethnicity, as well as also information about your medical history and clinical data collected about your participation in the study. All personnel with access to your records are obliged to always respect their confidentiality.

    To ensure your privacy, your name or other information that directly identifies you will not be included in the records provided for research purposes. The only people who will be able to link this code to your name are the study doctor and authorized personnel, who will be able to do so using a list that will be kept securely at the research center.

    Your coded data will be analyzed by researchers in the Department of Critical Care and Intensive Care for study-related activities. The data will be transferred to a computer database and processed to allow the results of this study to be analyzed, reported, and published. When publishing the results of the study, your identity will continue to be kept confidential. Under the Data Protection Law in Colombia 1581 of 2012, the Research Center will be responsible for guaranteeing the protection of your personal information. In the case of transferring your data to other countries where the laws do

not provide the same degree of guarantees and rights regarding data protection as the laws of Colombia, the data will be anonymized before the transfer.

You have the right to review personal information, and to request changes. If you decide to withdraw from the study, the data collected up to that point will continue to be processed, along with other data collected as part of the study.

17. **WHO CAN ANSWER YOUR QUESTIONS:**

If you have questions regarding the project, you can contact the Department of Critical Medicine and Intensive Care of the Fundación Santa Fe de Bogotá:

Principal investigators: Dr. Jorge Iván Alvarado Sánchez, Dr. Laura María Castillo

Telephone: (601) 6030303 Ext. 5889

Address: Carrera 7 # 117 – 15, Fourth floor expansion tower Intensive Care Unit – Adults)

The Corporate Research Ethics Committee of the Fundación Santa Fe de Bogotá has reviewed and approved this project.

If you have any questions or if you believe that your rights have been violated, you can contact the Corporate Research Ethics Committee of the Fundación Santa Fe de Bogotá:

Name of the president: Dr. Klaus Willy Mieth Alviar Telephone: 6030303 Ext 5402

Email: comiteinvestigativo@fsfb.org.co Address: Calle 119ª # 7 – 49

18. **PUBLICATION OF RESULTS:** The results of the study, whether positive, negative, or inconclusive, will be published by the Department of Critical Medicine and Intensive Care of the Fundación Santa Fe de Bogotá following ethical and legal regulations.

19. **DECLARATION OF INFORMED CONSENT**:

I, ______________________ with the type of document: citizenship card ( ), immigration card ( ), passport ( ), No.______________ as patient ( ) or legal representative ( ) of_______________ with document type: citizenship card ( ), card of Immigration ( ), passport ( ) No._____________ Declared that, by signing this informed consent, I certify all the following points:

• I have read (or had read to me) this informed consent form in its entirety and have received explanations about what they are going to do to me and what I am asked to do. I have had the opportunity to ask questions and understand that I may ask other questions about this study at any time.

• I have received a copy of this Consent Report form that I can keep for reference.

• I agree that my confidential personal information is available for review by the Research Group of the Department of Critical Care Medicine and Intensive Care or any health authority, institution, or entity

government assigned to this task in this country or in another country where early multimodal therapy is being considered for approval or, if applicable, by the Institutional Review Board or Ethics Committee.

• I authorize the researcher to have access to the hospital's medical records at any time during the study period.

• I authorize the researcher to process my study data and transfer it if necessary.

• I understand that all personal data will be encrypted and/or anonymized.

• I understand that I am free to withdraw from the study at any time, without justifying my decision and without affecting the medical care I receive, or that the researcher may also withdraw me for any reason to protect my safety.

• I understand that the results, whatever they may be, will be published by the Department of Critical Medicine and Intensive Care of the Fundación Santa Fe de Bogotá.

• I understand that I will be informed of any new information that could affect my willingness to continue participating in this study.

• I voluntarily agree to participate in this study.

• I wish to be informed of my results/my family member's results

YES___ NO ___

Telephone/cell phone: ____

Email: ____

Name of representative: ____

Identity document: ____

Signature of representative: ____

Date: _____/______/______

Hour: _____/_____

Address: ____

Name of representative: ____

Identity document: ____

Signature of representative: ______________________________________

Date: _____/______/______

Hour: _____/_____

Relationship: ____

Signature: ____

Date: _____/______/______

Hour: _____/_____

Name of witness No. 1: ____

Identity document: ____

Address: ____

Relationship: ____

Signature:____

Date:____/_____/_____

Hour: ____/____

Name of witness No. 2: ____

Identity document: ____

Address: ____

Relationship: ____

Signature: ____

Date: ____/_____/_____

Hour: ____/____

I hereby certify that I have informed this person(s) in detail about the project. If any additional information arises during the project that could affect the consent given by the representative, I will inform you promptly.

Investigator's name: ____

Identity document: ____

Date: ____/_____/_____

Hour: ____/____

Signature received a copy of the informed consent

Fundación
Santa Fe de Bogotá

Name: ____

Date: _____/______/______

Hour: _____/_____
