# Supplementary material for: Efficacy and safety of the early implementation of a multimodal rehabilitation program in mechanically ventilated patients: A randomized clinical trial protocol
Source: PLoS One. 2025 May 19;20(5):e0324335. doi: 10.1371/journal.pone.0324335 (PMC12088510; doi:10.1371/journal.pone.0324335)
Supplement: S6 File — (PDF) [file pone.0324335.s006.pdf]

OMAHA+ Assessment Checklist

	Parameters	Measurements
**O**	Oxigenation	1. $PaO^2/FiO^2 \geq 150$<br>2. SaO2 >90% and $FiO^2 \leq 0.4$<br>3. PEEP ≤ 8 cmH2O<br>4. The A-a O^2 Gradient<br>5. Oxygenation index: FiO^2 X MAP X 100/PaO^2<br>6. TOBIN (RR/TV) <105 breaths/min/L
**M**	Mechanical ventilation	1. TV > 5 ml/Kg<br>2. Vital capacity >10 ml/Kg<br>3. Maximal inspiratory pressure ≤ 20-25 cmH2O<br>4. RR ≤ 35 per minute<br>5. Measure maximal inspiratory pressure and maximal expiratory pressure in patients with neuromuscular disorders
**A**	Acid-base balance	1. Arterial blood gases without significant respiratory acidosis: $PaCO^2$ < 50 mmHg, PH > 7.32<br>2. Lactate<br>3. Venous oxygen saturation
**H**	Hemodynamics	1. SBP 90/160 mmHg<br>2. HR ≤ 140 beats per minute<br>3. Vasopressor support?<br>4. Heart arrhythmia?
**A**	Airway	1. Difficult airway?<br>2. Cuff Leak Test?
**+**	Clinical	1. Effective cough?<br>2. Abundant bronchial secretions?<br>3. Resolution of the acute condition requiring mechanical ventilation?<br>4. Mental status: sedated or minimally sedated<br>5. Agitated or anxious<br>6. Diaphoresis, cyanosis, excessive respiratory effort (use of accessory muscles, facial expression of stress, dyspnea)?

A-a O2 Gradient: Alveolar-arterial oxygen gradient; FiO2: Fraction of inspired oxygen; HR: Heart rate; MAP: Mean arterial pressure; PaCO2: Partial pressure of carbon dioxide in arterial blood; PaO2/FiO2: Ratio of arterial oxygen partial pressure (PaO2) to fractional inspired oxygen (FiO2); PEEP: Positive end-expiratory pressure; SaO2: Arterial oxygen saturation; SBP: Systolic blood pressure; TOBIN (RR/TV): Ratio of respiratory rate (RR) to tidal volume (TV), also known as the Rapid Shallow Breathing Index (RSBI); TV: Tidal volume.
