# Supplementary material for: Efficacy and safety of the early implementation of a multimodal rehabilitation program in mechanically ventilated patients: A randomized clinical trial protocol
Source: PLoS One. 2025 May 19;20(5):e0324335. doi: 10.1371/journal.pone.0324335 (PMC12088510; doi:10.1371/journal.pone.0324335)
Supplement: S7 File — (PDF) [file pone.0324335.s007.pdf]

**Case report form**

**Admission Data:**

**Patient Data:**

- Patient's Name: ____________________
- Code: ______________ (1, 2, 3...)
- Medical Record Number: ________________
- Sex: _______________
- Ethnicity: ____________ (Afro-Colombian, ROM, Indigenous, Raizal, None)
- Barthel Index: _____________
- Body Mass Index: __________________
- Diagnosis: ____________ ICD-10 Diagnostic Code: ________________
- Group: ______________ (Early vs. Late)
- Admitting Unit: ____________ (1 = Surgical, 2 = Neurological, 3 = Septic and Respiratory, 4 = Cardiovascular, 5 = Burns)
- SOFA Score: ___________
- APACHE II Score: ____________
- SAPS II Score: _______________
- Admission Date: ________________
- Discharge Date: ________________
- Time from Intubation to Therapy Start: ________________

**Respiratory Parameters:** Variables at the time of intubation, provided by respiratory therapy.

- Tidal Volume: ________________
- Respiratory Rate: ________________
- PEEP (Positive End-Expiratory Pressure): ________________
- Plateau Pressure: ________________
- Static Lung Compliance: ________________
- Driving Pressure: ________________
- Airway Resistance: ________________
- PaO2/FIO2 Ratio: ________________

**During Observation:** To properly record these data, daily patient evaluations are required.

- Mechanical Ventilation Days: _______________________________ (start date - end date)
- Ventilator-Free Days: _________________ (calculated at the end of observation)
- Delirium Days: __________________________________________________ (start date - end date)
- Hospitalization Days with Delirium: _______ (calculated at the end of observation)
- Sedation Days: _____________ (start date - end date)
- Sedation-Free Days: ________________ (calculated at the end of observation)
- Prolonged Mechanical Ventilation: ________________ (Yes/No)

- Tracheostomy: ______ (Yes/No)
- Adult Respiratory Distress Syndrome: _____ (Yes/No)
- Health Care-Associated Pneumonia: ________ (Yes/No)
- Need for Dialysis: ________ (Yes/No)
- Norepinephrine Support: ________ (Yes/No)
- Vasopressin Support: ________ (Yes/No)
- Inotropic Support: ________ (Yes/No)
- Maximum Norepinephrine Dose: _______ (mcg/kg/min)
- Maximum Vasopressin Dose: ______ (U/min)

**Weaning Data:** Weaning start will be determined by the therapist.

- Delta POCC: ____ (cmH2O)
- Muscle Pressure: _____ (cmH2O)
- P0.1 (Pressure in 0.1 seconds): ____ (cmH2O)
- Amount of Secretions via Orotracheal Tube: ____ (Yes/No)
- Asynchrony Index: ___ (%)
- NIF: ____ (cmH2O)
- Leak Percentage: ____ (% leak)
- Cough Peak Flow: ___ (L/min)
- Diaphragmatic Excursion Measurement: ____ (cm)
- Diaphragmatic Thickness Percentage Measurement: ____ (cm)

**Pre-Extubation Data:**

- Delta POCC: ____ (cmH2O)
- Muscle Pressure: _____ (cmH2O)
- P0.1 (Pressure in 0.1 seconds): ____ (cmH2O)
- Amount of Secretions via Orotracheal Tube: ____ (Yes/No)
- Asynchrony Index: ___ (%)
- NIF: ____ (cmH2O)
- Leak Percentage: ____ (% leak)
- Cough Peak Flow: ___ (L/min)
- Diaphragmatic Excursion Measurement: ____ (cm)
- Diaphragmatic Thickness Percentage Measurement: ____ (cm)
- OMAHA at Extubation: _______________ (+/-)

**Post-Extubation Data:**

- Dysphonia at 72 Hours: _______________ (Yes/No)
- Maximum Mobility Measured by JH-HLM at 24 hours: _______________
- Grip Strength at 24 hours: _______________
- Need for Non-Invasive Mechanical Ventilation at 48 hours: __________ (Yes/No)
- Dysphagia at 72 Hours: _______________ (Yes/No)
- Oral Intake Start: _______________ (1: 12-24 hours, 2 > 24 hours)
- Extubation Failure at 48 hours: _______________ (Yes/No)
- Muscle Strength Measured with MRC Scale at 24 hours: _______________

**End of Observation:** Functionality and Stay:

- Barthel Index at Hospital Discharge: _________________
- Cognitive Function Measured by MOCA: ________
- Quick Sensitivity and Dexterity Test: _______
- Hospital Stay Days: __________ (day the intensivist reports transfer to ward)
- ICU Stay Duration: ______________________ (days)
- Mortality: __________________________ (Yes/No)
- Functional Independence Status Measured by ADLs at Hospital Discharge: _______________
- Functional Independence Status Measured by ADLs at ICU Discharge: _______________

**Safety Variables: Outcomes:**

- Number of Adverse Events:
    - Altered Blood Pressure: ________ (Yes/No) (MAP decrease > 15%)
    - Cardiac Arrhythmia: ________ (Yes/No)
    - Oxygen Desaturation: ________ (Yes/No) (decrease below 80%)
    - Pain or Agitation: ________ (Yes/No)
    - Invasive Line Removal: ________ (Yes/No)
    - Gastrointestinal (nausea, vomiting, or diarrhea): ________ (Yes/No)
    - Tachypnea: ________ (Yes/No)
    - Altered Neurological Status: ________ (Yes/No)
- Number of Serious Adverse Events: _______
- Unplanned Extubation: ________ (Yes/No)
- Health Care-Associated Pneumonia: ________ (Yes/No)
- Bronchoaspiration: ________ (Yes/No)
