# Supplementary material for: Efficacy and safety of the early implementation of a multimodal rehabilitation program in mechanically ventilated patients: A randomized clinical trial protocol
Source: PLoS One. 2025 May 19;20(5):e0324335. doi: 10.1371/journal.pone.0324335 (PMC12088510; doi:10.1371/journal.pone.0324335)
Supplement: Attachment — Submitted filename: PONE-D-24-31135_R1_DE_07112024.pdf [file pone.0324335.s009.pdf]

PLOS ONE

Efficacy and Safety of the Early Implementation of a Multimodal Rehabilitation Program in Mechanically Ventilated Patients: A Randomized Clinical Trial Protocol

--Manuscript Draft--

Manuscript Number:	PONE-D-24-31135R1
Article Type:	Registered Report Protocol
Full Title:	Efficacy and Safety of the Early Implementation of a Multimodal Rehabilitation Program in Mechanically Ventilated Patients: A Randomized Clinical Trial Protocol
Short Title:	A Multimodal Rehabilitation Program for Mechanically Ventilated Patients: An RCT Protocol
Corresponding Author:	Jorge Iván Alvarado Sánchez<br>Fundacion Santa Fe de Bogota<br>Bogotá, COLOMBIA
Keywords:	Intensive care unit-acquired weakness , post-intensive care syndrome, Early physical therapy, Critical Care
Abstract:	Background<br>Intensive care unit-acquired weakness (ICUAW) and post-intensive care syndrome (PICS) are significant complications among critically ill patients, leading to prolonged hospital stays, increased healthcare costs, and reduced quality of life. Early physical therapy in the ICU has shown promise in mitigating these adverse outcomes, yet randomized controlled trials (RCTs) evaluating the combined effects of physical, respiratory, occupational, and speech therapy initiated within the first 24 to 72 hours are lacking.<br>Methods<br>This single center, controlled clinical trial aims to establish the efficacy and safety of an early multimodal rehabilitation program (MRP) compared with a late MRP in mechanically ventilated patients admitted to the ICU. Adult patients (≥18 years) with a Barthel score ≥70 and requiring invasive mechanical ventilation for more than 24 hours will be included. Participants will be randomly assigned to early MRP, initiated within 24 hours post-intubation, or late MRP, starting 72 hours post-intubation. The primary outcome is the duration of mechanical ventilation. Safety will be assessed by comparing the number of adverse events between groups.<br>The MRP includes passive and active interventions from physical, speech, respiratory, and occupational therapy teams. Therapy intensity and type will be adjusted according to the patient's Richmond Agitation-Sedation Scale (RASS) score. Patients with lower RASS scores (indicating deeper sedation) will receive primarily passive interventions (e.g., passive range-of-motion exercises), while those with higher RASS scores (indicating lighter sedation or alertness) will engage into more active therapies (e.g., active-assisted exercises and functional mobility activities). This adaptive approach ensures patient safety and optimizes therapy based on the patient's current sedation level and clinical condition.<br>Dissemination<br>The results will be published in peer-reviewed journals and presented at conferences.<br>Trial registration<br>ClinicalTrials.gov, NCT06133504. Registered on November 11, 2023.
Order of Authors:	Jorge Iván Alvarado Sánchez
	Laura Maria Castillo Morales
	Yenny Rocio Cardenas Bolivar
	Andrea Valentina Montañez-Nariño
	Maria Valentina Stozitzky-Ríos
	Catherine Lissell Arévalo Guerrero
	Miguel Leonardo Pulido Bobadilla
	Diana Marcela Melo Rojas

Powered by Editorial Manager® and ProduXion Manager® from Aries Systems Corporation

	Ana Gabriela López Rubio
	Diana Carolina Ortíz Moreno
	Paula Andrea Barreto Garzón
	Marisol Murrillo
	Andrés Felipe Mora-Salamanca

Response to Reviewers:

Letter to reviewers

Dear Editor,

Thank you for allowing us to send you the revised version of our study protocol. Below, you will find the responses to each of your requirements.

Journal Requirements:
When submitting your revision, we need you to address these additional requirements.
1.Please ensure that your manuscript meets PLOS ONE's style requirements, including those for file naming. The PLOS ONE style templates can be found at https://journals.plos.org/plosone/s/file?id=wjVg/PLOSOne_formatting_sample_main_body.pdf and
https://journals.plos.org/plosone/s/file?id=ba62/PLOSOne_formatting_sample_title_authors_affiliations.pdf

We have reviewed the manuscript and supplemental material and adjusted to the PLOS ONE’s style requirements.

2.Please provide a complete Data Availability Statement in the submission form, ensuring you include all necessary access information or a reason for why you are unable to make your data freely accessible. If your research concerns only data provided within your submission, please write "All data are in the manuscript and/or supporting information files" as your Data Availability Statement.

Thank you for your valuable feedback. In response to your request regarding the Data Availability Statement, we have updated it as follows:

•Data Availability Statement: All data generated or analyzed during this study will be available upon study completion and reasonable request. If the data cannot be made publicly accessible, this will be clearly stated along with the reasons. Currently, all relevant data are included in the manuscript or supporting information files.

We hope this revision meets the requirements, and we remain available for any further clarification.

3.Your ethics statement should only appear in the Methods section of your manuscript. If your ethics statement is written in any section besides the Methods, please delete it from any other section.

Thank you for your feedback. We have made the necessary adjustments, and the ethics statement now appears only in the Methods section of the manuscript, as requested.

Reviewers' comments:

Reviewer's Responses to Questions
Comments to the Author

Powered by Editorial Manager® and ProduXion Manager® from Aries Systems Corporation

Reviewer #1: There are some major statistical problems in this protocol:
1.A survival analysis is given as the data analysis technique for the primary outcome, yet the sample size is based on a t-test. The test of equality of survival curves must use sample size formulas based on survival data (e.g., Rosenberger and Lachin, Randomization in Clinical Trials, Wiley, 2016, Section 2.6.3).

Thank you for your insightful feedback. We would like to clarify that our primary outcome, the duration of invasive mechanical ventilation, was conceptualized and analyzed as a quantitative variable in units of time (days), not as a time-to-event variable typically used in survival analysis. For this reason, we did not consider the survival analysis into the sample size estimation. We used a difference in means approach, which we deemed more appropriate for this outcome.

In the revised manuscript, we have clarified this distinction to ensure that the variable is correctly understood as quantitative (days), and not a time-to-event outcome (lines 366-370). Although our outcome could be perceived as time-dependent, we felt that analyzing it as a difference in means better aligned with the nature of the data and our research objectives.

We acknowledge your suggestion regarding the use of survival data formulas and will carefully consider this point in future analyses or similar studies

2.The randomization procedure ("computer generated") is not given, nor a discussion of potential stratification, blocking, etc. (e.g., Rosenberger and Lachin, Randomization in Clinical Trials, Wiley, 2016, Chapter 3).

Thank you for your valuable feedback. In response to your suggestion regarding the randomization procedure, in the protocol we included a stratified randomization strategy. Specifically, participants will be stratified by age (<65 and ≥65 years), sex (male and female) and comorbidities (Charlson comorbidity index <8 and ≥8) resulting in eight strata. We added his information in the manuscript (lines 493-496).

3.Sealed envelopes were used in the 1948 streptomycin trial, but these days we use computer generated assignments available through Redcap or on a Shiny app.

Thank you for your valuable suggestion regarding the randomization procedure. We changed the sealed envelopes to a random allocation sequence provided by REDCap (lines 493-503).

4. The data analysts obviously cannot be blinded to the data! How can they do comparisons and test the primary outcome if they are blinded? What needs to be blinded is the assessment of the primary outcome, which should be done independently of those providing the intervention.

Thank you for your valuable observation. We agree that it is not feasible to blind the data analysts to the data itself. To address your concern, we have clarified that the assessment of the primary outcome will be performed by an independent evaluator, who will be blinded to the group assignments (lines 507-512 and 568-569). This ensures that the outcome assessment is done objectively and independently from those providing the intervention. The data analysts will have access to the full dataset for the statistical analysis.

5. Very complicated set of interventions, but if successful, the results will be interesting.

Thank you for your thoughtful feedback. We appreciate your recognition of the complexity and potential impact of our study.

Reviewer #2: Manuscript PONE-D-24-31135

Title: Efficacy and Safety of the Early Implementation of a Multimodal Rehabilitation Program in Mechanically Ventilated Patients: A Randomized Clinical Trial Protocol

Thank you to the authors for a well-articulated and referenced manuscript. The study protocol requires additional methodological detail and consideration of the most appropriate outcome measure for this study. The manuscript will be publishable with moderate adjustments, particularly to the Introduction and Methods, in this reviewer's view.

The fundamental question for the authors in the study design is to consider whether starting the input two days (48hrs) apart is enough to create a measurable study effect, and whether the sample groups are large enough to observe this effect. For instance, tailoring interventions to the RASS score could result in participants from both groups receiving similar and, or low volumes of therapy because heavy sedation or agitation excludes them for two days of their stay from receiving 'active' treatments.

Please adjust the manuscript and or respond to the comments below.

Abstract:

1. Please make it clearer for the reader as to how will the RASS score affect therapy input.

Thank you for your helpful feedback. We have revised the abstract to clarify how the RASS score influences therapy input (lines 55-62). Specifically, therapy intensity and type are now explicitly adjusted according to the patient's RASS score, with passive interventions for those with lower scores (indicating deeper sedation) and more active therapies for patients with higher scores (indicating lighter sedation or alertness). This adaptive approach ensures that the therapy is tailored to each patient's clinical condition (Table 1 and lines 224-236).

Introduction:

2.The study justification as articulated in the Introduction needs rework. The primary outcome measure is not post-ICU outcomes as purported as the main reason for early intervention, it is time on the ventilator. Why would the investigators not use a primary measure that is a post-ICU physical or quality of life outcome, as justified in by the Introduction?

Thank you for your valuable feedback. In response to your comment, we have revised the Introduction to clarify the justification for our study. Specifically, we have emphasized that the duration of mechanical ventilation is a key driver of adverse outcomes, such as those seen in post-intensive care syndrome (PICS) and is therefore an important target for early intervention. We have also highlighted how the primary outcome (time on the ventilator) aligns with the study's aim of reducing long-term complications by addressing this critical factor (lines 76-8[illegible]

Methods:

3.Eligibility Criteria – requires additional detail and justification.

Thank you for your feedback regarding the eligibility criteria. We have revised this section to provide additional detail and justification for each inclusion and exclusion criterion (lines 183-190). Specifically, we now clarify that:

•A Barthel score ≥70 ensures participants have a reasonable level of functional independence pre-ICU, allowing for accurate assessment of the rehabilitation intervention's impact.

•Invasive mechanical ventilation >24 hours ensures that patients have a sufficient duration of mechanical support to benefit from early rehabilitation.

4.Will patients with ICU length of stay shorter than 72 hours be included? How will this be accounted for in the study and statistical analyses? How might this impact the efficacy of group allocation (randomisation)?

Powered by Editorial Manager® and ProduXion Manager® from Aries Systems Corporation

Patients with ICU stays shorter than 72 hours will be included in the study. However, we recognize that shorter stays might impact their exposure to the full intervention. To address this:
•Primary Analysis: We will conduct an intention-to-treat analysis, where all randomized patients are analyzed in the groups they were assigned to, regardless of the length of their ICU stay or the level of intervention received.

•Secondary Analysis: We will perform a sensitivity analysis excluding patients with stays shorter than 72 hours to assess how this impacts the results. Additionally, a subgroup analysis will compare outcomes between patients with longer and shorter ICU stays.
•Statistical Adjustments: The duration of ICU stay will be included as a covariate in the statistical models to control for its impact on the primary outcome.
•Randomization Impact: Randomization will occur upon ICU admission, ensuring that the group assignment is not biased by predicted ICU stay. We will assess balance between groups post-randomization and implement adjustments if necessary.
(See statistical analysis section).

5. Line 181 - Will patients with isolated smoke inhalation injury (airway burn) be excluded? If so, why?

Thank you for your question. Yes, patients with isolated smoke inhalation injury (airway burn) will be excluded from the study (line 198). It is well known that these patients often require prolonged mechanical ventilation due to the nature of their injury, which could introduce significant variability into our study outcomes. Given that the primary outcome is the duration of mechanical ventilation, including these patients could skew the results and make it difficult to assess the effects of the intervention on the general ICU population (https://doi.org/10.1042/CS20040135

6.Line 183 - Why are patients with COPD excluded?

Thank you for your question. Patients with chronic obstructive pulmonary disease (COPD) are excluded from the study because they tend to require prolonged mechanical ventilation due to their underlying respiratory dysfunction. Including COPD patients could introduce variability in the primary outcome (duration of mechanical ventilation), making it more challenging to isolate the effects of the intervention on the general ICU population.
According to Sellares et al. (2011), COPD was identified as a significant predictor of prolonged weaning from mechanical ventilation, with an odds ratio of 2.97 (95% CI: 1.29–6.82). This finding further supports the exclusion of COPD patients to ensure that the study results are not biased by this group's typically prolonged ventilation requirements.
Reference:
1.Sellares J, Ferrer M, Cano E, et al. Predictors of prolonged weaning and survival during ventilator weaning in a respiratory ICU. Intensive Care Medicine. 2011;37(5):775-784.

7.Line 187 - How will life expectancy criteria be applied at recruitment?

Thank you for your question. The life expectancy criterion (≤180 days) will be applied at recruitment based on a combination of factors, including:
1.Primary diagnosis and comorbidities, such as advanced cancer, end-stage heart failure, or other critical irreversible conditions.
2.Medical judgment of the ICU team, considering the patient's recent clinical course and overall prognosis.
3.Functional status, as measured by the Barthel index, which is already included in our study to assess the patient's ability to perform activities of daily living.
This multi-faceted approach will ensure that only patients who are likely to benefit from the intervention are included in the study, while avoiding potential bias introduced by

Powered by Editorial Manager® and ProduXion Manager® from Aries Systems Corporation

including patients with very limited life expectancy (see exclusion criteria).

8.Line 204 - Currently the daily number of treatments is not standardised. This reviewer strongly suggests that the number of intervention events be standardised to one or two per day per discipline to reduce study variability.

9.Similarly, the volume of treatment (minutes of input) applied per participant is a significant variable in this study. How will the investigators account for the daily differences?

10.Line 212 - Is there a better way to schedule the interventions to reduce variation in active treatment volume per patient? For instance, rather than accept that therapy is not completed or sessions markedly shortened due to periods of high sedation and agitation, could the investigators apply a RASS driven protocol which optimises the daily input (minimises down time) for the patient through the standardising treatment according to the strengths of different disciplines of the team?

Thank you for your insightful feedback. In response to your comments, we have standardized the interventions while maintaining different volumes for each therapy (Table 1). Speech therapy will consist of two sessions of 10 minutes per day, while both physical therapy and respiratory therapy will involve two sessions of 30 minutes per day. Occupational therapy will be provided once daily for 30 minutes. We have also aligned the interventions with a RASS-driven protocol to optimize treatment and minimize downtime.
•For patients with a RASS ≤ -2, passive and passive-assisted interventions will focus on maintaining joint mobility, sensory input, and pulmonary function.
•For patients with a RASS between -1 and +1, more active rehabilitation will be provided, including motor activation, respiratory exercises, and cognitive tasks.
•For patients with a RASS ≥ 2, physical, speech, and respiratory therapies will continue as prescribed for the -1 to +1 group. However, occupational therapy will shift focus towards behavioral and environmental modulation to reduce agitation, incorporating sensory stimuli and family education to promote spatiotemporal orientatio[illegible]

11.Line 299 – How will adverse events be managed in the study analyses?

Adverse events will be systematically recorded and categorized according to their severity and their relationship to the intervention. Serious adverse events include altered blood pressure, cardiac arrhythmia, oxygen desaturation, agitation requiring sedation, removal of invasive lines, altered neurological status, accidental extubation, healthcare-associated pneumonia, and bronchoaspiration. These events will be carefully monitored throughout the stu[illegible]
Adverse events will be analyzed desc[illegible]ely, with the frequency and type of events reported for each study group. In addition, an intention-to-treat (ITT) analysis will be applied for the primary outcome, ensuring that participants who are withdrawn due to serious adverse events remain in the analysis according to their original group assignment. Secondary safety analyses will be conducted to compare the incidence of adverse events between the groups and to assess the overall safety profile of the interventions (see Patient withdrawal and statistical analysis sections)

12.Line 308 – Will the patient withdrawn due to complications no longer receive treatment in ICU? Is there any specific justification for why those with complications are withdrawn? Complications are relatively common in ICU and strictly applying this criteria may impact the sample size, feasibility of the study and ability to measure secondary outcomes with integrity.

Thank you for your thoughtful questions. Patients withdrawn due to complications will continue to receive full treatment in the ICU as part of the standard care. The decision to withdraw them from the study is solely for research purposes and is based on the need to ensure their safety in relation to the study interventions. These patients will continue to be monitored and treated for their clinical needs, but they will no longer

Powered by Editorial Manager® and ProduXion Manager® from Aries Systems Corporation

participate in the specific study interventions if a serious adverse event occurs. They will continue receiving any or all therapies according to the judgement of the critical medicine and intensive care team (physicians, therapists and nursing staff), but the frequency and intensity may vary compared to the multimodal rehabilitation program proposed in our study (additional reason to exclude them from the study).

The justification for withdrawing patients with complications is to prevent any potential harm or exacerbation of their condition as a result of the study interventions. We acknowledge that complications are relatively common in the ICU; however, we aim to balance patient safety with the integrity of the study.

Importantly, patients who are withdrawn will still be included in the analysis based on an intention-to-treat approach. This ensures that the sample size is preserved, and the feasibility and ability to measure secondary outcomes are maintained without compromising the safety of participants.

We have also planned to conduct subgroup analyses to explore the impact of complications on the study outcomes, which will provide further insights without compromising the integrity of the data. (see Patient withdrawal and statistical analysis sections)

13.Line 314 – This criterion is troublesome as the treatment is not standardised. How will non-compliance be confirmed and at what ‘amount’ of deviation from the protocol will constitute need for withdrawal? More detail needed please.

Thank you for your insightful feedback. We have removed that withdrawal criterion. Instead, we have added information about how patients will be monitored regarding their adherence to the intervention (lines 313-340). Additionally, we will make a subgroup analysis of adherent compared to non-adherent patients. It is important to mention that non-compliance and protocol deviations are serious research violations, and those terms do not apply to participant’s adherence to the intervention (https://research.umbc.edu/protocol-deviationsor-issues-of-non-compliance/). We follow current Good Clinical Practice guidelines, and we adhere to the Helsinki Declaration and the Colombian Legislation.

14.Line 328 – How will transfers to another facility be treated? Will their data prior to transfer be included in the study?

Thank you for your question. Data from patients transferred to another facility will be retained and if appropriate, it will be included in the intention to treat analysis. If the proportion of missing data due to the transfer is less than 20%, we will use data imputation methods to account for the missing values. This ensures that the contribution of these patients is not lost and that the sample size and statistical power are preserved. Transfers will be recorded as a separate variable, and we will explore their potential impact on the study outcomes through sensitivity analyses (line 293-301).

Outcomes:

15.Line 356 – how often will ventilator parameters be collected and how will they be included in the Statistical Analyses?
Thank you for your question. Ventilator parameters will be collected at three times. First, these parameters will be collected after randomization, ensuring that all patients start under comparable conditions. Second, they will be collected when the critical medicine and intensive care group decide that the patient can start the mechanical ventilation weaning (lines 432-439). Finally, the ventilation parameters will be recorded during the OMAHA assessment (just before extubating).
For the statistical analysis, we will perform comparisons using mean or median differences, depending on the distribution of the data. This will help us confirm that

Powered by Editorial Manager® and ProduXion Manager® from Aries Systems Corporation

there are no significant differences in ventilator settings between the groups at the start of the study, at the beginning of the mechanical ventilation weaning and before extubating (see outcomes section and figure [illegible]

16.The primary outcome is not justified by the Introduction as it focusses on post-ICU acquired weakness, PICS, physical and quality of life outcomes. It would seem more likely that the primary outcome has been chosen for convenience and not related to the substance of the study and planned interventions?

Thank you for your insightful feedback. We have revised the Introduction to better align it with the primary outcome of duration of mechanical ventilation. The updated Introduction now highlights the importance of early rehabilitation interventions in improving respiratory function and reducing the duration of mechanical ventilation. This outcome was chosen not only for its direct clinical relevance but also because reducing mechanical ventilation duration can contribute to better long-term outcomes, such as a reduction in post-ICU acquired weakness and PIC[illegible]

By emphasizing the link between the interventions and the immediate benefits on respiratory recovery, we believe the primary outcome is well-justified in the context of the study.

17.Line 371 – cardiac arrythmias are mentioned as a reason for withdrawal earlier. How will adverse events be delineated as an occurrence to be recorded, or result in participant withdrawal which will affect the sample size and power of the trial analyses?

Thank you for your question. Adverse events, such as cardiac arrhythmias leading to hemodynamic instability, will be recorded and monitored. It is important to highlight that to be withdraw due to a cardiac arrhythmia; this complication must lead to hemodynamic instability. Patients experiencing serious adverse events may be withdrawn for safety reasons. However, they will continue to receive full ICU care.

Data up to the point of withdrawal will be included in the intention-to-treat analysis, ensuring minimal impact on sample size and study power. We will also conduct sensitivity analyses to assess any potential impact of these withdrawals on the study outcomes. Additionally, we added 12 patients (20%) to the sample size. Adding these patients minimizes the risk of losing statistical power due to participants' withdrawal

18.Line 384 – The criteria 28 days after intubation or hospital discharge is ambiguous. Please clarify.

Thank you for your question. To clarify, all outcomes will be measured up to day 28 after intubation in case the patient remains hospitalized or up to hospital discharge, in case the hospitalization period lasted less than 28 days. We included this endpoint in order to maximize the comparability among patients who were discharge early (< 28 days after intubation) and those who remained in the hospital (≥ 28 days). There may be some patients whose hospitalization period could last several weeks or even months, and compare all patients at the moment of discharge could distort the results at T4 (lines 490-493).

Participant Timeline
19. Line 414 – is there a typo – 24-72 hours post-extubation?
Sample Size. 20.

Thank you for your observation. To clarify, we are indeed referring to 24-72 hours post-intubation, during which we will measure functional outcomes, incidence of dysphagia, dysphonia, delirium, ARDS, adverse events, and mortality. These time points are critical for assessing early outcomes related to the interventions. Please refer to Figures 1 and 2 for more details on the timeline and specific outcomes measured during this period.
We appreciate your attention to this detail and will ensure it is clearly stated in the revised manuscript.

Powered by Editorial Manager® and ProduXion Manager® from Aries Systems Corporation

20.Line 438 – 440 – is there a typo related to the group description for SD1 and SD2?

Thank you for your observation. After reviewing the text, we realized there is indeed a typo in the description of SD1 and SD2. SD1 should refer to the standard deviation in the group receiving early physical therapy, while SD2 should refer to the standard deviation in the group receiving late physical therapy. We will correct this in the revised manuscript to ensure clarity (lines 460-464)
We appreciate your careful attention to detail.

21.The primary outcome is measured in hours, however the sample size has been calculated using days. Does this make a difference to the mathematics of the calculation?

Thank you for your observation. We have already corrected this issue, and the primary outcome is now consistently measured and calculated in days throughout the study, including the sample size calculation. This ensures consistency in the methodology and avoids any discrepancies in the analysis.

22.The sample size seems particularly low for an ICU study. There are a multitude of variables which could affect the time ventilated (including the patient diagnosis or reason for critical care), the standardisation of the interventions and cause withdrawal of a patient according to the proposed criteria. Has the mathematics of this sample size calculation been applied to account for so many variables which may not be managed effectively by stratification of random allocation? Please revisit.

Thank you for your thoughtful comment. We recognize this limitation, especially when considering the impact of the intervention on other outcomes such as:
•Number of ventilator-free days (up to 28 days).
•Number of days with delirium (up to 28 days).
•Number of days with delirium in the ICU (up to 28 days).
•Number of days with delirium in the general ward, among others.
However, we plan to conduct a post-hoc power analysis upon reaching the target sample size, particularly for outcomes where statistical differences are not observed. This approach has been outlined in the statistical analysis section of the protocol to ensure that any potential limitations in statistical power are accounted for in our final assessment (lines 646-652).

Recruitment and Allocation
23.Stratification for age has been applied. Why not also for diagnosis?
Data collection and management

Thank you for your question. We have stratified for age because it is a well-established factor that significantly impacts outcomes in the ICU. However, stratifying for diagnosis was not included due to the wide variety of diagnoses and the complexity it would add to the stratification process. Instead, we plan to control for diagnosis through multivariable regression analyses in the statistical analysis phase. This will allow us to account for the potential influence of different diagnoses without complicating the randomization process.

24.is unclear how long a patient needs to remain in the study before their case is considered as part of the sample. For instance, with short ICU length of stay, will there be adequate time for the interventions to be applied and impact the outcome?

Thank you for your important question. We recognize that ICU length of stay may vary significantly among patients. To address this:

1.All patients will be included in the study and analyzed under the intention-to-treat principle, regardless of their ICU length of stay. This ensures that every patient's data contributes to the overall analysis.
2.For patients with short ICU stays, we acknowledge that the time may be insufficient for the intervention to have its full intended effect. Therefore, we will conduct a sensitivity analysis to explore how these patients impact the outcomes. This will allow

us to assess whether their inclusion affects the interpretation of the study results.

We hope this clarifies our approach to managing patients with varying ICU lengths of stay (see statistical analysis).

25.Line 501 – Data verification – this statement reads like a statistical 'get out of jail free' card. Why would extreme values be assessed for verification if not to exclude them from the study analysis? Please clarify the intent of this action in the study.

Thank you for your question. The verification of extreme values is not intended to exclude them from the analysis arbitrarily. Instead, we will assess extreme values through regression models that allow us to identify leverage points and evaluate whether removing them would significantly alter the model. If the extreme value does not modify the model, it will be kept in the analysis, as it may help explain important aspects of the data.

Additionally, we will verify all extreme values to ensure that any extreme values reflect true clinical situations and do not represent data entry errors. By using this rigorous approach, we ensure that extreme values are handled appropriately without compromising the integrity of the study.

26.Line 508 - ? typo (tense) – please check expression.

Thank you for pointing out the typo. The correct expression should be:
"If a patient provides their informed consent and wishes to withdraw from the study, we will inquire about their reasons for discontinuing participation."
We corrected this in the revised manuscript (lines 540-541).

Statistical Analysis
27.Exactly which variables will be carried forward using the intention-to-treat principles?
Thank you for your question. In the intention-to-treat (ITT) analysis, we will carry forward the variables that are key to the outcomes observed over the 90-day period, specifically:
•Primary outcome: Duration of mechanical ventilation.
•Secondary outcomes, including:
oNumber of ventilator-free days (up to 28 days).
oIncidence of delirium, sedation days, and adverse events such as dysphagia, dysphonia, ARDS, and mortality (assessed up to 90 days).
Only these outcomes will be included in the ITT analysis, ensuring the most relevant data are included despite any early withdrawal (see figure 1 and 2).

28.Line 544 - Please explain the risk variable. If the patient is not extubated, how can the time of mechanical ventilation be used in the proposed variable? How is this model interpreted? Also, as time on the ventilator is the primary variable, how can it be included as a co-variate in the same statistical model?

Thank you for your valuable feedback. We agree that time on mechanical ventilation is our primary outcome and will not be included as a covariable in the statistical model. For patients who are not extubated by the end of the follow-up period, we will censor their time on ventilation at the last point of observation (up to 28 days). This means that their data will be included up to the moment of censoring, reflecting the maximum duration of ventilation recorded during the study period.
The variable will be interpreted as the time until extubation for those successfully extubated, and for those censored, it will represent the maximum duration of ventilation observed without extubation. This approach ensures that all patients are included in the analysis while maintaining the integrity of the data.
We appreciate your input and will reflect these adjustments in the revised manuscript.

	29.The statistical modelling is complex and involves not only known variables but also interaction terms and ad hoc tests. Is the relatively small sample size with repeated measures longitudinal design likely to support an interpretable analysis for any of the secondary outcomes?<br><br>Thank you for your observation. We acknowledge the complexity of the statistical modeling, especially given the relatively small sample size. To address this concern, we have already planned to conduct a post-hoc power analysis to assess whether the sample size is sufficient to detect differences in both primary and secondary outcomes. Additionally, if certain analyses prove difficult to interpret due to the sample size or complexity, we will adjust our models accordingly, potentially simplifying the analysis or conducting exploratory analyses for the secondary outcomes. These considerations are outlined in the statistical analysis section.<br><br>Figures<br>30. Figure 1 – The interventions subsection doesn't add value or needs adjustment to demonstrate that the group allocation (time and volume of interventions) is the key variable in the study.<br><br>Thank you for your valuable feedback. We have adjusted Figure 1 to better emphasize that the key variable in the study is the group allocation, specifically in terms of timing and volume of interventions. The figure now clearly shows the differences in the timing of interventions (Early MRP within 24 hours and Late MRP after 72 hours) as well as the standardized frequency and duration of interventions for each group. A clarification has been added in the figure's footnote to reflect these adjustments.
**Additional Information:**	
**Question**	**Response**
**Financial Disclosure**<br><br>Enter a financial disclosure statement that describes the sources of funding for the work included in this submission. Review the submission guidelines for detailed requirements. View published research articles from *PLOS ONE* for specific examples.<br><br>This statement is required for submission and **will appear in the published article** if the submission is accepted. Please make sure it is accurate.	The author(s) received no specific funding for this work.

**Funded studies**<br>Enter a statement with the following details:<br>• Initials of the authors who received each award<br>• Grant numbers awarded to each author<br>• The full name of each funder<br>• URL of each funder website<br>• Did the sponsors or funders play any role in the study design, data collection and analysis, decision to publish, or preparation of the manuscript?<br><br>Did you receive funding for this work?	
**Competing Interests**<br><br>Use the instructions below to enter a competing interest statement for this submission. On behalf of all authors, disclose any competing interests that could be perceived to bias this work—acknowledging all financial support and any other relevant financial or non-financial competing interests.<br><br>This statement is required for submission and **will appear in the published article** if the submission is accepted. Please make sure it is accurate and that any funding sources listed in your Funding Information later in the submission form are also declared in your Financial Disclosure statement.<br><br>View published research articles from *PLOS ONE* for specific examples.	The authors have declared that no competing interests exist.

Powered by Editorial Manager® and ProduXion Manager® from Aries Systems Corporation

**NO authors have competing interests**<br><br>Enter: *The authors have declared that no competing interests exist.*<br><br>**Authors with competing interests**<br><br>Enter competing interest details beginning with this statement:<br><br>*I have read the journal's policy and the authors of this manuscript have the following competing interests: [insert competing interests here]*<br><br>* typeset	
**Ethics Statement**<br><br>Enter an ethics statement for this submission. This statement is required if the study involved:<br><br>• Human participants<br>• Human specimens or tissue<br>• Vertebrate animals or cephalopods<br>• Vertebrate embryos or tissues<br>• Field research<br><br>Write "N/A" if the submission does not require an ethics statement.<br><br>General guidance is provided below. Consult the submission guidelines for detailed instructions. **Make sure that all information entered here is included in the Methods section of the manuscript.**	The study was approved by the Ethics Committee at Fundación Santa Fe de Bogotá (CCEI-16320-2024).

Powered by Editorial Manager® and ProduXion Manager® from Aries Systems Corporation

**Format for specific study types**<br><br>**Human Subject Research (involving human participants and/or tissue)**<br>• Give the name of the institutional review board or ethics committee that approved the study<br>• Include the approval number and/or a statement indicating approval of this research<br>• Indicate the form of consent obtained (written/oral) or the reason that consent was not obtained (e.g. the data were analyzed anonymously)<br><br>**Animal Research (involving vertebrate animals, embryos or tissues)**<br>• Provide the name of the Institutional Animal Care and Use Committee (IACUC) or other relevant ethics board that reviewed the study protocol, and indicate whether they approved this research or granted a formal waiver of ethical approval<br>• Include an approval number if one was obtained<br>• If the study involved *non-human primates*, add *additional details* about animal welfare and steps taken to ameliorate suffering<br>• If anesthesia, euthanasia, or any kind of animal sacrifice is part of the study, include briefly which substances and/or methods were applied<br><br>**Field Research**<br><br>Include the following details if this study involves the collection of plant, animal, or other materials from a natural setting:<br>• Field permit number<br>• Name of the institution or relevant body that granted permission	
**Data Availability**<br><br>Authors are required to make the data underlying their research findings fully available, without restriction.<br><br>If data need to be collected, modified or processed specifically for your study, or if participants need to be recruited specifically for your study, then it should	Yes - any pilot data reported in this submission are fully available and data collected during the study will be made fully available without restriction upon study completion

occur only after your Registered Report Protocol is accepted for publication.<br><br>If the manuscript reports pilot results, the data underlying those results should be made available at the time of publication. Submission of this Registered Report Protocol implies a commitment that the data collected in the study, upon completion, will be made available with the Registered Report publication.<br><br>When legal or ethical requirements prohibit public data sharing, authors must indicate how researchers may obtain access to the data. See the PLOS Data Policy and FAQ for detailed information.<br><br>A Data Availability Statement describing where the data can be found is required at submission. Your answers to this question constitute the Data Availability Statement and **will be published in the article**, if accepted.<br><br>**Important:** Stating 'data available on request from the author' is not sufficient. If your data are only available upon request, select 'No' for the first question and explain your situation in the text box.<br><br>Do the authors confirm that all data underlying the findings of the study outlined in this Registered Report Protocol will be made fully available without restriction?	
**Describe where the data may be found in full sentences. If you are copying our sample text, replace any instances of XXX with the appropriate details.**<br><br>• If **no pilot data are reported in the manuscript**, please enter the following: *All relevant data from this study will be made available upon study completion.*	No datasets were generated or analysed during the current study. All relevant data from this study will be made available upon study completion

Powered by Editorial Manager® and ProduXion Manager® from Aries Systems Corporation

• If the manuscript reports pilot data **held in a public repository**, include URLs, accession numbers or DOIs. If this information will only be available after acceptance, indicate this by ticking the box below. For example: *All XXX files are available from the XXX database (accession number(s) XXX, XXX.).*<br>• If the manuscript reports pilot data contained **within the manuscript and/or Supporting Information files**, enter the following:<br>*All relevant data are within the manuscript and its Supporting Information files.*<br>• If neither of these applies but you are able to provide **details of access elsewhere**, with or without limitations, please do so. For example:<br><br>*Data cannot be shared publicly because of [XXX]. Data are available from the XXX Institutional Data Access / Ethics Committee (contact via XXX) for researchers who meet the criteria for access to confidential data.*<br><br>*The data underlying the results presented in the study are available from (include the name of the third party and contact information or URL).*<br>• This text is appropriate if the data are owned by a third party and authors do not have permission to share the data.<br><br>* typeset	
Additional data availability information:	

Manuscript Click here to access/download;Manuscript;PLOS ONE 2.0.docx

Efficacy and safety of the early implementation of a multimodal rehabilitation program in mechanically ventilated patients: A randomized clinical trial protocol

Laura Maria Castillo Morales[1¶], Jorge Iván Alvarado-Sánchez[1,2¶*], Yenny Rocío Cárdenas-Bolivar[1], Valentina Montañez-Nariño[1], María Valentina Stozitzky-Ríos[1], Catherine Lissell Arévalo Guerrero[3], Miguel Leonardo Pulido Bobadilla[3], Diana Marcela Melo Rojas[3], Ana Gabriela López Rubio[4], Diana Carolina Ortiz Moreno[4], Paula Andrea Barreto Garzón[3], Marisol Murillo[3], Andrés Felipe Mora-Salamanca[1]

[1] Department of Critical Medicine and Intensive Care, Fundación Santa Fe de Bogotá, Bogotá, Colombia.

[2] Department of Physiology, Faculty of Medicine, Universidad Nacional de Colombia, Bogotá, Colombia.

[3] Department of Physical Medicine & Rehabilitation, Fundación Santa Fe de Bogotá, Bogotá, Colombia.

[4] Department of Pulmonary Diseases, Fundación Santa Fe de Bogotá, Bogotá, Colombia.

* Corresponding author:

Email: jialvarados@unal.edu.co (JIAS)

¶ These authors contributed equally to this work

Abstract

Background

Intensive care unit-acquired weakness (ICUAW) and post-intensive care syndrome (PICS) are significant complications among critically ill patients, leading to prolonged hospital stays, increased healthcare costs, and reduced quality of life. Early physical therapy in the ICU has shown promise in mitigating these adverse outcomes, yet randomized controlled trials (RCTs) evaluating the combined effects of physical, respiratory, occupational, and speech therapy initiated within the first 24 to 72 hours are lacking.

Methods

This single center, controlled clinical trial aims to establish the efficacy and safety of an early multimodal rehabilitation program (MRP) compared with a late MRP in mechanically ventilated patients admitted to the ICU. Adult patients (≥18 years) with a Barthel score ≥70 and requiring invasive mechanical ventilation for more than 24 hours will be included. Participants will be randomly assigned to early MRP, initiated within 24 hours post-intubation, or late MRP, starting 72 hours post-intubation. The primary outcome is the duration of mechanical ventilation. Safety will be assessed by comparing the number of adverse events between groups.

The MRP includes passive and active interventions from physical, speech, respiratory, and occupational therapy teams. Therapy intensity and type will be adjusted according to the patient's Richmond Agitation-Sedation Scale (RASS) score. Patients with lower RASS scores (indicating deeper sedation) will receive primarily passive interventions (e.g., passive range-of-motion exercises), while those with higher RASS scores (indicating lighter sedation or alertness) will engage into more active therapies (e.g., active-assisted exercises and functional mobility activities). This adaptive approach ensures patient safety and optimizes therapy based on the patient's current sedation level and clinical condition.

Dissemination

The results will be published in peer-reviewed journals and presented at conferences.

Trial registration

ClinicalTrials.gov, NCT06133504. Registered on November 11, 2023.

Introduction

Post-intensive care syndrome (PICS) is a significant complication that affects a large proportion of critically ill patients. PICS encompasses a range of physical, cognitive, and mental health impairments that persist long after discharge from the intensive care unit (ICU) [1]. These complications contribute to prolonged hospital stays, increased healthcare costs, and reduced quality of life for survivors of critical illnesses [1,2]. Since these long-term complications are significant, reducing the ICU length of stay could ameliorate their incidence. Patients requiring mechanical ventilation often-present prolonged ICU stays compared to non-mechanically ventilated patients admitted in the ICU. Therefore, mechanical ventilation is a major driver of poor outcomes and a key target for early intervention.

Early physical therapy in the ICU has been shown to be a crucial intervention to mitigate these adverse outcomes [3–7]. By initiating mobilization and physical rehabilitation soon after ICU admission, studies have demonstrated improvements in muscle strength, a reduction in the duration of mechanical ventilation, and overall enhancement of functional recovery [3–7]. Early physical therapy reduces the deleterious effects of immobility, thus preventing the rapid onset of muscle atrophy and accelerating the weaning from mechanical

support. Consequently, the implementation of early physical therapy protocols is recognized as a vital component of ICU care [3–7].

Despite the established benefits of early physical therapy, there is a lack of randomized controlled trials (RCTs) evaluating the combined effects of physical, respiratory, occupational, and speech therapies within the first 24 to 72 hours after ICU admission. To our knowledge, no existing RCT has comprehensively assessed the synergistic impact of these four therapies initiated in this critical early period. Addressing this gap is essential for developing evidence-based protocols aimed at improving outcomes for ICU patients.

Methods: Participants, intervention and outcomes

The study protocol was prepared following the Standard Protocol Items: Recommendations for Interventional Trials (SPIRIT) [8]. The SPIRIT 2013 checklist and the full protocol (Spanish and its English translation) and can be consulted as S1, S2 and S3 Files, respectively. A summary of the schedule of enrolment, interventions and assessments can be consulted as Figure 1.

Figure 1. SPIRIT schedule of enrolment, interventions and assessments. HS: hospital stay; MRP: multimodal rehabilitation program; MS: mechanical ventilation. *Group allocation is based on the timing of interventions: Early MRP interventions start within the first 24 hours post-intubation, while Late MRP interventions begin after 72 hours. The frequency and duration of interventions are standardized across both groups: Speech therapy consists of 2 sessions of 10 minutes per day; Physical and Respiratory therapy involve 2 sessions of 30 minutes per day; and Occupational therapy includes 1 session of 30 minutes per day.

Research ethics approval

This study has been designed and will be conducted in accordance with the Helsinki Declaration [9], the Belmont Report [10], and the Colombian legislation (Resolución No. 8430 de 1993) [11]. The Ethics Committee at the Fundación Santa Fe de Bogotá approved this study on April 24, 2024 (CCEI-16320-2024). Protocols and amendments will be submitted to the Ethics Committee. Once approved, we will update the clinicaltrials.org registration (NCT06133504) and submit the amendments to this journal. Similarly, amendments will be communicated to researchers, clinical staff, and study participants.

The Ethics Committee audits the study twice a year, with progress reports submitted every three months. The interim analyses will be conducted by the Institutional Clinical Studies Office (in Spanish, Subdirección de Estudios Clínicos), which will provide a report to be submitted to the Ethics Committee. Based on the ethics committee's analysis, the trial may be suspended or terminated, considering protocol deviations and adverse events. Nonserious adverse events will be summarized and reported monthly, and the main investigator will immediately report all serious adverse events to the Ethics Committee within 24 hours of identifying the event.

ICU physicians, therapists, and nursing staff will be trained to obtain informed consent from eligible study participants or their legal representatives. Written informed consent will be obtained after an ICU physician or an ICU research team member has verified the inclusion and exclusion criteria and explained the study's purpose, intervention, duration, benefits, risks, confidentiality measures, and contact information to the participants or their legal representatives. The ICU physician will also clarify that participation is voluntary and will not affect the quality of their medical care if they choose not to participate or withdraw their consent at any time during the study. No compensation will be provided

to the study participants; however, patients experiencing adverse events or medical complications in any group will receive complete medical assistance. Written informed consent (in Spanish and English) can be found in S4 and S5 Files.

Personal data collected from study participants will be protected under confidentiality and personal data processing clauses stated in Colombian legislation (Ley No. 1581 de 2012) [12]. The collected data will be recorded and stored in REDCap, with exclusive access granted to the study researchers. The data will be anonymized to prevent identification of individuals. Third parties (data analysts, outcome assessors, and research auditors, among others) will only have access to anonymized datasets. However, the final trial dataset will be exclusively available to the ICU research team. After trial completion, the datasets and all related documentation will be stored for 20 years as required by Colombian legislation (Resolución No. 2378 de 2008) [13].

Study design and setting

A single center controlled clinical trial will be conducted with two groups of patients. The experimental group, also known as the "early multimodal rehabilitation program (MRP) arm", will start the MRP in the first 24 hours after

intubation. The control group, also known as the "late MRP" group, will start MRP 72 hours after intubation (Fig 2). Beyond the MRP starting time, clinical treatment will not vary between the groups.

Figure 2. Flow diagram of the study. APACHE II: Acute Physiology and Chronic Health disease Classification System II; ARDS: acute respiratory distress syndrome; ICU: intensive care unit; MRP: multimodal rehabilitation program; SAPS II: Simplified Acute Physiology Score II; SOFA: Sequential Organ Failure Assessment Score; T: time point.

Objective

Our main aim is to establish the efficacy and safety of early MRP compared with late MRP in mechanically ventilated patients admitted to the ICU. The main outcome is the difference in the duration of mechanical ventilation between the two groups. Safety will be assessed by comparing the number of serious and nonserious adverse events between groups. Additional secondary and exploratory efficacy outcomes will be measured and compared among groups.

Eligibility criteria

Inclusion criteria

Adult patients (≥18 years) with a Barthel score ≥70 admitted to the ICU who required invasive mechanical ventilation through an endotracheal tube for a period longer than 24 hours will be included. A Barthel score of ≥70 ensures that participants have a reasonable level of functional independence before ICU admission, allowing for accurate assessment of the impact of the rehabilitation intervention on post-ICU recovery. Requiring invasive mechanical ventilation for more than 24 hours targets a population with a sufficient duration of mechanical support to benefit from early rehabilitation interventions.

Exclusion criteria

- Invasive mechanical ventilation through tracheostomy or a nasotracheal tube.
- Head or neck surgery.
- Cardiac arrest.
- Airway burn.
- Burns involving ≥ 50% of the body surface area.
- Chronic obstructive pulmonary disease.
- Patients referred from another institution.

- Demyelinating diseases or neuromuscular junction disorders.
- Patients requiring neuromuscular blockade.
- Patients with a life expectancy of ≤180 days will be excluded from the study based on their primary diagnosis and comorbidities, the medical judgment of the ICU team, and their functional status as measured by the Barthel index.
- Patients who may not benefit from multimodal therapy according to medical criteria.
- ICU readmissions.
- Participation in other rehabilitation clinical trials.
- Liver or kidney transplant.
- Cytoreduction surgery (Sugarbaker procedure).

Interventions

The early MRP is defined as passive and active therapeutic interventions performed by physical, speech, respiratory, and occupational therapy teams, starting within the first 24 hours after orotracheal intubation. The Late MRP comprises the same interventions, but begins 72 hours after orotracheal intubation. These interventions are part of the standard care provided to all ICU patients and are routinely performed in our institution.

Each therapeutic intervention will be standardized based on the discipline (physical, speech, respiratory, or occupational therapy), and the patient's condition and tolerance. The specific intervention for each session will be adjusted according to the **Richmond Agitation-Sedation Scale (RASS) score** and functional assessments performed by the respective therapists.

RASS-Driven Protocol

The MRP intervention will be conducted following a RASS-driven protocol to optimize the daily therapeutic input and minimize downtime due to sedation or agitation. All therapy disciplines (physical, speech, respiratory, and occupational therapy) will provide interventions at each level of sedation, with the type and intensity of therapy adapted based on the RASS score (Table 1).

Table 1. Description of the multimodal rehabilitation program inteventions

		**Therapeutic discipline**			
**RASS score**		**Physical**	**Speech**	**Respiratory**	**Occupational**
≤ -2	At this deep sedation level,	Passive mobilization, muscle	Passive proprioceptive stimulation	Suctioning of secretions, passive	Multisensory stimulation (tactile,

	patients will receive passive and passive-assisted interventions	recruitment, joint mobilization, and reflex inhibition will be performed. The aim is to maintain joint mobility and prevent muscle atrophy.	(e.g., craniofacial alignment, jaw and tongue mobilization) and orofacial muscle stimulation will be conducted.	diaphragmatic training, and bronchial hygiene techniques will be provided to maintain pulmonary function.	proprioceptive, vestibular, gustatory, auditory, olfactory) will be applied to maintain sensory input and prevent cognitive decline.
-1 to +1	Patients who are more alert will engage in passive-assisted and active interventions	Active motor activation, bed mobility exercises, transfers, walking exercises, and electrostimulation for large muscle groups and respiratory muscles. The goal is to enhance functional mobility and strength.	Active neuromuscular stimulation, hydration of the oral mucosa, and exercises targeting supra- and infrahyoid muscles will be implemented.	Directed coughing, diaphragmatic exercises, incentive spirometry, and non-invasive respiratory muscle training will be applied to improve respiratory function and facilitate weaning from mechanical ventilation.	Active participation in daily tasks, focusing on sensory and cognitive tasks to enhance autonomy in functional movements such as reaching, grasping, and releasing.
≥ 2	At this level, most therapies will remain the same as in RASS -1 to +1, but occupational therapy will focus on behavioral modulation				Behavioral and environmental modulation will focus on calming techniques and reducing agitation through sensory stimuli and cognitive tasks, with family education for spatiotemporal orientation.

**Session duration (min)**	30	10	30	30
**Daily frecuency (number of sessions per day)**	2	2	2	1

Min: Minutes; RASS: Richmond Agitation-Sedation Scale.

Oxygenation Criteria for Intervention:

- Fraction of inspired oxygen (FiO2) ≤ 0.6.
- Peripheral oxygen saturation (SpO2) ≥ 90%.
- Respiratory rate ≤ 30 breaths per minute.
- Positive end-expiratory pressure (PEEP) ≤ 10 cmH2O.

Monitoring:

All staff involved will be trained to accurately assess the RASS score and other scales used in the study to reduce variability. Interventions will be documented consistently, and ICU monitoring or medical interventions that do not interfere with the study protocol will not be a cause for exclusion.

Participant withdrawal

Study participants will be withdrawn from the study in the following scenarios:

- Adverse events will be systematically recorded and categorized based on their severity and their potential relationship to the intervention. Serious

adverse events, such as altered blood pressure, cardiac arrhythmias leading to hemodynamic instability, oxygen desaturation, agitation, gastrointestinal complications, accidental removal of invasive lines or catheters, altered neurological status, accidental extubation, healthcare-associated pneumonia, and bronchoaspiration will be carefully monitored. Patients who experience serious adverse events or complications related to the study interventions may be withdrawn from the study for safety reasons. However, they will continue to receive full treatment in the ICU as part of standard care. The decisio[illegible]o withdraw participants is made solely for research purposes to prevent harm from study-specific interventions. All relevant data up to the point of withdrawal will be kept and included in the intention-to-treat analysis.

- Health complications: If a participant develops a health condition *de novo* that might affect their participation in the study (e.g., acute myocardial infarction, pulmonary embolism, intracerebral hemorrhage, cardiac dysfunction requiring inotropic or vasopressor support), they will be withdrawn from the study.

- Revocation of informed consent: Participants and their legal representatives have the right to revoke previously given consent to participate in this trial at any time. Medical attention will not have any repercussions.

- Early discontinuation of the trial: If early evidence indicates that early MRP is or is not beneficial in invasive mechanically ventilated patients, the study will be stopped, and the best MRP strategy will be offered to all patients. Similarly, if early evidence suggests that early MRP or late MRP is harmful, the study will be discontinued.

- Transference to another healthcare institution:

    - Data from patients transferred to another facility will be retained and if appropriate, it will be included in the intention to treat analysis. If the proportion of missing data due to the transfer is less than 20%, we will use data imputation methods to account for the missing values. This ensures that the contribution of these patients is not lost and that the sample size and statistical power are preserved. Transfers will be recorded as a separate variable, and

we will explore their potential impact on the study outcomes through sensitivity analyses.

Adherence strategies

We will implement educational strategies, audits, feedback, and reminders for all ICU staff. Educational strategies will focus on training nurses, physicians, and therapists on the informed consent process, study participant eligibility, and the interventions involved. A pilot study will be conducted to identify challenges in implementing the study protocol and to ensure comprehension of the processes and study forms. Based on the pilot study findings, we may adjust the initial forms and data collection tools.

Furthermore, we will monitor adherence to the MRP intervention among therapy staff via an electronic reporting system, which will also track RASS scores, vital signs before and after the intervention, session duration, and any adverse events. The reporting system will work as follows:

- Daily logs will be maintained by the clinical team, recording the frequency, duration, and type of interventions received by each study participant.

- A weekly review of these logs will be conducted by a monitoring committee to ensure adherence to the intervention protocol.
- Supervisors or study coordinators will periodically audit the therapeutic interventions to verify compliance with the study protocol.

Participants will be classified as adherent or non-adherent to the intervention according to the following criteria:

- Missed interventions: In case, the number of sessions missed exceeds more than 30% of the scheduled sessions during the ICU stay (due to health complications, scheduling conflicts, among other reasons) the patient will be classified as non-adherent.
- Session duration: Therapeutic sessions lasting less than 50% of the expected time (i.e., less than 15 minutes per session) are considered missing sessions. If this pattern repeats in more than 30% of scheduled sessions during the ICU, the patient will be classified as non-adherent.
- Incorrect interventions: Interventions applied incorrectly (e.g., applying active interventions to patients having a RASS score < -2) are considered missing sessions and follow the missing session criteria to classify patients as adherent or non-adherent.

The results will be communicated to the staff, and we will provide both general and individualized feedback while also gathering their feedback on the protocol implementation. If needed, additional training on specific topics will be offered to both the clinical staff and the research team.

Additionally, study reminders will be placed in the participant's room and the nurse station. Clinical staff will also have access to a 24-hour phone line for any trial-related questions.

Outcomes

We will collect the following baseline variables at the time of randomization (within the first 24 hours after intubation) to ensure comparability between groups: sociodemographic data (e.g., age, sex), clinical scores (Barthel, Sequential Organ Failure Assessment [SOFA] Score, Simplified Acute Physiology Score II [SAPS II], Acute Physiology and Chronic Health Disease Classification System II [APACHE II]), main diagnosis, body mass index, and ventilator parameters (tidal volume, respiratory rate, positive end-expiratory pressure, plateau pressure, airway resistance, and the ratio of

arterial oxygen partial pressure (PaO_2 in mmHg) to fractional inspired oxygen (PaO_2/FiO_2 ratio)).

Primary outcome

Our main outcomes are the difference in the duration of invasive mechanical ventilation, defined as the time (days) from orotracheal intubation to complete orothracheal extubation, between the experimental and control groups. We selected this outcome since it directly compares the efficacy of early MRP to that of late MRP for diminishing the time to extubation.

Safety outcomes

Concerning safety, we will measure the following adverse events and compare their occurrence between groups:

- Altered blood pressure: Decrease in systolic arterial pressure >30% or mean arterial pressure >15%.
- Cardiac arrhythmia leading to hemodynamic instability.
- Oxygen desaturation: Oxygen saturation <80% for at least 10 minutes.
- Gastrointestinal complications: nausea, vomiting or diarrhea.
- Agitation: RASS > 2 requiring sedation.

- Removal of invasive line.
- Altered neurological status: Decrease in Glasgow Coma Scale (GCS) <8.
- Accidental extubation.
- Healthcare-associated pneumonia.
- Bronchoaspiration.

Secondary outcomes

We will also measure differences between the experimental and control groups regarding the following outcomes at T4 (up to day 28 after intubation in case the patient remains hospitalized or up to hospital discharge, in case the hospitalization period lasted less than 28 days):

- Number of ventilator-free days (up to 28 days).
- Number of days with delirium (up to 28 days).
- Number of days with delirium in the ICU (up to 28 days).
- Number of days with delirium in the general ward (up to 28 days).
- Number of days under sedation (up to 28 days).
- Number of days without sedation (up to 28 days).
- Extubation failure.
- Need for noninvasive mechanical ventilation.

Exploratory outcomes

We will explore the following outcomes:

- Time from intubation to starting the MRP
- Muscle strength 24 hours postextubation.
- The highest level of mobility 24 hours postextubation.
- Grip strength 24 hours postextubation.
- Dysphagia 72 hours postextubation.
- Dysphonia 72 hours postextubation.
- The time from extubation to restarting oral intake.
- Requirement of noninvasive mechanical ventilation postextubation.
- Number of days hospitalized.
- Number of days in the ICU.
- Barthel score 28 days postintubation/hospital discharge.
- Cognitive function 28 days postintubation/hospital discharge.
- Activities of daily life assessment 28 days postintubation/hospital discharge.
- Rapid sensory and dexterity test 28 days postintubation/hospital discharge.

- Incidence of all-cause mortality up to 90 days postintubation.

Participant timeline

The data will be collected at six time points: before allocation, at the beginning of mechanical ventilation weaning, during the OMAHA+ assessment, 24-72 days postextubation, at hospital discharge or on the 28th day after orotracheal intubation, and 90 days after intubation (Figs 1 & 2). The list of variables and their descriptions and categorizations can be found in S2 and S3 Files.

Mechanical ventilation weaning will start when the following pre-requisite are accomplished:

- The indication to start mechanical ventilation has been successfully addressed.
- Patient is hemodynamically stable or the vasopressor/inotropic support is diminishing (in case the patient is on vasopressor/inotropic support).
- The awakening has started (diminishing sedative medications)
- FIO2 < 40% and SpO2 > 89%

The OMAHA+ assessment is an institutional checklist used in our ICU to identify patients, currently in the weaning of mechanical ventilation support process, who can be successfully extubated. It includes the Yang and Tobin

index, along with additional clinical, hemodynamic, and ventilatory parameters that reduce the risk of extubating failure and reintubation. To initiate the extubating process, a patient must meet at least 90% of the criteria on the OMAHA+ checklist. A full description of the OMAHA+ assessment can be found in S6 File.

Sample size

To estimate the sample size, we included previous parameters published by Schweickert and colleagues [14]. They reported that the mean duration of mechanical ventilation in patients receiving usual physical therapy was 6.1 (±1.4) days compared to 3.4 (±1.25) days in a group of patients receiving early therapy. Given an alpha error of 0.05 and a beta error of 0.9, we estimated the sample size according to the following formula:

$$n = \frac{2\,(\frac{\mathrm{Z\alpha}}{2} + Z\beta)^2 \;\times\; (\mathrm{SD1}^2 + \mathrm{SD2}^2)}{\mathrm{ES}^2}$$

where n is the sample size, $\frac{Z\alpha}{2}$ is the critical value of the normal distribution at α/2 (2.81), $Z\beta$ is the Zβ is the Z score associated with the beta level (1.28), $SD1$ is the standard deviation in the group receiving usual physical therapy, and $SD2$

is the standard deviation in the group receiving early physical therapy [14]. The effect size (ES) was estimated as follows:

$$ES = \frac{|\mu 1 - \mu 2|}{\sqrt{\frac{SD1^2 - SD2^2}{2}}}$$

where $\mu 1$ is the mean duration of mechanical ventilation in the group receiving usual physical therapy, $\mu 2$ is the mean duration in the group receiving early physical therapy, and $SD1$ and $SD2$ are the same standard deviations used in the first equation. By substituting the values into the second equation, the ES equals to 2.03.

$$ES = \frac{|\ 6.1 - 3.4\ |}{\sqrt{\frac{1.40^2 - 1.25^2}{2}}} = 2.03$$

Therefore, the resulting equation obtained by replacing the values in the first equation is:

$$n = \frac{2\ (2.81 + 1.28)^2\ \times\ (1.40^2 +\ 1.25^2)}{2.03^2} = 30.29 \sim 31\ patients$$

We estimated 31 patients per intervention group (62 patients in total). An additional 20% (6 patients per group) were added to minimize the loss of

statistical power in the case of patients withdrawing from the study or missing data.

Methods: Assignment of interventions

Recruitment and allocation

In 2023, more than 600 mechanically ventilated patients were admitted to our ICU. Consequently, we anticipate recruiting approximately 15 to 20 patients per month, accounting for exclusion criteria and the willingness of legal representatives or patients to participate in the study.

Study participants will be randomly assigned to each group using a stratified randomization strategy to ensure balance by age (<65 and ≥65 years), sex (male and female), and comorbidities (Charlson comorbidity index <8 and ≥8) resulting in eight strata. The allocation sequence will be generated through a computer-generated random numbers system, a function provided by REDCap. Study participants will be irreversible assign by REDCap to either the intervention group or the control group once eligibility is confirmed. The randomization process will be monitored and documented electronically within REDCap, ensuring transparency and minimizing the potential for bias.

Additionally, REDCap will maintain a log of all randomization events, which can be audited if necessary.

Given the nature of our study, which involves applying an MRP, it is not feasible to blind the intervention to either the medical staff or the patients. However, to minimize bias, the assessment of the primary outcome will be performed by an independent evaluator who is blinded to the group assignments of the participants. This evaluator will be responsible for assessing the outcome without knowledge of which intervention was applied. The statistical analysis will be conducted by an independent researcher who will have access to the data but will not be involved in the outcome assessment.

Methods: Data collection, management, and analysis

Data collection and management

The data will be collected at six time points (Fig 1). The case report form (CRF) does not include any personal data, and every study participant will be given a randomized identification number to anonymize the data. The CRF includes demographic and clinical variables (information from T0) and information already registered in the institutional electronic medical records system. Information from time points 1 and 2 will be recorded by a respiratory therapist. Each therapist, together with the ICU research team, will record data at time

points 3 and 4. Finally, an ICU research team member will contact the patients through a phone call or their relatives to verify their health status 90 days after intubation (T5).

Both the clinical and research teams will receive training on the proper completion of the case report form (CRF) and other study forms, which can be consulted as S7 File. Clear procedures will be established to ensure consistent data collection among the teams. The following strategies will be implemented:

- Data Verification: Two members of the clinical team will verify any extreme or outlier values identified between T0 and T4 to ensure accuracy and determine whether these values reflect true clinical outcomes or potential data entry errors.
- Electronic CRF: An electronic CRF and other study forms will be used to capture hours and dates related to each specific study time point, facilitating the monitoring of adherence to the study protocol.

If a patient provides their informed consent and wishes to withdraw from the study, we will inquire about their reasons for discontinuing participation. Similarly, if a patient is withdrawn or deviates from the study protocol for any

reason, this information will be summarized and published in the final report and scientific paper. Nonetheless, we will perform an intention-to-treat analysis and a sensitivity analysis that includes patient data up until the time point where they revoked consent or deviated from the protocol. All patients who experience serious adverse events will be included in the analysis.

Data management

Study data will be recorded in an electronic CRF on the REDCap platform (https://www.project-redcap.org/) using the randomized identification number assigned to each study participant. Relevant information from the institutional electronic medical records system will be extracted and entered into the electronic CRF.

To ensure data quality and reliability, we will implement the following strategies:

- Data Entry Checking: Verification of consistency, incongruities, reliability, outliers, and missing CRF data at the time of data entry in REDCap.

- Double Data Entry: Two independent researchers will enter the data, and the resulting databases will be compared to identify any discrepancies.

Statistical analysis

The data analysts performing the statistical analysis will be blinded to group allocation. The statistical analysis will be conducted as an intention-to-treat analysis. The normality of the quantitative variables will be assessed using the Shapiro–Wilk test. Normally distributed variables will be presented as means (standard deviations), and non-normally distributed variables as medians (interquartile ranges). Qualitative variables will be reported as frequencies and proportions.

To compare continuous variables between the two groups, a T-test or Mann-Whitney U test will be used depending on the distribution of the data, and a $\chi 2$ or Fisher's exact test will be used for categorical variables.

The primary outcome (duration of mechanical ventilation) will be evaluated by comparing the means between the two groups using a T-test (or Mann-Whitney U test if non-normal distribution is present). Additionally, we will explore the primary outcome using survival analysis methods to provide a complementary

understanding of the time-based data. A survival analys[illegible] and Cox regression will be performed to account for time on mechanical ventilation, with the intervention group (early vs. late) as the independent variable. A survival curve will be generated, and differences between the two groups will be analyzed using a log-rank test.

For patients who are not extubated by the end of the follow-up period, the time on mechanical ventilation will be censored at the last point of observation (up to 28 days). The variable will be interpreted as the time until extubation for those successfully extubated, and as the maximum duration of ventilation observed for censored patients.

To account for patients with ICU stays shorter than 72 hours, we will conduct a sensitivity analysis excluding these patients to assess how their inclusion or exclusion affects the results. Additionally, the duration of ICU stay will be included as a covariate in the primary analysis to control for its potential impact on the outcome of mechanical ventilation duration. We will also perform a subgroup analysis comparing patients with ICU stays shorter than 72 hours to those with longer stays, as well as adherent against non-adherent patients to assess any differential effects on the primary outcome.

To evaluate factors related to the time of mechanical ventilation, univariable Cox regression models will be performed for all variables included in the study. Continuous variables will be evaluated for linearity through the Martingale residual plot, and if linearity is not met, stratification will be applied based on plot interpretation. Variables with a p-value <0.25 and clinically important variables (e.g., age, sex, delirium) will be included in a multivariable Cox regression model, and the best model will be selected through a Backward Stepwise process based on Akaike (AIC) and Bayesian (BIC) information criteria.

Data Verification: Two clinical team members will verify any extreme or outlier values identified between T0 and T4 to ensure accuracy and determine whether these values reflect true clinical outcomes or potential data entry errors. Extreme values will be evaluated using regression models to identify leverage points. If removing a leverage point does not significantly modify the model, the extreme value will remain in the analysis as it may provide valuable information about the data.

Potential confounding factors will be evaluated through a second multiple regression model. In this model, the potentially confounding variable will be removed, and the resulting slope or estimator (adjusted estimate) will be calculated. If there is a change greater than 15%-20%, the confounding variable will be included in the final model.

To evaluate the interaction between two variables, another regression model will be performed, including a new variable resulting from multiplying the two potentially interacting variables. If the regression coefficient of this new variable has a significance level of less than 0.05, it is concluded that there is an interaction, and this variable will be included in the model.

The assumption of proportional hazard will be evaluated using Schoenfeld residuals, and influential values will be analyzed through residuals versus observations plots. Multicollinearity will be assessed through matrix curves.

Logistic regression models will be used to assess the relationship between dichotomous variables such as failed extubation, post-extubation non-invasive ventilation, prolonged mechanical ventilation, tracheostomy, and dysphonia. The assumptions of a logistic regression model, such as linearity in the log of

the odds, independence of errors, and absence of multicollinearity, will be evaluated through residual and graphical diagnostics.

A multiplicity adjustment strategy will be employed in statistical tests, such as the Bonferroni method or Holm-Bonferroni, to maintain the overall type 1 error rate at an acceptable level. A detailed exploration will be conducted through a sensitivity analysis of possible interactions between identified subgroups (e.g., RASS scale, protocol adherence). If no statistically significant differences are found, a post-hoc power analysis will assess whether the sample size was sufficient to detect differences in the primary and secondary outcomes.

Patients withdrawn due to complications will remain in the analysis under the intention-to-treat principle. This approach ensures the study's feasibility and allows for robust analysis of both primary and secondary outcomes. Subgroup analyses will also be conducted to explore the potential impact of complications on the study results.

Dissemination

Dissemination

We are committed to the complete and transparent publication of all study results, whether positive or negative. Once the data are analyzed, the results will be published in a peer-reviewed journal and presented at both Colombian and international events.

We will include as authors every individual who meets the criteria and recommendations proposed by McNutt and colleagues [15]. Once the manuscript is prepared, it will be submitted to American Journal Experts for grammar, style, and spell checking. No additional editing services or artificial intelligence will be used for manuscript enhancement.

Trial status

We have not yet recruited the first patient. We plan to start in December 2024 and expect to finish in December 2025. The data will be stored in our institutional Redcap platform and will be accessible upon reasonable request.

Supporting information

S1 Checklist. SPIRIT 2013 Checklist.

(PDF)

S2 Protocol. The Spanish protocol was approved by the research ethics committee.

(PDF)

S3 Protocol. The study protocol translated to English.

(PDF)

S4 Document. Informed Consent in Spanish.

(PDF)

S5 Document. Informed Consent in English.

(PDF)

S6 Checklist. OMAHA+ Assessment Checklist.

(PDF)

S7 CRF. Case report form.

(PDF)

Author Contributions

All the authors contributed to the conception and design of the study. AFMS will be the clinical trial coordinator. LMCM, JIAS and AFMS will enrol study participants. All authors will conduct the randomization and allocation. AVMN and AFMS will perform the follow-up. LMCM, JIAS, and AFMS will write the final report. All authors contributed to the critical revision of the manuscript. All the authors reviewed this protocol and agreed on its publication.

Data Availability Statement: All data generated or analyzed during this study will be available upon study completion and reasonable request. If the data cannot be made publicly accessible, this will be clearly stated along with the reasons. All data are in the manuscript and/or supporting information files.

Funding: This research received no specific grant from any funding agency in the public, commercial or not-for-profit sectors.

Competing interests: None declared.

References

1. Schwitzer E, Jensen KS, Brinkman L, DeFrancia L, VanVleet J, Baqi E, et al. Survival ≠ Recovery. CHEST Critical Care. 2023 Jun;1(1):100003.
2. Rawal G, Yadav S, Kumar R. Post-intensive care syndrome: An overview. J Transl Int Med. 2017 Jun 30;5(2):90–2.

3. Parry SM, Nydahl P, Needham DM. Implementing early physical rehabilitation and mobilisation in the ICU: institutional, clinician, and patient considerations. Intensive Care Med. 2018 Apr 1;44(4):470–3.

4. Menges D, Seiler B, Tomonaga Y, Schwenkglenks M, Puhan MA, Yebyo HG. Systematic early versus late mobilization or standard early mobilization in mechanically ventilated adult ICU patients: systematic review and meta-analysis. Crit Care. 2021 Dec 1;25(1).

5. Castro-Avila AC, Serón P, Fan E, Gaete M, Mickan S. Effect of early rehabilitation during intensive care unit stay on functional status: Systematic review and meta-analysis. PLoS One. 2015 Jul 1;10(7).

6. Berry MJ, Morris PE. Early exercise rehabilitation of muscle weakness in acute respiratory failure patients. Exerc Sport Sci Rev. 2013 Oct;41(4):208–15.

7. Zhang L, Hu W, Cai Z, Liu J, Wu J, Deng Y, et al. Early mobilization of critically ill patients in the intensive care unit: A systematic review and meta-analysis. PLoS One. 2019 Oct 1;14(10).

8. Chan AW, Tetzlaff JM, Altman DG, Dickersin K, Moher D. SPIRIT 2013: new guidance for content of clinical trial protocols. The Lancet. 2013 Jan;381(9861):91–2.

9. World Medical Association. World Medical Association Declaration of Helsinki Ethical Principles for Medical Research Involving Human Subjects. JAMA [Internet]. 2013 Nov 27;310(20):2191. Available from: https://oxford.universitypressscholarship.com/view/10.1093/acprof:oso/9780199241323.001.0001/acprof-9780199241323-chapter-25
10. Eckstein S. The Belmont Report: ethical principles and guidelines for the protection of human subjects of research. In: Manual for Research Ethics Committees. Cambridge University Press; 2003. p. 126–32.
11. Ministerio de Salud. Resolución 8430 de 1993 [Internet]. Bogotá: Ministerio de Salud; Oct 4, 1993 p. 1–19. Available from: https://www.minsalud.gov.co/sites/rid/Lists/BibliotecaDigital/RIDE/DE/DIJ/RESOLUCION-8430-DE-1993.PDF
12. Congreso de la República. Ley 1581 de 2012 [Internet]. Por la cual se dictan disposiciones generales para la protección de datos personales. Bogotá: Congreso de Colombia; Oct 17, 2012 p. 1–9. Available from: https://www.funcionpublica.gov.co/eva/gestornormativo/norma.php?i=49981
13. Ministerio de Protección Social. Resolución No. 2378 de 2008 [Internet]. s Buenas Prácticas Clínicas para las instituciones que conducen investigación con medicamentos en seres humanos 2008. Available from:

https://www.alcaldiabogota.gov.co/sisjur/normas/Norma1.jsp?i=31169&dt=S

14. Schweickert WD, Pohlman MC, Pohlman AS, Nigos C, Pawlik AJ, Esbrook CL, et al. Early physical and occupational therapy in mechanically ventilated, critically ill patients: a randomised controlled trial. The Lancet. 2009 May;373(9678):1874–82.

15. McNutt MK, Bradford M, Drazen JM, Hanson B, Howard B, Jamieson KH, et al. Transparency in authors' contributions and responsibilities to promote integrity in scientific publication. Proceedings of the National Academy of Sciences. 2018 Mar 13;115(11):2557–60.

 Click here to access/download;Figure;Fig 1.docx

	STUDY PERIOD						
	**Enrollment**	**Allocation**	**Post-allocation**				**Close-out**
**TIMEPOINT****	***-t_1***	**0**	***t_1***	***t_2***	***t_3***	***t_4***	***t_5***
**ENROLLMENT:**							
**Eligibility screen**	X						
**Informed consent**	X						
**Allocation**		X					
**INTERVENTIONS*:**							
Early MRP (≤24 hours)			◆────	────	────	────◆	
Late MRP (≥72 hours)			◆────	────	────	────◆	
**ASSESSMENTS:**							
Ventilation parameters	X	X					
Functional measurements					X	X	
Time from intubation to extubation					X		
Noninvasive Ventilation					X	X	
Extubation failure					X		
Delirium					X	X	
Days of MV, sedation, and HS						X	
Adverse events					X	X	
Mortality					X	X	X

Figure 1. SPIRIT schedule of enrolment, interventions and assessments. HS: hospital stay; MRP: multimodal rehabilitation program; MS: mechanical ventilation. *Group allocation is based on the timing of interventions: Early MRP interventions start within the first 24 hours post-intubation, while Late MRP interventions begin after 72 hours. The frequency and duration of interventions are standardized across both groups: Speech therapy consists of 2 sessions of 10 minutes per day; Physical and Respiratory therapy

involve 2 sessions of 30 minutes per day; and Occupational therapy includes 1 session of 30 minutes per day.

Figure 2

Click here to access/download;Figure;Fig 2.pdf

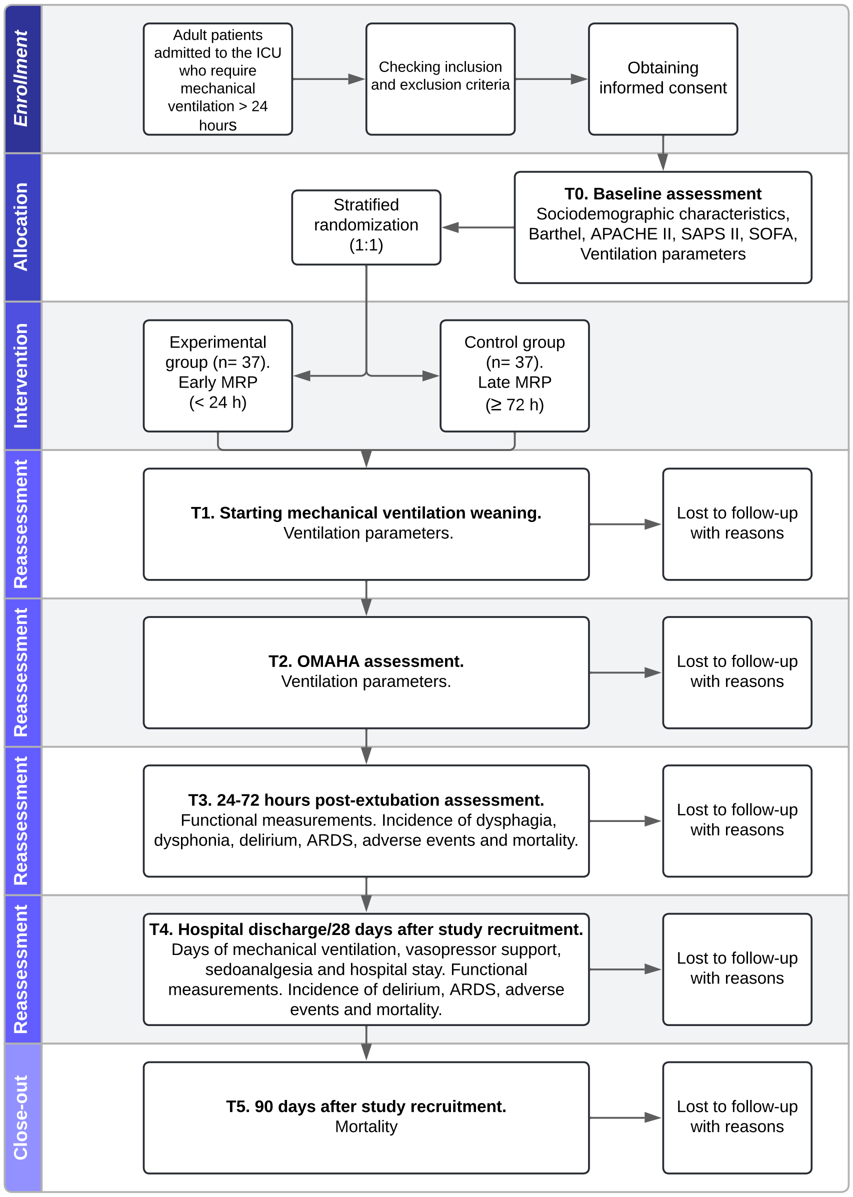

Supplementary material 1

Click here to access/download

Supporting Information

S1 Checklist. SPIRIT 2013 Checklist..pdf

Supplementary material 2

Click here to access/download
Supporting Information
S2 Protocol. Protocol in Spanish .pdf

Click here to access/download
Supporting Information
S4 Checklist. OMAHA+ Assessment Checklist..pdf

Supplementary material 5

Click here to access/download
Supporting Information
S5 CRF. Case report form..pdf

Click here to access/download
Supporting Information
S6 Document. Informed Consent in Spanish..pdf

Supplementary material 7

Click here to access/download
Supporting Information
S7 Document. Informed Consent in English..pdf

Click here to access/download
Supporting Information
S7_File.pdf

Click here to access/download
Supporting Information
S1_File.pdf

Click here to access/download
Supporting Information
S2_File.pdf

Click here to access/download
Supporting Information
S3_File.pdf

Click here to access/download
Supporting Information
S4_File.pdf

Click here to access/download
Supporting Information
S5_File.pdf

Click here to access/download
Supporting Information
S6_File.pdf

Supplementary material 3

Click here to access/download
Supporting Information
S3 Protocol. Protocol in English.pdf

Efficacy and safety of the early implementation of a multimodal rehabilitation program in mechanically ventilated patients: A randomized clinical trial protocol

Laura Maria Castillo Morales[1¶], Jorge Iván Alvarado-Sánchez[1,2¶*], Yenny Rocío Cárdenas-Bolivar[1], Valentina Montañez-Nariño[1], María Valentina Stozitzky-Ríos[1], Catherine Lissell Arévalo Guerrero[3], Miguel Leonardo Pulido Bobadilla[3], Diana Marcela Melo Rojas[3], Ana Gabriela López Rubio[4], Diana Carolina Ortiz Moreno[4], Paula Andrea Barreto Garzón[3], Marisol Murillo[3], Andrés Felipe Mora-Salamanca[1]

[1] Department of Critical Medicine and Intensive Care, Fundación Santa Fe de Bogotá, Bogotá, Colombia.

[2] Department of Physiology, Faculty of Medicine, Universidad Nacional de Colombia, Bogotá, Colombia.

[3] Department of Physical Medicine & Rehabilitation, Fundación Santa Fe de Bogotá, Bogotá, Colombia.

[4] Department of Pulmonary Diseases, Fundación Santa Fe de Bogotá, Bogotá, Colombia.

* Corresponding author:
Email: jialvarados@unal.edu.co (JIAS)

¶ These authors contributed equally to this work

Abstract

Background

Intensive care unit-acquired weakness (ICUAW) and post-intensive care syndrome (PICS) are significant complications among critically ill patients, leading to prolonged hospital stays, increased healthcare costs, and reduced quality of life. Early physical therapy in the ICU has shown promise in mitigating these adverse outcomes, yet randomized controlled trials (RCTs) evaluating the combined effects of physical, respiratory, occupational, and speech therapy initiated within the first 24 to 72 hours are lacking.

Methods

This single center, controlled clinical trial aims to establish the efficacy and safety of an early multimodal rehabilitation program (MRP) compared with a late MRP in mechanically ventilated patients admitted to the ICU. Adult patients (≥18 years) with a Barthel score ≥70 and requiring invasive mechanical ventilation for more than 24 hours will be included. Participants will be randomly assigned to early MRP, initiated within 24 hours post-intubation, or late MRP, starting 72 hours post-intubation. The primary outcome is the duration of mechanical ventilation. Safety will be assessed by comparing the number of adverse events between groups.

The MRP includes passive and active interventions from physical, speech, respiratory, and occupational therapy teams. Therapy intensity and type will be adjusted according to the patient's Richmond Agitation-Sedation Scale (RASS) score. Patients with lower RASS scores (indicating deeper sedation) will receive primarily passive interventions (e.g., passive range-of-motion exercises), while those with higher RASS scores (indicating lighter sedation or alertness) will engage into more active therapies (e.g., active-assisted exercises and functional mobility activities). This adaptive approach ensures patient safety and optimizes therapy based on the patient's current sedation level and clinical condition.

Dissemination

The results will be published in peer-reviewed journals and presented at conferences.

Trial registration

ClinicalTrials.gov, NCT06133504. Registered on November 11, 2023.

Introduction

Post-intensive care syndrome (PICS) is a significant complication that affects a large proportion of critically ill patients. PICS encompasses a range of physical, cognitive, and mental health impairments that persist long after discharge from the intensive care unit (ICU) [1]. These complications contribute to prolonged hospital stays, increased healthcare costs, and reduced quality of life for survivors of critical illnesses [1,2]. Since these long-term complications are significant, reducing the ICU length of stay could ameliorate their incidence. Patients requiring mechanical ventilation often-present prolonged ICU stays compared to non-mechanically ventilated patients admitted in the ICU. Therefore, mechanical ventilation is a major driver of poor outcomes and a key target for early interventions.

Early physical therapy in the ICU has been shown to be a crucial intervention to mitigate these adverse outcomes [3–7]. By initiating mobilization and physical rehabilitation soon after ICU admission, studies have demonstrated improvements in muscle strength, a reduction in the duration of mechanical ventilation, and overall enhancement of functional recovery [3–7]. Early physical therapy reduces the deleterious effects of immobility, thus preventing the rapid onset of muscle atrophy and accelerating the weaning from mechanical

support. Consequently, the implementation of early physical therapy protocols is recognized as a vital component of ICU care [3–7].

Despite the established benefits of early physical therapy, there is a lack of randomized controlled trials (RCTs) evaluating the combined effects of physical, respiratory, occupational, and speech therapies within the first 24 to 72 hours after ICU admission. To our knowledge, no existing RCT has comprehensively assessed the synergistic impact of these four therapies initiated in this critical early period. Addressing this gap is essential for developing evidence-based protocols aimed at improving outcomes for ICU patients.

Methods: Participants, intervention and outcomes

The study protocol was prepared following the Standard Protocol Items: Recommendations for Interventional Trials (SPIRIT) [8]. The SPIRIT 2013 checklist and the full protocol (Spanish and its English translation) and can be consulted as S1, S2 and S3 Files, respectively. A summary of the schedule of enrolment, interventions and assessments can be consulted as Figure 1.

Research ethics approval

This study has been designed and will be conducted in accordance with the Helsinki Declaration [9], the Belmont Report [10], and the Colombian legislation (Resolución No. 8430 de 1993) [11]. The Ethics Committee at the Fundación Santa Fe de Bogotá approved this study on April 24, 2024 (CCEI-16320-2024). Protocols and amendments will be submitted to the Ethics Committee. Once approved, we will update the clinicaltrials.org registration (NCT06133504) and submit the amendments to this journal. Similarly, amendments will be communicated to researchers, clinical staff, and study participants.

The Ethics Committee audits the study twice a year, with progress reports submitted every three months. The interim analyses will be conducted by the Institutional Clinical Studies Office (in Spanish, Subdirección de Estudios Clínicos), which will provide a report to be submitted to the Ethics Committee. Based on the ethics committee's analysis, the trial may be suspended or terminated, considering protocol deviations and adverse events. Nonserious adverse events will be summarized and reported monthly, and the main investigator will immediately report all serious adverse events to the Ethics Committee within 24 hours of identifying the event.

ICU physicians, therapists, and nursing staff will be trained to obtain informed consent from eligible study participants or their legal representatives. Written informed consent will be obtained after an ICU physician or an ICU research team member has verified the inclusion and exclusion criteria and explained the study's purpose, intervention, duration, benefits, risks, confidentiality measures, and contact information to the participants or their legal representatives. The ICU physician will also clarify that participation is voluntary and will not affect the quality of their medical care if they choose not to participate or withdraw their consent at any time during the study. No compensation will be provided to the study participants; however, patients experiencing adverse events or medical complications in any group will receive complete medical assistance. Written informed consent (in Spanish and English) can be found in S4 and S5 Files.

Personal data collected from study participants will be protected under confidentiality and personal data processing clauses stated in Colombian legislation (Ley No. 1581 de 2012) [12]. The collected data will be recorded and stored in REDCap, with exclusive access granted to the study researchers. The data will be anonymized to prevent identification of individuals. Third

parties (data analysts, outcome assessors, and research auditors, among others) will only have access to anonymized datasets. However, the final trial dataset will be exclusively available to the ICU research team. After trial completion, the datasets and all related documentation will be stored for 20 years as required by Colombian legislation (Resolución No. 2378 de 2008) [13].

Study design and setting

A single center controlled clinical trial will be conducted with two groups of patients. The experimental group, also known as the "early multimodal rehabilitation program (MRP) arm", will start the MRP in the first 24 hours after intubation. The control group, also known as the "late MRP" group, will start MRP 72 hours after intubation (Fig 2). Beyond the MRP starting time, clinical treatment will not vary between the groups.

Objective

Our main aim is to establish the efficacy and safety of early MRP compared with late MRP in mechanically ventilated patients admitted to the ICU. The main outcome is the difference in the duration of mechanical ventilation between the two groups. Safety will be assessed by comparing the number of

serious and nonserious adverse events between groups. Additional secondary and exploratory efficacy outcomes will be measured and compared among groups.

Eligibility criteria

Inclusion criteria

Adult patients (≥18 years) with a Barthel score ≥70 admitted to the ICU who required invasive mechanical ventilation through an endotracheal tube for a period longer than 24 hours will be included. A Barthel score of ≥70 ensures that participants have a reasonable level of functional independence before ICU admission, allowing for accurate assessment of the impact of the rehabilitation intervention on post-ICU recovery. Requiring invasive mechanical ventilation for more than 24 hours targets a population with a sufficient duration of mechanical support to benefit from early rehabilitation interventions.

Exclusion criteria

- Invasive mechanical ventilation through tracheostomy or a nasotracheal tube.
- Head or neck surgery.
- Cardiac arrest.

- Airway burn.
- Burns involving ≥ 50% of the body surface area.
- Chronic obstructive pulmonary disease.
- Patients referred from another institution.
- Demyelinating diseases or neuromuscular junction disorders.
- Patients requiring neuromuscular blockade.
- Patients with a life expectancy of ≤180 days will be excluded from the study based on their primary diagnosis and comorbidities, the medical judgment of the ICU team, and their functional status as measured by the Barthel index.
- Patients who may not benefit from multimodal therapy according to medical criteria.
- ICU readmissions.
- Participation in other rehabilitation clinical trials.
- Liver or kidney transplant.
- Cytoreduction surgery (Sugarbaker procedure).

Interventions

The early MRP is defined as passive and active therapeutic interventions performed by physical, speech, respiratory, and occupational therapy teams, starting within the first 24 hours after orotracheal intubation. The Late MRP comprises the same interventions, but begins 72 hours after orotracheal intubation. These interventions are part of the standard care provided to all ICU patients and are routinely performed in our institution.

Each therapeutic intervention will be standardized based on the discipline (physical, speech, respiratory, or occupational therapy), and the patient’s condition and tolerance. The specific intervention for each session will be adjusted according to the **Richmond Agitation-Sedation Scale (RASS) score** and functional assessments performed by the respective therapists.

RASS-Driven Protocol

The MRP intervention will be conducted following a RASS-driven protocol to optimize the daily therapeutic input and minimize downtime due to sedation or agitation. All therapy disciplines (physical, speech, respiratory, and occupational therapy) will provide interventions at each level of sedation, with the type and intensity of therapy adapted based on the RASS score (Table 1).

Table 1. Description of the multimodal rehabilitation program inteventions

		Therapeutic discipline			
RASS score		Physical	Speech	Respiratory	Occupational
≤ -2	At this deep sedation level, patients will receive passive and passive-assisted interventions	Passive mobilization, muscle recruitment, joint mobilization, and reflex inhibition will be performed. The aim is to maintain joint mobility and prevent muscle atrophy.	Passive proprioceptive stimulation (e.g., craniofacial alignment, jaw and tongue mobilization) and orofacial muscle stimulation will be conducted.	Suctioning of secretions, passive diaphragmatic training, and bronchial hygiene techniques will be provided to maintain pulmonary function.	Multisensory stimulation (tactile, proprioceptive, vestibular, gustatory, auditory, olfactory) will be applied to maintain sensory input and prevent cognitive decline.
-1 to +1	Patients who are more alert will engage in passive-assisted and active interventions	Active motor activation, bed mobility exercises, transfers, walking exercises, and electrostimulation for large muscle groups and respiratory muscles. The goal is to enhance functional mobility and strength.	Active neuromuscular stimulation, hydration of the oral mucosa, and exercises targeting supra- and infrahyoid muscles will be implemented.	Directed coughing, diaphragmatic exercises, incentive spirometry, and non-invasive respiratory muscle training will be applied to improve respiratory function and facilitate weaning from mechanical ventilation.	Active participation in daily tasks, focusing on sensory and cognitive tasks to enhance autonomy in functional movements such as reaching, grasping, and releasing.
≥ 2	At this level, most therapies will remain the same as				Behavioral and environmental modulation will focus on calming

	in RASS -1 to +1, but occupational therapy will focus on behavioral modulation				techniques and reducing agitation through sensory stimuli and cognitive tasks, with family education for spatiotemporal orientation.
**Session duration (min)**		30	10	30	30
**Daily frecuency (number of sessions per day)**		2	2	2	1

Min: Minutes; RASS: Richmond Agitation-Sedation Scale.

Oxygenation Criteria for Intervention:

- Fraction of inspired oxygen (FiO2) ≤ 0.6.
- Peripheral oxygen saturation (SpO2) ≥ 90%.
- Respiratory rate ≤ 30 breaths per minute.
- Positive end-expiratory pressure (PEEP) ≤ 10 cmH2O.

Monitoring:

All staff involved will be trained to accurately assess the RASS score and other scales used in the study to reduce variability. Interventions will be documented consistently, and ICU monitoring or medical interventions that do not interfere with the study protocol will not be a cause for exclusion.

Participant withdrawal

Study participants will be withdrawn from the study in the following scenarios:

- Adverse events will be systematically recorded and categorized based on their severity and their potential relationship to the intervention. Serious adverse events, such as altered blood pressure, cardiac arrhythmias leading to hemodynamic instability, oxygen desaturation, agitation, gastrointestinal complications, accidental removal of invasive lines or catheters, altered neurological status, accidental extubation, healthcare-associated pneumonia, and bronchoaspiration will be carefully monitored. Patients who experience serious adverse events or complications related to the study interventions may be withdrawn from the study for safety reasons. However, they will continue to receive full treatment in the ICU as part of standard care. The decision to withdraw participants is made solely for research purposes to prevent harm from study-specific interventions. All relevant data up to the point of withdrawal will be kept and included in the intention-to-treat analysis.

- Health complications: If a participant develops a health condition *de novo* that might affect their participation in the study (e.g., acute myocardial infarction, pulmonary embolism, intracerebral hemorrhage, cardiac

dysfunction requiring inotropic or vasopressor support), they will be withdrawn from the study.

- Revocation of informed consent: Participants and their legal representatives have the right to revoke previously given consent to participate in this trial at any time. Medical attention will not have any repercussions.

- Early discontinuation of the trial: If early evidence indicates that early MRP is or is not beneficial in invasive mechanically ventilated patients, the study will be stopped, and the best MRP strategy will be offered to all patients. Similarly, if early evidence suggests that early MRP or late MRP is harmful, the study will be discontinued.

- Transference to another healthcare institution:

    - Data from patients transferred to another facility will be retained and if appropriate, it will be included in the intention to treat analysis. If the proportion of missing data due to the transfer is less than 20%, we will use data imputation methods to account for the missing values. This ensures that the contribution of these patients

is not lost and that the sample size and statistical power are preserved. Transfers will be recorded as a separate variable, and we will explore their potential impact on the study outcomes through sensitivity analyses.

Adherence strategies

We will implement educational strategies, audits, feedback, and reminders for all ICU staff. Educational strategies will focus on training nurses, physicians, and therapists on the informed consent process, study participant eligibility, and the interventions involved. A pilot study will be conducted to identify challenges in implementing the study protocol and to ensure comprehension of the processes and study forms. Based on the pilot study findings, we may adjust the initial forms and data collection tools.

Furthermore, we will monitor adherence to the MRP intervention among therapy staff via an electronic reporting system, which will also track RASS scores, vital signs before and after the intervention, session duration, and any adverse events. The reporting system will work as follows:

- Daily logs will be maintained by the clinical team, recording the frequency, duration, and type of interventions received by each study participant.
- A weekly review of these logs will be conducted by a monitoring committee to ensure adherence to the intervention protocol.
- Supervisors or study coordinators will periodically audit the therapeutic interventions to verify compliance with the study protocol.

Participants will be classified as adherent or non-adherent to the intervention according to the following criteria:

- Missed interventions: In case, the number of sessions missed exceeds more than 30% of the scheduled sessions during the ICU stay (due to health complications, scheduling conflicts, among other reasons) the patient will be classified as non-adherent.
- Session duration: Therapeutic sessions lasting less than 50% of the expected time (i.e., less than 15 minutes per session) are considered missing sessions. If this pattern repeats in more than 30% of scheduled sessions during the ICU, the patient will be classified as non-adherent.

- Incorrect interventions: Interventions applied incorrectly (e.g., applying active interventions to patients having a RASS score < -2) are considered missing sessions and follow the missing session criteria to classify patients as adherent or non-adherent.

The results will be communicated to the staff, and we will provide both general and individualized feedback while also gathering their feedback on the protocol implementation. If needed, additional training on specific topics will be offered to both the clinical staff and the research team.

Additionally, study reminders will be placed in the participant's room and the nurse station. Clinical staff will also have access to a 24-hour phone line for any trial-related questions.

Outcomes

We will collect the following baseline variables at the time of randomization (within the first 24 hours after intubation) to ensure comparability between groups: sociodemographic data (e.g., age, sex), clinical scores (Barthel, Sequential Organ Failure Assessment [SOFA] Score, Simplified Acute Physiology Score II [SAPS II], Acute Physiology and Chronic

Health Disease Classification System II [APACHE II]), main diagnosis, body mass index, and ventilator parameters (tidal volume, respiratory rate, positive end-expiratory pressure, plateau pressure, airway resistance, and the ratio of arterial oxygen partial pressure (PaO2 in mmHg) to fractional inspired oxygen (PaO2/FiO2 ratio)).

Primary outcome

Our main outcomes are the difference in the duration of invasive mechanical ventilation, defined as the time (days) from orotracheal intubation to complete orothracheal extubation, between the experimental and control groups. We selected this outcome since it directly compares the efficacy of early MRP to that of late MRP for diminishing the time to extubation.

Safety outcomes

Concerning safety, we will measure the following adverse events and compare their occurrence between groups:

- Altered blood pressure: Decrease in systolic arterial pressure >30% or mean arterial pressure >15%.
- Cardiac arrhythmia leading to hemodynamic instability.

- Oxygen desaturation: Oxygen saturation <80% for at least 10 minutes.
- Gastrointestinal complications: nausea, vomiting or diarrhea.
- Agitation: RASS > 2 requiring sedation.
- Removal of invasive line.
- Altered neurological status: Decrease in Glasgow Coma Scale (GCS) <8.
- Accidental extubation.
- Healthcare-associated pneumonia.
- Bronchoaspiration.

Secondary outcomes

We will also measure differences between the experimental and control groups regarding the following outcomes at T4 (up to day 28 after intubation in case the patient remains hospitalized or up to hospital discharge, in case the hospitalization period lasted less than 28 days):

- Number of ventilator-free days (up to 28 days).
- Number of days with delirium (up to 28 days).
- Number of days with delirium in the ICU (up to 28 days).
- Number of days with delirium in the general ward (up to 28 days).
- Number of days under sedation (up to 28 days).

- Number of days without sedation (up to 28 days).
- Extubation failure.
- Need for noninvasive mechanical ventilation.

Exploratory outcomes

We will explore the following outcomes:

- Time from intubation to starting the MRP
- Muscle strength 24 hours postextubation.
- The highest level of mobility 24 hours postextubation.
- Grip strength 24 hours postextubation.
- Dysphagia 72 hours postextubation.
- Dysphonia 72 hours postextubation.
- The time from extubation to restarting oral intake.
- Requirement of noninvasive mechanical ventilation postextubation.
- Number of days hospitalized.
- Number of days in the ICU.
- Barthel score 28 days postintubation/hospital discharge.
- Cognitive function 28 days postintubation/hospital discharge.

- Activities of daily life assessment 28 days postintubation/hospital discharge.
- Rapid sensory and dexterity test 28 days postintubation/hospital discharge.
- Incidence of all-cause mortality up to 90 days postintubation.

Participant timeline

The data will be collected at six time points: before allocation, at the beginning of mechanical ventilation weaning, during the OMAHA+ assessment, 24-72 days postextubation, at hospital discharge or on the 28th day after orotracheal intubation, and 90 days after intubation (Figs 1 & 2). The list of variables and their descriptions and categorizations can be found in S2 and S3 Files.

Mechanical ventilation weaning will start when the following pre-requisite are accomplished:

- The indication to start mechanical ventilation has been successfully addressed.
- Patient is hemodynamically stable or the vasopressor/inotropic support is diminishing (in case the patient is on vasopressor/inotropic support).
- The awakening has started (diminishing sedative medications)

- FIO2 < 40% and SpO2 > 89%

The OMAHA+ assessment is an institutional checklist used in our ICU to identify patients, currently in the weaning of mechanical ventilation support process, who can be successfully extubated. It includes the Yang and Tobin index, along with additional clinical, hemodynamic, and ventilatory parameters that reduce the risk of extubating failure and reintubation. To initiate the extubating process, a patient must meet at least 90% of the criteria on the OMAHA+ checklist. A full description of the OMAHA+ assessment can be found in S6 File.

Sample size

To estimate the sample size, we included previous parameters published by Schweickert and colleagues [14]. They reported that the mean duration of mechanical ventilation in patients receiving usual physical therapy was 6.1 (±1.4) days compared to 3.4 (±1.25) days in a group of patients receiving early therapy. Given an alpha error of 0.05 and a beta error of 0.9, we estimated the sample size according to the following formula:

$$n = \frac{2\left(\frac{Z\alpha}{2} + Z\beta\right)^2 \times \left(\mathrm{SD1}^2 + \mathrm{SD2}^2\right)}{\mathrm{ES}^2}$$

where n is the sample size, $\frac{Z\alpha}{2}$ is the critical value of the normal distribution at α/2 (2.81), $Z\beta$ is the Zβ is the Z score associated with the beta level (1.28), $SD1$ is the standard deviation in the group receiving usual physical therapy, and $SD2$ is the standard deviation in the group receiving early physical therapy [14]. The effect size (ES) was estimated as follows:

$$ES = \frac{|\mu 1 - \mu 2|}{\sqrt{\frac{SD1^2 - SD2^2}{2}}}$$

where $\mu 1$ is the mean duration of mechanical ventilation in the group receiving usual physical therapy, $\mu 2$ is the mean duration in the group receiving early physical therapy, and $SD1$ and $SD2$ are the same standard deviations used in the first equation. By substituting the values into the second equation, the ES equals to 2.03.

$$ES = \frac{|\ 6.1 - 3.4\ |}{\sqrt{\frac{1.40^2 - 1.25^2}{2}}} = 2.03$$

Therefore, the resulting equation obtained by replacing the values in the first equation is:

$$n = \frac{2\,(2.81 + 1.28)^2 \times (1.40^2 + 1.25^2)}{2.03^2} = 30.29 \sim 31\ patients$$

We estimated 31 patients per intervention group (62 patients in total). An additional 20% (6 patients per group) were added to minimize the loss of statistical power in the case of patients withdrawing from the study or missing data.

Methods: Assignment of interventions

Recruitment and allocation

In 2023, more than 600 mechanically ventilated patients were admitted to our ICU. Consequently, we anticipate recruiting approximately 15 to 20 patients per month, accounting for exclusion criteria and the willingness of legal representatives or patients to participate in the study.

Study participants will be randomly assigned to each group using a stratified randomization strategy to ensure balance by age (<65 and ≥ 65 years), sex (male and female), and comorbidities (Charlson comorbidity index <8 and ≥ 8) resulting in eight strata. The allocation sequence will be generated through a computer-generated random numbers system, a function provided by REDCap. Study participants will be irreversible assign by REDCap to either

the intervention group or the control group once eligibility is confirmed. The randomization process will be monitored and documented electronically within REDCap, ensuring transparency and minimizing the potential for bias. Additionally, REDCap will maintain a log of all randomization events, which can be audited if necessary.

Given the nature of our study, which involves applying an MRP, it is not feasible to blind the intervention to either the medical staff or the patients. However, to minimize bias, the assessment of the primary outcome will be performed by an independent evaluator who is blinded to the group assignments of the participants. This evaluator will be responsible for assessing the outcome without knowledge of which intervention was applied. The statistical analysis will be conducted by an independent researcher who will have access to the data but will not be involved in the outcome assessment.

Methods: Data collection, management, and analysis

Data collection and management

The data will be collected at six time points (Fig 1). The case report form (CRF) does not include any personal data, and every study participant will be given a randomized identification number to anonymize the data. The CRF includes demographic and clinical variables (information from T0) and information

already registered in the institutional electronic medical records system. Information from time points 1 and 2 will be recorded by a respiratory therapist. Each therapist, together with the ICU research team, will record data at time points 3 and 4. Finally, an ICU research team member will contact the patients through a phone call or their relatives to verify their health status 90 days after intubation (T5).

Both the clinical and research teams will receive training on the proper completion of the case report form (CRF) and other study forms, which can be consulted as S7 File. Clear procedures will be established to ensure consistent data collection among the teams. The following strategies will be implemented:

- Data Verification: Two members of the clinical team will verify any extreme or outlier values identified between T0 and T4 to ensure accuracy and determine whether these values reflect true clinical outcomes or potential data entry errors.
- Electronic CRF: An electronic CRF and other study forms will be used to capture hours and dates related to each specific study time point, facilitating the monitoring of adherence to the study protocol.

If a patient provides their informed consent and wishes to withdraw from the study, we will inquire about their reasons for discontinuing participation. Similarly, if a patient is withdrawn or deviates from the study protocol for any reason, this information will be summarized and published in the final report and scientific paper. Nonetheless, we will perform an intention-to-treat analysis and a sensitivity analysis that includes patient data up until the time point where they revoked consent or deviated from the protocol. All patients who experience serious adverse events will be included in the analysis.

Data management

Study data will be recorded in an electronic CRF on the REDCap platform (https://www.project-redcap.org/) using the randomized identification number assigned to each study participant. Relevant information from the institutional electronic medical records system will be extracted and entered into the electronic CRF.

To ensure data quality and reliability, we will implement the following strategies:

- Data Entry Checking: Verification of consistency, incongruities, reliability, outliers, and missing CRF data at the time of data entry in REDCap.
- Double Data Entry: Two independent researchers will enter the data, and the resulting databases will be compared to identify any discrepancies.

Statistical analysis

The data analysts performing the statistical analysis will be blinded to group allocation. The statistical analysis will be conducted as an intention-to-treat analysis. The normality of the quantitative variables will be assessed using the Shapiro–Wilk test. Normally distributed variables will be presented as means (standard deviations), and non-normally distributed variables as medians (interquartile ranges). Qualitative variables will be reported as frequencies and proportions.

To compare continuous variables between the two groups, a T-test or Mann-Whitney U test will be used depending on the distribution of the data, and a $\chi2$ or Fisher's exact test will be used for categorical variables.

The primary outcome (duration of mechanical ventilation) will be evaluated by comparing the means between the two groups using a T-test (or Mann-Whitney U test if non-normal distribution is present). Additionally, we will explore the primary outcome using survival analysis methods to provide a complementary understanding of the time-based data. A survival analysis and Cox regression will be performed to account for time on mechanical ventilation, with the intervention group (early vs. late) as the independent variable. A survival curve will be generated, and differences between the two groups will be analyzed using a log-rank test.

For patients who are not extubated by the end of the follow-up period, the time on mechanical ventilation will be censored at the last point of observation (up to 28 days). The variable will be interpreted as the time until extubation for those successfully extubated, and as the maximum duration of ventilation observed for censored patients.

To account for patients with ICU stays shorter than 72 hours, we will conduct a sensitivity analysis excluding these patients to assess how their inclusion or exclusion affects the results. Additionally, the duration of ICU stay will be included as a covariate in the primary analysis to control for its potential impact

on the outcome of mechanical ventilation duration. We will also perform a subgroup analysis comparing patients with ICU stays shorter than 72 hours to those with longer stays, as well as adherent against non-adherent patients to assess any differential effects on the primary outcome.

To evaluate factors related to the time of mechanical ventilation, univariable Cox regression models will be performed for all variables included in the study. Continuous variables will be evaluated for linearity through the Martingale residual plot, and if linearity is not met, stratification will be applied based on plot interpretation. Variables with a p-value <0.25 and clinically important variables (e.g., age, sex, delirium) will be included in a multivariable Cox regression model, and the best model will be selected through a Backward Stepwise process based on Akaike (AIC) and Bayesian (BIC) information criteria.

Data Verification: Two clinical team members will verify any extreme or outlier values identified between T0 and T4 to ensure accuracy and determine whether these values reflect true clinical outcomes or potential data entry errors. Extreme values will be evaluated using regression models to identify leverage points. If removing a leverage point does not significantly modify the model,

the extreme value will remain in the analysis as it may provide valuable information about the data.

Potential confounding factors will be evaluated through a second multiple regression model. In this model, the potentially confounding variable will be removed, and the resulting slope or estimator (adjusted estimate) will be calculated. If there is a change greater than 15%-20%, the confounding variable will be included in the final model.

To evaluate the interaction between two variables, another regression model will be performed, including a new variable resulting from multiplying the two potentially interacting variables. If the regression coefficient of this new variable has a significance level of less than 0.05, it is concluded that there is an interaction, and this variable will be included in the model.

The assumption of proportional hazard will be evaluated using Schoenfeld residuals, and influential values will be analyzed through residuals versus observations plots. Multicollinearity will be assessed through matrix curves.

Logistic regression models will be used to assess the relationship between dichotomous variables such as failed extubation, post-extubation non-invasive ventilation, prolonged mechanical ventilation, tracheostomy, and dysphonia. The assumptions of a logistic regression model, such as linearity in the log of the odds, independence of errors, and absence of multicollinearity, will be evaluated through residual and graphical diagnostics.

A multiplicity adjustment strategy will be employed in statistical tests, such as the Bonferroni method or Holm-Bonferroni, to maintain the overall type 1 error rate at an acceptable level. A detailed exploration will be conducted through a sensitivity analysis of possible interactions between identified subgroups (e.g., RASS scale, protocol adherence). If no statistically significant differences are found, a post-hoc power analysis will assess whether the sample size was sufficient to detect differences in the primary and secondary outcomes.

Patients withdrawn due to complications will remain in the analysis under the intention-to-treat principle. This approach ensures the study's feasibility and allows for robust analysis of both primary and secondary outcomes. Subgroup analyses will also be conducted to explore the potential impact of complications on the study results.

Dissemination

Dissemination

We are committed to the complete and transparent publication of all study results, whether positive or negative. Once the data are analyzed, the results will be published in a peer-reviewed journal and presented at both Colombian and international events.

We will include as authors every individual who meets the criteria and recommendations proposed by McNutt and colleagues [15]. Once the manuscript is prepared, it will be submitted to American Journal Experts for grammar, style, and spell checking. No additional editing services or artificial intelligence will be used for manuscript enhancement.

Trial status

We have not yet recruited the first patient. We plan to start in October 2024 and expect to finish in December 2025. The data will be stored in our institutional Redcap platform and will be accessible upon reasonable request.

Supporting information

S1 Checklist. SPIRIT 2013 Checklist.

(PDF)

S2 Protocol. The Spanish protocol was approved by the research ethics committee.

(PDF)

S3 Protocol. The study protocol translated to English.

(PDF)

S4 Document. Informed Consent in Spanish.

(PDF)

S5 Document. Informed Consent in English.

(PDF)

S6 Checklist. OMAHA+ Assessment Checklist.

(PDF)

S7 CRF. Case report form.

(PDF)

Author Contributions

All the authors contributed to the conception and design of the study. AFMS will be the clinical trial coordinator. LMCM, JIAS and AFMS will enrol study participants. All authors will conduct the randomization and allocation. AVMN and AFMS will perform the follow-up. LMCM, JIAS, and AFMS will write the final report. All authors contributed to the critical revision of the manuscript. All the authors reviewed this protocol and agreed on its publication.

Data Availability Statement: All data generated or analyzed during this study will be available upon study completion and reasonable request. If the data

cannot be made publicly accessible, this will be clearly stated along with the reasons. Currently, all relevant data are included in the manuscript or supporting information files.

Funding: This research received no specific grant from any funding agency in the public, commercial or not-for-profit sectors.

Competing interests: None declared.

References

1. Schwitzer E, Jensen KS, Brinkman L, DeFrancia L, VanVleet J, Baqi E, et al. Survival ≠ Recovery. CHEST Critical Care. 2023 Jun;1(1):100003.

2. Rawal G, Yadav S, Kumar R. Post-intensive care syndrome: An overview. J Transl Int Med. 2017 Jun 30;5(2):90–2.

3. Parry SM, Nydahl P, Needham DM. Implementing early physical rehabilitation and mobilisation in the ICU: institutional, clinician, and patient considerations. Intensive Care Med. 2018 Apr 1;44(4):470–3.

4. Menges D, Seiler B, Tomonaga Y, Schwenkglenks M, Puhan MA, Yebyo HG. Systematic early versus late mobilization or standard early mobilization in mechanically ventilated adult ICU patients: systematic review and meta-analysis. Crit Care. 2021 Dec 1;25(1).

5. Castro-Avila AC, Serón P, Fan E, Gaete M, Mickan S. Effect of early rehabilitation during intensive care unit stay on functional status: Systematic review and meta-analysis. PLoS One. 2015 Jul 1;10(7).

6. Berry MJ, Morris PE. Early exercise rehabilitation of muscle weakness in acute respiratory failure patients. Exerc Sport Sci Rev. 2013 Oct;41(4):208–15.

7. Zhang L, Hu W, Cai Z, Liu J, Wu J, Deng Y, et al. Early mobilization of critically ill patients in the intensive care unit: A systematic review and meta-analysis. PLoS One. 2019 Oct 1;14(10).

8. Chan AW, Tetzlaff JM, Altman DG, Dickersin K, Moher D. SPIRIT 2013: new guidance for content of clinical trial protocols. The Lancet. 2013 Jan;381(9861):91–2.

9. World Medical Association. World Medical Association Declaration of Helsinki Ethical Principles for Medical Research Involving Human Subjects. JAMA [Internet]. 2013 Nov 27;310(20):2191. Available from: https://oxford.universitypressscholarship.com/view/10.1093/acprof:oso/9780199241323.001.0001/acprof-9780199241323-chapter-25

10. Eckstein S. The Belmont Report: ethical principles and guidelines for the protection of human subjects of research. In: Manual for Research Ethics Committees. Cambridge University Press; 2003. p. 126–32.

11. Ministerio de Salud. Resolución 8430 de 1993 [Internet]. Bogotá: Ministerio de Salud; Oct 4, 1993 p. 1–19. Available from: https://www.minsalud.gov.co/sites/rid/Lists/BibliotecaDigital/RIDE/DE/DIJ/RESOLUCION-8430-DE-1993.PDF

12. Congreso de la República. Ley 1581 de 2012 [Internet]. Por la cual se dictan disposiciones generales para la protección de datos personales. Bogotá: Congreso de Colombia; Oct 17, 2012 p. 1–9. Available from: https://www.funcionpublica.gov.co/eva/gestornormativo/norma.php?i=49981

13. Ministerio de Protección Social. Resolución No. 2378 de 2008 [Internet]. s Buenas Prácticas Clínicas para las instituciones que conducen investigación con medicamentos en seres humanos 2008. Available from: https://www.alcaldiabogota.gov.co/sisjur/normas/Norma1.jsp?i=31169&dt=S

14. Schweickert WD, Pohlman MC, Pohlman AS, Nigos C, Pawlik AJ, Esbrook CL, et al. Early physical and occupational therapy in mechanically ventilated, critically ill patients: a randomised controlled trial. The Lancet. 2009 May;373(9678):1874–82.

15. McNutt MK, Bradford M, Drazen JM, Hanson B, Howard B, Jamieson KH, et al. Transparency in authors' contributions and responsibilities to promote integrity in scientific publication. Proceedings of the National Academy of Sciences. 2018 Mar 13;115(11):2557–60.

Efficacy and safety of the early implementation of a multimodal rehabilitation program in mechanically ventilated patients: A randomized clinical trial protocol

Laura Maria Castillo Morales[1¶], Jorge Iván Alvarado-Sánchez[1,2¶*], Yenny Rocío Cárdenas-Bolivar[1], Valentina Montañez-Nariño[1], María Valentina Stozitzky-Ríos[1], Catherine Lissell Arévalo Guerrero[3], Miguel Leonardo Pulido Bobadilla[3], Diana Marcela Melo Rojas[3], Ana Gabriela López Rubio[4], Diana Carolina Ortiz Moreno[4], Paula Andrea Barreto Garzón[3], Marisol Murillo[3], Andrés Felipe Mora-Salamanca[1]

[1] Department of Critical Medicine and Intensive Care, Fundación Santa Fe de Bogotá, Bogotá, Colombia.

[2] Department of Physiology, Faculty of Medicine, Universidad Nacional de Colombia, Bogotá, Colombia.

[3] Department of Physical Medicine & Rehabilitation, Fundación Santa Fe de Bogotá, Bogotá, Colombia.

[4] Department of Pulmonary Diseases, Fundación Santa Fe de Bogotá, Bogotá, Colombia.

* Corresponding author:
Email: jialvarados@unal.edu.co (JIAS)

¶ These authors contributed equally to this work

// Abstract

Background

Intensive care unit-acquired weakness (ICUAW) and post-intensive care syndrome (PICS) are significant complications among critically ill patients, leading to prolonged hospital stays, increased healthcare costs, and reduced quality of life. Early physical therapy in the ICU has shown promise in mitigating these adverse outcomes, yet randomized controlled trials (RCTs) evaluating the combined effects of physical, respiratory, occupational, and speech therapy initiated within the first 24 to 72 hours are lacking.

Methods

This single center, controlled clinical trial aims to establish the efficacy and safety of an early multimodal rehabilitation program (MRP) compared with a late MRP in mechanically ventilated patients admitted to the ICU. Adult patients (≥18 years) with a Barthel score ≥70 and requiring invasive mechanical ventilation for more than 24 hours will be included. Participants will be randomly assigned to early MRP, initiated within 24 hours post-intubation, or late MRP, starting 72 hours post-intubation. The primary outcome is the duration of mechanical ventilation. Safety will be assessed by comparing the number of adverse events between groups.

The MRP includes passive and active interventions from physical, speech, respiratory, and occupational therapy teams. Therapy intensity and type will be adjusted according to the patient's Richmond Agitation-Sedation Scale (RASS) score. Patients with lower RASS scores (indicating deeper sedation) will receive primarily passive interventions (e.g., passive range-of-motion exercises), while those with higher RASS scores (indicating lighter sedation or alertness) will engage into more active therapies (e.g., active-assisted exercises and functional mobility activities). This adaptive approach ensures patient safety and optimizes therapy based on the patient's current sedation level and clinical condition.

Dissemination

The results will be published in peer-reviewed journals and presented at conferences.

Trial registration

ClinicalTrials.gov, NCT06133504. Registered on November 11, 2023.

Introduction

Post-intensive care syndrome (PICS) is a significant complication that affects a large proportion of critically ill patients. PICS encompasses a range of physical, cognitive, and mental health impairments that persist long after discharge from the intensive care unit (ICU) [1]. These complications contribute to prolonged hospital stays, increased healthcare costs, and reduced quality of life for survivors of critical illnesses [1,2]. Since these long-term complications are significant, reducing the ICU length of stay could ameliorate their incidence. Patients requiring mechanical ventilation often-present prolonged ICU stays compared to non-mechanically ventilated patients admitted in the ICU. Therefore, mechanical ventilation is a major driver of poor outcomes and a key target for early interventions.

Early physical therapy in the ICU has been shown to be a crucial intervention to mitigate these adverse outcomes [3–7]. By initiating mobilization and physical rehabilitation soon after ICU admission, studies have demonstrated improvements in muscle strength, a reduction in the duration of mechanical ventilation, and overall enhancement of functional recovery [3–7]. Early physical therapy reduces the deleterious effects of immobility, thus preventing the rapid onset of muscle atrophy and accelerating the weaning from mechanical

support. Consequently, the implementation of early physical therapy protocols is recognized as a vital component of ICU care [3–7].

Despite the established benefits of early physical therapy, there is a lack of randomized controlled trials (RCTs) evaluating the combined effects of physical, respiratory, occupational, and speech therapies within the first 24 to 72 hours after ICU admission. To our knowledge, no existing RCT has comprehensively assessed the synergistic impact of these four therapies initiated in this critical early period. Addressing this gap is essential for developing evidence-based protocols aimed at improving outcomes for ICU patients.

Methods: Participants, intervention and outcomes

The study protocol was prepared following the Standard Protocol Items: Recommendations for Interventional Trials (SPIRIT) [8]. The SPIRIT 2013 checklist and the full protocol (Spanish and its English translation) and can be consulted as S1, S2 and S3 Files, respectively. A summary of the schedule of enrolment, interventions and assessments can be consulted as Figure 1.

Figure 1. SPIRIT schedule of enrolment, interventions and assessments. HS: hospital stay; MRP: multimodal rehabilitation program; MS: mechanical ventilation. *Group allocation is based on the timing of interventions: Early MRP interventions start within the first 24 hours post-intubation, while Late MRP interventions begin after 72 hours. The frequency and duration of interventions are standardized across both groups: Speech therapy consists of 2 sessions of 10 minutes per day; Physical and Respiratory therapy involve 2 sessions of 30 minutes per day; and Occupational therapy includes 1 session of 30 minutes per day.

Research ethics approval

This study has been designed and will be conducted in accordance with the Helsinki Declaration [9], the Belmont Report [10], and the Colombian legislation (Resolución No. 8430 de 1993) [11]. The Ethics Committee at the Fundación Santa Fe de Bogotá approved this study on April 24, 2024 (CCEI-16320-2024). Protocols and amendments will be submitted to the Ethics Committee. Once approved, we will update the clinicaltrials.org registration (NCT06133504) and submit the amendments to this journal. Similarly, amendments will be communicated to researchers, clinical staff, and study participants.

The Ethics Committee audits the study twice a year, with progress reports submitted every three months. The interim analyses will be conducted by the Institutional Clinical Studies Office (in Spanish, Subdirección de Estudios Clínicos), which will provide a report to be submitted to the Ethics Committee. Based on the ethics committee's analysis, the trial may be suspended or terminated, considering protocol deviations and adverse events. Nonserious adverse events will be summarized and reported monthly, and the main investigator will immediately report all serious adverse events to the Ethics Committee within 24 hours of identifying the event.

ICU physicians, therapists, and nursing staff will be trained to obtain informed consent from eligible study participants or their legal representatives. Written informed consent will be obtained after an ICU physician or an ICU research team member has verified the inclusion and exclusion criteria and explained the study's purpose, intervention, duration, benefits, risks, confidentiality measures, and contact information to the participants or their legal representatives. The ICU physician will also clarify that participation is voluntary and will not affect the quality of their medical care if they choose not to participate or withdraw their consent at any time during the study. No compensation will be provided

to the study participants; however, patients experiencing adverse events or medical complications in any group will receive complete medical assistance. Written informed consent (in Spanish and English) can be found in S4 and S5 Files.

Personal data collected from study participants will be protected under confidentiality and personal data processing clauses stated in Colombian legislation (Ley No. 1581 de 2012) [12]. The collected data will be recorded and stored in REDCap, with exclusive access granted to the study researchers. The data will be anonymized to prevent identification of individuals. Third parties (data analysts, outcome assessors, and research auditors, among others) will only have access to anonymized datasets. However, the final trial dataset will be exclusively available to the ICU research team. After trial completion, the datasets and all related documentation will be stored for 20 years as required by Colombian legislation (Resolución No. 2378 de 2008) [13].

Study design and setting

A single center controlled clinical trial will be conducted with two groups of patients. The experimental group, also known as the "early multimodal rehabilitation program (MRP) arm", will start the MRP in the first 24 hours after

intubation. The control group, also known as the "late MRP" group, will start MRP 72 hours after intubation (Fig 2). Beyond the MRP starting time, clinical treatment will not vary between the groups.

Figure 2. Flow diagram of the study. APACHE II: Acute Physiology and Chronic Health disease Classification System II; ARDS: acute respiratory distress syndrome; ICU: intensive care unit; MRP: multimodal rehabilitation program; SAPS II: Simplified Acute Physiology Score II; SOFA: Sequential Organ Failure Assessment Score; T: time point.

Objective

Our main aim is to establish the efficacy and safety of early MRP compared with late MRP in mechanically ventilated patients admitted to the ICU. The main outcome is the difference in the duration of mechanical ventilation between the two groups. Safety will be assessed by comparing the number of serious and nonserious adverse events between groups. Additional secondary and exploratory efficacy outcomes will be measured and compared among groups.

Eligibility criteria

Inclusion criteria

Adult patients (≥18 years) with a Barthel score ≥70 admitted to the ICU who required invasive mechanical ventilation through an endotracheal tube for a period longer than 24 hours will be included. A Barthel score of ≥70 ensures that participants have a reasonable level of functional independence before ICU admission, allowing for accurate assessment of the impact of the rehabilitation intervention on post-ICU recovery. Requiring invasive mechanical ventilation for more than 24 hours targets a population with a sufficient duration of mechanical support to benefit from early rehabilitation interventions.

Exclusion criteria

- Invasive mechanical ventilation through tracheostomy or a nasotracheal tube.
- Head or neck surgery.
- Cardiac arrest.
- Airway burn.
- Burns involving ≥ 50% of the body surface area.
- Chronic obstructive pulmonary disease.
- Patients referred from another institution.

- Demyelinating diseases or neuromuscular junction disorders.
- Patients requiring neuromuscular blockade.
- Patients with a life expectancy of ≤180 days will be excluded from the study based on their primary diagnosis and comorbidities, the medical judgment of the ICU team, and their functional status as measured by the Barthel index.
- Patients who may not benefit from multimodal therapy according to medical criteria.
- ICU readmissions.
- Participation in other rehabilitation clinical trials.
- Liver or kidney transplant.
- Cytoreduction surgery (Sugarbaker procedure).

Interventions

The early MRP is defined as passive and active therapeutic interventions performed by physical, speech, respiratory, and occupational therapy teams, starting within the first 24 hours after orotracheal intubation. The Late MRP comprises the same interventions, but begins 72 hours after orotracheal intubation. These interventions are part of the standard care provided to all ICU patients and are routinely performed in our institution.

Each therapeutic intervention will be standardized based on the discipline (physical, speech, respiratory, or occupational therapy), and the patient's condition and tolerance. The specific intervention for each session will be adjusted according to the **Richmond Agitation-Sedation Scale (RASS) score** and functional assessments performed by the respective therapists.

RASS-Driven Protocol

The MRP intervention will be conducted following a RASS-driven protocol to optimize the daily therapeutic input and minimize downtime due to sedation or agitation. All therapy disciplines (physical, speech, respiratory, and occupational therapy) will provide interventions at each level of sedation, with the type and intensity of therapy adapted based on the RASS score (Table 1).

Table 1. Description of the multimodal rehabilitation program inteventions

		**Therapeutic discipline**			
**RASS score**		**Physical**	**Speech**	**Respiratory**	**Occupational**
≤ -2	At this deep sedation level,	Passive mobilization, muscle	Passive proprioceptive stimulation	Suctioning of secretions, passive	Multisensory stimulation (tactile,

<table>
<tr><td></td><td>patients will receive passive and passive-assisted interventions</td><td>recruitment, joint mobilization, and reflex inhibition will be performed. The aim is to maintain joint mobility and prevent muscle atrophy.</td><td>(e.g., craniofacial alignment, jaw and tongue mobilization) and orofacial muscle stimulation will be conducted.</td><td>diaphragmatic training, and bronchial hygiene techniques will be provided to maintain pulmonary function.</td><td>proprioceptive, vestibular, gustatory, auditory, olfactory) will be applied to maintain sensory input and prevent cognitive decline.</td></tr>
<tr><td>-1 to +1</td><td>Patients who are more alert will engage in passive-assisted and active interventions</td><td rowspan="2">Active motor activation, bed mobility exercises, transfers, walking exercises, and electrostimulation for large muscle groups and respiratory muscles. The goal is to enhance functional mobility and strength.</td><td rowspan="2">Active neuromuscular stimulation, hydration of the oral mucosa, and exercises targeting supra- and infrahyoid muscles will be implemented.</td><td rowspan="2">Directed coughing, diaphragmatic exercises, incentive spirometry, and non-invasive respiratory muscle training will be applied to improve respiratory function and facilitate weaning from mechanical ventilation.</td><td>Active participation in daily tasks, focusing on sensory and cognitive tasks to enhance autonomy in functional movements such as reaching, grasping, and releasing.</td></tr>
<tr><td>≥ 2</td><td>At this level, most therapies will remain the same as in RASS -1 to +1, but occupational therapy will focus on behavioral modulation</td><td>Behavioral and environmental modulation will focus on calming techniques and reducing agitation through sensory stimuli and cognitive tasks, with family education for spatiotemporal orientation.</td></tr>
</table>

**Session duration (min)**	30	10	30	30
**Daily frecuency (number of sessions per day)**	2	2	2	1

Min: Minutes; RASS: Richmond Agitation-Sedation Scale.

Oxygenation Criteria for Intervention:

- Fraction of inspired oxygen (FiO_2) ≤ 0.6.
- Peripheral oxygen saturation (SpO_2) ≥ 90%.
- Respiratory rate ≤ 30 breaths per minute.
- Positive end-expiratory pressure (PEEP) ≤ 10 cmH_2O.

Monitoring:

All staff involved will be trained to accurately assess the RASS score and other scales used in the study to reduce variability. Interventions will be documented consistently, and ICU monitoring or medical interventions that do not interfere with the study protocol will not be a cause for exclusion.

Participant withdrawal

Study participants will be withdrawn from the study in the following scenarios:

- Adverse events will be systematically recorded and categorized based on their severity and their potential relationship to the intervention. Serious

adverse events, such as altered blood pressure, cardiac arrhythmias leading to hemodynamic instability, oxygen desaturation, agitation, gastrointestinal complications, accidental removal of invasive lines or catheters, altered neurological status, accidental extubation, healthcare-associated pneumonia, and bronchoaspiration will be carefully monitored. Patients who experience serious adverse events or complications related to the study interventions may be withdrawn from the study for safety reasons. However, they will continue to receive full treatment in the ICU as part of standard care. The decision to withdraw participants is made solely for research purposes to prevent harm from study-specific interventions. All relevant data up to the point of withdrawal will be kept and included in the intention-to-treat analysis.

- Health complications: If a participant develops a health condition *de novo* that might affect their participation in the study (e.g., acute myocardial infarction, pulmonary embolism, intracerebral hemorrhage, cardiac dysfunction requiring inotropic or vasopressor support), they will be withdrawn from the study.

- Revocation of informed consent: Participants and their legal representatives have the right to revoke previously given consent to participate in this trial at any time. Medical attention will not have any repercussions.

- Early discontinuation of the trial: If early evidence indicates that early MRP is or is not beneficial in invasive mechanically ventilated patients, the study will be stopped, and the best MRP strategy will be offered to all patients. Similarly, if early evidence suggests that early MRP or late MRP is harmful, the study will be discontinued.

- Transference to another healthcare institution:

    - Data from patients transferred to another facility will be retained and if appropriate, it will be included in the intention to treat analysis. If the proportion of missing data due to the transfer is less than 20%, we will use data imputation methods to account for the missing values. This ensures that the contribution of these patients is not lost and that the sample size and statistical power are preserved. Transfers will be recorded as a separate variable, and

we will explore their potential impact on the study outcomes through sensitivity analyses.

Adherence strategies

We will implement educational strategies, audits, feedback, and reminders for all ICU staff. Educational strategies will focus on training nurses, physicians, and therapists on the informed consent process, study participant eligibility, and the interventions involved. A pilot study will be conducted to identify challenges in implementing the study protocol and to ensure comprehension of the processes and study forms. Based on the pilot study findings, we may adjust the initial forms and data collection tools.

Furthermore, we will monitor adherence to the MRP intervention among therapy staff via an electronic reporting system, which will also track RASS scores, vital signs before and after the intervention, session duration, and any adverse events. The reporting system will work as follows:

- Daily logs will be maintained by the clinical team, recording the frequency, duration, and type of interventions received by each study participant.

- A weekly review of these logs will be conducted by a monitoring committee to ensure adherence to the intervention protocol.
- Supervisors or study coordinators will periodically audit the therapeutic interventions to verify compliance with the study protocol.

Participants will be classified as adherent or non-adherent to the intervention according to the following criteria:

- Missed interventions: In case, the number of sessions missed exceeds more than 30% of the scheduled sessions during the ICU stay (due to health complications, scheduling conflicts, among other reasons) the patient will be classified as non-adherent.
- Session duration: Therapeutic sessions lasting less than 50% of the expected time (i.e., less than 15 minutes per session) are considered missing sessions. If this pattern repeats in more than 30% of scheduled sessions during the ICU, the patient will be classified as non-adherent.
- Incorrect interventions: Interventions applied incorrectly (e.g., applying active interventions to patients having a RASS score < -2) are considered missing sessions and follow the missing session criteria to classify patients as adherent or non-adherent.

The results will be communicated to the staff, and we will provide both general and individualized feedback while also gathering their feedback on the protocol implementation. If needed, additional training on specific topics will be offered to both the clinical staff and the research team.

Additionally, study reminders will be placed in the participant's room and the nurse station. Clinical staff will also have access to a 24-hour phone line for any trial-related questions.

Outcomes

We will collect the following baseline variables at the time of randomization (within the first 24 hours after intubation) to ensure comparability between groups: sociodemographic data (e.g., age, sex), clinical scores (Barthel, Sequential Organ Failure Assessment [SOFA] Score, Simplified Acute Physiology Score II [SAPS II], Acute Physiology and Chronic Health Disease Classification System II [APACHE II]), main diagnosis, body mass index, and ventilator parameters (tidal volume, respiratory rate, positive end-expiratory pressure, plateau pressure, airway resistance, and the ratio of

arterial oxygen partial pressure (PaO2 in mmHg) to fractional inspired oxygen (PaO2/FiO2 ratio)).

Primary outcome

Our main outcomes are the difference in the duration of invasive mechanical ventilation, defined as the time (days) from orotracheal intubation to complete orothracheal extubation, between the experimental and control groups. We selected this outcome since it directly compares the efficacy of early MRP to that of late MRP for diminishing the time to extubation.

Safety outcomes

Concerning safety, we will measure the following adverse events and compare their occurrence between groups:

- Altered blood pressure: Decrease in systolic arterial pressure >30% or mean arterial pressure >15%.
- Cardiac arrhythmia leading to hemodynamic instability.
- Oxygen desaturation: Oxygen saturation <80% for at least 10 minutes.
- Gastrointestinal complications: nausea, vomiting or diarrhea.
- Agitation: RASS > 2 requiring sedation.

- Removal of invasive line.
- Altered neurological status: Decrease in Glasgow Coma Scale (GCS) <8.
- Accidental extubation.
- Healthcare-associated pneumonia.
- Bronchoaspiration.

Secondary outcomes

We will also measure differences between the experimental and control groups regarding the following outcomes at T4 (up to day 28 after intubation in case the patient remains hospitalized or up to hospital discharge, in case the hospitalization period lasted less than 28 days):

- Number of ventilator-free days (up to 28 days).
- Number of days with delirium (up to 28 days).
- Number of days with delirium in the ICU (up to 28 days).
- Number of days with delirium in the general ward (up to 28 days).
- Number of days under sedation (up to 28 days).
- Number of days without sedation (up to 28 days).
- Extubation failure.
- Need for noninvasive mechanical ventilation.

Exploratory outcomes

We will explore the following outcomes:

- Time from intubation to starting the MRP
- Muscle strength 24 hours postextubation.
- The highest level of mobility 24 hours postextubation.
- Grip strength 24 hours postextubation.
- Dysphagia 72 hours postextubation.
- Dysphonia 72 hours postextubation.
- The time from extubation to restarting oral intake.
- Requirement of noninvasive mechanical ventilation postextubation.
- Number of days hospitalized.
- Number of days in the ICU.
- Barthel score 28 days postintubation/hospital discharge.
- Cognitive function 28 days postintubation/hospital discharge.
- Activities of daily life assessment 28 days postintubation/hospital discharge.
- Rapid sensory and dexterity test 28 days postintubation/hospital discharge.

- Incidence of all-cause mortality up to 90 days postintubation.

Participant timeline

The data will be collected at six time points: before allocation, at the beginning of mechanical ventilation weaning, during the OMAHA+ assessment, 24-72 days postextubation, at hospital discharge or on the 28th day after orotracheal intubation, and 90 days after intubation (Figs 1 & 2). The list of variables and their descriptions and categorizations can be found in S2 and S3 Files.

Mechanical ventilation weaning will start when the following pre-requisite are accomplished:

- The indication to start mechanical ventilation has been successfully addressed.
- Patient is hemodynamically stable or the vasopressor/inotropic support is diminishing (in case the patient is on vasopressor/inotropic support).
- The awakening has started (diminishing sedative medications)
- FIO2 < 40% and SpO2 > 89%

The OMAHA+ assessment is an institutional checklist used in our ICU to identify patients, currently in the weaning of mechanical ventilation support process, who can be successfully extubated. It includes the Yang and Tobin

index, along with additional clinical, hemodynamic, and ventilatory parameters that reduce the risk of extubating failure and reintubation. To initiate the extubating process, a patient must meet at least 90% of the criteria on the OMAHA+ checklist. A full description of the OMAHA+ assessment can be found in S6 File.

Sample size

To estimate the sample size, we included previous parameters published by Schweickert and colleagues [14]. They reported that the mean duration of mechanical ventilation in patients receiving usual physical therapy was 6.1 (±1.4) days compared to 3.4 (±1.25) days in a group of patients receiving early therapy. Given an alpha error of 0.05 and a beta error of 0.9, we estimated the sample size according to the following formula:

$$n = \frac{2\,(\frac{\mathrm{Z\alpha}}{2} + Z\beta)^2 \times (\mathrm{SD1}^2 + \mathrm{SD2}^2)}{\mathrm{ES}^2}$$

where n is the sample size, $\frac{Z\alpha}{2}$ is the critical value of the normal distribution at α/2 (2.81), $Z\beta$ is the Zβ is the Z score associated with the beta level (1.28), $SD1$ is the standard deviation in the group receiving usual physical therapy, and $SD2$

is the standard deviation in the group receiving early physical therapy [14]. The effect size (ES) was estimated as follows:

$$ES = \frac{|\mu 1 - \mu 2|}{\sqrt{\frac{SD1^2 - SD2^2}{2}}}$$

where $\mu 1$ is the mean duration of mechanical ventilation in the group receiving usual physical therapy, $\mu 2$ is the mean duration in the group receiving early physical therapy, and $SD1$ and $SD2$ are the same standard deviations used in the first equation. By substituting the values into the second equation, the ES equals to 2.03.

$$ES = \frac{|\ 6.1 - 3.4\ |}{\sqrt{\frac{1.40^2 - 1.25^2}{2}}} = 2.03$$

Therefore, the resulting equation obtained by replacing the values in the first equation is:

$$n = \frac{2\ (2.81 + 1.28)^2\ \times\ \ (1.40^2 +\ 1.25^2)}{2.03^2} = 30.29 \sim 31\ patients$$

We estimated 31 patients per intervention group (62 patients in total). An additional 20% (6 patients per group) were added to minimize the loss of

statistical power in the case of patients withdrawing from the study or missing data.

Methods: Assignment of interventions

Recruitment and allocation

In 2023, more than 600 mechanically ventilated patients were admitted to our ICU. Consequently, we anticipate recruiting approximately 15 to 20 patients per month, accounting for exclusion criteria and the willingness of legal representatives or patients to participate in the study.

Study participants will be randomly assigned to each group using a stratified randomization strategy to ensure balance by age (<65 and ≥65 years), sex (male and female), and comorbidities (Charlson comorbidity index <8 and ≥8) resulting in eight strata. The allocation sequence will be generated through a computer-generated random numbers system, a function provided by REDCap. Study participants will be irreversible assign by REDCap to either the intervention group or the control group once eligibility is confirmed. The randomization process will be monitored and documented electronically within REDCap, ensuring transparency and minimizing the potential for bias.

Additionally, REDCap will maintain a log of all randomization events, which can be audited if necessary.

Given the nature of our study, which involves applying an MRP, it is not feasible to blind the intervention to either the medical staff or the patients. However, to minimize bias, the assessment of the primary outcome will be performed by an independent evaluator who is blinded to the group assignments of the participants. This evaluator will be responsible for assessing the outcome without knowledge of which intervention was applied. The statistical analysis will be conducted by an independent researcher who will have access to the data but will not be involved in the outcome assessment.

Methods: Data collection, management, and analysis

Data collection and management

The data will be collected at six time points (Fig 1). The case report form (CRF) does not include any personal data, and every study participant will be given a randomized identification number to anonymize the data. The CRF includes demographic and clinical variables (information from T0) and information already registered in the institutional electronic medical records system. Information from time points 1 and 2 will be recorded by a respiratory therapist. Each therapist, together with the ICU research team, will record data at time

points 3 and 4. Finally, an ICU research team member will contact the patients through a phone call or their relatives to verify their health status 90 days after intubation (T5).

Both the clinical and research teams will receive training on the proper completion of the case report form (CRF) and other study forms, which can be consulted as S7 File. Clear procedures will be established to ensure consistent data collection among the teams. The following strategies will be implemented:

- Data Verification: Two members of the clinical team will verify any extreme or outlier values identified between T0 and T4 to ensure accuracy and determine whether these values reflect true clinical outcomes or potential data entry errors.
- Electronic CRF: An electronic CRF and other study forms will be used to capture hours and dates related to each specific study time point, facilitating the monitoring of adherence to the study protocol.

If a patient provides their informed consent and wishes to withdraw from the study, we will inquire about their reasons for discontinuing participation. Similarly, if a patient is withdrawn or deviates from the study protocol for any

reason, this information will be summarized and published in the final report and scientific paper. Nonetheless, we will perform an intention-to-treat analysis and a sensitivity analysis that includes patient data up until the time point where they revoked consent or deviated from the protocol. All patients who experience serious adverse events will be included in the analysis.

Data management

Study data will be recorded in an electronic CRF on the REDCap platform (https://www.project-redcap.org/) using the randomized identification number assigned to each study participant. Relevant information from the institutional electronic medical records system will be extracted and entered into the electronic CRF.

To ensure data quality and reliability, we will implement the following strategies:

- Data Entry Checking: Verification of consistency, incongruities, reliability, outliers, and missing CRF data at the time of data entry in REDCap.

- Double Data Entry: Two independent researchers will enter the data, and the resulting databases will be compared to identify any discrepancies.

Statistical analysis

The data analysts performing the statistical analysis will be blinded to group allocation. The statistical analysis will be conducted as an intention-to-treat analysis. The normality of the quantitative variables will be assessed using the Shapiro–Wilk test. Normally distributed variables will be presented as means (standard deviations), and non-normally distributed variables as medians (interquartile ranges). Qualitative variables will be reported as frequencies and proportions.

To compare continuous variables between the two groups, a T-test or Mann-Whitney U test will be used depending on the distribution of the data, and a $\chi 2$ or Fisher's exact test will be used for categorical variables.

The primary outcome (duration of mechanical ventilation) will be evaluated by comparing the means between the two groups using a T-test (or Mann-Whitney U test if non-normal distribution is present). Additionally, we will explore the primary outcome using survival analysis methods to provide a complementary

understanding of the time-based data. A survival analysis and Cox regression will be performed to account for time on mechanical ventilation, with the intervention group (early vs. late) as the independent variable. A survival curve will be generated, and differences between the two groups will be analyzed using a log-rank test.

For patients who are not extubated by the end of the follow-up period, the time on mechanical ventilation will be censored at the last point of observation (up to 28 days). The variable will be interpreted as the time until extubation for those successfully extubated, and as the maximum duration of ventilation observed for censored patients.

To account for patients with ICU stays shorter than 72 hours, we will conduct a sensitivity analysis excluding these patients to assess how their inclusion or exclusion affects the results. Additionally, the duration of ICU stay will be included as a covariate in the primary analysis to control for its potential impact on the outcome of mechanical ventilation duration. We will also perform a subgroup analysis comparing patients with ICU stays shorter than 72 hours to those with longer stays, as well as adherent against non-adherent patients to assess any differential effects on the primary outcome.

To evaluate factors related to the time of mechanical ventilation, univariable Cox regression models will be performed for all variables included in the study. Continuous variables will be evaluated for linearity through the Martingale residual plot, and if linearity is not met, stratification will be applied based on plot interpretation. Variables with a p-value <0.25 and clinically important variables (e.g., age, sex, delirium) will be included in a multivariable Cox regression model, and the best model will be selected through a Backward Stepwise process based on Akaike (AIC) and Bayesian (BIC) information criteria.

Data Verification: Two clinical team members will verify any extreme or outlier values identified between T0 and T4 to ensure accuracy and determine whether these values reflect true clinical outcomes or potential data entry errors. Extreme values will be evaluated using regression models to identify leverage points. If removing a leverage point does not significantly modify the model, the extreme value will remain in the analysis as it may provide valuable information about the data.

Potential confounding factors will be evaluated through a second multiple regression model. In this model, the potentially confounding variable will be removed, and the resulting slope or estimator (adjusted estimate) will be calculated. If there is a change greater than 15%-20%, the confounding variable will be included in the final model.

To evaluate the interaction between two variables, another regression model will be performed, including a new variable resulting from multiplying the two potentially interacting variables. If the regression coefficient of this new variable has a significance level of less than 0.05, it is concluded that there is an interaction, and this variable will be included in the model.

The assumption of proportional hazard will be evaluated using Schoenfeld residuals, and influential values will be analyzed through residuals versus observations plots. Multicollinearity will be assessed through matrix curves.

Logistic regression models will be used to assess the relationship between dichotomous variables such as failed extubation, post-extubation non-invasive ventilation, prolonged mechanical ventilation, tracheostomy, and dysphonia. The assumptions of a logistic regression model, such as linearity in the log of

the odds, independence of errors, and absence of multicollinearity, will be evaluated through residual and graphical diagnostics.

A multiplicity adjustment strategy will be employed in statistical tests, such as the Bonferroni method or Holm-Bonferroni, to maintain the overall type 1 error rate at an acceptable level. A detailed exploration will be conducted through a sensitivity analysis of possible interactions between identified subgroups (e.g., RASS scale, protocol adherence). If no statistically significant differences are found, a post-hoc power analysis will assess whether the sample size was sufficient to detect differences in the primary and secondary outcomes.

Patients withdrawn due to complications will remain in the analysis under the intention-to-treat principle. This approach ensures the study's feasibility and allows for robust analysis of both primary and secondary outcomes. Subgroup analyses will also be conducted to explore the potential impact of complications on the study results.

Dissemination

Dissemination

We are committed to the complete and transparent publication of all study results, whether positive or negative. Once the data are analyzed, the results will be published in a peer-reviewed journal and presented at both Colombian and international events.

We will include as authors every individual who meets the criteria and recommendations proposed by McNutt and colleagues [15]. Once the manuscript is prepared, it will be submitted to American Journal Experts for grammar, style, and spell checking. No additional editing services or artificial intelligence will be used for manuscript enhancement.

Trial status

We have not yet recruited the first patient. We plan to start in December 2024 and expect to finish in December 2025. The data will be stored in our institutional Redcap platform and will be accessible upon reasonable request.

Supporting information

S1 Checklist. SPIRIT 2013 Checklist.

(PDF)

S2 Protocol. The Spanish protocol was approved by the research ethics committee.

(PDF)

S3 Protocol. The study protocol translated to English.

(PDF)

S4 Document. Informed Consent in Spanish.

(PDF)

S5 Document. Informed Consent in English.

(PDF)

S6 Checklist. OMAHA+ Assessment Checklist.

(PDF)

S7 CRF. Case report form.

(PDF)

Author Contributions

All the authors contributed to the conception and design of the study. AFMS will be the clinical trial coordinator. LMCM, JIAS and AFMS will enrol study participants. All authors will conduct the randomization and allocation. AVMN and AFMS will perform the follow-up. LMCM, JIAS, and AFMS will write the final report. All authors contributed to the critical revision of the manuscript. All the authors reviewed this protocol and agreed on its publication.

Data Availability Statement: All data generated or analyzed during this study will be available upon study completion and reasonable request. If the data cannot be made publicly accessible, this will be clearly stated along with the reasons. All data are in the manuscript and/or supporting information files.

Funding: This research received no specific grant from any funding agency in the public, commercial or not-for-profit sectors.

Competing interests: None declared.

References

1. Schwitzer E, Jensen KS, Brinkman L, DeFrancia L, VanVleet J, Baqi E, et al. Survival ≠ Recovery. CHEST Critical Care. 2023 Jun;1(1):100003.
2. Rawal G, Yadav S, Kumar R. Post-intensive care syndrome: An overview. J Transl Int Med. 2017 Jun 30;5(2):90–2.

3. Parry SM, Nydahl P, Needham DM. Implementing early physical rehabilitation and mobilisation in the ICU: institutional, clinician, and patient considerations. Intensive Care Med. 2018 Apr 1;44(4):470–3.
4. Menges D, Seiler B, Tomonaga Y, Schwenkglenks M, Puhan MA, Yebyo HG. Systematic early versus late mobilization or standard early mobilization in mechanically ventilated adult ICU patients: systematic review and meta-analysis. Crit Care. 2021 Dec 1;25(1).
5. Castro-Avila AC, Serón P, Fan E, Gaete M, Mickan S. Effect of early rehabilitation during intensive care unit stay on functional status: Systematic review and meta-analysis. PLoS One. 2015 Jul 1;10(7).
6. Berry MJ, Morris PE. Early exercise rehabilitation of muscle weakness in acute respiratory failure patients. Exerc Sport Sci Rev. 2013 Oct;41(4):208–15.
7. Zhang L, Hu W, Cai Z, Liu J, Wu J, Deng Y, et al. Early mobilization of critically ill patients in the intensive care unit: A systematic review and meta-analysis. PLoS One. 2019 Oct 1;14(10).
8. Chan AW, Tetzlaff JM, Altman DG, Dickersin K, Moher D. SPIRIT 2013: new guidance for content of clinical trial protocols. The Lancet. 2013 Jan;381(9861):91–2.

9. World Medical Association. World Medical Association Declaration of Helsinki Ethical Principles for Medical Research Involving Human Subjects. JAMA [Internet]. 2013 Nov 27;310(20):2191. Available from: https://oxford.universitypressscholarship.com/view/10.1093/acprof:oso/9780199241323.001.0001/acprof-9780199241323-chapter-25

10. Eckstein S. The Belmont Report: ethical principles and guidelines for the protection of human subjects of research. In: Manual for Research Ethics Committees. Cambridge University Press; 2003. p. 126–32.

11. Ministerio de Salud. Resolución 8430 de 1993 [Internet]. Bogotá: Ministerio de Salud; Oct 4, 1993 p. 1–19. Available from: https://www.minsalud.gov.co/sites/rid/Lists/BibliotecaDigital/RIDE/DE/DIJ/RESOLUCION-8430-DE-1993.PDF

12. Congreso de la República. Ley 1581 de 2012 [Internet]. Por la cual se dictan disposiciones generales para la protección de datos personales. Bogotá: Congreso de Colombia; Oct 17, 2012 p. 1–9. Available from: https://www.funcionpublica.gov.co/eva/gestornormativo/norma.php?i=49981

13. Ministerio de Protección Social. Resolución No. 2378 de 2008 [Internet]. s Buenas Prácticas Clínicas para las instituciones que conducen investigación con medicamentos en seres humanos 2008. Available from:

https://www.alcaldiabogota.gov.co/sisjur/normas/Norma1.jsp?i=31169&dt=S

14. Schweickert WD, Pohlman MC, Pohlman AS, Nigos C, Pawlik AJ, Esbrook CL, et al. Early physical and occupational therapy in mechanically ventilated, critically ill patients: a randomised controlled trial. The Lancet. 2009 May;373(9678):1874–82.

15. McNutt MK, Bradford M, Drazen JM, Hanson B, Howard B, Jamieson KH, et al. Transparency in authors' contributions and responsibilities to promote integrity in scientific publication. Proceedings of the National Academy of Sciences. 2018 Mar 13;115(11):2557–60.

Letter to reviewers

Dear Editor,

Thank you for allowing us to send you the revised version of our study protocol. Below, you will find the responses to each of your requirements.

Journal Requirements:
When submitting your revision, we need you to address these additional requirements.

1. *Please ensure that your manuscript meets PLOS ONE's style requirements, including those for file naming. The PLOS ONE style templates can be found at https://journals.plos.org/plosone/s/file?id=wjVg/PLOSOne_formatting_sample_main_body.pdf and https://journals.plos.org/plosone/s/file?id=ba62/PLOSOne_formatting_sample_title_authors_affiliations.pdf*

We have reviewed the manuscript and supplemental material and adjusted to the PLOS ONE’s style requirements.

2. *Please provide a complete Data Availability Statement in the submission form, ensuring you include all necessary access information or a reason for why you are unable to make your data freely accessible. If your research concerns only data provided within your submission, please write "All data are in the manuscript and/or supporting information files" as your Data Availability Statement.*

Thank you for your valuable feedback. In response to your request regarding the Data Availability Statement, we have updated it as follows:

- Data Availability Statement: All data generated or analyzed during this study will be available upon study completion and reasonable request. If the data cannot be made publicly accessible, this will be clearly stated along with the reasons. Currently, all relevant data are included in the manuscript or supporting information files.

We hope this revision meets the requirements, and we remain available for any further clarification.

3. *Your ethics statement should only appear in the Methods section of your manuscript. If your ethics statement is written in any section besides the Methods, please delete it from any other section.*

Thank you for your feedback. We have made the necessary adjustments, and the ethics statement now appears only in the Methods section of the manuscript, as requested.

Reviewers' comments:

Reviewer's Responses to Questions
Comments to the Author

Reviewer #1: There are some major statistical problems in this protocol:

1. *A survival analysis is given as the data analysis technique for the primary outcome, yet the sample size is based on a t-test. The test of equality of survival curves must use sample size formulas based on survival data (e.g., Rosenberger and Lachin, Randomization in Clinical Trials, Wiley, 2016, Section 2.6.3).*

Thank you for your insightful feedback. We would like to clarify that our primary outcome, the duration of invasive mechanical ventilation, was conceptualized and analyzed as a quantitative variable in units of time (days), not as a time-to-event variable typically used in survival analysis. For this reason, we did not consider the survival analysis into the sample size estimation. We used a difference in means approach, which we deemed more appropriate for this outcome.

In the revised manuscript, we have clarified this distinction to ensure that the variable is correctly understood as quantitative (days), and not a time-to-event outcome (lines 366-370). Although our outcome could be perceived as time-dependent, we felt that analyzing it as a difference in means better aligned with the nature of the data and our research objectives.

We acknowledge your suggestion regarding the use of survival data formulas and will carefully consider this point in future analyses or similar studies

2. *The randomization procedure ("computer generated") is not given, nor a discussion of potential stratification, blocking, etc. (e.g., Rosenberger and Lachin, Randomization in Clinical Trials, Wiley, 2016, Chapter 3).*

Thank you for your valuable feedback. In response to your suggestion regarding the randomization procedure, in the protocol we included a stratified randomization strategy. Specifically, participants will be stratified by age (<65 and ≥65 years), sex (male and female) and comorbidities (Charlson comorbidity index <8 and ≥8) resulting in eight strata. We added his information in the manuscript (lines 493-496).

3. *Sealed envelopes were used in the 1948 streptomycin trial, but these days we use computer generated assignments available through Redcap or on a Shiny app.*

Thank you for your valuable suggestion regarding the randomization procedure. We changed the sealed envelopes to a random allocation sequence provided by REDCap (lines 493-503).

4. *The data analysts obviously cannot be blinded to the data! How can they do comparisons and test the primary outcome if they are blinded? What needs to be blinded is the assessment of the primary outcome, which should be done independently of those providing the intervention.*

Thank you for your valuable observation. We agree that it is not feasible to blind the data analysts to the data itself. To address your concern, we have clarified that the assessment of the primary outcome will be performed by an independent evaluator, who will be blinded to the group assignments (lines 507-512 and 568-569). This ensures that the outcome assessment is done objectively and independently from those providing the intervention. The data analysts will have access to the full dataset for the statistical analysis.

5. *Very complicated set of interventions, but if successful, the results will be interesting.*

Thank you for your thoughtful feedback. We appreciate your recognition of the complexity and potential impact of our study.

Reviewer #2: Manuscript PONE-D-24-31135

Title: Efficacy and Safety of the Early Implementation of a Multimodal Rehabilitation Program in Mechanically Ventilated Patients: A Randomized Clinical Trial Protocol

Thank you to the authors for a well-articulated and referenced manuscript. The study protocol requires additional methodological detail and consideration of the most appropriate outcome measure for this study. The manuscript will be publishable with moderate adjustments, particularly to the Introduction and Methods, in this reviewer's view.

The fundamental question for the authors in the study design is to consider whether starting the input two days (48hrs) apart is enough to create a measurable study effect, and whether the sample groups are large enough to observe this effect. For instance, tailoring interventions to the RASS score could result in participants from both groups receiving similar and, or low volumes of therapy because heavy sedation or agitation excludes them for two days of their stay from receiving 'active' treatments.

Please adjust the manuscript and or respond to the comments below.

Abstract:

1. *Please make it clearer for the reader as to how will the RASS score affect therapy input.*

Thank you for your helpful feedback. We have revised the abstract to clarify how the RASS score influences therapy input (lines 55-62). Specifically, therapy intensity and type are now explicitly adjusted according to the patient's RASS score, with passive interventions for

those with lower scores (indicating deeper sedation) and more active therapies for patients with higher scores (indicating lighter sedation or alertness). This adaptive approach ensures that the therapy is tailored to each patient's clinical condition (Table 1 and lines 224-236).

Introduction:

2. *The study justification as articulated in the Introduction needs rework. The primary outcome measure is not post-ICU outcomes as purported as the main reason for early intervention, it is time on the ventilator. Why would the investigators not use a primary measure that is a post-ICU physical or quality of life outcome, as justified in by the Introduction?*

Thank you for your valuable feedback. In response to your comment, we have revised the Introduction to clarify the justification for our study. Specifically, we have emphasized that the duration of mechanical ventilation is a key driver of adverse outcomes, such as those seen in post-intensive care syndrome (PICS) and is therefore an important target for early intervention. We have also highlighted how the primary outcome (time on the ventilator) aligns with the study's aim of reducing long-term complications by addressing this critical factor (lines 76-86).

Methods:

3. *Eligibility Criteria – requires additional detail and justification.*

Thank you for your feedback regarding the eligibility criteria. We have revised this section to provide additional detail and justification for each inclusion and exclusion criterion (lines 183-190). Specifically, we now clarify that:

- A Barthel score ≥70 ensures participants have a reasonable level of functional independence pre-ICU, allowing for accurate assessment of the rehabilitation intervention's impact.

- Invasive mechanical ventilation >24 hours ensures that patients have a sufficient duration of mechanical support to benefit from early rehabilitation.

4. *Will patients with ICU length of stay shorter than 72 hours be included? How will this be accounted for in the study and statistical analyses? How might this impact the efficacy of group allocation (randomisation)?*

Patients with ICU stays shorter than 72 hours will be included in the study. However, we recognize that shorter stays might impact their exposure to the full intervention. To address this:

- Primary Analysis: We will conduct an intention-to-treat analysis, where all randomized patients are analyzed in the groups they were assigned to, regardless of the length of their ICU stay or the level of intervention received.

- Secondary Analysis: We will perform a sensitivity analysis excluding patients with stays shorter than 72 hours to assess how this impacts the results. Additionally,

a subgroup analysis will compare outcomes between patients with longer and shorter ICU stays.

- Statistical Adjustments: The duration of ICU stay will be included as a covariate in the statistical models to control for its impact on the primary outcome.
- Randomization Impact: Randomization will occur upon ICU admission, ensuring that the group assignment is not biased by predicted ICU stay. We will assess balance between groups post-randomization and implement adjustments if necessary.

(See statistical analysis section).

5. *Line 181 - Will patients with isolated smoke inhalation injury (airway burn) be excluded? If so, why?*

Thank you for your question. Yes, patients with isolated smoke inhalation injury (airway burn) will be excluded from the study (line 198). It is well known that these patients often require prolonged mechanical ventilation due to the nature of their injury, which could introduce significant variability into our study outcomes. Given that the primary outcome is the duration of mechanical ventilation, including these patients could skew the results and make it difficult to assess the effects of the intervention on the general ICU population (https://doi.org/10.1042/CS20040135).

6. *Line 183 - Why are patients with COPD excluded?*

Thank you for your question. Patients with chronic obstructive pulmonary disease (COPD) are excluded from the study because they tend to require prolonged mechanical ventilation due to their underlying respiratory dysfunction. Including COPD patients could introduce variability in the primary outcome (duration of mechanical ventilation), making it more challenging to isolate the effects of the intervention on the general ICU population.

According to Sellares et al. (2011), COPD was identified as a significant predictor of prolonged weaning from mechanical ventilation, with an odds ratio of 2.97 (95% CI: 1.29–6.82). This finding further supports the exclusion of COPD patients to ensure that the study results are not biased by this group’s typically prolonged ventilation requirements.

Reference:

1. Sellares J, Ferrer M, Cano E, et al. Predictors of prolonged weaning and survival during ventilator weaning in a respiratory ICU. *Intensive Care Medicine*. 2011;37(5):775-784.

7. *Line 187 - How will life expectancy criteria be applied at recruitment?*

Thank you for your question. The life expectancy criterion (≤180 days) will be applied at recruitment based on a combination of factors, including:

1. Primary diagnosis and comorbidities, such as advanced cancer, end-stage heart failure, or other critical irreversible conditions.
2. Medical judgment of the ICU team, considering the patient's recent clinical course and overall prognosis.
3. Functional status, as measured by the Barthel index, which is already included in our study to assess the patient's ability to perform activities of daily living.

This multi-faceted approach will ensure that only patients who are likely to benefit from the intervention are included in the study, while avoiding potential bias introduced by including patients with very limited life expectancy (see exclusion criteria).

8. *Line 204 - Currently the daily number of treatments is not standardised. This reviewer strongly suggests that the number of intervention events be standardised to one or two per day per discipline to reduce study variability.*

9. *Similarly, the volume of treatment (minutes of input) applied per participant is a significant variable in this study. How will the investigators account for the daily differences?*

10. *Line 212 - Is there a better way to schedule the interventions to reduce variation in active treatment volume per patient? For instance, rather than accept that therapy is not completed or sessions markedly shortened due to periods of high sedation and agitation, could the investigators apply a RASS driven protocol which optimises the daily input (minimises down time) for the patient through the standardising treatment according to the strengths of different disciplines of the team?*

Thank you for your insightful feedback. In response to your comments, we have standardized the interventions while maintaining different volumes for each therapy (Table 1). Speech therapy will consist of two sessions of 10 minutes per day, while both physical therapy and respiratory therapy will involve two sessions of 30 minutes per day. Occupational therapy will be provided once daily for 30 minutes. We have also aligned the interventions with a RASS-driven protocol to optimize treatment and minimize downtime.

- For patients with a RASS ≤ -2, passive and passive-assisted interventions will focus on maintaining joint mobility, sensory input, and pulmonary function.
- For patients with a RASS between -1 and +1, more active rehabilitation will be provided, including motor activation, respiratory exercises, and cognitive tasks.
- For patients with a RASS ≥ 2, physical, speech, and respiratory therapies will continue as prescribed for the -1 to +1 group. However, occupational therapy will shift focus towards behavioral and environmental modulation to reduce agitation, incorporating sensory stimuli and family education to promote spatiotemporal orientation.

11. *Line 299 – How will adverse events be managed in the study analyses?*

Adverse events will be systematically recorded and categorized according to their severity and their relationship to the intervention. Serious adverse events include altered blood pressure, cardiac arrhythmia, oxygen desaturation, agitation requiring sedation, removal of invasive lines, altered neurological status, accidental extubation, healthcare-associated pneumonia, and bronchoaspiration. These events will be carefully monitored throughout the study.
Adverse events will be analyzed descriptively, with the frequency and type of events reported for each study group. In addition, an intention-to-treat (ITT) analysis will be applied for the primary outcome, ensuring that participants who are withdrawn due to serious adverse events remain in the analysis according to their original group assignment. Secondary safety analyses will be conducted to compare the incidence of adverse events between the groups and to assess the overall safety profile of the interventions (see Patient withdrawal and statistical analysis sections)

12. *Line 308 – Will the patient withdrawn due to complications no longer receive treatment in ICU? Is there any specific justification for why those with complications are withdrawn? Complications are relatively common in ICU and strictly applying this criteria may impact the sample size, feasibility of the study and ability to measure secondary outcomes with integrity.*

Thank you for your thoughtful questions. Patients withdrawn due to complications will continue to receive full treatment in the ICU as part of the standard care. The decision to withdraw them from the study is solely for research purposes and is based on the need to ensure their safety in relation to the study interventions. These patients will continue to be monitored and treated for their clinical needs, but they will no longer participate in the specific study interventions if a serious adverse event occurs. They will continue receiving any or all therapies according to the judgement of the critical medicine and intensive care team (physicians, therapists and nursing staff), but the frequency and intensity may vary compared to the multimodal rehabilitation program proposed in our study (additional reason to exclude them from the study).

The justification for withdrawing patients with complications is to prevent any potential harm or exacerbation of their condition as a result of the study interventions. We acknowledge that complications are relatively common in the ICU; however, we aim to balance patient safety with the integrity of the study.

Importantly, patients who are withdrawn will still be included in the analysis based on an intention-to-treat approach. This ensures that the sample size is preserved, and the feasibility and ability to measure secondary outcomes are maintained without compromising the safety of participants.

We have also planned to conduct subgroup analyses to explore the impact of complications on the study outcomes, which will provide further insights without compromising the integrity of the data. (see Patient withdrawal and statistical analysis sections)

13. *Line 314 – This criterion is troublesome as the treatment is not standardised. How will non-compliance be confirmed and at what 'amount' of deviation from the protocol will constitute need for withdrawal? More detail needed please.*

Thank you for your insightful feedback. We have removed that withdrawal criterion. Instead, we have added information about how patients will be monitored regarding their adherence to the intervention (lines 313-340). Additionally, we will make a subgroup analysis of adherent compared to non-adherent patients. It is important to mention that non-compliance and protocol deviations are serious research violations, and those terms do not apply to participant's adherence to the intervention (https://research.umbc.edu/protocol-deviationsor-issues-of-non-compliance/). We follow current Good Clinical Practice guidelines, and we adhere to the Helsinki Declaration and the Colombian Legislation.

14. *Line 328 – How will transfers to another facility be treated? Will their data prior to transfer be included in the study?*

Thank you for your question. Data from patients transferred to another facility will be retained and if appropriate, it will be included in the intention to treat analysis. If the proportion of missing data due to the transfer is less than 20%, we will use data imputation methods to account for the missing values. This ensures that the contribution of these patients is not lost and that the sample size and statistical power are preserved. Transfers will be recorded as a separate variable, and we will explore their potential impact on the study outcomes through sensitivity analyses (line 293-301).

Outcomes:

15. *Line 356 – how often will ventilator parameters be collected and how will they be included in the Statistical Analyses?*

Thank you for your question. Ventilator parameters will be collected at three times. First, these parameters will be collected after randomization, ensuring that all patients start under comparable conditions. Second, they will be collected when the critical medicine and intensive care group decide that the patient can start the mechanical ventilation weaning (lines 432-439). Finally, the ventilation parameters will be recorded during the OMAHA assessment (just before extubating).

For the statistical analysis, we will perform comparisons using mean or median differences, depending on the distribution of the data. This will help us confirm that there are no

significant differences in ventilator settings between the groups at the start of the study, at the beginning of the mechanical ventilation weaning and before extubating (see outcomes section and figure 2).

16. *The primary outcome is not justified by the Introduction as it focusses on post-ICU acquired weakness, PICS, physical and quality of life outcomes. It would seem more likely that the primary outcome has been chosen for convenience and not related to the substance of the study and planned interventions?*

Thank you for your insightful feedback. We have revised the Introduction to better align it with the primary outcome of duration of mechanical ventilation. The updated Introduction now highlights the importance of early rehabilitation interventions in improving respiratory function and reducing the duration of mechanical ventilation. This outcome was chosen not only for its direct clinical relevance but also because reducing mechanical ventilation duration can contribute to better long-term outcomes, such as a reduction in post-ICU acquired weakness and PICS.

By emphasizing the link between the interventions and the immediate benefits on respiratory recovery, we believe the primary outcome is well-justified in the context of the study.

17. *Line 371 – cardiac arrythmias are mentioned as a reason for withdrawal earlier. How will adverse events be delineated as an occurrence to be recorded, or result in participant withdrawal which will affect the sample size and power of the trial analyses?*

Thank you for your question. Adverse events, such as cardiac arrhythmias leading to hemodynamic instability, will be recorded and monitored. It is important to highlight that to be withdraw due to a cardiac arrhythmia; this complication must lead to hemodynamic instability. Patients experiencing serious adverse events may be withdrawn for safety reasons. However, they will continue to receive full ICU care.

Data up to the point of withdrawal will be included in the intention-to-treat analysis, ensuring minimal impact on sample size and study power. We will also conduct sensitivity analyses to assess any potential impact of these withdrawals on the study outcomes. Additionally, we added 12 patients (20%) to the sample size. Adding these patients minimizes the risk of losing statistical power due to participants' withdrawal

18. *Line 384 – The criteria 28 days after intubation or hospital discharge is ambiguous. Please clarify.*

Thank you for your question. To clarify, all outcomes will be measured up to day 28 after intubation in case the patient remains hospitalized or up to hospital discharge, in case the hospitalization period lasted less than 28 days. We included this endpoint in order to maximize the comparability among patients who were discharge early (< 28 days after

intubation) and those who remained in the hospital (≥ 28 days). There may be some patients whose hospitalization period could last several weeks or even months, and compare all patients at the moment of discharge could distort the results at T4 (lines 490-493).

Participant Timeline

19. *Line 414 – is there a typo – 24-72 hours post-extubation? Sample Size. 20.*

Thank you for your observation. To clarify, we are indeed referring to 24-72 hours post-intubation, during which we will measure functional outcomes, incidence of dysphagia, dysphonia, delirium, ARDS, adverse events, and mortality. These time points are critical for assessing early outcomes related to the interventions. Please refer to Figures 1 and 2 for more details on the timeline and specific outcomes measured during this period.
We appreciate your attention to this detail and will ensure it is clearly stated in the revised manuscript.

20. *Line 438 – 440 – is there a typo related to the group description for SD1 and SD2?*

Thank you for your observation. After reviewing the text, we realized there is indeed a typo in the description of SD1 and SD2. SD1 should refer to the standard deviation in the group receiving early physical therapy, while SD2 should refer to the standard deviation in the group receiving late physical therapy. We will correct this in the revised manuscript to ensure clarity (lines 460-464)
We appreciate your careful attention to detail.

21. *The primary outcome is measured in hours, however the sample size has been calculated using days. Does this make a difference to the mathematics of the calculation?*

Thank you for your observation. We have already corrected this issue, and the primary outcome is now consistently measured and calculated in days throughout the study, including the sample size calculation. This ensures consistency in the methodology and avoids any discrepancies in the analysis.

22. *The sample size seems particularly low for an ICU study. There are a multitude of variables which could affect the time ventilated (including the patient diagnosis or reason for critical care), the standardisation of the interventions and cause withdrawal of a patient according to the proposed criteria. Has the mathematics of this sample size calculation been applied to account for so many variables which may not be managed effectively by stratification of random allocation? Please revisit.*

Thank you for your thoughtful comment. We recognize this limitation, especially when considering the impact of the intervention on other outcomes such as:

- Number of ventilator-free days (up to 28 days).
- Number of days with delirium (up to 28 days).

- Number of days with delirium in the ICU (up to 28 days).
- Number of days with delirium in the general ward, among others.

However, we plan to conduct a post-hoc power analysis upon reaching the target sample size, particularly for outcomes where statistical differences are not observed. This approach has been outlined in the statistical analysis section of the protocol to ensure that any potential limitations in statistical power are accounted for in our final assessment (lines 646-652).

Recruitment and Allocation

23. *Stratification for age has been applied. Why not also for diagnosis? Data collection and management*

Thank you for your question. We have stratified for age because it is a well-established factor that significantly impacts outcomes in the ICU. However, stratifying for diagnosis was not included due to the wide variety of diagnoses and the complexity it would add to the stratification process. Instead, we plan to control for diagnosis through multivariable regression analyses in the statistical analysis phase. This will allow us to account for the potential influence of different diagnoses without complicating the randomization process.

24. *is unclear how long a patient needs to remain in the study before their case is considered as part of the sample. For instance, with short ICU length of stay, will there be adequate time for the interventions to be applied and impact the outcome?*

Thank you for your important question. We recognize that ICU length of stay may vary significantly among patients. To address this:

1. All patients will be included in the study and analyzed under the intention-to-treat principle, regardless of their ICU length of stay. This ensures that every patient's data contributes to the overall analysis.
2. For patients with short ICU stays, we acknowledge that the time may be insufficient for the intervention to have its full intended effect. Therefore, we will conduct a sensitivity analysis to explore how these patients impact the outcomes. This will allow us to assess whether their inclusion affects the interpretation of the study results.

We hope this clarifies our approach to managing patients with varying ICU lengths of stay (see statistical analysis).

25. *Line 501 – Data verification – this statement reads like a statistical 'get out of jail free' card. Why would extreme values be assessed for verification if not to exclude them from the study analysis? Please clarify the intent of this action in the study.*

Thank you for your question. The verification of extreme values is not intended to exclude them from the analysis arbitrarily. Instead, we will assess extreme values through regression models that allow us to identify leverage points and evaluate whether removing them would significantly alter the model. If the extreme value does not modify the model, it will be kept in the analysis, as it may help explain important aspects of the data.

Additionally, we will verify all extreme values to ensure that any extreme values reflect true clinical situations and do not represent data entry errors. By using this rigorous approach, we ensure that extreme values are handled appropriately without compromising the integrity of the study

26. Line 508 - ? typo (tense) – please check expression.

Thank you for pointing out the typo. The correct expression should be: "If a patient provides their informed consent and wishes to withdraw from the study, we will inquire about their reasons for discontinuing participation."
We corrected this in the revised manuscript (lines 540-541).

Statistical Analysis

27. Exactly which variables will be carried forward using the intention-to-treat principles?

Thank you for your question. In the intention-to-treat (ITT) analysis, we will carry forward the variables that are key to the outcomes observed over the 90-day period, specifically:

- Primary outcome: Duration of mechanical ventilation.
- Secondary outcomes, including:
    - Number of ventilator-free days (up to 28 days).
    - Incidence of delirium, sedation days, and adverse events such as dysphagia, dysphonia, ARDS, and mortality (assessed up to 90 days).

Only these outcomes will be included in the ITT analysis, ensuring the most relevant data are included despite any early withdrawal (see figure 1 and 2).

28. Line 544 - Please explain the risk variable. If the patient is not extubated, how can the time of mechanical ventilation be used in the proposed variable? How is this model interpreted? Also, as time on the ventilator is the primary variable, how can it be included as a co-variate in the same statistical model?

Thank you for your valuable feedback. We agree that time on mechanical ventilation is our primary outcome and will not be included as a covariable in the statistical model.
For patients who are not extubated by the end of the follow-up period, we will censor their time on ventilation at the last point of observation (up to 28 days). This means that their data will be included up to the moment of censoring, reflecting the maximum duration of ventilation recorded during the study period.
The variable will be interpreted as the time until extubation for those successfully extubated, and for those censored, it will represent the maximum duration of ventilation observed without extubation. This approach ensures that all patients are included in the analysis while maintaining the integrity of the data.
We appreciate your input and will reflect these adjustments in the revised manuscript.

29. *The statistical modelling is complex and involves not only known variables but also interaction terms and ad hoc tests. Is the relatively small sample size with repeated measures longitudinal design likely to support an interpretable analysis for any of the secondary outcomes?*

Thank you for your observation. We acknowledge the complexity of the statistical modeling, especially given the relatively small sample size. To address this concern, we have already planned to conduct a post-hoc power analysis to assess whether the sample size is sufficient to detect differences in both primary and secondary outcomes.
Additionally, if certain analyses prove difficult to interpret due to the sample size or complexity, we will adjust our models accordingly, potentially simplifying the analysis or conducting exploratory analyses for the secondary outcomes. These considerations are outlined in the statistical analysis section.

Figures

30. Figure 1 – The interventions subsection doesn't add value or needs adjustment to demonstrate that the group allocation (time and volume of interventions) is the key variable in the study.

Thank you for your valuable feedback. We have adjusted Figure 1 to better emphasize that the key variable in the study is the group allocation, specifically in terms of timing and volume of interventions. The figure now clearly shows the differences in the timing of interventions (Early MRP within 24 hours and Late MRP after 72 hours) as well as the standardized frequency and duration of interventions for each group. A clarification has been added in the figure's footnote to reflect these adjustments.
