# Supplementary material for: Efficacy and safety of the early implementation of a multimodal rehabilitation program in mechanically ventilated patients: A randomized clinical trial protocol
Source: PLoS One. 2025 May 19;20(5):e0324335. doi: 10.1371/journal.pone.0324335 (PMC12088510; doi:10.1371/journal.pone.0324335)
Supplement: Attachment — Submitted filename: Letter to reviewer.docx [file pone.0324335.s008.docx]

**Letter to reviewers**

**Dear Editor,**

Thank you for allowing us to send you the revised version of our study protocol. Below, you will find the responses to each of your requirements.

Journal Requirements:

When submitting your revision, we need you to address these additional requirements.

1. *Please ensure that your manuscript meets PLOS ONE's style requirements, including those for file naming. The PLOS ONE style templates can be found at*
   [*https://journals.plos.org/plosone/s/file?id=wjVg/PLOSOne_formatting_sample_main_body.pdf*](https://journals.plos.org/plosone/s/file?id=wjVg/PLOSOne_formatting_sample_main_body.pdf) *and* 
   [*https://journals.plos.org/plosone/s/file?id=ba62/PLOSOne_formatting_sample_title_authors_affiliations.pdf*](https://journals.plos.org/plosone/s/file?id=ba62/PLOSOne_formatting_sample_title_authors_affiliations.pdf)

We have reviewed the manuscript and supplemental material and adjusted to the PLOS ONE’s style requirements.

2. *Please provide a complete Data Availability Statement in the submission form, ensuring you include all necessary access information or a reason for why you are unable to make your data freely accessible. If your research concerns only data provided within your submission, please write "All data are in the manuscript and/or supporting information files" as your Data Availability Statement.*

Thank you for your valuable feedback. In response to your request regarding the Data Availability Statement, we have updated it as follows:

- Data Availability Statement: All data generated or analyzed during this study will be available upon study completion and reasonable request. If the data cannot be made publicly accessible, this will be clearly stated along with the reasons. Currently, all relevant data are included in the manuscript or supporting information files.

We hope this revision meets the requirements, and we remain available for any further clarification.

3. *Your ethics statement should only appear in the Methods section of your manuscript. If your ethics statement is written in any section besides the Methods, please delete it from any other section.*

Thank you for your feedback. We have made the necessary adjustments, and the ethics statement now appears only in the Methods section of the manuscript, as requested.

Reviewers' comments:

Reviewer's Responses to Questions

Comments to the Author

Reviewer #1: There are some major statistical problems in this protocol:

1. *A survival analysis is given as the data analysis technique for the primary outcome, yet the sample size is based on a t-test. The test of equality of survival curves must use sample size formulas based on survival data (e.g., Rosenberger and Lachin, Randomization in Clinical Trials, Wiley, 2016, Section 2.6.3).*

Thank you for your insightful feedback. We would like to clarify that our primary outcome, the duration of invasive mechanical ventilation, was conceptualized and analyzed as a quantitative variable in units of time (days), not as a time-to-event variable typically used in survival analysis. For this reason, we did not consider the survival analysis into the sample size estimation. We used a difference in means approach, which we deemed more appropriate for this outcome.

In the revised manuscript, we have clarified this distinction to ensure that the variable is correctly understood as quantitative (days), and not a time-to-event outcome (lines 366-370). Although our outcome could be perceived as time-dependent, we felt that analyzing it as a difference in means better aligned with the nature of the data and our research objectives.

We acknowledge your suggestion regarding the use of survival data formulas and will carefully consider this point in future analyses or similar studies

2. *The randomization procedure ("computer generated") is not given, nor a discussion of potential stratification, blocking, etc. (e.g., Rosenberger and Lachin, Randomization in Clinical Trials, Wiley, 2016, Chapter 3).*

Thank you for your valuable feedback. In response to your suggestion regarding the randomization procedure, in the protocol we included a stratified randomization strategy. Specifically, participants will be stratified by age (<65 and ≥65 years), sex (male and female) and comorbidities (Charlson comorbidity index <8 and ≥8) resulting in eight strata. We added his information in the manuscript (lines 493-496).

3. *Sealed envelopes were used in the 1948 streptomycin trial, but these days we use computer generated assignments available through Redcap or on a Shiny app.*

Thank you for your valuable suggestion regarding the randomization procedure. We changed the sealed envelopes to a random allocation sequence provided by REDCap (lines 493-503).

4. *The data analysts obviously cannot be blinded to the data! How can they do comparisons and test the primary outcome if they are blinded? What needs to be blinded is the assessment of the primary outcome, which should be done independently of those providing the intervention.*

Thank you for your valuable observation. We agree that it is not feasible to blind the data analysts to the data itself. To address your concern, we have clarified that the assessment of the primary outcome will be performed by an independent evaluator, who will be blinded to the group assignments (lines 507-512 and 568-569). This ensures that the outcome assessment is done objectively and independently from those providing the intervention. The data analysts will have access to the full dataset for the statistical analysis.

5. *Very complicated set of interventions, but if successful, the results will be interesting.*

Thank you for your thoughtful feedback. We appreciate your recognition of the complexity and potential impact of our study.

Reviewer #2: Manuscript PONE-D-24-31135

Title: Efficacy and Safety of the Early Implementation of a Multimodal Rehabilitation Program in Mechanically Ventilated Patients: A Randomized Clinical Trial Protocol

Thank you to the authors for a well-articulated and referenced manuscript. The study protocol requires additional methodological detail and consideration of the most appropriate outcome measure for this study. The manuscript will be publishable with moderate adjustments, particularly to the Introduction and Methods, in this reviewer’s view.

The fundamental question for the authors in the study design is to consider whether starting the input two days (48hrs) apart is enough to create a measurable study effect, and whether the sample groups are large enough to observe this effect. For instance, tailoring interventions to the RASS score could result in participants from both groups receiving similar and, or low volumes of therapy because heavy sedation or agitation excludes them for two days of their stay from receiving ‘active’ treatments.

Please adjust the manuscript and or respond to the comments below.

Abstract:
*1. Please make it clearer for the reader as to how will the RASS score affect therapy input.*

Thank you for your helpful feedback. We have revised the abstract to clarify how the RASS score influences therapy input (lines 55-62). Specifically, therapy intensity and type are now explicitly adjusted according to the patient’s RASS score, with passive interventions for those with lower scores (indicating deeper sedation) and more active therapies for patients with higher scores (indicating lighter sedation or alertness). This adaptive approach ensures that the therapy is tailored to each patient’s clinical condition (Table 1 and lines 224-236).

Introduction:

2. *The study justification as articulated in the Introduction needs rework. The primary outcome measure is not post-ICU outcomes as purported as the main reason for early intervention, it is time on the ventilator. Why would the investigators not use a primary measure that is a post-ICU physical or quality of life outcome, as justified in by the Introduction?*

Thank you for your valuable feedback. In response to your comment, we have revised the Introduction to clarify the justification for our study. Specifically, we have emphasized that the duration of mechanical ventilation is a key driver of adverse outcomes, such as those seen in post-intensive care syndrome (PICS) and is therefore an important target for early intervention. We have also highlighted how the primary outcome (time on the ventilator) aligns with the study’s aim of reducing long-term complications by addressing this critical factor (lines 76-86).

Methods:

3. *Eligibility Criteria – requires additional detail and justification.*

Thank you for your feedback regarding the eligibility criteria. We have revised this section to provide additional detail and justification for each inclusion and exclusion criterion (lines 183-190). Specifically, we now clarify that:

- A Barthel score ≥70 ensures participants have a reasonable level of functional independence pre-ICU, allowing for accurate assessment of the rehabilitation intervention’s impact.
- Invasive mechanical ventilation >24 hours ensures that patients have a sufficient duration of mechanical support to benefit from early rehabilitation.

4. *Will patients with ICU length of stay shorter than 72 hours be included? How will this be accounted for in the study and statistical analyses? How might this impact the efficacy of group allocation (randomisation)?*

Patients with ICU stays shorter than 72 hours will be included in the study. However, we recognize that shorter stays might impact their exposure to the full intervention. To address this:

- Primary Analysis: We will conduct an intention-to-treat analysis, where all randomized patients are analyzed in the groups they were assigned to, regardless of the length of their ICU stay or the level of intervention received.
- Secondary Analysis: We will perform a sensitivity analysis excluding patients with stays shorter than 72 hours to assess how this impacts the results. Additionally, a subgroup analysis will compare outcomes between patients with longer and shorter ICU stays.
- Statistical Adjustments: The duration of ICU stay will be included as a covariate in the statistical models to control for its impact on the primary outcome.
- Randomization Impact: Randomization will occur upon ICU admission, ensuring that the group assignment is not biased by predicted ICU stay. We will assess balance between groups post-randomization and implement adjustments if necessary.

(See statistical analysis section).

5. *Line 181 - Will patients with isolated smoke inhalation injury (airway burn) be excluded? If so, why?*

Thank you for your question. Yes, patients with isolated smoke inhalation injury (airway burn) will be excluded from the study (line 198). It is well known that these patients often require prolonged mechanical ventilation due to the nature of their injury, which could introduce significant variability into our study outcomes. Given that the primary outcome is the duration of mechanical ventilation, including these patients could skew the results and make it difficult to assess the effects of the intervention on the general ICU population (<https://doi.org/10.1042/CS20040135>).

6. *Line 183 - Why are patients with COPD excluded?*

Thank you for your question. Patients with **chronic obstructive pulmonary disease (COPD)** are excluded from the study because they tend to require **prolonged mechanical ventilation** due to their underlying respiratory dysfunction. Including COPD patients could introduce variability in the primary outcome (duration of mechanical ventilation), making it more challenging to isolate the effects of the intervention on the general ICU population.

According to **Sellares et al. (2011)**, COPD was identified as a significant predictor of **prolonged weaning** from mechanical ventilation, with an odds ratio of **2.97** (95% CI: 1.29–6.82). This finding further supports the exclusion of COPD patients to ensure that the study results are not biased by this group’s typically prolonged ventilation requirements.

**Reference:**

1. Sellares J, Ferrer M, Cano E, et al. Predictors of prolonged weaning and survival during ventilator weaning in a respiratory ICU. Intensive Care Medicine. 2011;37(5):775-784.
7. *Line 187 - How will life expectancy criteria be applied at recruitment?*

Thank you for your question. The life expectancy criterion (≤180 days) will be applied at recruitment based on a combination of factors, including:

1. Primary diagnosis and comorbidities, such as advanced cancer, end-stage heart failure, or other critical irreversible conditions.
2. Medical judgment of the ICU team, considering the patient’s recent clinical course and overall prognosis.
3. Functional status, as measured by the Barthel index, which is already included in our study to assess the patient’s ability to perform activities of daily living.

This multi-faceted approach will ensure that only patients who are likely to benefit from the intervention are included in the study, while avoiding potential bias introduced by including patients with very limited life expectancy (see exclusion criteria).

8. *Line 204 - Currently the daily number of treatments is not standardised. This reviewer strongly suggests that the number of intervention events be standardised to one or two per day per discipline to reduce study variability.*
9. *Similarly, the volume of treatment (minutes of input) applied per participant is a significant variable in this study. How will the investigators account for the daily differences?*
10. *Line 212 - Is there a better way to schedule the interventions to reduce variation in active treatment volume per patient? For instance, rather than accept that therapy is not completed or sessions markedly shortened due to periods of high sedation and agitation, could the investigators apply a RASS driven protocol which optimises the daily input (minimises down time) for the patient through the standardising treatment according to the strengths of different disciplines of the team?*

Thank you for your insightful feedback. In response to your comments, we have standardized the interventions while maintaining different volumes for each therapy (Table 1). Speech therapy will consist of two sessions of 10 minutes per day, while both physical therapy and respiratory therapy will involve two sessions of 30 minutes per day. Occupational therapy will be provided once daily for 30 minutes. We have also aligned the interventions with a RASS-driven protocol to optimize treatment and minimize downtime.

- For patients with a RASS ≤ -2, passive and passive-assisted interventions will focus on maintaining joint mobility, sensory input, and pulmonary function.
- For patients with a RASS between -1 and +1, more active rehabilitation will be provided, including motor activation, respiratory exercises, and cognitive tasks.
- For patients with a RASS ≥ 2, physical, speech, and respiratory therapies will continue as prescribed for the -1 to +1 group. However, occupational therapy will shift focus towards behavioral and environmental modulation to reduce agitation, incorporating sensory stimuli and family education to promote spatiotemporal orientation.

11. *Line 299 – How will adverse events be managed in the study analyses?*

Adverse events will be systematically recorded and categorized according to their severity and their relationship to the intervention. Serious adverse events include altered blood pressure, cardiac arrhythmia, oxygen desaturation, agitation requiring sedation, removal of invasive lines, altered neurological status, accidental extubation, healthcare-associated pneumonia, and bronchoaspiration. These events will be carefully monitored throughout the study.

Adverse events will be analyzed descriptively, with the frequency and type of events reported for each study group. In addition, an intention-to-treat (ITT) analysis will be applied for the primary outcome, ensuring that participants who are withdrawn due to serious adverse events remain in the analysis according to their original group assignment. Secondary safety analyses will be conducted to compare the incidence of adverse events between the groups and to assess the overall safety profile of the interventions (see Patient withdrawal and statistical analysis sections)

12. *Line 308 – Will the patient withdrawn due to complications no longer receive treatment in ICU? Is there any specific justification for why those with complications are withdrawn? Complications are relatively common in ICU and strictly applying this criteria may impact the sample size, feasibility of the study and ability to measure secondary outcomes with integrity.*

Thank you for your thoughtful questions. Patients withdrawn due to complications will continue to receive full treatment in the ICU as part of the standard care. The decision to withdraw them from the study is solely for research purposes and is based on the need to ensure their safety in relation to the study interventions. These patients will continue to be monitored and treated for their clinical needs, but they will no longer participate in the specific study interventions if a serious adverse event occurs. They will continue receiving any or all therapies according to the judgement of the critical medicine and intensive care team (physicians, therapists and nursing staff), but the frequency and intensity may vary compared to the multimodal rehabilitation program proposed in our study (additional reason to exclude them from the study).

The justification for withdrawing patients with complications is to prevent any potential harm or exacerbation of their condition as a result of the study interventions. We acknowledge that complications are relatively common in the ICU; however, we aim to balance patient safety with the integrity of the study.

Importantly, patients who are withdrawn will still be included in the analysis based on an intention-to-treat approach. This ensures that the sample size is preserved, and the feasibility and ability to measure secondary outcomes are maintained without compromising the safety of participants.

We have also planned to conduct subgroup analyses to explore the impact of complications on the study outcomes, which will provide further insights without compromising the integrity of the data. (see Patient withdrawal and statistical analysis sections)

13. *Line 314 – This criterion is troublesome as the treatment is not standardised. How will non-compliance be confirmed and at what ‘amount’ of deviation from the protocol will constitute need for withdrawal? More detail needed please.*

Thank you for your insightful feedback. We have removed that withdrawal criterion. Instead, we have added information about how patients will be monitored regarding their adherence to the intervention (lines 313-340). Additionally, we will make a subgroup analysis of adherent compared to non-adherent patients. It is important to mention that non-compliance and protocol deviations are serious research violations, and those terms do not apply to participant’s adherence to the intervention (<https://research.umbc.edu/protocol-deviationsor-issues-of-non-compliance/>). We follow current Good Clinical Practice guidelines, and we adhere to the Helsinki Declaration and the Colombian Legislation.

14. *Line 328 – How will transfers to another facility be treated? Will their data prior to transfer be included in the study?*

Thank you for your question. Data from patients transferred to another facility will be retained and if appropriate, it will be included in the intention to treat analysis. If the proportion of missing data due to the transfer is less than 20%, we will use data imputation methods to account for the missing values. This ensures that the contribution of these patients is not lost and that the sample size and statistical power are preserved. Transfers will be recorded as a separate variable, and we will explore their potential impact on the study outcomes through sensitivity analyses (line 293-301).

Outcomes:

15. *Line 356 – how often will ventilator parameters be collected and how will they be included in the Statistical Analyses?*

Thank you for your question. Ventilator parameters will be collected **at three times. First, these parameters will be collected after randomization**, ensuring that all patients start under comparable conditions. Second, they will be collected when the critical medicine and intensive care group decide that the patient can start the mechanical ventilation weaning (lines 432-439). Finally, the ventilation parameters will be recorded during the OMAHA assessment (just before extubating).

For the statistical analysis, we will perform comparisons using **mean or median differences**, depending on the distribution of the data. This will help us confirm that there are no significant differences in ventilator settings between the groups at the start of the study, at the beginning of the mechanical ventilation weaning and before extubating (see outcomes section and figure 2).

16. *The primary outcome is not justified by the Introduction as it focusses on post-ICU acquired weakness, PICS, physical and quality of life outcomes. It would seem more likely that the primary outcome has been chosen for convenience and not related to the substance of the study and planned interventions?*

Thank you for your insightful feedback. We have revised the Introduction to better align it with the primary outcome of duration of mechanical ventilation. The updated Introduction now highlights the importance of early rehabilitation interventions in improving respiratory function and reducing the duration of mechanical ventilation. This outcome was chosen not only for its direct clinical relevance but also because reducing mechanical ventilation duration can contribute to better long-term outcomes, such as a reduction in post-ICU acquired weakness and PICS.

By emphasizing the link between the interventions and the immediate benefits on respiratory recovery, we believe the primary outcome is well-justified in the context of the study.

17. *Line 371 – cardiac arrythmias are mentioned as a reason for withdrawal earlier. How will adverse events be delineated as an occurrence to be recorded, or result in participant withdrawal which will affect the sample size and power of the trial analyses?*

Thank you for your question. Adverse events, such as cardiac arrhythmias leading to hemodynamic instability, will be recorded and monitored. It is important to highlight that to be withdraw due to a cardiac arrhythmia; this complication must lead to hemodynamic instability. Patients experiencing serious adverse events may be withdrawn for safety reasons. However, they will continue to receive full ICU care.

Data up to the point of withdrawal will be included in the intention-to-treat analysis, ensuring minimal impact on sample size and study power. We will also conduct sensitivity analyses to assess any potential impact of these withdrawals on the study outcomes. Additionally, we added 12 patients (20%) to the sample size. Adding these patients minimizes the risk of losing statistical power due to participants’ withdrawal

18. *Line 384 – The criteria 28 days after intubation or hospital discharge is ambiguous. Please clarify.*

Thank you for your question. To clarify, all outcomes will be measured up to day 28 after intubation in case the patient remains hospitalized or up to hospital discharge, in case the hospitalization period lasted less than 28 days. We included this endpoint in order to maximize the comparability among patients who were discharge early (< 28 days after intubation) and those who remained in the hospital (≥ 28 days). There may be some patients whose hospitalization period could last several weeks or even months, and compare all patients at the moment of discharge could distort the results at T4 (lines 490-493).

Participant Timeline

19. *Line 414 – is there a typo – 24-72 hours post-extubation?
    Sample Size. 20.*

Thank you for your observation. To clarify, we are indeed referring to 24-72 hours post-intubation, during which we will measure functional outcomes, incidence of dysphagia, dysphonia, delirium, ARDS, adverse events, and mortality. These time points are critical for assessing early outcomes related to the interventions. Please refer to Figures 1 and 2 for more details on the timeline and specific outcomes measured during this period.

We appreciate your attention to this detail and will ensure it is clearly stated in the revised manuscript.

20. *Line 438 – 440 – is there a typo related to the group description for SD1 and SD2?*

Thank you for your observation. After reviewing the text, we realized there is indeed a typo in the description of SD1 and SD2. SD1 should refer to the standard deviation in the group receiving early physical therapy, while SD2 should refer to the standard deviation in the group receiving late physical therapy. We will correct this in the revised manuscript to ensure clarity (lines 460-464)

We appreciate your careful attention to detail.

21. *The primary outcome is measured in hours, however the sample size has been calculated using days. Does this make a difference to the mathematics of the calculation?*

Thank you for your observation. We have already corrected this issue, and the primary outcome is now consistently measured and calculated in days throughout the study, including the sample size calculation. This ensures consistency in the methodology and avoids any discrepancies in the analysis.

22. *The sample size seems particularly low for an ICU study. There are a multitude of variables which could affect the time ventilated (including the patient diagnosis or reason for critical care), the standardisation of the interventions and cause withdrawal of a patient according to the proposed criteria. Has the mathematics of this sample size calculation been applied to account for so many variables which may not be managed effectively by stratification of random allocation? Please revisit.*

Thank you for your thoughtful comment. We recognize this limitation, especially when considering the impact of the intervention on other outcomes such as:

- Number of ventilator-free days (up to 28 days).
- Number of days with delirium (up to 28 days).
- Number of days with delirium in the ICU (up to 28 days).
- Number of days with delirium in the general ward, among others.

However, we plan to conduct a post-hoc power analysis upon reaching the target sample size, particularly for outcomes where statistical differences are not observed. This approach has been outlined in the statistical analysis section of the protocol to ensure that any potential limitations in statistical power are accounted for in our final assessment (lines 646-652).

Recruitment and Allocation

23. *Stratification for age has been applied. Why not also for diagnosis?
    Data collection and management*

Thank you for your question. We have stratified for age because it is a well-established factor that significantly impacts outcomes in the ICU. However, stratifying for diagnosis was not included due to the wide variety of diagnoses and the complexity it would add to the stratification process. Instead, we plan to control for diagnosis through multivariable regression analyses in the statistical analysis phase. This will allow us to account for the potential influence of different diagnoses without complicating the randomization process.

24. *is unclear how long a patient needs to remain in the study before their case is considered as part of the sample. For instance, with short ICU length of stay, will there be adequate time for the interventions to be applied and impact the outcome?*

Thank you for your important question. We recognize that ICU length of stay may vary significantly among patients. To address this:

1. All patients will be included in the study and analyzed under the intention-to-treat principle, regardless of their ICU length of stay. This ensures that every patient’s data contributes to the overall analysis.
2. For patients with short ICU stays, we acknowledge that the time may be insufficient for the intervention to have its full intended effect. Therefore, we will conduct a sensitivity analysis to explore how these patients impact the outcomes. This will allow us to assess whether their inclusion affects the interpretation of the study results.

We hope this clarifies our approach to managing patients with varying ICU lengths of stay (see statistical analysis).

25. *Line 501 – Data verification – this statement reads like a statistical ‘get out of jail free’ card. Why would extreme values be assessed for verification if not to exclude them from the study analysis? Please clarify the intent of this action in the study.*

Thank you for your question. The verification of extreme values is not intended to exclude them from the analysis arbitrarily. Instead, we will assess extreme values through regression models that allow us to identify leverage points and evaluate whether removing them would significantly alter the model. If the extreme value does not modify the model, it will be kept in the analysis, as it may help explain important aspects of the data.

Additionally, we will verify all extreme values to ensure that any extreme values reflect true clinical situations and do not represent data entry errors. By using this rigorous approach, we ensure that extreme values are handled appropriately without compromising the integrity of the study

26. *Line 508 - ? typo (tense) – please check expression.*

Thank you for pointing out the typo. The correct expression should be:
"If a patient provides their informed consent and wishes to withdraw from the study, we will inquire about their reasons for discontinuing participation."

We corrected this in the revised manuscript (lines 540-541).

Statistical Analysis

27. *Exactly which variables will be carried forward using the intention-to-treat principles?*

Thank you for your question. In the intention-to-treat (ITT) analysis, we will carry forward the variables that are key to the outcomes observed over the 90-day period, specifically:

- Primary outcome: Duration of mechanical ventilation.
- Secondary outcomes, including:
  - Number of ventilator-free days (up to 28 days).
  - Incidence of delirium, sedation days, and adverse events such as dysphagia, dysphonia, ARDS, and mortality (assessed up to 90 days).

Only these outcomes will be included in the ITT analysis, ensuring the most relevant data are included despite any early withdrawal (see figure 1 and 2).

28. *Line 544 - Please explain the risk variable. If the patient is not extubated, how can the time of mechanical ventilation be used in the proposed variable? How is this model interpreted? Also, as time on the ventilator is the primary variable, how can it be included as a co-variate in the same statistical model?*

Thank you for your valuable feedback. We agree that time on mechanical ventilation is our primary outcome and will not be included as a covariable in the statistical model.

For patients who are not extubated by the end of the follow-up period, we will censor their time on ventilation at the last point of observation (up to 28 days). This means that their data will be included up to the moment of censoring, reflecting the maximum duration of ventilation recorded during the study period.

The variable will be interpreted as the time until extubation for those successfully extubated, and for those censored, it will represent the maximum duration of ventilation observed without extubation. This approach ensures that all patients are included in the analysis while maintaining the integrity of the data.

We appreciate your input and will reflect these adjustments in the revised manuscript.

29. *The statistical modelling is complex and involves not only known variables but also interaction terms and ad hoc tests. Is the relatively small sample size with repeated measures longitudinal design likely to support an interpretable analysis for any of the secondary outcomes?*

Thank you for your observation. We acknowledge the complexity of the statistical modeling, especially given the relatively small sample size. To address this concern, we have already planned to conduct a post-hoc power analysis to assess whether the sample size is sufficient to detect differences in both primary and secondary outcomes.

Additionally, if certain analyses prove difficult to interpret due to the sample size or complexity, we will adjust our models accordingly, potentially simplifying the analysis or conducting exploratory analyses for the secondary outcomes. These considerations are outlined in the statistical analysis section.

Figures
*30. Figure 1 – The interventions subsection doesn’t add value or needs adjustment to demonstrate that the group allocation (time and volume of interventions) is the key variable in the study.*

Thank you for your valuable feedback. We have adjusted Figure 1 to better emphasize that the key variable in the study is the group allocation, specifically in terms of timing and volume of interventions. The figure now clearly shows the differences in the timing of interventions (Early MRP within 24 hours and Late MRP after 72 hours) as well as the standardized frequency and duration of interventions for each group. A clarification has been added in the figure’s footnote to reflect these adjustments.
