# Supplementary material for: Efficacy and safety of the early implementation of a multimodal rehabilitation program in mechanically ventilated patients: A randomized clinical trial protocol
Source: PLoS One. 2025 May 19;20(5):e0324335. doi: 10.1371/journal.pone.0324335 (PMC12088510; doi:10.1371/journal.pone.0324335)
Supplement: Attachment — Submitted filename: Letter.docx [file pone.0324335.s010.docx]

Letter to reviewer

Thank you for allowing us to send you the revised version of our study protocol. Below, you will find the responses to each of your requirements.

1. Thank you for making these adjustments. It is important that the interventions are tailored to behavior eg agitation, not simply a RASS score as patient alertness does not equate directly with cooperation with therapy.

**Response:** Thank you for your thoughtful observation. We recognize that tailoring interventions to patient behavior, such as agitation or cooperation, is crucial. As detailed in the RASS-Driven Protocol (lines 248-258) the RASS score serves as a foundational guide, but therapy interventions are further adjusted in real-time based on the patient’s behavior and readiness to cooperate. This ensures a personalized approach that considers both sedation levels and the patient’s behavioral state.

To enhance clarity, we have updated the abstract to reflect this nuance, ensuring consistency with the protocol and manuscript (lines 61-64). We appreciate your attention to this important detail.

2. This response is remarkably challengeable as much as it relates to the choice of outcome which is convenient.

Is mechanical ventilation a key driver of adverse outcomes or is it a marker of other factors which are far more likely to correlate with complications and adverse outcomes, especially post-ICU syndrome? For example, to be mechanically ventilated requires sedation and muscle relaxants as well as a cocktail of other pharmacological interventions which are likely to be primary drivers of post-ICU syndrome. Further, bed rest associated with mechanical ventilation is likely to be implicated as a direct cause of multiple adverse events.

Is there a specific reason that the authors are avoiding a more contemporary approach with a patient centric measure, particularly one that would be a marker of impact or severity of post-ICU syndrome, as their primary outcome?

**Response:**

Thank you for your insightful feedback. We acknowledge the nuanced relationship between mechanical ventilation and adverse outcomes such as post-ICU syndrome (PICS). While mechanical ventilation itself may not be the sole driver of these complications, it is a significant surrogate marker of critical illness severity and a key modifiable factor in ICU management. As such, its duration is a clinically relevant outcome that aligns with our study's aim to mitigate long-term complications through early multimodal rehabilitation.

We have clarified in the manuscript that mechanical ventilation duration reflects not only the time spent on ventilatory support but also the cumulative effects of associated interventions, such as sedation, neuromuscular blockade, and immobility, all of which contribute to PICS. By targeting a reduction in ventilator days, we aim to address these underlying factors, thereby indirectly influencing post-ICU outcomes.

The choice of this primary outcome also considers practical and methodological reasons. Mechanical ventilation duration is:

- **Objective and measurable**: Unlike quality-of-life measures, which are often subjective and influenced by a range of confounding factors, ventilator duration is directly linked to ICU management strategies and provides a clear endpoint for evaluating the efficacy of early rehabilitation.
- **Proven relevance**: Studies, including those cited in the manuscript, have demonstrated that reducing ventilator days is associated with improved muscle strength, earlier mobilization, and potentially better long-term recovery.

We recognize the value of more patient-centric outcomes, particularly those that assess the severity or impact of PICS. To address this, we have included secondary and exploratory outcomes, such as post-extubation muscle strength, Barthel Index scores, and quality-of-life assessments at 28 and 90 days post-intubation. These outcomes will provide a broader perspective on the long-term impact of our intervention while complementing the primary outcome.

We have revised the Introduction to explicitly address these points, emphasizing the rationale for our primary outcome while acknowledging its relationship to other factors driving adverse outcomes. Additionally, we highlight the inclusion of patient-centric measures as secondary endpoints to capture the broader impact of early rehabilitation.

We appreciate your thoughtful comment, which has helped refine and strengthen the study’s justification

3. This response is self contradicting in the context of the intervention. Would the authors not be interested in determining if their intervention was effective in reducing mechanical ventilation time for those with prolonged critical care support needs?

**Response:** Thank you for this insightful comment. We recognize the potential value of evaluating the intervention in patients with prolonged critical care support needs, such as those with isolated smoke inhalation injuries. However, our decision to exclude these patients was based on several considerations.

First, in our clinical setting, patients with airway burns often have concurrent extensive burns involving large areas of body surface. This adds significant complexity to their clinical course, including increased risk of infections, fluid imbalances, and metabolic demands, which further prolong mechanical ventilation and ICU stays. These factors introduce considerable variability that could confound the study outcomes and obscure the true effects of the multimodal rehabilitation program in the broader ICU population.

Second, the pathophysiology and clinical management of patients with airway burns differ substantially from those without these injuries. The severe airway inflammation, necrosis, and obstruction typical in these cases often require prolonged ventilatory support, independent of rehabilitation interventions. Including such a group might bias the results and make it challenging to assess the efficacy of our intervention for the general ICU population.

Finally, our primary goal in this trial is to evaluate the intervention in a more homogenous population where factors influencing the duration of mechanical ventilation—such as immobility and sedation practices—are modifiable through early rehabilitation. Including highly complex subpopulations like these could dilute the observed effects of the intervention.

That said, we acknowledge the importance of assessing the efficacy of our program in subpopulations with unique critical care needs. The findings from this study will help establish a foundation for future research to explore the program’s potential benefits in patients with airway burns and other complex conditions.

4. Thank you - please add a short justification with this reference to the Exclusion criteria.

**Response:** The justification for excluding COPD patients has been added to the exclusion criteria as requested (lines 215-217).

5. Thank you - please summarize this additional detail into the Exclusion criteria section.

**Response:** Thank you for your comment. We have summarized the additional details regarding the life expectancy criterion into the exclusion criteria section, specifying that patients with a life expectancy of ≤180 days will be excluded based on their primary diagnosis, comorbidities, recent clinical course, medical judgment by the ICU team, and functional status as assessed by the Barthel index (lines 222-225).

6. Could the Clavien-Dindo classifications be an appropriate framework to use in this instance?

**Response:** Thank you for your comment. The Clavien-Dindo classification has been exclusively used to categorize surgical complications. Thus, this classification is inappropriate for our study. We decided to use the Common Terminology Criteria for Adverse Events classification which has been used primarily in oncological studies, but it is also used in other context such as clinical trials (lines 270-302).

7. This is important however, if the primary outcome remains time on the ventilator, how will the ITT principle be able to be applied if the patient is withdrawn while still ventilated?

Also, if the adverse event is deemed by the treating intensive care physician NOT to be related to the multimodal rehab, why would the patient be removed from the study and an adverse reaction recorded as associated with the study intervention?

**Response:** Thank you for your insightful question. The intention-to-treat (ITT) principle will still be applied to ensure the integrity of the primary outcome, even for patients who are withdrawn while still ventilated. Specifically:

a) **Primary Outcome (Time on Ventilator):** For patients withdrawn due to complications, their time on the ventilator will be recorded up until the point of withdrawal. This data will be included in the analysis as censored observations, acknowledging that the true duration of ventilation could not be fully captured due to withdrawal. This approach ensures that their contribution to the primary outcome is preserved without biasing the results.
b) **Statistical Handling:** In the analysis, survival methods such as Kaplan-Meier estimates and Cox regression models will be used to account for censored data. This ensures that the duration of ventilation for withdrawn patients is appropriately incorporated without compromising the ITT principle or the validity of the results.
c) **Subgroup and Sensitivity Analyses:** Additional analyses will explore the potential impact of withdrawn patients on the study outcomes. These analyses will help determine whether their withdrawal introduces any systematic differences and ensure the robustness of the study conclusions.

Not all participants who experience an adverse event or complication will be withdrawn from the study. In the literature, most common adverse events in clinical trials are grade 1 and 2. Therefore, participants experiencing minor adverse events should not be withdrawn from the study solely for experiencing an adverse event, but based on an analysis of the adverse event. Nonetheless they will be informed regarding the adverse event and ask if they want to continue to participate. Severe adverse events will be analyzed by the ethics committee and they will determine if the participant continues in the study (lines 292-307).

By combining these methods, we aim to uphold the ITT principle while balancing patient safety and maintaining the validity of the primary outcome analysis. Thank you for raising this critical point. It has helped clarify our approach to managing these cases.

8. Thank you for these changes. There are multiple non-patient related reasons for why a participant does not adhere to a research study protocol - including staffing in the ICU; surgery; procedures including central line changes and insertions; and, poorly controlled sedation practices. To exclude patients from the study for reasons such as these may impact the study sample size greatly and thus, it would be an enhancement to the design if it was adjusted to maintain patients with deviations from protocol which are related to such events which are beyond the control of the patient and the researchers.

**Response:** Thank you for your thoughtful feedback. We completely agree that deviations from the protocol due to factors beyond the control of the patient or researchers, such as ICU staffing limitations, surgeries, procedures, or sedation management, should not lead to automatic withdrawal. To address this concern, we have revised our protocol to account for these scenarios and ensure the study sample size is maintained without compromising the integrity of the analysis (lines 381-411).

9. Is there any reason why these data couldn't be recorded daily? For example, with the morning physiotherapy treatment, record the ventilator settings.

**Response:** Thank you for your thoughtful suggestion. We agree that collecting ventilator parameters daily, particularly in conjunction with the morning physiotherapy treatment, could provide valuable insights into the dynamic evolution of mechanical ventilation and its relationship with the intervention.

To enhance the study design while balancing feasibility, we propose the following adjustments:

a) **Daily Collection:** Ventilator parameters will be recorded daily, at a consistent time, ideally before the physiotherapy session. This will ensure that data are standardized and reflective of the patient's baseline respiratory status before the intervention (lines 426-429).
b) **Expanded Analysis:** These additional data points will be analyzed as an exploratory outcome to assess trends in ventilator support requirements over time and their relationship to the intervention. This could provide further insights into the impact of the program on ventilatory dependency (lines 490-506).
c) **Primary Outcome Integrity:** The daily recordings will complement the primary outcome analysis without introducing additional complexity, as the primary comparison of baseline ventilator settings will still focus on demonstrating equivalence between groups.

By implementing this adjustment, we aim to enrich the study data without overburdening the clinical team. Thank you for highlighting this opportunity to strengthen our research methodology.

10. Please add these explanations into the Statistical Analysis methods.

**Response:** We have incorporated these explanations into the Statistical Analysis methods section for clarity and transparency (lines 654-733).

11. Reference please.

**Response:** We added a reference in the introduction (Na SJ, Ko R-E, Nam J, Ko MG, Jeon K. Factors associated with prolonged weaning from mechanical ventilation in medical patients. Ther Adv Respir Dis. 2022;16. doi:10.1177/17534666221117005).

12. Reference please. As noted, mechanical ventilation is a convenient indicator for statistical purposes, but is it the best measure to identify the impact of an early MRP?

**Response:** Thank you for your observation. Mechanical ventilation was chosen as an indicator due to its well-documented utility in critically ill patients as a proxy for disease severity and the progression of organ dysfunction. While we acknowledge that mechanical ventilation may not be the sole or perfect measure to evaluate the impact of an early MRP, it provides a quantifiable, objective, and clinically relevant endpoint. Moreover, its association with ICU outcomes makes it a convenient and practical indicator for statistical analyses in this context.

13. Is this exclusion applied if the patient has had H&N surgery at any time in the past or within last 2 years prior to admission? How will acute H&N grafting surgery during the ICU admission affect the study participation? Will the patient be excluded mid-protocol?

**Response:** Thank you for your thoughtful question. In response, we have refined the exclusion criterion and clarified our approach to managing cases involving head and neck (H&N) surgeries:

- Exclusion Criterion: Patients with a history of head and neck surgery at any time prior to ICU admission will be excluded from the study. This is based on the well-documented risk of dysphagia and/or bronchoaspiration associated with head and neck surgeries, which could confound the interpretation of our results by introducing variables unrelated to the MRP being evaluated (line 211).
- Management of Acute Head and Neck Surgeries During ICU Stay:

Patients who undergo acute head and neck surgery during their ICU stay will not be withdrawn from the study. Instead, these cases will be managed as handling minor deviations/external factors:

- Deviations due to such surgeries, along with other ICU procedures (e.g., central line insertions), staffing limitations, or uncontrolled sedation practices, will not lead to participant withdrawal.
- These events will be documented as protocol deviations and addressed in sensitivity analyses to evaluate their potential impact on study outcomes.

This approach ensures adherence to the intention-to-treat principle while maintaining the study’s methodological integrity. By excluding patients with a prior history of head and neck surgery and managing intra-study surgeries as deviations, we aim to minimize confounding variables and ensure a robust analysis of the MRP’s effects.

14. Is this exclusion applied if the patient has had acute coronary syndrome at any time in the past or within a period prior to admission? How will acute cardiac arrest during the ICU admission affect the study participation? Will the patient be excluded mid-protocol?

**Response:** Thank you for your valuable question. We have clarified the exclusion criteria and provided additional details regarding the management of patients with acute coronary syndrome (ACS) or cardiac arrest to ensure transparency and alignment with the study's objectives (line 212).

Patients with a history of ACS will not be excluded from the study unless the ACS directly led to ICU admission due to cardiac arrest. This clarification ensures that participants with prior or stable cardiac conditions are included, preserving a representative ICU population and enhancing the generalizability of the study findings. In contrast, patients admitted to the ICU specifically because of cardiac arrest will be excluded. This is due to the unique rehabilitation and clinical outcomes associated with cardiac arrest, which could confound the interpretation of the study results.

For patients who experience cardiac arrest during their ICU stay, we have determined that these cases will not lead to withdrawal from the study. Instead, such events will be categorized as adverse events and their association with the interventions will be analyzed. Deviations related to cardiac arrest, such as altered rehabilitation schedules or changes in sedation practices, will be documented. To ensure the robustness of the results, these deviations will be thoroughly evaluated through sensitivity analyses to assess their potential impact on the study outcomes.

By explicitly clarifying the exclusion criteria and addressing intra-study cardiac arrest events as adverse events, this approach minimizes unnecessary exclusions while adhering to the intention-to-treat principle. It allows for a realistic evaluation of the MRP within the complex ICU environment, balancing methodological rigor with clinical relevance.

We appreciate your insightful feedback and believe these clarifications strengthen the study protocol. Should you have additional suggestions or require further elaboration, we are happy to provide further details.

15. Presumably this exclusion will only be applied at study eligibility assessment for recruitment?

**Response:** Thank you for your question. The exclusion criterion for demyelinating diseases or neuromuscular junction disorders applies exclusively during the initial eligibility assessment at the time of study recruitment (lines 219-220).

16. Presumably this is not routine practice in the study ICU?

**Response:** Thank you for your question. The use of neuromuscular blockade is not routine practice in the study ICU; it is reserved for specific clinical situations such as severe acute respiratory distress syndrome (ARDS) or refractory hypoxemia. As such, this exclusion criterion applies only to a limited subset of patients and is not expected to significantly impact study recruitment.

17. Please add an example to aid justification of this catch all exclusion criteria.

**Response:** Thank you for your suggestion. We have removed the exclusion criterion, "Patients who may not benefit from multimodal therapy according to medical criteria," as our existing inclusion and exclusion criteria already adequately address this consideration. This ensures clarity and avoids redundancy in patient selection.

18. How will this exclusion criteria be applied for those who have been readmitted to ICU within 28 days of the their acute burn? How will the outcome measures be adjusted if this were the case?

**Response:** Thank you for your question. Our exclusion criteria specifically apply to patients with burns involving more than 50% of total body surface area or burns of the airway. These criteria are applied at the time of initial ICU admission, ensuring that patients meeting these exclusions are not enrolled in the study.

In the case of readmission to the ICU within 28 days following an acute burn, the exclusion criteria would still apply, as the original diagnosis and clinical status would remain unchanged. Therefore, such patients would not be included in the study population.

It is also worth noting that, based on our 2023 ICU data, we admitted only eight ventilated burn patients during the year. All of these patients had burns involving more than 50% of their total body surface area and/or burns of the airway, which fall within our exclusion criteria. This further reinforces the low probability of including burn patients in this study.

19. Wouldn't the exclusion of all cancer diagnoses receiving active treatment at the eligibility assessment be a more applicable criteria? Why is this very specific procedure identified as an exclusion from a multimodal physical intervention when it is likely that all those receiving active treatment for cancer will likely respond variably or inconsistently to the study intervention?

**Response:** Thank you for your thoughtful suggestion. We agree that excluding all patients with a cancer diagnosis receiving active treatment may provide a more consistent and homogeneous study population. Therefore, we will modify the exclusion criterion to encompass all patients actively receiving cancer treatment, as these individuals are likely to respond variably to the multimodal intervention due to the effects of both their disease and ongoing therapies (lines 229-230).

20. Please change 'at each' to 'according to'.

**Response:** Thank you for your suggestion. The requested change from "at each" to "according to" has been made in the manuscript (line 253).

21. Please add - RASS score 'recorded at or most recent prior to, the commencement of treatment'.

**Response:** This change has been made (lines 252-255)

22. One would argue that this decision needs to be more nuanced and as a result of a documented discussion with the attending physician about whether the adverse event was intervention related?

**Response:** Thank you for your insightful comment. We agree that the decision to withdraw participants should be more nuanced and involve input from the attending physician. To address this, we have revised the protocol to specify that the decision to withdraw participants will be made in consultation with the attending physician and based on a documented discussion to determine whether the adverse event is directly related to the study-specific intervention (lines 292-297)

23. How many patients (with adverse events related to the intervention) would constitute making an early termination decision?

**Response:** Thank you for your question. There is no specific number of patients required to early terminate the study. This decision will be made by an Independent Data Monitoring and Safety Committee based on the number of adverse events, their severity, its association with the intervention, among other factors. The Committee could paused the study while investigating a single serious adverse event.

Additionally, there will be three moments when the study’s safety will be evaluated:

a) After reporting each serious adverse event
b) During the interim analyses (the first analysis will be performed after recruiting 33% of the sample and the second after reaching 66%)
c) During the Ethics Committee study audits (twice per year).

Although we cannot state a threshold for the number of adverse events, the study will continuously be monitored to early identify adverse events related to the intervention and be paused or terminated if necessarily.

24. Please reconsider this choice. This reviewer argues that it is not reflective of the ethos of the study intervention and the authors should be brave in their choice of outcomes which should be patient centric and far more likely to be directly linked to allied health input than time on the ventilator

**Response:**

This is addressed in response 2.

25. At what point in the OMAHA assessment might the failure of extubation be decided?

**Response:** The OMAHA assessment is designed to evaluate readiness for extubation by ensuring that patients meet at least 90% of the checklist criteria, which includes clinical, ventilatory, and hemodynamic parameters. Failure of extubation might be decided during or immediately after the assessment if the patient does not meet the required criteria for extubation readiness, such as insufficient respiratory strength, poor oxygenation, or unstable hemodynamics. Additionally, if a patient is extubated but exhibits signs of respiratory failure (e.g., increased work of breathing, hypoxemia, or hypercapnia), extubation failure would be recognized post-extubation during close monitoring.

26. How many of these patients were burn patients?

**Response:** Out of the 600 patients admitted to our ICU in 2023, only 8 were burn patients.

27. Would there be 15 - 20 eligible burn patients per month admitted to the facility ICU?

**Response:** No, this number significantly exceeds the actual admissions of burn patients requiring mechanical ventilation in our ICU. Based on our 2023 data, we admitted only 8 ventilated burn patients over the entire year. This corresponds to less than one ventilated burn patient per month on average, making it highly unlikely to have 15-20 eligible ventilated burn patients per month.

28. The Charlson comorbidity index is less sensitive than the Elixhauser index. Consider using the latter.

**Response:** Thank you for your suggestion. While we recognize the advantages of the Elixhauser index, we have chosen the Charlson comorbidity index for this study due to its widespread use, ease of implementation, and established predictive value in critically ill patients.

29. Is this analysis viable with a censorship date of 28 days and only 62 patients in total planned for the study sample?

**Response:** Thank you for raising this important point. We acknowledge that the combination of a small sample size (74 patients) and a 28-day censorship date may limit the statistical power to detect significant differences, particularly for time-to-event outcomes. To address this, we have already planned to employ Kaplan-Meier survival analysis and Cox regression models with censoring, which are well-suited for small sample sizes and time-dependent outcomes (lines 673-677). Patients who remain mechanically ventilated at the end of the 28-day period or are withdrawn while still ventilated will be treated as censored observations, ensuring adherence to the intention-to-treat principle.

Additionally, ventilator-free days, although an imperfect outcome, are a common outcome measure in ICU studies and is reported up to 28 days in the literature (Yehya N, Harhay MO, Curley MAQ, Schoenfeld DA, Reeder RW. Reappraisal of Ventilator-Free Days in Critical Care Research. Am J Respir Crit Care Med. 2019 Oct 1;200(7):828-836. doi: 10.1164/rccm.201810-2050CP).

We appreciate your thoughtful feedback, which has helped us clarify and strengthen the statistical framework of our study.
